# Fungal communities in sediments of subtropical Chinese seas as estimated by DNA metabarcoding

**DOI:** 10.1038/srep26528

**Published:** 2016-05-20

**Authors:** Wei Li, Meng Meng Wang, Xi Guang Wang, Xiao Li Cheng, Jia Jia Guo, Xiao Meng Bian, Lei Cai

**Affiliations:** 1 College of Marine Life Sciences, Ocean University of China, Qingdao 266003, China; 2 Institute of Agricultural Information, Chinese Academy of Agricultural Sciences, Beijing 100081, China; 3 State Key Laboratory of Mycology, Institute of Microbiology, Chinese Academy of Sciences, Beijing 100101, China

## Abstract

Ribosomal RNA internal transcribed spacer-1 (ITS1) metabarcoding was used to investigate the distribution patterns of fungal communities and the factors influencing these patterns in subtropical Chinese seas, including the southern and northern Yellow Sea and the Bohai Sea. These seas were found to harbor high levels of fungal diversity, with 816 operational taxonomic units (OTUs) that span 130 known genera, 36 orders, 14 classes and 5 phyla. Ascomycota was the most abundant phylum, containing 72.18% and 79.61% of all OTUs and sequences, respectively, followed by Basidiomycota (19.98%, 18.64%), Zygomycota (1.10%, 0.11%), Chytridiomycota (0.25%, 0.04%) and Rozellomycota (0.12%, 0.006%). The compositions of fungal communities across these three sea regions were found to be vary, which may be attributed to sediment source, geographical distance, latitude and some environmental factors such as the temperature and salinity of bottom water, water depth, total nitrogen, and the ratio of total organic carbon to nitrogen. Among these environmental factors, the temperature of bottom water is the most important driver that governs the distribution patterns of fungal communities across the sampled seas. Our data also suggest that the cold-water mass of the Yellow Sea likely balances competitive relationships between fungal taxa rather than increasing species richness levels.

As subtropical Chinese seas, both the Yellow Sea (YS) and Bohai Sea (BHS) are semi-enclosed continental seas of the northwestern Pacific Ocean in northern China^1^ (Fig. 1). The YS, composed of the northern Yellow Sea (NYS) and the southern Yellow Sea (SYS), is commonly considered one of the broadest marginal seas in the world. The physical and biotic environments of these sea regions have been greatly affected by Korean and Chinese rivers that carry abundant organic materials and terrestrial organisms^2^,^3^. In addition, as marginal seas of China, these seas have been profoundly influenced by the economic development of coastal areas, and their ecosystems have clearly been degraded. As a result, the biodiversity of the YS and BHS regions is declining for various reasons, particularly overfishing and environmental deterioration (pollution and coastal construction) resulting from anthropogenic activity in recent years^3^.

Previously reported data on the distribution of several plant and animal species indicate that species diversity levels decrease distinctly from the SYS to the NYS and BHS^1^,^4^,^5^. In the central region of the YS, a cold water mass (CWM) formed in water remains within a bottom layer under the thermocline at 15–30 m depth in summer, playing a critical role in the maintenance of high levels of local bottom fauna biodiversity^1^. Recently, through the use of molecular tools, researchers have found that the diversity and community compositions of planktonic nanociliates^5^ and bacteria^6^ in these sea regions have been profoundly influenced by biogeographic distance and environmental factors, particularly the temperature of bottom water (TBW). Fungi represent an important group of microbes living in marine ecosystems that play a critical role in the decomposition and mineralization of organic matter^7^,^8^. Moreover, temperature has been regarded as an important driver of fungal biogeography in marine environments^8^,^9^. Thus, new questions have been raised regarding how the CWM of the YS influences fungal communities inhabiting the northern Chinese seas and how the YS is associated with ecological importance of fungi in marine ecosystems.

Over the past decade, molecular tools have been widely used to estimate fungal diversity levels and numerous fungal taxa have been retrieved from marine various environments^10^, such as deep-sea sediments^11^,^12^,^13^,^14^,^15^,^16^, costal water and sediments^17^, and marine invertebrates^18^ among others. However, no data of molecular survey data on fungal diversity levels in these subtropical China seas, i.e., the SYS, NYS and BHS, are currently available. This lack of information on fungal diversity and distribution patterns has hindered the conservation and utilization of fungal resources. Using ITS1 metabarcoding, the current study aims to address the following questions: 1) What are the fungal diversity and distribution patterns characteristic of these seas? 2) Are the effects of spatial and environmental factors on fungal communities similar to those of other organisms (for CWM in particular)?

## Results

### Taxonomic assignment and community compositions

In total, 122,448 raw reads with a median length of 321 bases were obtained from 30 samples with pyrosequencing on one 1/4-plate. After the filtering process, 102,990 quality-filtered ITS1 reads assigned to 3,404 OTUs with 1,056 singletons were found from 30 samples. After the OTUs with fewer than five reads were removed, 816 OTUs (58.67%) of 79,847 reads (80.37%) were assigned to the fungal kingdom and 575 OTUs (41.33%) of 19,498 reads (19.63%) failed to be assigned to any known kingdom (Tables S2 and S3).

For the fungal kingdom, the phylum-level assignment of 52 OTUs remained elusive, and the other 764 OTUs spanned five fungal phyla, 14 known classes, 36 orders, 77 families, and 130 genera (Fig. 2, Tables S4 and S5). For the SYS, NYS and BHS, neither OTU richness (F = 0.44, *P* = 0.648) nor the Shannon index (F = 0.637, *P* = 0.537) differed according to a one-way ANOVA (Fig. 3). In addition, no significant correlation was observed between any one of the variables tested for OTU richness (*P* > 0.05).

In Ascomycota, Sordariomycetes (23.78% of all of the OTUs, 28.27% of all of the sequences) was found to be the most abundant class, followed by Dothideomycetes (25.25%, 26.49%) and Eurotiomycetes (19.24%, 23.75%). These abundant classes showed coherent distributions in different sampled sites with relatively higher abundance compared to other classes (Figure S1, Table S4). At the order level, Pleosporales was found to be the most abundant order, accounting for 10.91% and 17.60% of all of the OTUs and sequences, respectively, followed by Chaetothyriales (11.76%, 16.61%), Capnodiales (12.50%, 8.25%), Eurotiales (6.25%, 6.24%) and Xylariales (6.50%, 5.28%) (Fig. 2).

Within Basidiomycota, Agaricomycetes (6.25% of all of the OTUs, 8.48% of all of the sequences) was the most abundant class, followed by Tremellomycetes (7.35%, 6.21%) and Cystobasidiomycetes (3.19%, 2.29%). The least abundant classes were Agaricostilbomycetes (0.12%, 0.014%), Wallemiomycetes (0.25%, 0.041%) and Pucciniomycetes (0.25%, 0.015%) (Table S4). The most abundant order was found to be Polyporales (3.43%, 7.67%), followed by Tremellales (7.11%, 6.17%), Erythrobasidiales (2.94%, 2.11%) and Sporidiobolales (2.57%, 1.59%) (Fig. 2).

The following 16 genera were found to be the most common (>10 OTUs were present in all of the samples) in the sampled regions: *Penicillium* (31 OTUs), *Trichomerium* (30 OTUs), *Nigrospora* (28 OTUs), *Shiraia* (25 OTUs), *Erythrobasidium* (22 OTUs), *Mycosphaerella* (19 OTUs), *Cryptococcus* (18 OTUs), *Rhodotorula* (17 OTUs), *Fusarium* (15 OTUs), *Eutypa* (14 OTUs), *Ganoderma* (14 OTUs), *Perenniporia* (14 OTUs), *Phialemonium* (14 OTUs), *Metschnikowia* (11 OTUs), *Sarocladium* (11 OTUs) and *Phialophora* (11 OTUs) (Table S5). Other genera were frequently isolated from marine sediments, including *Aspergillus* (10 OTUs in 25 samples), *Hypoxylon* (10 OTUs in 30 samples), *Libertella* (10 OTUs in 30 samples) and *Xylaria* (10 OTUs in 30 samples).

### Spatial distribution of community compositions and the effects of variables

Among the 816 OTUs examined, 241 OTUs were only found in one or two regions (Fig. 4a), indicating a notable variability of species compositions across these regions. The plot of non-metric multidimensional scaling (NMDS) shows that the spatial distribution of fungal community compositions varied considerably among the SYS, NYS and BHS (Fig. 5). Our ANOSIM analysis results also show that the fungal community in the SYS differs significantly from that in the BHS (R^2^ = 0.4791, *P* = 0.001) and NYS (R^2^ = 0.2932, *P* = 0.015). However, no significant difference between the NYS and BHS (R^2^ = 0.2291, *P* = 0.055) was found.

Community compositions in the sampled regions were heavily shaped by most of the variables tested according to our db-RDA analysis, with the exception of total organic carbon (TOC) and pH (Fig. 6a). Nine variables explained 39.24% of variances (F = 1.4353, *P* = 0.001), indicating that these variables can partially account for fungal community dynamics across our sampled regions. After variable selection by AIC, TBW and latitude were found to be the main variables associated with dissimilarity changes in community compositions (Fig. 6b), explaining 16.49% of the variance (F = 2.6653, *P* = 0.001). The total variances were significantly explained by TBW (accounting for a 7.43% proportion (F = 2.4026, *P* = 0.001)) and latitude (accounting for a 9.06% proportion (F = 2.9279, *P* = 0.001)). Interestingly, fungal communities in different regions presented various responses to the same variable (Table 1). For example, TBW was found to be significantly correlated with fungal communities in the YS (*P* = 0.02) but not with the entire region or the BHS (*P* > 0.05). Water depth (WD) and salinity showed significant correlations with the entire region (*P* = 0.011, *P* = 0.003) but not with the YS and BHS (*P* > 0.05). The ratio of total organic carbon to nitrogen (C/N) only significantly affected community compositions in the BHS (*P* = 0.026).

## Discussion

Fungal diversity in Chinese seas sediments is poorly understood^19^, which impedes our understanding of the ecological importance of fungi in the marine ecosystem of China. In this study, 816 fungal taxa at the OTU level were revealed, greatly advancing our understanding of fungal diversity in Chinese seas. Moreover, several sequences (more than one fifth of all of the sequences) matched the database poorly and failed to be assigned to any known taxa, indicating the existence of considerable unknown biodiversity. These poorly recognized groups revealed by molecular approaches may prove to be important components of these ecosystems^20^.

Of the 130 fungal genera uncovered in our study, most of them are widespread in terrestrial, freshwater^9^,^19^ and marine habitats^8^,^21^. For example, *Aspergillus, Fusarium, Penicillium, Talaromyces*, and *Trichoderma* were found to be dominant in wetland sediments along the Yangtze River^22^, which discharges large amounts of sediment into the SYS^2^. *Bionectria, Cercophora, Didymella, Gliocladium, Hypocrea, Hypoxylon, Nectria, Nigrospora, Oidiodendron, Ramichloridium* and *Trichoderma*, which have recently been found in Chinese freshwater^19^, and the typical EcM fungal genera *Rhizopogon* and *Sebacina*^23^ were also recovered from our samples. Moreover, several of the genera detected are plant pathogens, and some are reasonably assumed to be dead or dormant in sediments of the sampled seas. Our data suggest that compositions of the fungal community are profoundly affected by the passive dispersal of spores and other propagates from terrestrial environments through terrestrial runoff and riverine inputs.

In Ascomycota, members of Sordariomycetes, Dothideomycetes and Eurotiomycetes found in large proportions in subtropical Chinese seas have also been frequently found in deep-sea sediments in India^24^, the Pacific Ocean^15^ and Arctic fjords^16^, indicating that these classes are ubiquitous in marine sediments. Members of Dothideomycetes, and particularly the order Pleosporales, are believed to have adapted or developed resistance to low temperatures and high levels of osmotic pressure, which may be essential to survival in marine habitats^21^. *Alternaria*^16^, *Aureobasidium*^21^, *Cladosporium*^16^,^21^ and *Phoma*^13^,^24^, which have often been found in deep-sea environments, were consistently observed in our study. In Eurotiomycetes, *Aspergillus* and *Penicillium* are known to be ubiquitous in various marine substrates as well as in marine sponge invertebrates^18^.

Fungal phylotypes of Basidiomycota are the most frequently recovered eukaryotes from deep sediment samples of the Peru Margin, and some species may play important roles in the utilization and recycling of nutrients^12^. Members of Agaricomycetes are dominant in anoxic mangrove sediments of Saint Vincent Bay^25^. In the seas sampled in our study, the most abundant class of Basidiomycota was Agaricomycetes, primarily represented by Polyporales sequences. This finding is unusual, as most species of this order are typical terrestrial wood decomposers and do not live in ocean sediments. Therefore, these Polyporales sequences targeted by sediments are assumed to be dead or possibly dormant spores that fell from the air or that were perhaps flushed into seas via rivers or terrestrial runoff. The yeast genera *Bullera, Cryptococcus, Hannaella, Rhodotorula* and *Sporobolomyces*, which are often found in deep-sea sediments^12^,^13^,^21^,^24^ and European coastal sediments^17^, accounted for 37.63% of all of the Basidiomycota sequences examined in this study. Two yeast genera, *Dioszegia* and *Erythrobasidium*, were uncovered from marine environments for the first time.

Chytridiomycota and Zygomycota detected at low levels in this study were also recovered from deep-sea environments^21^,^26^,^27^ and coastal regions^17^,^28^. Members of the two fungal phyla may be considerably more diverse and numerous in marine habitats than we initially imagined, as the PCR processes and primers used to generate most marine clone libraries are biased toward Dikarya fungi^20^,^29^,^30^. Moreover, Chytridiomycota and groups accounting for mostly unicellular species are difficult to infer for their phylogeny based on ITS regions due to high levels of genetic divergence and the current paucity of reference sequences^13^,^20^,^30^. Interestingly, chytrid-like sequences recovered from freshwater environments are most closely related to fungi that are known to parasitize algae and protists^20^, indicating that the taxa of Chytridiomycota found in this study are likely pathogens of other benthic organisms.

It is well known that various commonly used ITS primers may introduce biases during the amplification of different parts of ITS regions. As fungus-specific primers, ITS1F and ITS2, which facilitate the selective amplification of fungal ITS1 fragments from mycorrhizal and other environmental samples^31^,^32^, show considerable mismatching with Chytridiomycota, Glomeromycota, Microsporidia, Saccharomycetes, several taxa within Dothideomycetes, and Tremellomycetes^29^. Nevertheless, a few studies have showed that the ITS1 region targeted by ITS1F/ITS2 primers can present similar patterns in the fungal community structure as those exhibited by the ITS2 region^33^,^34^. Although several new primers designed for the amplification of ITS1^32^ and ITS2^32^,^35^ would allow for the selective investigation of fungal communities from various environmental samples, no universally accepted approach to covering all fungi has been developed^29^.

Sediment sources of the BHS, NYS and SYS are mainly from Korean and Chinese rivers. Among these rivers, the Yangtze River and Yellow River are the main sources, contributing 4.7–5 × 10^8^ and 10 × 10^8^ tons of sediment per year, respectively^2^. Sediments of the southern part of the YS mainly derive from the Yangtze River, whereas sediments of the southern area of the BHS and the northern part of the YS are mainly derived from the Yellow River. Therefore, it is not surprising that we found clear difference among fungal communities in the BHS and NYS and SYS (Figs 5 and 6). Moreover, the fact that sites located near estuaries of the Yellow (sites 27, 28, 29 and 30) and Yangtze Rivers (sites 1, 2, 4, 5 and 6) were grouped separately into two distinct clusters (Figure S2) clearly highlights the effects of river discharge.

Geographical distance is an important driver of effective fungal community structuring in soils, rhizospheres and wetlands^22^,^36^,^37^, and of bacterial biogeography in marine sediments^6^,^38^. As in these previous studies, the present study found that the beta-diversity of fungal communities in sediments of subtropical Chinese seas was significantly correlated with geographic distance (Table 1), supporting the hypothesis that dispersal limitations of taxa within habitats constitute another critical factor that shapes space variations in species assemblages^39^. However, the SYS, NYS and BHS were not found to differ significantly in terms of OTU richness (*P* > 0.05) and the Shannon index (*P* > 0.05), which was found to differ between marine algae^4^ and animals^1^,^5^ in the sampled seas (biodiversity levels increased from the BHS to the YS).

In addition to spatial factors, the physiochemical compositions of marine habitats were found to heavily affect microbial communities, such as bacteria^6^,^40^,^41^, viruses^42^ and nanociliates^5^. For fungi living in marine environments, pH levels were found to significantly affect the number of colony forming units in certain North Sea locations^43^. Both pH and C/N were shown to affect the mycobiomes of soils sampled in the southern maritime Antarctic^44^. In contrast to these results, in the present study, pH was found to have no significant effect on either fungal richness levels or distribution patterns in our sampled seas, whereas other environmental variables such as WD, TBW, salinity, total nitrogen (TN) and C/N significantly influenced the spatial distribution patterns of fungal communities (Fig. 6a, Table 1). In addition, the results of the db-RDA variable selection tests (Fig. 6b) are consistent with the conclusion that temperature plays a critical role in the determination of fungal biogeography in marine environments^8^,^9^.

Typically, the CWM of the YS emerges in winter and remains within a bottom layer under the thermocline at a depth of 15–30 m in summer^1^. During our sampling period, the effects of CWM on the TBW in the local sea region were obvious and are supported by the fact that the sampled sites located in the central region of the YS showed lower TBW levels than the other sites (Table S1). As a result, these sites (site 8 to site 13, site 15, site 16 and site 18) with TBW levels of less than 10 °C are closely related within the fungal community (Figs 6 and S2). Generally, species richness will decline with a decrease in temperature levels^45^, however, no obvious decline in fungal OTU richness in sites with lower TBW levels was observed (*P* > 0.05). Our network analysis shows that 174 OTUs (21.32% of total OTUs) appeared in one or two temperature categories (Fig. 4b), revealing that a clear variation of species compositions occurred along temperature gradients. These 41 OTUs occurring only within a certain specific temperature category may be taxa with narrow temperature adaptation features. In this case, we hypothesize that CWM may play a role in balancing the competitive relationship between cold- and warm-adapted species, proving critical to the alternation of species composition rather than to the enhancement of species richness. For instance, microbiome experiments have suggested that this increase in temperature may reduce species abundance in some cases through competitive exclusion^46^,^47^.

## Methods

### Sediment sample collection

**Figure 1:**
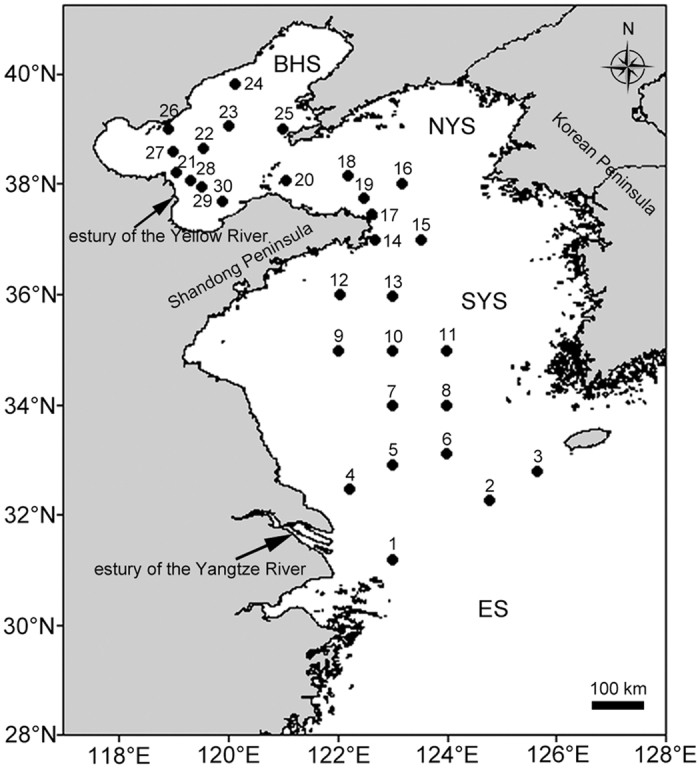
Sampled sites in the SYS (the southern Yellow Sea), NYS (northern Yellow Sea) and BHS (Bohai Sea) of China. ES = the East Sea. The figure was generated using Golden Software Surfer^®^ 13 (http://www.goldensoftware.com/products/surfer) and Adobe Photoshop CC (https://www.adobe.com/products/photoshop.html).

**Figure 2:**
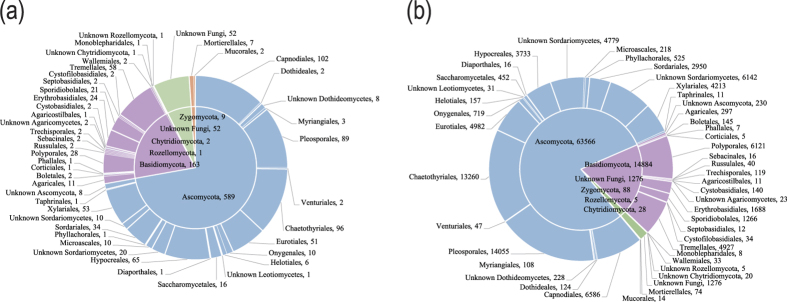
Taxonomic distributions of (**a**) 816 OTUs and (**b**) 79,847 sequences at the fungal phylum and order levels across the entire region sampled.

**Figure 3:**
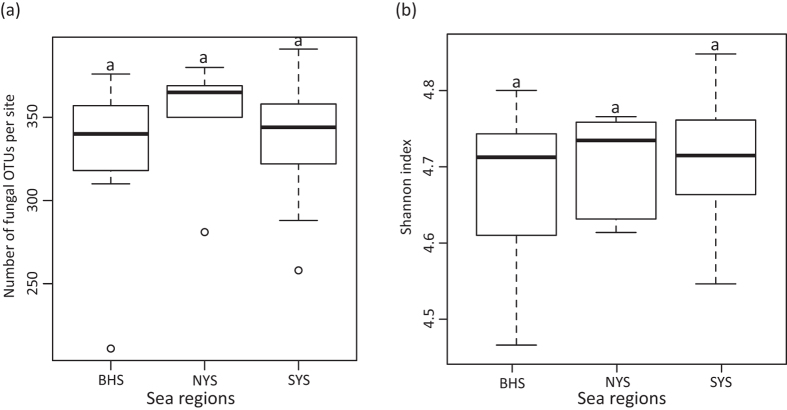
(**a**) OTU richness per site and (**b**) Shannon index in three sea regions as demonstrated by boxplot with median and 95% confidence intervals displayed. Bars without shared letters indicate significant differences at the level of *P* value = 0.05.

**Figure 4:**
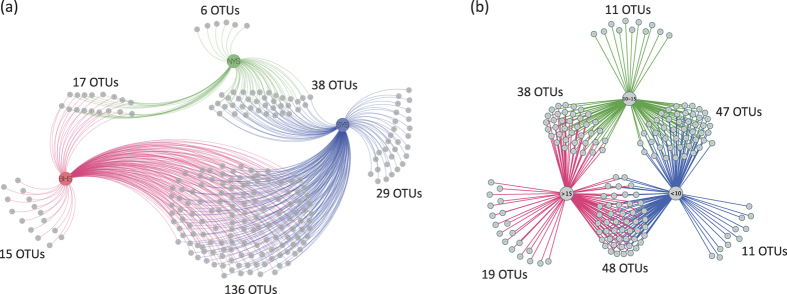
Network diagram of (**a**) 241 OTUs occurring only within one or two regions of the BHS (red color), NYS (green color) and SYS (blue color), and (**b**) 174 OTUs occurring only within one or two temperature categories of <10 °C (blue color), 10–15 °C (green color) and >15 °C (red color).

**Figure 5:**
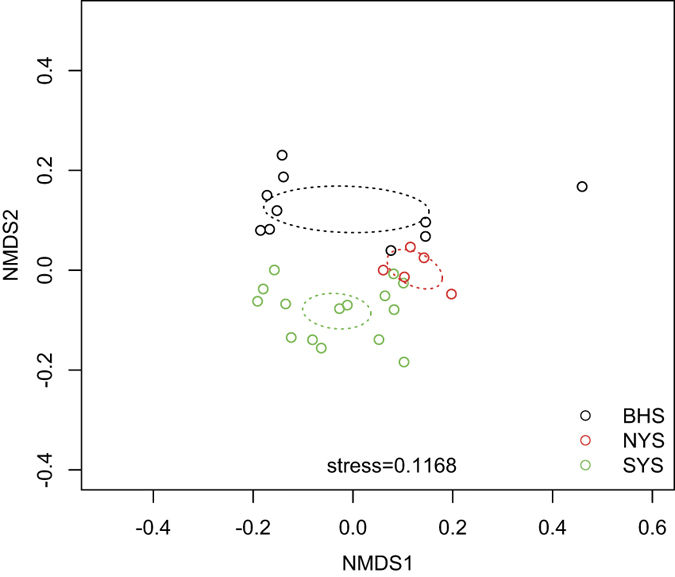
Non-metric multidimensional scaling (NMDS) plot of Bray-Curtis dissimilarity on abundance data transformed by a lg (x + 1) algorithm where x was designed as the number of normalized counts by DESeq2 package. The BHS, NYS and SYS are represented with the black, red and green circles, respectively. Dotted ellipses indicate 95% confidence intervals around centroids of three sea regions.

**Figure 6:**
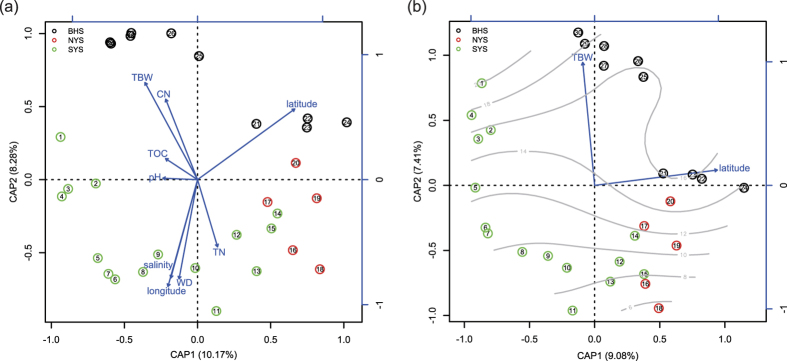
Distance based redundancy analysis (db-RDA) of Bray-Curtis distance on normalized and transformed abundance data. Three sampled regions are indicated with the black (the BHS), red (the NYS) and green circles (the SYS), respectively. Rectangles represent spatial or environmental factors. (**a**) The db-RDA ordination without variable selection tests. (**b**) The db-RDA ordination after variable selection tests. Gray lines indicate surface fitting of TBW.

The YS, which includes the SYS and NYS, has a surface area of 380 × 10^9^ m^2^ and an average depth of 44 m with a maximum depth of 100 m in the center. The BHS covers a 77 × 10^9^ m^2^ sea area and has an 18 m average water depth with a 70 m maximum depth in the northern section of the Bohai Strait. Thirty sediment samples (Fig. 1, Table S1) were collected from the SYS, NYS and BHS using a 0.05 m^3^ stainless steel grab sampler (Wildco^®^, Florida, USA) in June 2013 aboard the *Dongfanghong* 2# research vessel. The collected sediment was undisturbed and compact. Ten sub-cores of sediment were collected using 3.5 cm-inner-diameter pre-cleaned glass tubes sealed with sterile plastic film from each sampler. These glass tubes filled with sediment were immediately stored at −80 °C until total DNA extraction.

### Environmental and spatial variables

The spatial and environmental variables included sediment longitude, latitude and physiochemical properties (e.g., TOC, TN, C/N, pH, WD, TBW and salinity of bottom water) (Table S1). Salinity, TBW and WD were measured using a Seabird 911 conductivity-temperature-depth (CTD) instrument. TOC and TN in sediments were determined using an Elemental Analyzer (EA3000, Euro Vector SpA, Milan, Italy) in our laboratory. The pH was measured by adding 10 ml of distilled water to 4 g of sediment and recording pH levels using a pH electrode (STARTER 300, OHAUS, Beijing, China). Geographic distances between the sites (with kilometer units) (Table S6) were calculated using the distm function of the geosphere package in R^48^ based on the longitude and latitude of the sites.

### DNA extraction and 454-pyrosequencing

In the laboratory, aliquots of sediment (2–8 cm in depth) from the central parts of the sub-cores of each sampler were removed using a sterile spatula and mixed thoroughly. Genomic DNA extraction was performed on 0.5 g of composite sediment using the FastDNA^®^ Spin kit for soil (MP Biomedicals, Solon, OH, USA) according to the manufacturer’s instructions. The remaining parts of the pooled samples were stored for physicochemical analysis.

The DNA samples were amplified using fungal-specific primer pairs ITS1F (5′-(*A*) CTTGGTCATTTAGAGGAAGTAA-3′) and ITS2 (5′-(*B*) GCTGCGTTCTTCATCGATGC-3′) to generate PCR ITS1 rRNA fragments of approximately 400 bp, where *A* and *B* represent the two fusion primers (GCCTCCCTCGCGCCATCAG and GCCTTGCCAGCCCGCTCAG)^49^. The conditions for PCR included an initial hot start incubation (5 min at 94 °C) followed by 34 cycles of denaturation at 94 °C for 30 s, annealing at 55 °C for 30 s and extension at 72 °C for 1 min, followed by a final extension at 72 °C for 15 min (GS Titanium LV emPCR Purification Kit, TaKaRa DR500A and D4030A). The PCR products were pyrosequenced using a Genome Sequencer FLX 454 System (454 Life Sciences/Roche Applied Biosystems, Nutley, NJ, USA). The 454 SFF files and corresponding barcode files were deposited in the National Center for Biotechnology Information Sequence Read Archives (SRA) as BioProject ID SRP067807.

### Bioinformatics analysis

Raw sequences were assigned to samples based on the barcodes and were trimmed (qaverage = 30, qwindowsize = 50, minlength = 200, maxambig = 1, maxhomop = 8, bdiffs = 1) with the trim.seqs command in Mothur 1.31.2^50^. Then, ITSx 1.0.11^51^ was applied to trim out 18S and 5.8S rRNA genes and remove the compromised and nontarget sequences with a minlength setting for ITS1 of 50 bases and other parameters based on default settings (Tedersoo, personal communication). The Uchime_ref command in USEARCH^52^ was used to detect and remove chimeras using the unified system for the DNA-based fungal species (UNITE) and international nucleotide sequence databases (INSDC) fungal ITS databases (the version released on March 11, 2015) as reference^53^,^54^. These filtered sequences were clustered into operational taxonomic units (OTUs) at a 97% sequence similarity threshold using CD-Hit (www.cd-hit.org). OTUs represented by fewer than 5 sequences were removed, as these OTUs tend to be artifactual^55^.

The longest sequence of each OTU was selected as the representative for local BLASTn 2.2.30+ searches^56^ with a conservative setting (word_size = 7, penalty = −3, reward = 1) against UNITE + INSDC database. Rules employed for the taxonomic placement of OTUs^37^ were adopted here. The BLASTn e-value < e^−50^ was used as cutoff to assign a sequence to the fungal kingdom. However, query sequences with an e-value between e^−20^ and e^−50^ were manually checked against the 100 best-matching sequences for accurate assignment. These above queries were assigned to the fungal kingdom and were labeled by genus, family, order, or class at 90, 85, 80, and 75% sequence identity, respectively. The e-value > e^−20^ was used as threshold to classify sequences as “unknown”.

### Networks

To display putative major OTUs associated with sea regions and temperatures, two networks were produced to visualize shared OTUs among three sea regions (the SYS, NYS and BHS) and three temperature categories (<10 °C, 10 °C –15 °C and >15 °C) using Cytoscape 3.0.1^57^.

### Statistical analyses

OTU richness (S), Shannon’s diversity (H) and evenness (H/lnS) values with sequence counts were calculated. A one-way ANOVA followed by a pairwise t-test was used to explore variations in the richness of OTUs and Shannon’s diversity levels across the sea regions (the SYS, NYS and BHS).

NMDS was used to compare dissimilarities among the fungal communities across three sea regions based on Bray-Curtis dissimilarity^58^. Prior to our NMDS analyses, sequence counts were normalized using the DESeq2^59^ package in R^48^ and were then transformed using a lg (x + 1) algorithm^60^ where x was designated as the number of normalized counts. The anosim function in the vegan package of R was applied to evaluate changes in beta-diversity levels based on Bray-Curtis dissimilarity and permutations = 999.

A distance-based redundancy analysis (db-RDA) with Bray-Curtis distances was performed to visualize the relationship between compositional variations and environmental or spatial variables^60^. To further identify important variables associated with community structures, the step function in R with Akaike’s information criterion (AIC) was used to build an alternative db-RDA model. The significance of the models, axes and terms were tested using ANOVA function in the vegan package. All samples were clustered using the Euclidean distance of scores for the first two axes of the two above listed db-RDA models. In addition, correlations between Bray-Curtis dissimilarities of the fungal community and spatial or environmental variables in different sea regions were checked through a Mantel test^22^.

## Additional Information

**How to cite this article**: Li, W. *et al*. Fungal communities in sediments of subtropical Chinese seas as estimated by DNA metabarcoding. *Sci. Rep.* **6**, 26528; doi: 10.1038/srep26528 (2016).

## Supplementary Material

Supplementary Information

Supplementary Information

## Figures and Tables

**Table 1:** Correlations between Bray-Curtis distances of fungal community and spatial or environmental variables checked through a Mantel test.

		Spatial variables			Environmental variables						
		GD	Longitude	Latitude	TBW	WD	TOC	TN	C/N	pH	Salinity
Entire region	r	0.2959	0.2431	0.3220	0.0976	0.1535	−0.0032	0.0232	0.0647	−0.0591	0.2642
	*P*	0.001*	0.007*	0.004*	0.133	0.011*	0.464	0.313	0.221	0.666	0.003*
YS	r	0.6630	0.2285	0.6956	0.3355	0.0094	0.1847	0.0891	0.1228	−0.0200	0.1656
	*P*	0.001*	0.039*	0.001*	0.002*	0.415	0.053	0.246	0.095	0.550	0.064
BHS	r	0.3665	0.0185	0.5371	0.1091	−0.1256	−0.0746	0.2464	0.2943	−0.0550	−0.0454
	*P*	0.028*	0.299	0.016*	0.232	0.672	0.627	0.121	0.026*	0.552	0.601

GD = geographical distance (km), TBW = temperature of bottom water (°C), WD = water depth (m), TOC = total organic carbon (mg/g), TN = total nitrogen (mg/g), C/N = the ratio of total organic carbon to nitrogen. The asterisk indicates significance at the level of *P* value = 0.05.

## References

[b1] Liu J. Y. Status of marine biodiversity of the China seas. PLoS ONE 8, e50719 (2013). 23320065 10.1371/journal.pone.0050719 PMC3540058

[b2] Yang S. Y., Jung H. S., Lim D. I. & Li C. X. A review on the provenance discrimination of sediments in the Yellow Sea. Earth-Sci. Rev. 63, 93–120 (2003).

[b3] He Q. . Economic development and coastal ecosystem change in China. Sci. Rep. 4, 5995 (2014). 25104138 10.1038/srep05995 PMC4125988

[b4] Tseng C. K. Seaweeds in the Yellow Sea and the Bohai Sea of China. (Science Press, Beijing, 2009).

[b5] Dong J. . SSU rDNA sequence diversity and seasonally differentiated distribution of nanoplanktonic ciliates in neritic Bohai and Yellow Seas as revealed by T-RFLP. PLoS ONE 9, e102640 (2014). 25025156 10.1371/journal.pone.0102640 PMC4099327

[b6] Liu J. . Bacterial and archaeal communities in sediments of the north Chinese marginal seas. Microb. Ecol. 70, 105–117 (2015). 25501892 10.1007/s00248-014-0553-8

[b7] Hyde K. D. . Role of fungi in marine ecosystems. Biodivers. Conserv. 7, 1147–1161 (1998).

[b8] Jones E. B. G. Marine fungi: some factors influencing biodiversity. Fungal Divers. 4, 53–73 (2000).

[b9] Shearer C. A. . Fungal biodiversity in aquatic habitats. Biodivers. Conserv. 16, 49–67 (2007).

[b10] Manohar C. S. & Raghukumar C. Fungal diversity from various marine habitats deduced through culture-independent studies. FEMS Microbiol. Lett. 341, 69–78 (2013). 23363246 10.1111/1574-6968.12087

[b11] Alexander E. . Microbial eukaryotes in the hypersaline anoxic L’Atalante deep-sea basin. Environ. Microbiol. 11, 360–381 (2009). 18826436 10.1111/j.1462-2920.2008.01777.x

[b12] Edgcomb V. P., Beaudoin D., Gast R., Biddle J. F. & Teske A. Marine subsurface eukaryotes: the fungal majority. Environ. Microbiol. 13, 172–183 (2011). 21199255 10.1111/j.1462-2920.2010.02318.x

[b13] Lai X. . Fungal communities from methane hydrate-bearing deep-sea marine sediments in South China Sea. ISME J. 1, 756–762 (2007). 18059498 10.1038/ismej.2007.51

[b14] Thaler A. D., Dover C. L. V. & Vilgalys R. Ascomycete phylotypes recovered from a Gulf of Mexico methane seep are identical to an uncultured deep-sea fungal clade from the Pacific. Fungal Ecol. 5, 270–273 (2012).

[b15] Xu W., Pang K. L. & Luo Z. H. High fungal diversity and abundance recovered in the deep-sea sediments of the Pacific Ocean. Microb. Ecol. 68, 688–698 (2014). 25004994 10.1007/s00248-014-0448-8

[b16] Zhang T., Wang N. F., Zhang Y. Q., Liu H. Y. & Yu L. Y. Diversity and distribution of fungal communities in the marine sediments of Kongsfjorden, Svalbard (High Arctic). Sci. Rep. 5, 14524 (2015). 26494429 10.1038/srep14524 PMC4615975

[b17] Richards T. A. . Molecular diversity and distribution of marine fungi across 130 European environmental samples. Proc. R. Soc. B 282, 20152243 (2015). 10.1098/rspb.2015.2243 PMC4685826 26582030

[b18] Li Q. & Wang G. Diversity of fungal isolates from three Hawaiian marine sponges. Microbiol. Res. 164, 233–241 (2009). 17681460 10.1016/j.micres.2007.07.002

[b19] Hu D. M., Liu F. & Cai L. Biodiversity of aquatic fungi in China. Mycology 4, 125–168 (2013).

[b20] Richards T. A., Jones M. D., Leonard G. & Bass D. Marine fungi: their ecology and molecular diversity. Ann. Rev. Mar. Sci. 4, 495–522 (2012). 10.1146/annurev-marine-120710-100802 22457985

[b21] Nagano Y. & Nagahama T. Fungal diversity in deep-sea extreme environments. Fungal Ecol. 5, 463–471 (2012). 10.1007/978-3-642-23342-5_9 22222832

[b22] Wu B. . The biogeography of fungal communities in wetland sediments along the Changjiang River and other sites in China. ISME J. 7, 1299–1309 (2013). 23446835 10.1038/ismej.2013.29 PMC3695295

[b23] Tedersoo L. & Smith M. E. Lineages of ectomycorrhizal fungi revisited: foraging strategies and novel lineages revealed by sequences from belowground. Fungal Biol. Rev. 27, 83–99 (2013).

[b24] Singh P., Raghukumar C., Verma P. & Shouche Y. Phylogenetic diversity of culturable fungi from the deep-sea sediments of the Central Indian Basin and their growth characteristics. Fungal Divers. 40, 89–102 (2010).

[b25] Arfi Y., Marchand C., Wartel M. & Record E. Fungal diversity in anoxic-sulfidic sediments in a mangrove soil. Fungal Ecol. 5, 282–285 (2012).

[b26] Nagano Y. . Fungal diversity in deep-sea sediments-the presence of novel fungal groups. Fungal Ecol. 3, 316–325 (2010).

[b27] Nagahama T., Takahashi E., Nagano Y., Abdel-Wahab M. A. & Miyazaki M. Molecular evidence that deep-branching fungi are major fungal components in deep-sea methane cold-seep sediments. Environ. Microbiol. 13, 2359–2370 (2011). 21605311 10.1111/j.1462-2920.2011.02507.x

[b28] Cury J. C. . Microbial diversity of a Brazilian coastal region influenced by an upwelling system and anthropogenic activity. PLoS ONE 6, e16553 (2011). 21304582 10.1371/journal.pone.0016553 PMC3029357

[b29] Tedersoo L. . Shotgun metagenomes and multiple primer pair-barcode combinations of amplicons reveal biases in metabarcoding analyses of fungi. MycoKeys 10, 1–43 (2015).

[b30] Ishii N., Ishida S. & Kagami M. PCR primers for assessing community structure of aquatic fungi including Chytridiomycota and Cryptomycota. Fungal Ecol. 13, 33–43 (2015).

[b31] Tedersoo L. . 454 Pyrosequencing and Sanger sequencing of tropical mycorrhizal fungi provide similar results but reveal substantial methodological biases. New Phytol. 188, 291–301 (2010). 20636324 10.1111/j.1469-8137.2010.03373.x

[b32] Toju H., Tanabe A. S., Yamamoto S. & Sato H. High-coverage ITS primers for the DNA-based identification of ascomycetes and basidiomycetes in environmental samples. PLoS ONE 7, e40863 (2012). 22808280 10.1371/journal.pone.0040863 PMC3395698

[b33] Mello A. . ITS-1 versus ITS-2 pyrosequencing: a comparison of fungal populations in truffle grounds. Mycologia 103, 1184–1193 (2011). 21700633 10.3852/11-027

[b34] Bazzicalupo A. L., Bálint M. & Schmitt I. Comparison of ITS1 and ITS2 rDNA in 454 sequencing of hyperdiverse fungal communities. Fungal Ecol. 6, 102–109 (2013).

[b35] Ihrmark K. . New primers to amplify the fungal ITS2 region-evaluation by 454-sequencing of artificial and natural communities. FEMS Microbiol. Ecol. 82, 666–677 (2012). 22738186 10.1111/j.1574-6941.2012.01437.x

[b36] Bahram M., Peay K. G. & Tedersoo L. Local-scale biogeography and spatiotemporal variability in communities of mycorrhizal fungi. New Phytol. 205, 1454–1463 (2015). 25767850 10.1111/nph.13206

[b37] Tedersoo L. . Global diversity and geography of soil fungi. Science 346, 1256688 (2014). 25430773 10.1126/science.1256688

[b38] Schauer R., Bienhold C., Ramette A. & Harder J. Bacterial diversity and biogeography in deep-sea surface sediments of the South Atlantic Ocean. ISME J. 4, 159–170 (2010). 19829317 10.1038/ismej.2009.106

[b39] Green J. L. . Spatial scaling of microbial eukaryote diversity. Nature 432, 747–750 (2004). 15592411 10.1038/nature03034

[b40] Herlemann D. P. R., Woelk J., Labrenz M. & Jürgens K. Diversity and abundance of “Pelagibacterales” (SAR11) in the Baltic Sea salinity gradient. Syst. Appl. Microbiol. 37, 601–604 (2014). 25444644 10.1016/j.syapm.2014.09.002

[b41] Vigneron A. . Phylogenetic and functional diversity of microbial communities associated with subsurface sediments of the Sonora Margin, Guaymas Basin. PLoS ONE 9, e104427 (2014). 25099369 10.1371/journal.pone.0104427 PMC4123917

[b42] Hurwitz B. L., Westveld A. H., Brum J. R. & Sullivan M. B. Modeling ecological drivers in marine viral communities using comparative metagenomics and network analyses. PNAS. 111, 10714–10719 (2014). 25002514 10.1073/pnas.1319778111 PMC4115555

[b43] Krause E., Wichels A., Gimenez L. & Gerdts G. Marine fungi may benefit from ocean acidification. Aquat. Microb. Ecol. 69, 59–67 (2013).

[b44] Dennis P. G. . Soil fungal community composition does not alter along a latitudinal gradient through the maritime and sub-Antarctic. Fungal Ecol. 5, 403–408 (2012).

[b45] Kerr J. T. & Packer L. Habitat heterogeneity as a determinant of mammal species richness in high-energy regions. Nature 385, 252–254 (1997).

[b46] Chapin F. S. III, Shaver G. R., Giblin A. E., Nadelhoffer K. J. & Laundre J. A. Responses of arctic tundra to experimental and observed changes in climate. Ecology 76, 694–711 (1995).

[b47] Petchey O. L., McPhearson P. T., Casey T. M. & Morin P. J. Environmental warming alters food-web structure and ecosystem function. Nature 402, 69–72 (1999).

[b48] Team, R. D. C. R*: A language and environment for statistical computing.* R foundation for statistical computing, Vienna, Austria. (2015) Available at: http://www. R-project. Org/.

[b49] Buée M. . 454 Pyrosequencing analyses of forest soils reveal an unexpectedly high fungal diversity. New Phytol. 184, 449–456 (2009). 19703112 10.1111/j.1469-8137.2009.03003.x

[b50] Schloss P. D. . Introducing mothur: open-source, platform-independent, community-supported software for describing and comparing microbial communities. Appl. Environ. Microbiol. 75, 7537–7541 (2009). 19801464 10.1128/AEM.01541-09 PMC2786419

[b51] Bengtsson-Palme J. . Improved software detection and extraction of ITS1 and ITS2 from ribosomal ITS sequences of fungi and other eukaryotes for analysis of environmental sequencing data. Methods Ecol. Evol. 4, 914–919 (2013).

[b52] Edgar R. C. UPARSE: highly accurate OTU sequences from microbial amplicon reads. Nat. Methods 10, 996–998 (2013). 23955772 10.1038/nmeth.2604

[b53] Kõljalg U. . Towards a unified paradigm for sequence-based identification of fungi. Mol. Ecol. 22, 5271–5277 (2013). 24112409 10.1111/mec.12481

[b54] Nilsson R. H. . A comprehensive, automatically updated fungal ITS sequence dataset for reference-based chimera control in environmental sequencing efforts. Microbes Environ. 30, 145–150 (2015). 25786896 10.1264/jsme2.ME14121 PMC4462924

[b55] Lindahl B. D. . Fungal community analysis by high-throughput sequencing of amplified markers–a user’s guide. New Phytol. 199, 288–299 (2013). 23534863 10.1111/nph.12243 PMC3712477

[b56] Altschul S. F. . Gapped BLAST and PSI-BLAST: a new generation of protein database search programs. Nucleic Acids Res. 25, 3389–3402 (1997). 9254694 10.1093/nar/25.17.3389 PMC146917

[b57] Cline M. S. . Integration of biological networks and gene expression data using Cytoscape. Nat. Protoc. 2, 2366–2382 (2007). 17947979 10.1038/nprot.2007.324 PMC3685583

[b58] Ramette A. Multivariate analyses in microbial ecology. FEMS Microbiol. Ecol. 62, 142–160 (2007). 17892477 10.1111/j.1574-6941.2007.00375.x PMC2121141

[b59] Senés-Guerrero C. & Schüβler A. A conserved arbuscular mycorrhizal fungal core-species community colonizes potato roots in the Andes. Fungal Divers. doi: 10.1007/s13225-015-0328-7 (2015).

[b60] Legendre P. & Gallagher E. D. Ecologically meaningful transformations for ordination of species data. Oecologia 129, 271–280 (2001). 10.1007/s004420100716 28547606

