# Supplementary material for: Fungal communities in sediments of subtropical Chinese seas as estimated by DNA metabarcoding
Source: Sci Rep. 2016 May 20;6:26528. doi: 10.1038/srep26528 (PMC4873734; doi:10.1038/srep26528)
Supplement: Supplementary Information [file srep26528-s1.pdf]

Fungal communities in sediments of subtropical Chinese seas as estimated by DNA metabarcoding

Wei Li[1]*, Meng Meng Wang[1], Xi Guang Wang[2], Xiao Li Cheng[1, 3], Jia Jia Guo[1], Xiao Meng Bian[1] & Lei Cai[3]*

[1] College of Marine Life Sciences, Ocean University of China, Qingdao 266003, China

[2] Institute of Agricultural Information, Chinese Academy of Agricultural Sciences, Beijing 100081, China

[3] State Key Laboratory of Mycology, Institute of Microbiology, University of Chinese Academy of Sciences, Beijing 100101, China

*Corresponding authors: liwei01@ouc.edu.cn; cail@im.ac.cn

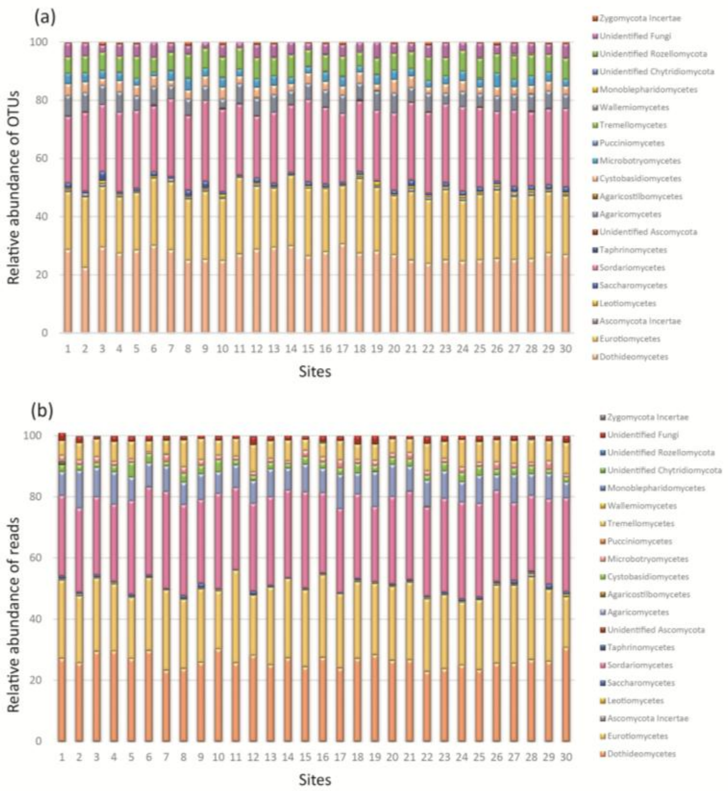

Supplementary Figure S1. Relative abundances of (a) OTU and (b) read numbers at class level in different sampled sites.

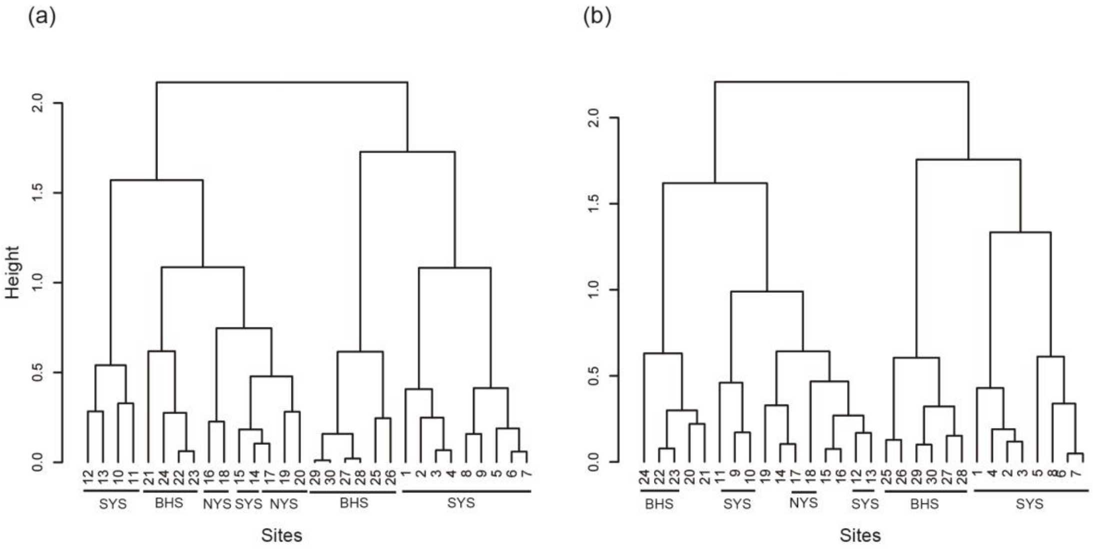

Supplementary Figure S2 Hierarchic clustering of sampled sites using the Euclidean distance of scores for the first two axes of the db-RDA model without variable selection tests (a) and after variable selection tests (b).

Supplementary Table S1. Information of sampled sites including spatial and environmental factors, OTU richness, Shannon index, and evenness values.

**Site**	**Region**	**Longitude (°)**	**Latitude (°)**	**WD (m)**	**TBW (℃)**	**Salinity(‰)**	**TOC (mg/g)**	**TN (mg/g)**	**C/N**	**pH**	**Richness of OTU**	**Shannon index**	**Pielou' evenness**
1	SYS	123	31.2	63	19.8743	34.019	0.92	0.06	15.1	8.36	391	4.847397	0.8121351
2	SYS	124.78	32.27	47.8	14.6316	31.945	1.13	0.08	14.04	8.55	293	4.730683	0.8328414
3	SYS	125.67	32.8	90	12.5611	33.293	1.85	0.08	23.85	8.26	288	4.6663	0.8240037
4	SYS	122.22	32.47	25	20.4331	30.381	1.36	0.07	20	8.02	332	4.745514	0.8174683
5	SYS	123	32.93	32	14.4658	31.323	0.97	0.07	14.79	8.15	344	4.847999	0.8300456
6	SYS	124	33.13	60	10.8012	32.679	1.33	0.09	14.13	8.16	346	4.701174	0.8041089
7	SYS	123	34	67.2	9.9863	32.955	1.23	0.12	10.59	8.37	312	4.706288	0.819482
8	SYS	124	34	78.9	8.8943	32.82	1.13	0.1	11.19	8.43	352	4.778653	0.8149648
9	SYS	122	35	50.5	8.1514	32.105	0.79	0.08	9.83	8.3	368	4.858158	0.8222902
10	SYS	123	35	73	9.3679	32.981	1.29	0.14	9.38	8.27	342	4.718191	0.808628
11	SYS	124	35	80.8	8.8383	32.735	0.83	0.09	9.03	8.17	258	4.5413	0.8178161
12	SYS	122	36	43.5	7.4973	31.458	1.13	0.1	11.59	8.23	373	4.755529	0.8030846
13	SYS	123	36	70	8.6907	32.742	1.39	0.18	7.93	8.15	336	4.625713	0.7951908
14	SYS	122.67	37	34	14.3032	31.085	1.04	0.06	17.14	8.36	364	4.662401	0.7906189
15	SYS	123.52	37	73	7.8796	32.168	1.02	0.06	18.49	8.4	348	4.561595	0.7794663
16	NYS	123.18	38.05	64.3	6.384	32.081	0.8	0.07	11.13	8.14	369	4.764123	0.8060036
17	NYS	122.69	37.48	48.5	13.2141	31.158	0.79	0.03	26.61	8.19	350	4.77172	0.8145739
18	NYS	122.18	38.19	52.8	4.3177	31.642	1.73	0.2	8.54	8.21	281	4.612119	0.8179902
19	NYS	122.47	37.77	34	9.8419	31.177	1.06	0.05	19.21	8.22	380	4.738954	0.7977807
20	NYS	121.04	38.09	24.3	13.6521	30.887	0.94	0.05	18.91	8.22	365	4.630726	0.7848824
21	BHS	119.08	38.22	17	17.583	29.598	1.97	0.07	27.97	8.29	352	4.609745	0.7861588
22	BHS	119.54	38.66	27.5	15.222	30.736	1.25	0.11	11.41	8.17	310	4.608213	0.8033043
23	BHS	120	39.09	22.7	18.4987	30.596	0.79	0.05	15.34	8.16	319	4.564712	0.7917712

24	BHS	120.12	39.85	21	13.5339	29.515	1.03	0.09	11.22	8.19	211	4.456939	0.8327834
25	BHS	121	39.03	35	14.5109	30.76	0.74	0.05	15.65	8.14	343	4.707228	0.8063455
26	BHS	118.87	39.02	21.3	17.1929	30.405	0.74	0.05	14.11	8.14	318	4.748065	0.8240234
27	BHS	118.97	38.62	24.5	11.8816	30.433	1.67	0.08	20.86	8.37	337	4.759442	0.8177619
28	BHS	119.28	38.09	17.5	18.4314	29.079	1.66	0.06	29.3	8.3	362	4.729922	0.8028186
29	BHS	119.48	37.96	18.3	18.2201	29.308	1.62	0.06	25.15	8.28	357	4.807908	0.8179864
30	BHS	119.88	37.7	16.4	20.9746	29.281	1.25	0.05	24.77	8.26	376	4.724247	0.7967242

Supplementary Table S2. Read counts of OTUs in different sampled sites.

**OTUs**	**1**	**2**	**3**	**4**	**5**	**6**	**7**	**8**	**9**	**10**	**11**	**12**	**13**	**14**	**15**	**16**	**17**	**18**	**19**	**20**	**21**	**22**	**23**	**24**	**25**	**26**	**27**	**28**	**29**	**30**	**sum**
OTU1	300	140	96	157	120	150	66	109	86	134	75	191	109	143	91	150	107	113	165	180	138	122	133	44	162	103	109	179	126	267	4065
OTU2	13	130	117	156	130	192	150	222	217	136	137	129	188	270	259	244	184	124	185	0	3	0	1	0	0	0	0	0	1	0	3188
OTU3	13	10	9	11	4	1	13	11	42	193	101	331	221	267	233	159	207	113	172	259	204	164	218	87	10	17	9	16	10	28	3133
OTU4	7	7	0	5	79	169	159	144	85	125	83	115	129	203	221	128	154	74	167	171	205	106	123	47	5	5	7	7	4	11	2745
OTU5	167	144	115	196	111	144	123	157	153	168	75	84	27	29	34	30	31	14	39	27	14	32	29	4	153	72	166	123	129	111	2701
OTU6	95	0	1	0	0	0	0	1	2	0	1	1	2	46	63	58	65	43	78	74	72	59	41	14	195	187	224	255	199	151	1927
OTU7	12	11	8	9	12	9	15	10	15	10	4	87	116	127	162	121	171	66	232	228	151	80	140	34	10	4	12	7	13	5	1881
OTU8	34	26	18	22	23	25	22	34	38	20	14	23	29	41	33	30	38	12	46	291	297	193	160	52	29	17	22	30	23	28	1670
OTU9	183	92	83	76	81	99	110	139	79	1	0	0	0	0	0	3	0	0	0	2	0	0	1	0	0	8	89	150	114	284	1594
OTU10	44	68	35	51	37	44	33	99	82	44	40	25	33	81	77	81	45	35	41	79	53	26	33	13	27	48	41	50	42	32	1439
OTU11	63	20	42	69	51	44	38	74	54	49	42	27	56	62	45	51	53	15	80	61	21	29	19	12	37	45	62	58	33	36	1348
OTU12	46	9	5	6	4	18	59	53	62	81	39	64	67	64	58	80	57	22	57	79	71	41	48	12	28	33	25	38	34	83	1343
OTU13	33	33	19	45	19	17	24	109	102	4	27	39	34	65	24	20	40	14	31	31	37	42	18	17	0	0	12	35	61	83	1035
OTU14	45	36	30	38	21	34	21	37	48	36	27	35	40	35	41	57	23	13	34	45	53	18	22	15	23	25	45	29	25	62	1013
OTU15	51	12	22	16	7	47	12	43	29	41	26	36	19	42	57	25	19	15	55	29	29	41	19	12	28	39	38	40	45	43	937

OTU16	100	67	55	58	60	5	1	0	1	0	0	0	0	1	9	1	4	1	5	5	11	0	1	0	105	75	84	68	65	115	897
OTU17	51	32	26	29	17	46	44	62	16	7	0	2	3	0	0	2	8	0	0	0	5	0	6	2	157	123	42	32	36	74	822
OTU18	9	1	3	4	1	2	0	9	43	57	39	50	27	48	62	33	46	23	58	78	91	45	42	23	2	3	7	2	2	4	814
OTU19	208	11	13	25	21	44	22	33	44	13	30	19	22	46	40	46	36	17	18	20	22	15	12	12	6	0	5	2	0	2	804
OTU20	66	37	59	71	26	65	33	44	27	22	10	11	9	27	22	21	23	13	30	14	29	12	5	5	0	0	0	0	0	66	747
OTU21	145	44	29	47	37	44	8	10	11	9	5	11	10	4	7	7	6	1	9	11	7	4	5	1	46	44	48	55	48	29	742
OTU22	9	10	5	4	8	6	12	13	23	36	13	64	42	56	40	49	29	16	55	51	39	30	37	10	9	10	0	17	7	27	727
OTU23	13	14	10	11	2	6	10	9	13	8	9	7	16	42	44	64	61	20	42	51	34	32	52	9	19	14	29	13	12	14	680
OTU24	91	22	21	25	21	16	19	30	22	15	11	17	24	22	23	15	12	9	10	12	13	5	6	1	38	29	43	43	25	31	671
OTU25	51	23	26	52	28	57	22	50	43	30	26	38	26	39	17	12	8	0	0	14	8	7	17	5	1	0	0	0	29	37	666
OTU26	40	21	18	32	32	35	21	41	13	28	4	31	9	28	16	18	8	10	22	22	16	8	10	2	9	8	3	29	23	64	621
OTU27	22	12	10	15	39	13	15	28	9	16	12	17	16	16	30	14	27	20	32	24	22	22	26	15	39	18	19	13	11	26	598
OTU28	44	18	9	10	16	5	28	40	33	24	8	15	18	6	13	23	26	18	25	20	23	34	16	1	12	11	23	7	21	22	569
OTU29	28	11	14	13	7	2	5	20	8	36	15	17	8	18	3	15	41	19	13	23	20	33	18	1	37	36	44	9	11	43	568
OTU30	25	13	13	21	2	9	8	10	24	27	8	51	4	20	16	9	23	12	63	8	15	21	10	6	11	12	52	35	19	15	562
OTU31	16	13	22	11	22	8	12	22	23	19	14	15	9	13	12	33	43	16	24	8	10	10	29	0	9	35	16	20	26	15	525
OTU32	2	2	0	6	26	18	20	27	30	41	9	24	25	35	28	36	16	18	32	29	22	27	23	17	0	2	0	0	1	2	518
OTU33	19	8	6	30	27	3	4	14	38	8	3	48	6	8	9	16	23	8	0	5	11	27	23	21	16	15	13	31	6	48	494
OTU34	19	10	12	19	23	34	9	36	32	16	15	9	9	39	7	17	11	18	9	37	17	3	21	4	15	8	7	22	11	1	490
OTU35	24	10	6	21	11	5	17	31	39	9	5	9	9	19	11	27	29	12	21	29	21	19	18	2	17	14	16	3	13	10	477
OTU36	19	7	7	19	6	11	8	6	20	17	4	20	5	17	15	10	18	5	17	34	39	14	16	5	10	11	13	16	17	40	446
OTU37	50	34	23	30	20	4	5	5	8	33	2	3	9	7	6	4	1	4	2	4	8	1	1	0	38	17	16	28	21	56	440
OTU38	0	6	37	29	22	26	35	63	37	48	33	68	13	0	0	1	1	1	1	0	0	0	0	0	0	0	0	0	0	0	421
OTU39	8	5	10	15	11	5	4	14	17	13	8	11	10	24	17	16	10	6	36	40	25	15	16	4	11	11	13	17	14	9	415
OTU40	2	5	9	12	21	3	11	23	33	21	16	10	7	13	27	17	11	6	30	17	33	23	5	6	5	5	1	2	6	1	381
OTU41	13	33	2	100	35	47	9	1	0	119	4	0	4	0	3	1	0	0	0	0	0	0	0	1	0	0	7	0	1	0	380

OTU42	8	18	9	15	7	4	9	13	6	17	14	22	11	20	2	3	10	7	46	5	10	14	5	5	19	7	17	22	13	9	367
OTU43	24	8	11	25	3	13	5	10	22	21	6	14	6	8	12	5	16	6	11	9	12	9	3	3	9	19	26	9	16	12	353
OTU44	10	4	14	3	7	14	8	18	16	11	3	22	11	7	6	10	19	6	6	4	13	6	13	1	31	32	17	14	16	6	348
OTU45	18	4	8	16	19	15	8	20	31	16	14	12	6	10	8	24	20	3	13	9	10	5	4	2	1	9	2	9	10	21	347
OTU46	20	5	8	6	16	13	8	12	12	7	20	10	8	20	11	12	17	11	29	22	13	6	9	1	6	7	4	12	10	4	339
OTU47	15	4	8	16	7	12	8	17	5	6	2	21	14	25	4	7	13	16	12	16	8	9	12	3	16	16	12	10	0	24	338
OTU48	14	8	8	31	9	8	14	25	6	17	12	15	10	14	7	7	19	1	14	23	13	6	6	3	3	4	15	7	6	5	330
OTU49	2	2	2	2	4	2	1	3	2	1	2	3	15	32	27	33	31	17	25	27	34	17	26	6	2	0	1	3	3	5	330
OTU50	2	1	1	28	29	31	23	37	30	23	37	24	19	16	0	1	2	1	4	5	3	2	2	1	2	1	3	0	0	1	329
OTU51	0	0	0	0	0	0	0	0	0	4	0	0	0	5	1	2	2	1	1	1	3	1	3	0	90	35	63	37	50	28	327
OTU52	5	10	4	12	3	9	7	11	12	8	7	5	6	13	5	12	10	3	70	24	8	8	11	3	12	6	3	10	3	8	308
OTU53	20	13	22	13	7	11	16	5	25	11	5	19	7	16	17	5	4	1	6	4	2	2	5	3	6	9	9	16	6	19	304
OTU54	6	15	11	7	10	7	4	11	5	7	11	14	15	18	5	7	10	15	21	15	26	6	12	1	6	13	3	11	4	7	303
OTU55	14	18	12	1	12	18	2	14	3	23	21	6	4	17	10	18	16	8	18	29	3	5	7	3	1	0	1	7	4	5	300
OTU56	22	17	4	14	14	11	9	11	16	13	3	17	7	15	2	12	9	8	1	32	7	7	12	2	10	2	3	3	4	8	295
OTU57	51	17	22	1	2	0	3	1	0	0	1	0	1	0	1	0	1	1	0	1	2	0	0	0	31	23	54	28	18	19	278
OTU58	4	7	1	9	2	1	4	2	18	18	15	19	35	19	6	12	11	10	11	14	6	2	5	4	8	8	5	11	2	3	272
OTU59	18	9	8	0	12	16	7	10	10	5	8	7	7	2	1	5	15	4	3	13	13	3	3	0	7	26	9	27	5	9	262
OTU60	0	6	3	1	1	2	2	8	0	19	4	24	8	5	25	16	23	17	22	9	25	13	7	4	3	2	3	2	5	0	259
OTU61	3	10	0	7	4	2	19	17	12	14	10	10	10	0	14	15	32	2	15	18	0	2	2	3	15	7	0	5	5	0	253
OTU62	13	12	3	9	1	2	1	3	9	5	10	11	6	2	7	17	8	3	6	14	10	6	6	0	17	14	18	18	13	7	251
OTU63	18	8	9	5	11	12	13	11	9	13	8	12	3	12	7	10	7	7	3	6	9	6	9	3	6	4	4	2	6	12	245
OTU64	0	4	1	20	5	2	4	19	17	40	2	5	5	7	12	12	9	1	18	10	5	9	0	1	8	2	0	11	5	8	242
OTU65	9	12	11	7	8	9	11	5	28	8	2	7	3	7	6	4	7	4	14	17	8	8	5	1	0	2	6	14	7	11	241
OTU66	6	2	2	4	3	5	23	0	12	14	5	12	8	7	8	23	10	1	11	15	4	3	19	0	7	5	6	13	4	9	241
OTU67	15	9	3	10	2	4	5	0	9	17	7	13	9	5	7	6	4	2	13	11	11	7	5	1	17	8	9	6	8	16	239

OTU68	35	10	7	50	0	0	0	0	0	1	0	0	0	0	0	0	0	0	0	0	1	0	1	3	17	32	20	16	14	29	236
OTU69	15	5	1	4	17	0	13	22	3	4	3	9	13	14	19	19	4	1	1	4	2	5	5	0	6	15	4	2	9	12	231
OTU70	16	7	9	13	6	17	25	27	2	0	0	0	0	0	1	0	0	0	0	1	2	0	0	0	18	23	7	18	15	21	228
OTU71	0	1	5	1	1	0	0	0	0	0	2	1	0	2	1	0	17	5	33	50	29	43	24	7	0	1	0	2	1	2	228
OTU72	43	2	14	3	5	8	3	7	17	8	1	8	3	8	11	11	13	8	9	3	13	2	3	0	0	1	4	1	8	11	228
OTU73	7	22	8	20	15	29	12	33	27	3	0	1	0	0	1	0	0	0	0	0	0	0	1	0	0	0	1	0	19	26	225
OTU74	2	5	3	4	1	8	2	4	4	1	3	9	3	3	5	5	11	1	11	28	19	23	26	4	4	4	5	7	10	9	224
OTU75	10	2	2	8	13	11	0	14	12	30	4	3	4	4	8	6	6	8	13	6	13	2	9	1	13	2	2	5	4	2	217
OTU76	6	8	2	10	3	5	14	19	2	10	10	2	13	12	4	3	3	9	11	16	1	4	4	1	5	7	8	6	11	6	215
OTU77	7	18	1	14	10	3	0	1	5	0	1	17	3	5	9	2	7	0	25	4	5	4	0	2	29	8	17	7	2	6	212
OTU78	7	8	3	4	0	9	10	6	9	0	0	20	20	8	20	11	2	1	8	1	4	13	2	1	0	2	14	5	13	9	210
OTU79	2	1	1	0	10	8	0	10	1	10	1	18	2	12	3	1	7	0	6	20	5	11	4	3	12	13	8	26	7	5	207
OTU80	12	9	1	0	0	0	0	0	7	0	5	11	1	12	3	8	9	5	4	3	6	7	6	2	14	10	21	22	19	10	207
OTU81	0	1	0	0	26	39	9	14	33	36	0	2	1	5	4	0	3	1	9	11	0	4	3	1	0	0	0	0	0	0	202
OTU82	4	6	9	1	11	8	1	8	5	6	1	5	5	14	3	9	8	10	13	10	14	4	5	4	5	2	2	11	12	4	200
OTU83	2	2	7	11	2	13	8	6	1	3	6	2	5	4	12	13	14	2	13	21	6	8	5	4	4	5	5	5	9	1	199
OTU84	7	10	14	4	6	5	2	11	4	13	2	6	2	11	11	5	15	5	11	11	7	5	1	4	3	5	11	3	3	2	199
OTU85	16	9	0	6	2	10	5	5	3	14	7	15	7	11	6	0	6	3	6	8	5	5	12	1	3	3	10	8	2	9	197
OTU86	4	5	4	4	2	6	3	5	6	1	1	3	4	4	7	1	2	1	2	34	43	9	22	4	4	2	0	7	3	4	197
OTU87	0	0	0	11	17	15	14	20	48	14	16	18	0	0	0	0	5	1	0	0	4	0	0	0	0	0	0	0	0	0	183
OTU88	15	1	1	21	10	6	7	14	8	2	8	3	2	10	0	9	11	0	16	0	2	4	5	0	1	4	4	8	7	3	182
OTU89	10	7	3	1	5	1	3	4	5	10	12	5	2	4	4	2	9	3	7	8	7	7	5	1	2	2	12	6	10	14	171
OTU90	0	0	0	0	0	0	0	0	0	0	0	0	20	40	28	23	53	0	0	0	0	0	0	0	0	0	0	0	0	0	164
OTU91	15	6	4	11	1	1	2	5	1	6	2	4	4	5	8	7	9	5	5	5	8	1	3	0	3	4	8	8	9	6	156
OTU92	0	0	0	1	0	0	3	11	6	3	8	17	3	6	12	21	2	2	29	9	10	5	6	0	0	1	0	0	0	0	155
OTU93	25	1	2	3	4	8	6	10	7	13	3	10	1	3	2	8	4	1	1	2	3	0	0	1	11	7	1	4	4	7	152

OTU94	35	22	18	19	0	0	4	0	0	0	0	1	0	0	0	0	0	0	0	0	0	1	0	0	0	0	0	18	22	10	150
OTU95	5	6	5	6	2	7	11	8	3	1	6	3	7	2	5	5	6	4	7	4	7	3	5	0	3	4	7	13	2	2	149
OTU96	11	3	0	3	7	6	4	5	6	2	3	4	6	8	12	6	7	4	4	3	4	3	3	3	7	6	6	3	6	2	147
OTU97	6	2	0	1	6	8	7	5	8	2	5	6	5	6	7	6	9	5	4	7	8	3	4	1	2	5	1	10	4	4	147
OTU98	0	0	1	0	1	4	0	1	2	0	0	1	15	8	25	18	26	6	9	6	9	4	5	0	0	1	0	2	1	2	147
OTU99	1	4	2	1	2	0	9	11	2	2	4	3	3	6	3	3	9	0	11	5	23	12	5	1	1	2	9	2	8	2	146
OTU100	12	7	5	13	6	4	7	11	8	1	0	0	2	10	3	0	2	0	1	6	1	1	5	0	10	4	11	2	4	9	145
OTU101	6	6	1	0	5	7	13	1	6	4	1	11	2	4	1	5	4	5	0	13	1	2	5	0	12	13	14	2	1	0	145
OTU102	0	0	12	1	0	9	0	6	8	4	6	4	7	18	2	2	3	1	22	1	4	4	1	0	9	3	6	1	0	10	144
OTU103	11	4	5	7	4	2	7	13	8	5	1	4	4	2	8	4	3	0	1	2	4	2	3	0	5	10	6	1	12	3	141
OTU104	31	0	4	3	3	2	2	3	4	19	1	4	2	3	1	2	4	1	2	8	2	1	1	1	0	0	4	9	7	16	140
OTU105	0	0	0	0	0	16	13	18	19	25	13	31	1	1	0	1	0	0	0	0	1	0	0	0	0	0	0	0	0	0	139
OTU106	2	5	5	1	3	3	3	1	1	2	2	4	4	7	2	4	2	3	8	8	13	6	2	2	4	10	12	1	3	13	136
OTU107	17	0	3	8	0	11	3	3	5	0	3	4	5	2	7	2	4	0	8	4	6	1	3	1	3	3	10	4	4	9	133
OTU108	1	0	0	3	1	2	0	0	1	11	4	6	6	10	17	18	7	6	4	12	9	2	11	0	0	1	0	0	0	0	132
OTU109	0	0	2	9	9	2	0	8	12	0	3	6	5	8	1	2	3	7	0	5	6	0	2	5	4	2	11	8	4	8	132
OTU110	14	11	3	5	8	4	4	5	7	3	2	3	2	0	0	0	0	7	3	0	2	5	8	1	4	6	9	12	0	0	128
OTU111	11	13	1	2	0	0	2	11	0	0	1	2	2	2	12	10	13	0	0	13	13	4	1	0	4	2	2	1	2	2	126
OTU112	8	1	5	3	5	2	2	2	7	6	1	15	8	6	17	17	7	2	11	0	0	0	0	0	0	0	0	0	0	0	125
OTU113	8	12	4	4	2	3	2	2	8	2	1	5	2	4	5	3	10	3	7	5	3	1	0	0	7	1	7	10	2	2	125
OTU114	16	5	5	7	8	1	6	0	20	6	0	4	0	1	3	0	2	2	3	3	5	2	3	0	3	5	6	3	0	5	124
OTU115	5	3	2	0	3	5	1	5	11	5	4	1	6	16	3	1	3	1	5	8	5	5	5	1	5	5	2	3	2	2	123
OTU116	3	0	1	0	1	3	1	17	22	0	1	2	7	0	0	0	2	1	4	2	2	2	0	2	5	7	0	0	21	15	121
OTU117	12	0	5	5	1	12	2	7	0	3	0	5	3	1	6	3	4	5	1	7	7	1	3	1	0	4	4	0	1	16	119
OTU118	3	6	6	7	2	3	0	7	0	7	3	1	2	7	7	9	2	3	2	9	3	5	8	0	4	0	0	6	6	1	119
OTU119	27	0	7	1	5	7	0	3	4	5	3	5	1	3	3	3	1	0	4	6	5	5	1	0	2	2	3	4	4	4	118

OTU120	10	6	3	0	1	1	0	2	7	5	0	1	2	1	2	3	3	3	7	2	4	2	5	1	4	5	5	6	15	9	115
OTU121	13	3	6	5	4	4	4	6	5	5	0	1	6	3	2	3	7	1	2	6	5	1	1	1	3	3	3	5	4	3	115
OTU122	12	6	2	5	11	2	7	3	6	0	0	10	2	0	9	1	0	2	0	10	5	3	3	0	5	3	0	5	0	3	115
OTU123	4	3	5	4	5	3	2	3	6	0	3	1	1	7	3	5	6	2	3	10	2	2	7	3	6	2	9	3	2	2	114
OTU124	13	1	3	1	3	4	8	5	7	3	2	6	3	4	5	4	3	1	4	6	9	0	5	1	2	3	3	2	2	1	114
OTU125	2	0	3	1	4	0	2	26	12	0	4	7	5	3	3	0	11	0	4	4	1	0	9	0	0	2	5	0	2	2	112
OTU126	1	4	5	0	1	4	1	3	6	1	4	2	1	2	10	1	7	6	8	2	5	12	5	1	0	6	0	0	12	1	111
OTU127	1	3	0	4	0	6	1	3	4	3	5	10	1	2	2	4	6	3	7	3	2	4	3	2	1	11	7	3	0	8	109
OTU128	5	0	0	11	6	7	1	5	1	0	0	3	1	14	4	0	6	6	0	5	5	0	1	0	9	2	13	0	2	1	108
OTU129	4	2	3	6	1	10	8	4	5	3	6	3	0	2	11	4	1	1	1	7	3	5	1	0	1	2	4	2	4	2	106
OTU130	4	2	9	2	2	0	16	6	3	1	2	2	4	2	2	4	4	1	4	3	0	3	5	1	1	10	2	4	0	5	104
OTU131	10	5	1	3	2	1	7	3	2	4	2	3	3	4	2	2	4	2	4	2	4	2	5	0	0	3	10	10	1	3	104
OTU132	1	3	2	0	0	1	1	4	3	5	1	5	1	1	4	0	2	2	1	0	1	2	0	0	9	12	9	6	9	19	104
OTU133	5	2	2	4	1	5	0	8	0	4	0	13	9	1	5	6	2	1	5	1	1	1	1	3	4	1	6	2	1	9	103
OTU134	12	0	2	4	4	6	0	2	5	4	0	8	1	10	2	0	2	0	0	1	3	2	7	2	0	0	4	13	1	6	101
OTU135	4	2	2	0	3	2	3	3	6	3	0	0	6	9	7	1	4	2	4	4	6	2	1	0	7	2	14	0	0	1	98
OTU136	1	0	0	0	0	0	0	0	0	0	0	3	0	9	5	12	4	1	18	16	16	5	7	0	0	0	0	0	0	0	97
OTU137	10	4	1	10	1	5	0	0	0	8	0	0	2	2	1	0	0	0	3	1	5	0	4	0	6	7	4	11	3	8	96
OTU138	7	5	2	1	0	0	0	1	5	4	2	7	2	4	9	1	4	0	3	1	5	5	1	0	2	4	1	6	6	7	95
OTU139	13	20	7	22	4	5	1	0	0	0	0	0	0	0	0	0	0	0	0	0	0	0	0	0	0	0	13	1	2	6	94
OTU140	4	0	1	3	0	1	3	9	6	1	0	1	5	4	1	3	7	1	0	5	6	8	1	1	0	2	8	1	6	2	90
OTU141	0	0	0	0	0	5	3	0	1	0	3	2	0	0	1	14	11	2	12	6	8	4	14	0	0	0	0	1	1	2	90
OTU142	2	7	0	9	8	2	0	3	1	0	0	0	0	8	0	4	11	2	0	0	0	0	0	0	1	5	7	6	3	9	88
OTU143	0	1	0	0	2	0	0	15	8	11	0	17	1	3	1	9	2	0	0	2	0	8	6	0	0	0	2	0	0	0	88
OTU144	12	0	0	5	1	10	1	8	0	2	8	1	0	2	7	5	2	1	1	3	0	0	2	0	0	4	0	3	7	2	87
OTU145	17	0	3	4	2	7	4	1	1	0	0	8	0	1	2	0	5	0	2	2	4	2	0	0	1	0	1	2	5	13	87

OTU146	13	0	3	1	1	1	1	2	6	4	3	2	3	4	3	1	3	4	4	5	2	0	2	0	6	0	1	0	5	6	86
OTU147	0	3	1	5	2	6	7	15	2	2	2	5	1	8	8	7	8	3	0	0	0	0	0	0	0	0	0	0	1	0	86
OTU148	7	3	4	3	5	1	7	5	8	4	2	0	2	1	0	5	2	2	0	2	1	2	2	0	3	3	5	3	1	3	86
OTU149	6	3	2	3	5	7	4	1	0	2	1	2	2	7	7	6	1	3	2	1	2	2	0	1	2	5	3	1	4	0	85
OTU150	1	5	7	4	9	4	2	6	2	2	1	4	0	1	1	2	0	1	2	11	5	2	3	0	3	0	3	3	1	0	85
OTU151	9	2	1	2	1	4	3	5	4	4	1	0	4	3	3	5	3	3	1	2	2	1	6	0	1	1	4	3	2	2	82
OTU152	3	0	4	6	1	3	1	5	0	4	0	1	4	3	5	3	7	1	6	6	3	5	2	1	2	0	2	0	3	1	82
OTU153	5	0	4	3	1	2	1	3	1	3	0	6	2	6	4	1	0	1	2	3	8	3	6	1	6	0	3	2	2	3	82
OTU154	2	0	0	1	1	1	2	4	1	2	2	0	2	0	0	2	4	4	1	4	2	1	2	1	27	8	3	4	0	0	81
OTU155	4	0	0	5	6	3	3	2	1	2	2	6	4	6	1	7	1	1	9	1	2	1	5	3	2	1	0	0	1	1	80
OTU156	0	0	0	2	1	4	2	0	0	6	0	3	2	0	7	11	11	4	9	0	3	2	0	0	0	0	8	2	0	0	77
OTU157	0	0	0	3	4	4	4	3	0	2	1	6	5	3	1	9	3	0	4	6	0	2	0	0	5	5	0	3	2	2	77
OTU158	4	0	1	1	0	0	2	13	2	4	0	0	10	0	6	10	7	2	0	5	2	2	0	0	0	0	4	0	0	1	76
OTU159	2	4	3	3	3	0	5	2	6	0	1	2	0	0	12	5	3	0	0	1	9	0	0	4	3	5	0	1	2	0	76
OTU160	2	0	3	2	6	0	4	1	1	3	0	5	3	4	0	2	4	2	4	1	3	2	2	0	1	2	4	6	4	3	74
OTU161	0	0	1	4	0	2	0	4	2	2	0	0	1	0	6	3	0	3	6	17	4	9	0	0	0	0	0	0	0	10	74
OTU162	0	0	0	0	2	4	1	3	3	0	3	5	6	5	6	2	0	4	4	7	2	0	2	2	0	2	0	4	2	4	73
OTU163	16	0	6	1	9	0	2	0	0	10	2	0	0	0	0	0	0	0	4	0	0	0	0	0	0	5	9	2	6	0	72
OTU164	0	0	0	0	0	0	0	6	1	0	10	0	5	0	0	9	0	3	0	5	18	0	0	0	15	0	0	0	0	0	72
OTU165	3	3	2	1	0	4	2	3	7	0	2	1	1	4	4	1	3	1	11	3	5	1	4	0	0	0	0	0	0	6	72
OTU166	0	5	0	11	0	0	0	4	0	0	1	0	1	6	0	2	1	0	11	7	1	4	0	3	7	2	0	2	4	0	72
OTU167	7	5	0	8	0	0	6	0	0	4	1	0	0	0	0	0	2	0	0	0	0	2	0	0	6	0	2	3	10	15	71
OTU168	3	4	0	6	1	7	3	6	3	3	2	4	1	2	1	3	2	0	3	6	2	1	1	0	0	1	0	1	2	3	71
OTU169	1	0	1	1	1	2	0	6	6	14	0	3	4	1	0	4	4	0	0	3	0	0	1	2	0	0	2	3	8	4	71
OTU170	7	1	1	2	3	4	3	3	1	3	2	0	4	3	0	3	2	0	3	1	5	1	0	0	2	2	3	0	4	7	70
OTU171	1	1	1	1	3	1	2	4	5	2	2	0	1	7	3	12	4	0	1	3	3	0	2	0	3	2	3	1	1	1	70

OTU172	4	1	3	0	1	3	6	0	2	2	6	2	0	3	6	5	2	1	4	7	1	1	0	0	3	1	0	2	2	2	70
OTU173	1	2	0	7	2	3	7	3	1	6	1	5	0	4	2	0	8	0	2	3	3	0	1	0	1	3	3	0	0	1	69
OTU174	3	0	0	0	0	5	0	1	0	2	0	22	7	5	0	0	4	8	1	0	0	0	0	0	4	0	0	0	0	7	69
OTU175	2	1	0	4	0	1	3	4	11	2	0	0	2	3	7	1	4	1	1	1	0	0	3	2	4	4	2	2	1	2	68
OTU176	2	9	0	5	1	0	8	0	1	0	2	2	1	1	1	2	1	0	4	2	1	5	0	0	4	1	1	2	4	8	68
OTU177	0	0	0	0	0	0	0	0	0	0	0	1	0	0	0	0	0	0	4	26	5	13	12	5	0	0	0	1	0	0	67
OTU178	2	4	0	6	4	0	0	6	5	1	0	0	2	3	0	1	2	1	1	5	4	0	2	0	1	0	4	1	1	11	67
OTU179	3	2	0	0	4	1	0	8	0	1	0	3	0	1	0	9	3	0	0	0	2	3	7	0	0	2	2	3	3	10	67
OTU180	4	2	0	2	0	0	2	0	2	6	2	8	0	1	1	3	2	4	1	2	1	0	2	0	3	5	2	0	2	9	66
OTU181	4	1	5	3	5	0	0	0	0	0	0	3	1	6	0	7	0	5	6	9	5	1	0	0	0	1	0	2	2	0	66
OTU182	1	6	0	4	2	0	0	1	0	3	0	0	2	5	2	7	4	2	3	4	0	5	0	0	5	1	2	0	3	3	65
OTU183	3	3	1	3	3	3	1	1	9	2	5	0	6	1	3	0	3	4	4	0	3	2	0	0	0	1	0	0	2	2	65
OTU184	6	1	1	2	2	1	0	0	2	2	0	0	0	6	6	1	0	2	1	3	4	3	6	6	3	0	4	1	1	1	65
OTU185	2	0	1	3	1	2	3	2	2	0	1	5	2	1	6	4	0	3	2	4	2	3	2	0	3	2	3	2	3	1	65
OTU186	5	4	2	7	5	2	2	1	0	0	0	0	5	0	1	1	5	4	7	0	1	1	5	0	0	1	0	1	2	2	64
OTU187	0	0	0	0	0	4	0	1	3	7	0	12	0	1	0	7	4	0	0	1	1	3	0	2	10	2	1	2	3	0	64
OTU188	4	0	3	3	0	0	1	1	0	2	0	0	2	8	0	0	7	0	5	5	15	0	0	0	0	0	7	0	0	0	63
OTU189	1	0	1	2	1	0	6	1	0	2	0	0	0	0	1	0	0	1	1	2	0	1	1	2	16	4	12	5	2	1	63
OTU190	0	4	0	4	0	9	0	0	2	10	2	0	0	10	1	7	3	0	3	0	3	0	0	0	2	0	2	0	0	0	62
OTU191	4	1	0	11	0	0	0	4	0	0	0	3	2	0	6	0	1	3	10	0	1	0	4	3	0	0	0	1	5	3	62
OTU192	4	2	1	3	1	2	1	0	3	1	0	0	2	2	1	4	10	0	5	2	4	3	1	1	1	1	1	2	3	1	62
OTU193	13	0	7	0	11	3	0	1	0	0	2	1	1	0	0	0	17	1	0	0	0	1	0	0	0	0	0	0	0	4	62
OTU194	6	0	2	2	1	4	1	0	2	0	0	5	2	1	5	1	0	0	1	9	0	0	0	1	2	3	3	2	3	6	62
OTU195	1	3	3	2	2	7	2	0	0	0	0	2	0	1	0	2	1	4	3	0	4	1	1	3	4	0	0	8	0	7	61
OTU196	9	3	0	9	0	9	0	0	0	1	1	2	1	1	2	1	3	2	1	0	2	4	1	0	1	2	3	0	3	0	61
OTU197	3	1	1	0	1	2	0	1	1	2	1	0	4	0	0	3	1	2	0	1	0	2	1	0	20	5	9	0	0	0	61

OTU198	12	0	0	1	3	2	0	2	3	0	1	3	2	4	3	1	4	0	0	1	4	1	1	2	2	2	4	2	0	1	61
OTU199	2	2	1	1	5	7	0	3	7	2	3	0	0	0	2	0	2	2	6	1	2	0	0	2	7	0	0	1	1	2	61
OTU200	1	0	0	0	1	7	0	13	0	6	1	6	10	4	0	0	2	0	2	3	1	0	0	0	0	0	0	0	1	2	60
OTU201	3	1	0	1	1	2	4	2	3	2	2	1	7	3	3	3	0	6	1	0	6	0	2	0	2	2	3	0	0	0	60
OTU202	3	0	0	0	8	0	0	4	4	2	0	0	4	0	0	1	2	1	0	0	2	0	0	0	7	1	8	13	0	0	60
OTU203	0	0	0	5	1	0	1	6	0	5	3	5	5	1	0	4	0	3	0	0	4	0	0	0	0	2	1	6	2	6	60
OTU204	2	0	0	0	0	4	1	0	0	2	2	4	4	2	1	7	0	0	3	7	7	2	3	1	3	0	0	4	0	1	60
OTU205	1	6	0	1	1	1	1	4	3	2	0	0	5	3	2	5	4	4	1	0	3	0	2	0	3	3	1	0	2	1	59
OTU206	4	2	1	1	0	4	2	5	2	1	1	1	1	4	0	1	3	5	0	0	4	1	4	0	2	1	2	1	5	1	59
OTU207	0	2	0	2	0	1	7	5	7	1	0	0	1	0	0	0	2	0	3	0	0	0	1	0	2	0	5	0	0	20	59
OTU208	1	0	0	0	0	0	0	2	1	2	0	3	9	6	0	2	1	3	0	3	4	2	2	6	1	1	0	2	2	4	57
OTU209	1	0	1	0	1	0	1	1	1	0	1	2	1	1	4	1	2	0	1	2	1	1	0	0	11	17	3	0	2	1	57
OTU210	0	6	0	5	0	0	0	6	0	2	0	0	3	0	13	0	6	0	0	3	0	0	1	2	2	0	0	3	4	0	56
OTU211	8	0	1	0	0	3	0	0	4	0	0	0	2	2	0	0	6	0	0	1	5	0	2	0	2	0	12	5	0	2	55
OTU212	0	3	0	2	0	0	1	8	5	7	0	2	1	3	0	3	0	1	2	5	0	0	0	0	2	3	3	1	0	3	55
OTU213	0	1	0	0	0	0	0	1	1	1	0	8	1	0	6	0	2	0	0	0	2	3	3	0	4	10	8	0	0	4	55
OTU214	6	6	2	1	0	1	0	2	1	3	11	1	0	0	1	2	0	0	1	1	2	2	0	0	3	1	1	0	3	4	55
OTU215	4	2	1	3	3	1	1	4	3	1	3	1	1	3	1	3	0	2	1	2	3	1	0	0	3	1	2	2	2	0	54
OTU216	0	4	2	0	0	0	0	0	3	5	1	0	1	10	3	5	0	0	1	0	7	0	0	0	1	0	1	4	6	0	54
OTU217	1	2	0	2	4	3	1	0	1	5	0	3	2	2	0	0	5	0	2	2	3	1	2	0	1	2	1	1	2	6	54
OTU218	1	4	1	1	2	1	0	2	3	0	1	2	3	3	4	2	4	0	3	2	3	1	2	0	3	0	1	2	1	1	53
OTU219	0	1	0	0	3	2	3	0	1	3	0	2	2	0	1	2	1	2	0	8	1	3	4	0	1	0	11	1	0	1	53
OTU220	1	0	6	4	3	1	1	2	0	0	2	2	1	2	3	0	0	0	5	4	3	1	2	0	1	1	2	2	1	3	53
OTU221	8	6	0	0	1	2	0	0	0	0	0	15	1	0	0	5	5	0	0	0	0	1	0	0	4	0	0	2	0	2	52
OTU222	0	0	0	0	6	0	2	0	2	6	2	3	0	5	6	0	3	0	0	0	0	5	1	0	0	3	3	4	1	0	52
OTU223	0	1	0	0	0	0	0	1	1	1	0	0	0	0	0	3	2	0	10	11	4	5	1	5	2	0	1	2	1	1	52

OTU224	9	2	10	4	0	2	0	1	1	1	0	0	0	0	0	0	0	0	0	1	0	0	0	0	0	4	3	11	2	0	51
OTU225	0	1	0	0	0	5	2	2	5	1	3	4	4	3	3	1	3	0	7	1	4	1	0	0	0	0	1	0	0	0	51
OTU226	7	1	0	1	4	2	0	4	0	0	0	4	0	0	0	0	2	0	0	8	0	0	0	0	2	2	12	2	0	0	51
OTU227	13	0	1	2	2	0	2	0	1	1	1	3	1	1	2	0	3	1	3	1	2	2	1	0	0	2	2	1	0	3	51
OTU228	0	2	0	0	1	4	0	6	0	1	13	0	0	6	0	3	0	1	1	2	0	0	4	0	1	0	0	0	0	5	50
OTU229	2	3	2	1	4	0	3	2	0	0	0	1	3	1	2	1	3	0	1	1	0	0	2	1	1	0	2	4	3	7	50
OTU230	1	0	3	3	1	0	1	2	0	1	0	1	1	3	0	2	3	1	2	5	6	2	4	0	1	2	1	1	0	2	49
OTU231	0	0	0	4	5	0	5	3	2	0	0	0	0	0	4	6	1	1	0	6	5	3	0	0	2	0	0	2	0	0	49
OTU232	0	1	0	3	1	1	0	0	4	4	0	1	3	3	1	0	3	1	1	3	1	5	0	3	0	1	0	5	0	3	48
OTU233	2	2	1	0	1	1	0	2	1	1	0	2	3	4	0	1	0	1	3	4	6	5	0	0	1	1	0	3	2	1	48
OTU234	1	0	1	1	1	3	3	0	3	3	1	0	1	0	2	4	3	0	1	1	0	0	0	0	0	2	4	6	3	4	48
OTU235	2	1	5	0	0	0	0	1	3	0	1	9	2	2	0	3	3	0	0	1	1	2	1	5	0	0	1	2	0	3	48
OTU236	1	2	1	1	0	1	1	0	2	0	0	1	0	0	1	3	3	0	2	1	3	2	1	0	3	1	2	9	2	4	47
OTU237	2	1	2	1	3	1	1	5	2	2	0	4	1	0	1	3	1	0	1	6	1	1	2	0	1	0	1	1	3	0	47
OTU238	21	0	0	0	0	4	0	0	0	0	0	0	0	7	0	4	0	0	0	4	0	0	0	0	0	6	1	0	0	0	47
OTU239	0	0	0	0	0	0	0	1	3	0	0	0	0	10	0	0	7	1	0	0	0	0	6	0	0	5	9	0	5	0	47
OTU240	0	0	0	1	0	0	0	0	1	1	0	1	0	0	0	0	1	0	0	0	0	0	1	0	21	9	7	0	2	1	46
OTU241	5	1	0	2	0	2	3	2	1	1	4	0	0	0	1	3	1	0	2	2	1	0	2	0	3	0	1	4	4	1	46
OTU242	7	0	1	2	1	2	1	4	1	2	1	0	2	0	0	0	3	0	0	0	0	0	0	0	6	2	3	2	6	0	46
OTU243	2	0	1	5	0	1	4	0	3	1	2	0	0	4	2	0	1	1	1	0	4	0	0	0	4	2	2	2	0	3	45
OTU244	3	0	0	1	0	1	0	2	2	0	0	1	8	3	3	1	0	2	0	3	2	0	3	1	1	0	1	4	1	2	45
OTU245	0	13	1	2	2	4	0	0	0	0	0	0	0	8	0	0	0	0	0	3	0	0	2	2	7	0	0	0	0	0	44
OTU246	2	4	1	2	1	0	1	0	0	2	1	2	0	0	3	0	3	1	5	4	2	2	1	0	2	0	2	2	1	0	44
OTU247	1	1	1	0	0	0	2	0	3	0	0	2	1	4	2	6	1	0	5	0	1	2	2	0	2	1	3	2	1	1	44
OTU248	0	0	3	5	4	0	6	5	0	0	1	2	3	3	0	0	0	1	0	0	0	0	0	0	0	5	0	3	2	1	44
OTU249	0	0	0	1	0	2	0	1	8	8	1	0	0	2	3	0	0	0	3	4	0	1	0	1	3	0	2	0	4	0	44

OTU250	0	0	1	0	1	0	0	0	0	1	0	0	2	0	1	5	3	3	8	5	3	0	0	0	1	6	0	2	1	1	44
OTU251	0	7	0	0	0	0	7	10	0	0	0	0	0	6	0	2	0	0	5	2	0	2	0	0	0	0	3	0	0	0	44
OTU252	0	0	2	0	0	11	0	0	3	11	0	0	0	0	0	0	1	0	0	0	2	4	0	2	0	0	1	0	6	0	43
OTU253	2	0	0	2	0	0	0	0	3	0	1	5	0	1	6	5	0	0	1	10	1	0	2	0	1	0	0	0	1	2	43
OTU254	1	0	0	1	2	2	3	1	0	0	0	2	0	2	2	1	6	2	0	1	0	1	0	0	0	1	0	3	7	5	43
OTU255	1	3	2	9	0	3	0	2	0	0	0	0	0	4	0	3	0	0	5	4	1	2	1	0	1	0	1	1	0	0	43
OTU256	8	0	0	2	1	1	2	0	3	1	0	1	1	1	2	1	2	0	0	0	5	0	0	1	0	0	0	6	1	4	43
OTU257	0	0	2	4	1	5	0	1	1	0	0	1	2	2	3	4	4	0	0	1	2	0	0	1	0	0	1	1	0	7	43
OTU258	2	0	1	1	1	3	0	5	0	1	0	0	4	6	2	0	0	2	4	0	3	2	1	0	0	0	3	0	1	0	42
OTU259	0	1	3	1	1	0	1	1	3	1	0	2	2	2	2	1	2	4	2	4	0	0	0	0	0	2	2	3	1	1	42
OTU260	5	0	0	0	1	0	4	3	1	10	1	1	1	0	3	0	2	0	1	2	0	1	1	1	0	0	1	2	0	1	42
OTU261	1	2	0	2	0	1	1	2	3	3	0	1	1	0	2	0	2	0	2	4	2	1	3	1	2	3	1	1	0	1	42
OTU262	3	0	0	2	2	1	3	5	0	1	0	1	2	3	1	0	0	1	1	2	4	1	2	0	0	1	0	1	0	5	42
OTU263	1	0	1	1	0	0	0	4	1	0	0	5	1	2	3	0	0	0	0	0	1	0	0	0	0	2	5	2	8	4	41
OTU264	5	1	2	1	4	8	3	3	2	0	0	0	4	1	1	1	0	0	1	2	1	0	0	0	0	1	0	0	0	0	41
OTU265	2	0	0	0	0	0	0	1	3	1	2	1	1	4	1	1	4	1	1	1	3	3	0	1	3	1	1	3	1	0	40
OTU266	5	0	1	0	0	0	2	3	0	2	0	2	2	0	0	1	0	1	6	2	7	0	1	0	0	0	0	4	0	1	40
OTU267	7	0	0	8	6	0	1	0	0	0	0	0	0	2	0	0	2	0	0	1	0	4	0	0	0	0	5	0	1	2	39
OTU268	11	0	1	5	0	3	2	3	0	0	0	0	0	0	0	0	0	0	0	1	0	0	0	0	6	1	2	3	0	1	39
OTU269	4	0	0	0	0	3	7	0	0	0	0	2	0	6	0	4	0	5	0	3	0	0	3	0	0	1	0	1	0	0	39
OTU270	4	0	2	0	1	1	5	1	3	1	3	2	2	0	2	1	0	1	1	1	0	3	0	0	1	0	3	0	0	1	39
OTU271	1	2	4	1	1	0	12	0	0	0	0	0	0	0	0	0	0	0	0	0	2	0	0	0	0	4	2	3	3	4	39
OTU272	1	0	0	0	4	0	0	0	4	0	0	1	2	3	0	0	0	0	4	1	0	10	1	0	7	0	0	0	0	0	38
OTU273	0	0	0	0	0	0	0	0	0	0	0	0	0	3	0	9	7	0	5	0	0	0	1	0	4	0	3	0	0	6	38
OTU274	4	0	2	1	0	4	0	3	0	2	0	2	1	0	0	0	0	0	0	0	0	0	0	0	8	3	3	0	0	4	37
OTU275	2	3	5	0	0	0	2	0	2	1	1	0	1	2	2	1	1	1	4	2	1	2	0	0	0	0	1	1	0	2	37

OTU276	3	3	0	0	7	0	0	4	1	0	0	0	0	4	0	6	1	0	5	0	1	0	0	0	0	0	0	1	0	1	37
OTU277	0	0	0	0	0	0	0	0	0	0	0	0	0	0	0	0	0	0	1	0	0	9	0	0	9	11	7	0	0	0	37
OTU278	1	0	0	2	2	3	2	3	2	0	0	6	0	0	1	1	2	0	1	0	4	2	0	2	1	0	0	1	1	0	37
OTU279	0	0	0	2	0	0	0	0	0	3	0	7	0	0	0	0	2	0	0	8	6	0	1	0	0	0	0	4	3	1	37
OTU280	0	0	0	1	1	1	0	0	0	5	8	7	0	0	0	0	0	0	0	0	0	0	0	0	2	0	0	5	2	5	37
OTU281	0	0	1	1	0	2	0	0	0	4	0	2	1	1	0	0	0	0	3	0	0	3	1	1	2	8	1	0	2	4	37
OTU282	12	6	0	2	2	0	0	0	0	0	0	1	0	4	0	0	0	0	0	0	0	5	0	0	0	0	2	0	1	1	36
OTU283	2	0	2	1	1	1	0	3	2	1	1	1	0	0	0	0	0	0	1	0	0	0	0	0	0	2	3	1	3	11	36
OTU284	2	1	0	2	1	1	1	1	1	1	2	1	1	1	2	1	2	2	0	3	0	2	1	2	0	3	2	0	0	0	36
OTU285	2	0	0	1	2	7	1	0	1	0	0	3	0	0	0	1	0	1	4	1	0	1	4	0	0	0	1	1	3	2	36
OTU286	8	0	0	0	3	0	0	0	0	0	0	0	0	0	0	0	0	0	0	1	6	0	3	0	1	13	0	0	1	0	36
OTU287	0	0	0	0	3	0	0	2	0	0	0	2	0	1	1	4	10	0	0	2	1	0	0	0	1	6	0	2	0	0	35
OTU288	12	0	0	1	1	0	0	0	0	1	0	4	0	2	0	0	0	0	0	1	0	0	1	2	0	4	0	4	1	1	35
OTU289	0	0	0	0	1	10	0	2	3	0	3	3	0	0	0	0	0	1	0	0	0	0	0	1	2	0	0	0	1	7	34
OTU290	1	0	0	2	0	3	0	0	2	0	0	2	0	4	2	2	0	0	1	2	1	0	2	3	3	0	0	2	0	2	34
OTU291	2	1	0	1	1	2	0	0	3	1	0	0	0	4	0	1	1	0	3	4	1	0	0	0	1	2	3	0	1	2	34
OTU292	14	0	0	0	1	0	0	0	0	0	0	0	0	2	0	2	0	6	1	0	0	2	1	0	0	0	3	1	1	0	34
OTU293	0	1	0	2	0	0	0	1	2	1	0	0	4	1	1	2	0	0	3	0	1	0	0	0	7	5	2	0	1	0	34
OTU294	0	0	0	0	0	3	0	0	1	3	0	9	0	0	1	3	0	2	0	0	0	0	2	0	2	0	0	1	2	5	34
OTU295	0	0	0	0	0	0	0	0	1	0	1	4	2	11	0	0	0	3	2	0	0	3	1	3	1	0	0	2	0	0	34
OTU296	0	0	0	5	5	0	0	0	0	0	0	0	2	0	0	6	5	1	2	0	0	0	0	0	4	0	1	0	3	0	34
OTU297	5	0	0	0	0	0	1	2	0	2	0	0	1	1	0	0	1	0	1	0	2	1	0	2	1	1	0	0	9	4	34
OTU298	0	0	2	0	1	2	0	3	1	1	0	2	1	2	8	0	1	1	2	0	1	0	0	0	2	2	0	0	1	0	33
OTU299	2	4	0	0	1	0	1	0	1	2	0	1	2	0	3	4	1	2	0	2	2	1	1	0	0	0	1	0	0	2	33
OTU300	2	3	2	0	0	1	0	0	0	2	0	0	1	5	0	2	2	0	1	1	3	2	0	0	1	0	1	1	2	1	33
OTU301	3	3	1	0	0	0	0	0	0	1	0	0	6	0	1	2	0	2	0	1	0	2	0	0	0	0	0	1	10	0	33

OTU302	0	8	0	3	0	5	0	0	0	0	0	0	0	0	0	0	0	0	6	0	3	0	0	0	0	0	0	0	0	7	32
OTU303	0	1	1	0	1	0	1	0	2	0	0	1	0	1	3	0	0	0	4	3	5	2	5	0	0	0	0	1	0	1	32
OTU304	0	1	0	0	2	5	1	0	0	1	0	3	0	4	1	1	1	2	2	3	1	2	0	0	0	0	0	0	0	2	32
OTU305	2	0	0	0	0	0	0	0	0	0	0	0	0	0	0	0	0	0	0	2	0	0	0	0	5	4	0	11	6	2	32
OTU306	2	0	1	0	2	1	0	1	2	3	0	0	4	4	1	1	0	0	4	0	1	3	1	0	0	0	0	0	1	0	32
OTU307	3	3	0	1	0	0	1	1	4	1	0	0	1	0	0	0	3	0	2	1	0	0	1	0	2	1	1	1	4	1	32
OTU308	0	1	1	0	0	0	0	0	3	0	0	0	0	0	1	2	0	0	4	6	0	4	0	0	0	0	0	3	2	5	32
OTU309	8	3	0	1	1	2	2	0	1	2	0	0	2	4	1	1	0	0	0	0	0	1	0	0	0	0	0	0	3	0	32
OTU310	0	0	0	6	0	0	0	8	0	0	0	4	0	0	0	2	4	0	0	0	0	0	0	1	0	1	0	0	6	0	32
OTU311	12	0	0	0	4	3	3	0	0	0	1	0	0	0	0	0	0	2	0	0	0	0	0	0	0	0	1	0	0	6	32
OTU312	8	0	0	3	0	0	2	2	0	3	0	0	1	0	0	1	1	0	0	3	0	2	0	2	0	0	0	0	0	4	32
OTU313	1	0	3	0	7	4	0	1	2	0	0	0	0	0	0	0	0	0	0	0	1	0	0	2	0	0	4	0	4	2	31
OTU314	0	0	0	1	0	0	0	0	0	1	4	1	0	0	1	2	4	1	5	6	0	0	1	0	0	0	1	0	0	3	31
OTU315	1	2	0	0	1	2	2	6	1	0	0	1	1	1	0	1	2	1	3	0	0	0	1	0	2	1	1	1	0	0	31
OTU316	1	3	0	0	0	0	1	1	4	1	1	2	2	1	1	1	2	1	1	3	1	0	1	0	0	2	0	1	0	0	31
OTU317	0	0	1	0	0	0	0	0	0	6	0	3	0	1	2	0	3	1	2	0	0	0	1	0	0	0	0	0	0	11	31
OTU318	1	0	0	3	1	0	1	1	0	0	1	3	1	0	0	0	0	0	2	7	0	0	0	0	2	0	1	0	2	5	31
OTU319	1	0	3	5	0	1	1	2	1	0	0	1	0	1	0	1	2	0	0	0	1	0	1	0	4	0	0	3	0	3	31
OTU320	0	0	0	0	0	0	0	1	0	0	0	1	26	0	0	0	0	0	1	1	0	0	0	0	0	0	0	0	1	0	31
OTU321	0	0	0	0	0	0	0	0	2	3	0	3	0	5	0	0	0	0	0	0	1	2	0	3	2	2	2	0	4	2	31
OTU322	4	5	0	0	0	0	1	5	0	0	0	0	0	3	0	1	3	2	0	0	0	1	1	0	0	0	1	0	0	3	30
OTU323	0	1	0	1	0	0	1	1	0	2	0	0	0	0	0	0	1	0	0	7	9	0	0	0	0	2	4	0	0	1	30
OTU324	1	3	0	0	0	1	0	9	0	1	0	1	0	0	0	1	4	0	0	5	0	1	0	0	0	0	0	0	2	0	29
OTU325	0	0	0	1	1	0	5	0	1	0	1	0	0	3	7	4	4	0	0	0	0	0	1	1	0	0	0	0	0	0	29
OTU326	8	0	1	1	0	1	0	3	0	0	0	0	0	0	0	5	0	0	0	5	0	0	0	0	0	0	0	3	2	0	29
OTU327	0	0	0	1	1	1	2	0	2	0	0	8	0	4	2	0	0	0	0	0	0	0	1	0	0	0	0	4	0	2	28

OTU328	0	0	2	0	0	0	0	0	1	0	0	0	0	1	0	0	0	0	1	0	0	0	0	0	9	0	0	5	2	7	28
OTU329	0	1	0	0	0	0	0	1	0	1	0	2	4	1	1	4	1	2	2	2	3	0	0	1	0	1	0	0	1	0	28
OTU330	0	1	1	0	1	0	0	1	1	0	0	4	0	3	4	1	0	0	1	3	1	0	0	0	0	1	2	1	0	2	28
OTU331	0	0	0	0	3	4	0	0	3	1	0	1	0	0	1	1	0	0	1	0	1	0	1	0	1	6	2	0	2	0	28
OTU332	0	0	0	4	0	0	0	0	0	0	0	0	2	1	0	4	0	5	8	0	0	0	2	0	0	0	0	2	0	0	28
OTU333	0	0	3	2	2	1	1	0	0	0	4	2	0	0	1	0	0	3	0	0	0	3	1	0	1	0	0	0	2	2	28
OTU334	1	2	0	0	1	0	1	0	1	6	0	0	3	0	3	0	0	0	1	1	0	2	1	1	2	0	1	1	0	0	28
OTU335	0	0	0	1	3	0	0	1	2	5	0	1	1	1	0	0	0	0	0	0	0	0	0	0	11	0	0	0	0	1	27
OTU336	0	0	1	3	0	2	3	1	0	2	0	0	0	3	5	1	0	3	0	0	0	1	0	0	1	0	0	0	0	1	27
OTU337	0	1	3	1	0	1	0	2	0	0	0	1	2	2	3	0	4	0	2	0	1	0	1	0	1	0	1	0	0	1	27
OTU338	0	0	0	1	2	0	0	0	0	0	0	0	2	3	0	0	0	0	2	0	0	0	0	3	0	0	7	1	6	0	27
OTU339	1	0	0	3	2	1	1	2	3	0	0	3	0	1	0	1	1	0	0	1	0	2	0	0	1	1	2	1	0	0	27
OTU340	2	0	0	2	0	2	0	2	2	6	0	0	0	1	0	3	0	1	0	0	1	0	0	2	0	0	0	0	2	1	27
OTU341	0	0	1	1	2	1	0	1	1	0	0	0	4	0	1	1	3	1	2	3	0	0	0	0	0	0	0	1	2	1	26
OTU342	0	0	0	0	0	0	0	0	0	0	0	0	0	0	0	7	0	0	14	0	0	0	0	1	0	0	3	0	0	1	26
OTU343	0	0	0	0	0	0	0	0	17	3	0	0	0	0	0	0	0	0	0	0	0	0	0	2	0	0	3	0	0	1	26
OTU344	2	3	2	0	0	0	2	1	4	0	0	1	0	0	0	0	0	0	0	0	0	0	1	1	2	1	1	5	0	0	26
OTU345	1	3	0	0	0	0	2	1	2	0	0	1	0	0	0	0	0	1	2	0	1	1	0	0	0	2	1	2	1	5	26
OTU346	3	0	0	0	1	0	2	0	0	0	0	0	2	2	0	1	0	0	5	0	0	0	4	0	3	0	2	1	0	0	26
OTU347	1	0	1	6	0	0	0	0	2	2	0	0	0	0	0	8	0	0	3	0	0	0	0	1	0	0	0	2	0	0	26
OTU348	0	0	0	2	0	1	0	1	1	1	0	1	1	3	0	2	0	1	1	2	0	2	3	0	0	2	0	0	1	1	26
OTU349	0	0	0	0	0	0	0	9	2	0	0	0	1	0	0	0	0	1	5	0	1	0	0	0	4	0	1	2	0	0	26
OTU350	4	0	0	0	0	0	2	0	0	0	0	0	1	0	0	2	0	0	2	0	0	1	4	0	1	4	0	2	1	1	25
OTU351	5	1	0	1	0	1	0	1	0	0	0	6	1	1	0	0	1	0	0	0	0	0	2	0	1	1	0	1	0	2	25
OTU352	0	1	0	0	0	2	1	0	1	0	0	1	1	0	2	0	1	0	5	2	0	0	2	0	1	0	1	1	1	2	25
OTU353	0	1	1	2	0	1	0	2	0	2	0	0	1	1	0	1	1	2	0	3	2	0	2	0	0	0	1	0	0	2	25

OTU354	1	4	0	0	0	2	0	4	3	1	1	3	1	2	0	0	0	2	0	1	0	0	0	0	0	0	0	0	0	0	25
OTU355	12	0	0	0	0	1	5	0	0	2	0	0	0	0	0	0	0	0	0	0	0	0	0	0	0	0	2	0	0	3	25
OTU356	0	0	0	0	0	0	0	0	0	0	0	0	0	0	0	0	0	0	1	0	1	0	0	0	0	5	18	0	0	0	25
OTU357	0	0	0	0	1	0	0	0	0	0	0	0	0	0	0	14	6	0	0	0	0	0	0	0	1	1	1	1	0	0	25
OTU358	2	3	1	0	0	0	1	2	0	0	0	2	0	0	1	2	0	1	0	2	1	0	0	0	3	1	0	1	1	1	25
OTU359	8	0	3	0	5	0	1	0	0	0	0	0	0	3	0	2	0	0	1	1	0	0	0	0	0	0	0	0	0	0	24
OTU360	4	0	0	2	0	0	1	0	0	0	0	0	0	0	4	0	10	0	0	0	0	0	0	0	0	3	0	0	0	0	24
OTU361	0	0	1	1	1	2	0	0	6	0	2	0	0	0	0	0	0	0	4	0	0	0	1	0	2	1	0	0	1	2	24
OTU362	0	0	0	1	0	1	0	2	1	0	0	0	0	0	0	0	0	0	0	0	0	4	0	0	0	0	13	0	1	1	24
OTU363	1	0	0	0	2	3	1	1	1	3	0	0	0	3	0	2	1	1	1	0	0	0	0	0	0	0	0	4	0	0	24
OTU364	1	2	0	0	0	0	0	0	1	2	2	0	0	1	0	0	1	1	1	0	1	1	0	2	2	0	1	2	2	1	24
OTU365	0	3	1	0	0	1	0	0	3	0	0	0	0	0	0	0	0	0	0	0	0	0	0	0	0	2	0	1	3	10	24
OTU366	0	0	0	0	0	0	0	0	1	0	0	5	0	2	0	0	0	8	2	0	0	0	0	0	1	2	0	0	1	2	24
OTU367	0	1	0	0	2	1	0	2	1	0	1	1	2	0	1	0	0	0	0	3	0	1	0	1	1	1	2	3	0	0	24
OTU368	0	0	1	1	0	3	0	0	0	0	0	2	2	0	4	1	0	0	1	2	1	1	0	1	1	0	1	1	1	0	24
OTU369	0	0	0	0	0	0	0	0	2	6	0	4	4	2	3	2	0	0	0	0	0	0	0	0	0	0	1	0	0	0	24
OTU370	1	0	0	0	2	0	2	1	1	0	2	1	0	0	1	0	1	0	0	0	0	0	1	0	3	0	1	3	1	2	23
OTU371	1	0	2	3	0	0	0	0	3	3	0	0	1	2	0	2	2	1	0	0	0	1	1	0	1	0	0	0	0	0	23
OTU372	0	0	0	0	0	1	0	0	0	0	0	15	3	0	0	3	0	0	0	0	0	0	0	0	0	0	0	0	0	1	23
OTU373	0	2	1	0	0	1	1	1	1	0	0	1	0	0	1	0	1	0	1	1	2	2	0	1	2	2	1	0	0	1	23
OTU374	3	2	0	0	0	0	5	0	0	0	0	2	0	0	0	2	0	0	0	3	2	0	4	0	0	0	0	0	0	0	23
OTU375	6	0	0	0	1	0	2	0	0	2	1	1	0	3	0	0	0	0	0	0	0	2	0	0	0	0	0	4	1	0	23
OTU376	0	0	0	0	0	0	0	0	0	0	0	0	0	0	1	0	0	0	1	0	1	0	0	0	2	1	4	3	5	5	23
OTU377	0	0	0	0	0	0	0	0	0	3	0	0	2	0	1	0	2	0	5	0	7	0	0	1	0	0	0	1	0	0	22
OTU378	0	0	0	0	0	0	0	0	0	0	0	4	0	0	0	0	0	0	0	0	0	0	0	0	8	4	6	0	0	0	22
OTU379	9	0	0	0	0	0	0	0	0	0	0	0	0	0	0	0	0	0	0	0	0	0	0	0	0	0	0	0	0	13	22

OTU380	0	1	0	0	0	0	0	0	0	0	0	0	0	0	0	3	0	1	0	10	0	5	2	0	0	0	0	0	0	0	22
OTU381	0	0	0	0	5	1	1	0	0	0	0	0	2	0	0	5	0	0	0	1	3	0	0	0	0	3	0	0	1	0	22
OTU382	1	0	0	1	1	0	2	0	2	0	0	4	0	0	1	1	1	1	0	0	0	1	0	0	2	1	0	0	1	2	22
OTU383	0	0	0	1	1	0	0	0	0	0	0	0	1	4	0	0	0	0	9	0	2	0	0	0	0	0	2	0	0	2	22
OTU384	0	1	0	0	0	0	2	0	4	0	2	0	2	3	0	1	3	1	0	0	2	0	1	0	0	0	0	0	0	0	22
OTU385	1	0	1	1	4	0	0	3	0	1	0	0	2	0	0	1	0	0	0	0	0	0	0	0	3	1	0	3	1	0	22
OTU386	3	0	1	0	0	0	0	0	0	0	0	0	0	3	0	1	0	0	2	2	0	0	0	0	0	0	3	1	3	3	22
OTU387	0	0	0	0	0	0	0	0	0	1	0	3	1	0	0	2	1	0	10	0	1	0	1	0	0	2	0	0	0	0	22
OTU388	0	0	0	0	0	4	0	0	0	0	0	0	0	0	1	0	0	1	0	0	0	2	3	2	0	5	0	2	0	2	22
OTU389	0	0	0	0	0	0	0	0	0	0	0	0	0	0	0	0	0	21	0	0	0	0	0	0	0	0	0	0	0	0	21
OTU390	2	0	0	0	0	0	0	0	4	0	0	9	3	0	0	0	0	0	0	0	0	0	0	0	1	0	2	0	0	0	21
OTU391	0	0	0	0	3	0	0	3	2	0	1	1	0	0	0	0	3	0	1	0	0	5	2	0	0	0	0	0	0	0	21
OTU392	2	1	1	0	0	0	9	0	3	0	0	0	0	0	0	0	0	0	0	0	2	0	0	0	0	0	0	0	3	0	21
OTU393	1	0	0	0	3	2	0	0	0	0	1	0	3	2	0	3	0	1	0	0	1	0	1	0	0	1	0	0	2	0	21
OTU394	0	0	1	2	0	2	2	1	0	0	0	0	0	0	3	0	1	0	3	2	0	0	1	0	3	0	0	0	0	0	21
OTU395	20	0	0	0	0	0	0	0	0	0	0	0	0	0	0	0	0	0	0	0	0	0	0	0	0	0	0	1	0	0	21
OTU396	0	0	0	0	0	0	0	0	0	0	0	0	0	0	0	0	6	3	8	1	3	0	0	0	0	0	0	0	0	0	21
OTU397	0	0	0	11	0	0	0	0	0	0	0	1	0	5	0	0	0	0	0	0	3	0	0	0	0	0	0	0	0	1	21
OTU398	0	0	0	9	0	0	0	0	0	2	0	0	5	0	0	0	0	0	0	0	5	0	0	0	0	0	0	0	0	0	21
OTU399	1	1	0	1	0	3	0	0	0	3	0	1	2	0	0	3	1	0	1	0	0	1	1	0	0	1	0	0	0	0	20
OTU400	0	3	0	0	2	0	0	0	0	0	0	0	0	1	0	0	0	0	2	2	0	0	1	0	5	0	0	0	0	4	20
OTU401	5	0	0	0	0	0	0	2	0	0	0	0	0	0	2	1	0	3	0	0	0	0	2	0	0	0	2	3	0	0	20
OTU402	2	2	1	1	1	0	1	0	2	1	0	0	0	1	0	1	0	0	0	1	0	2	0	0	1	0	0	2	1	0	20
OTU403	1	0	0	0	1	0	0	1	1	0	0	3	0	2	0	0	1	1	3	0	1	1	1	0	0	1	1	0	1	0	20
OTU404	0	0	0	0	1	5	7	0	0	0	0	2	0	0	0	0	1	0	0	2	0	0	0	0	1	0	0	0	1	0	20
OTU405	0	0	0	0	0	0	4	0	0	0	0	0	0	0	0	16	0	0	0	0	0	0	0	0	0	0	0	0	0	0	20

OTU406	0	0	2	1	0	0	0	0	0	3	3	0	0	0	0	0	1	0	0	0	3	0	0	0	1	0	2	1	1	2	20
OTU407	0	0	1	4	1	0	0	3	3	0	0	0	0	4	0	0	0	0	0	3	0	0	1	0	0	0	0	0	0	0	20
OTU408	0	0	1	1	0	0	2	2	1	0	1	0	2	0	0	0	0	0	1	0	0	0	1	2	0	2	1	0	2	1	20
OTU409	2	0	0	0	4	0	5	1	3	0	0	0	0	0	0	0	0	0	0	0	1	4	0	0	0	0	0	0	0	0	20
OTU410	3	0	0	0	0	0	0	7	0	0	0	0	3	0	0	0	0	0	0	0	0	0	0	0	0	0	6	1	0	0	20
OTU411	2	2	0	0	1	1	0	1	2	1	0	0	0	0	1	2	2	1	0	0	1	0	2	0	0	0	0	0	0	0	19
OTU412	0	8	0	3	2	0	0	0	0	0	0	2	0	0	0	0	0	0	0	2	2	0	0	0	0	0	0	0	0	0	19
OTU413	2	2	0	0	1	0	0	0	0	0	0	0	1	0	0	0	2	0	0	3	0	1	0	0	0	1	0	0	3	3	19
OTU414	0	0	1	0	0	0	0	2	3	0	0	0	0	0	1	3	0	0	0	0	3	0	5	0	0	0	0	0	1	0	19
OTU415	0	0	0	0	0	0	2	1	0	0	0	4	0	0	1	0	0	4	0	0	0	1	1	1	0	3	0	0	1	0	19
OTU416	0	0	0	0	0	2	0	1	1	0	1	1	1	1	3	1	0	0	0	0	1	1	0	0	1	1	1	0	1	1	19
OTU417	2	0	0	1	1	1	0	1	0	0	0	0	1	0	1	0	0	0	1	2	0	0	1	0	5	0	1	0	1	0	19
OTU418	1	1	1	0	0	3	0	1	2	0	0	2	0	1	0	3	0	0	0	1	1	1	0	0	0	0	1	0	0	0	19
OTU419	0	0	0	0	0	0	0	0	0	0	0	0	0	0	0	0	0	0	0	0	2	0	0	0	0	3	1	10	1	2	19
OTU420	0	0	0	0	1	0	0	0	0	2	0	1	0	3	1	2	0	0	0	0	0	0	2	0	0	0	1	1	2	3	19
OTU421	0	10	0	0	0	0	0	0	0	1	0	3	0	0	4	0	0	0	0	0	0	0	0	0	0	0	0	1	0	0	19
OTU422	3	0	0	0	0	0	1	0	0	3	1	0	0	1	0	1	1	0	1	0	0	0	0	0	0	4	0	1	2	0	19
OTU423	0	0	1	0	3	1	0	2	0	0	1	1	0	4	1	0	2	0	0	1	2	0	0	0	0	0	0	0	0	0	19
OTU424	0	0	0	0	0	0	0	0	0	2	5	0	8	0	3	0	0	1	0	0	0	0	0	0	0	0	0	0	0	0	19
OTU425	0	0	0	0	1	0	0	0	0	0	0	0	0	0	1	2	1	0	1	4	0	0	0	0	6	2	0	1	0	0	19
OTU426	0	0	0	1	0	0	2	3	0	0	1	0	2	0	0	0	2	2	0	0	0	1	2	1	0	0	0	1	1	0	19
OTU427	2	0	0	2	1	2	1	0	0	0	0	0	3	1	0	1	0	0	0	0	0	0	0	0	0	0	0	0	0	6	19
OTU428	0	0	1	0	6	0	0	0	0	0	0	0	0	5	0	0	0	0	0	0	3	4	0	0	0	0	0	0	0	0	19
OTU429	2	0	2	0	0	0	0	2	1	0	0	0	0	1	0	0	0	0	0	6	1	0	0	0	0	1	2	1	0	0	19
OTU430	0	0	5	0	0	0	0	2	0	2	0	1	1	2	0	0	0	0	1	0	0	0	0	1	0	0	0	0	0	3	18
OTU431	2	0	0	1	2	2	0	1	0	0	0	0	1	0	1	1	0	1	0	0	0	0	0	1	1	1	1	0	1	1	18

OTU432	0	0	0	0	0	1	1	0	0	0	1	0	0	0	0	4	0	0	0	1	1	2	0	1	4	0	0	1	0	1	18
OTU433	0	0	0	0	0	0	0	0	0	0	0	6	0	0	0	2	0	0	9	0	0	0	0	0	0	0	0	1	0	0	18
OTU434	0	0	0	0	0	0	0	0	0	6	0	0	0	0	0	0	0	0	0	0	0	0	0	0	5	0	0	7	0	0	18
OTU435	0	0	0	0	0	0	0	3	0	0	0	0	0	0	0	3	0	0	0	0	1	0	1	0	0	0	5	0	1	4	18
OTU436	0	0	0	0	2	1	2	1	0	0	0	2	0	1	1	1	0	0	1	1	1	0	0	0	0	2	0	1	0	1	18
OTU437	0	0	1	3	0	0	1	0	0	0	0	1	0	1	1	1	2	0	0	1	1	0	0	0	1	1	0	0	1	2	18
OTU438	5	0	0	1	0	0	1	0	0	0	1	0	0	0	0	0	0	0	0	0	4	3	1	0	0	0	1	0	0	1	18
OTU439	0	0	0	2	0	0	0	4	0	0	0	0	0	0	0	4	0	0	5	0	0	0	0	0	1	0	1	1	0	0	18
OTU440	2	1	0	0	1	0	0	2	0	1	0	1	1	0	0	1	0	1	1	0	0	0	0	0	0	2	2	2	0	0	18
OTU441	0	0	0	0	0	0	0	0	0	4	0	0	0	0	0	0	1	0	1	0	0	0	0	0	1	3	2	5	1	0	18
OTU442	0	0	0	0	0	0	0	6	0	0	0	0	0	0	11	0	0	0	0	0	0	0	0	0	0	0	0	0	0	0	17
OTU443	0	1	0	0	0	0	1	0	0	0	1	1	3	2	1	0	0	0	1	0	1	0	1	0	1	1	0	0	1	1	17
OTU444	0	0	0	1	0	1	3	3	0	0	0	0	0	1	0	1	0	0	2	0	0	0	0	0	0	1	2	0	0	2	17
OTU445	0	0	0	0	0	1	0	0	0	0	0	1	0	0	0	0	0	0	0	0	0	0	10	0	0	0	0	5	0	0	17
OTU446	0	0	0	2	3	0	0	0	0	5	0	0	0	0	0	2	0	0	0	2	0	1	0	0	0	0	2	0	0	0	17
OTU447	0	0	0	1	0	2	0	0	0	0	0	0	0	0	0	0	0	0	0	0	3	0	4	0	0	0	0	5	2	0	17
OTU448	1	0	1	0	2	3	0	0	1	0	0	0	1	0	0	1	0	0	1	1	0	1	0	1	2	0	1	0	0	0	17
OTU449	1	0	1	0	1	7	0	1	0	0	0	0	0	1	0	0	0	0	1	0	0	0	0	0	0	0	0	2	0	2	17
OTU450	2	0	0	6	0	0	0	0	1	1	0	0	0	0	0	0	0	0	0	0	0	1	0	0	0	0	3	1	0	2	17
OTU451	1	0	0	0	0	0	0	0	2	2	1	1	0	1	1	0	0	0	2	1	0	1	1	1	0	1	0	0	1	0	17
OTU452	3	0	0	0	0	0	0	0	0	0	0	0	3	1	0	0	1	0	1	1	0	0	0	0	4	0	0	0	1	2	17
OTU453	0	0	0	0	2	0	0	0	1	0	0	3	0	0	0	0	1	0	0	0	0	1	0	0	1	0	1	4	0	3	17
OTU454	0	0	1	0	2	0	0	0	1	0	0	0	0	0	0	2	0	0	0	0	0	0	1	8	0	0	2	0	0	0	17
OTU455	1	0	0	0	2	0	0	1	2	0	0	1	1	1	1	0	0	0	1	1	2	0	1	0	0	0	0	0	1	0	16
OTU456	2	0	0	1	0	0	0	0	1	1	0	1	1	0	0	0	0	0	0	0	0	0	0	0	0	2	0	0	2	5	16
OTU457	1	0	0	0	0	2	1	0	1	0	0	0	0	0	0	0	1	0	1	0	2	4	1	0	0	1	1	0	0	0	16

OTU458	3	0	0	0	0	0	0	2	3	0	0	0	0	0	1	3	0	0	2	0	1	0	1	0	0	0	0	0	0	0	16
OTU459	0	0	0	0	0	0	1	0	2	2	0	0	0	0	1	0	0	1	4	1	1	0	1	0	0	0	0	1	1	0	16
OTU460	0	0	0	0	0	0	0	0	0	0	1	0	3	0	4	0	1	0	3	2	0	0	0	0	2	0	0	0	0	0	16
OTU461	0	0	0	0	0	0	0	0	0	0	0	0	0	0	0	0	0	7	6	3	0	0	0	0	0	0	0	0	0	0	16
OTU462	0	0	0	0	3	0	0	0	0	0	0	1	0	0	0	0	0	0	0	0	0	0	0	0	0	0	3	3	5	1	16
OTU463	0	4	0	0	2	0	0	0	0	0	0	0	0	0	0	0	2	0	0	0	0	0	0	0	2	0	0	6	0	0	16
OTU464	0	0	0	4	1	0	0	0	0	0	0	0	0	2	0	0	0	1	0	0	1	3	1	0	0	0	0	0	0	3	16
OTU465	0	0	0	0	0	0	0	1	1	0	0	0	0	0	0	0	0	0	0	0	0	0	0	0	4	0	7	0	0	3	16
OTU466	0	0	0	1	3	0	0	0	1	0	1	1	0	0	0	0	0	1	1	0	0	0	4	0	2	0	0	0	0	0	15
OTU467	0	3	1	0	0	0	1	0	1	0	1	0	1	0	1	1	0	1	1	0	0	0	1	0	1	0	0	1	0	0	15
OTU468	0	0	0	1	0	1	0	1	0	1	1	1	0	0	1	0	1	0	0	1	1	0	2	0	0	1	0	1	0	1	15
OTU469	0	0	0	0	0	0	0	0	0	0	0	4	0	0	0	0	4	0	0	0	0	0	0	2	4	0	0	0	1	0	15
OTU470	0	1	0	0	0	0	0	0	0	1	0	1	0	2	2	0	1	0	1	0	0	0	0	0	0	1	2	2	1	0	15
OTU471	0	2	1	0	0	0	1	1	0	0	1	0	1	1	0	1	1	0	0	0	1	0	0	0	0	0	0	2	2	0	15
OTU472	0	0	0	0	2	0	3	0	6	0	0	0	3	0	0	0	0	0	0	0	0	0	0	0	1	0	0	0	0	0	15
OTU473	0	1	0	0	3	0	1	0	0	1	1	0	0	0	0	0	0	0	0	2	0	1	0	0	0	0	3	0	1	1	15
OTU474	0	0	1	1	0	0	0	0	0	0	0	0	0	1	2	0	3	0	1	0	2	1	0	0	0	1	1	0	1	0	15
OTU475	0	0	0	0	0	2	1	2	0	1	2	0	2	0	0	0	0	0	0	1	0	0	2	0	0	0	0	0	0	2	15
OTU476	0	2	0	0	0	0	2	0	1	0	0	0	1	0	0	0	5	0	0	0	0	0	0	0	0	3	0	0	1	0	15
OTU477	1	0	0	0	0	1	1	0	0	0	0	0	1	0	1	1	0	2	1	0	0	2	3	1	0	0	0	0	0	0	15
OTU478	0	0	0	1	1	0	1	0	2	2	1	0	1	1	0	0	0	0	1	2	0	0	0	0	0	1	0	1	0	0	15
OTU479	0	1	0	0	1	0	0	0	0	0	0	0	1	1	2	2	0	0	1	0	0	1	0	1	2	2	0	0	0	0	15
OTU480	0	0	0	0	0	0	0	0	1	1	0	5	1	0	0	0	0	2	0	0	0	0	5	0	0	0	0	0	0	0	15
OTU481	0	1	0	0	1	0	0	2	0	0	0	0	0	1	0	0	0	0	2	1	0	0	0	0	1	1	2	1	0	2	15
OTU482	1	0	0	0	0	0	1	0	0	0	1	2	0	0	0	1	0	2	1	0	1	1	1	0	1	0	1	1	0	0	15
OTU483	2	0	0	0	2	0	0	0	2	0	0	0	0	0	0	0	2	0	3	0	1	0	1	0	0	1	0	1	0	0	15

OTU484	0	0	0	3	0	0	0	1	0	0	0	5	0	0	0	1	0	0	0	0	0	0	1	0	0	4	0	0	0	0	15
OTU485	4	6	0	0	1	3	0	0	0	0	0	0	0	0	0	0	0	0	0	0	0	0	0	0	0	0	0	1	0	0	15
OTU486	1	0	0	1	0	0	1	1	0	1	0	0	0	0	0	2	1	0	1	1	0	1	0	0	1	2	0	1	0	0	15
OTU487	0	0	0	0	0	0	0	0	0	0	5	1	0	3	2	0	0	2	0	0	0	0	0	0	0	0	0	0	1	0	14
OTU488	4	0	0	0	3	0	0	0	1	0	0	0	0	2	0	0	2	0	0	0	0	0	0	0	0	0	0	0	2	0	14
OTU489	0	1	0	0	1	1	1	0	1	0	0	0	2	1	2	0	0	0	0	2	0	0	0	0	0	0	1	1	0	0	14
OTU490	0	0	0	0	3	0	0	0	0	2	1	0	0	0	1	0	0	1	1	1	0	0	0	0	0	0	0	4	0	0	14
OTU491	1	0	1	0	0	1	0	0	1	0	2	1	0	0	0	1	0	0	2	1	0	0	0	0	0	0	0	1	1	1	14
OTU492	0	0	0	1	1	1	2	0	1	0	1	0	0	0	1	1	0	0	0	1	0	1	0	0	0	1	0	0	2	0	14
OTU493	1	0	0	0	0	0	0	0	1	0	0	1	1	0	1	0	0	1	4	1	0	1	0	1	0	0	0	0	1	0	14
OTU494	4	0	0	0	0	0	0	0	0	2	0	0	3	0	2	0	0	0	1	0	0	1	0	0	0	0	1	0	0	0	14
OTU495	1	0	0	0	0	1	0	3	0	0	0	1	0	1	2	0	0	0	1	0	0	0	0	0	1	1	0	1	0	1	14
OTU496	1	0	0	0	1	1	0	0	1	0	0	0	0	2	1	0	2	0	0	2	0	0	0	1	1	0	0	1	0	0	14
OTU497	0	2	0	3	1	0	0	0	0	0	0	0	1	0	1	0	0	1	0	2	1	0	0	0	0	0	0	2	0	0	14
OTU498	1	0	0	0	0	1	0	0	2	0	4	0	0	0	0	0	0	0	4	0	0	0	0	0	2	0	0	0	0	0	14
OTU499	6	0	0	0	0	1	0	0	0	1	1	1	0	0	0	0	0	0	0	1	0	0	0	0	0	0	0	0	1	2	14
OTU500	2	0	0	0	0	0	1	1	0	5	0	0	0	0	0	1	1	0	1	0	0	0	0	1	1	0	0	0	0	0	14
OTU501	0	0	0	0	0	1	0	2	1	0	0	1	0	2	0	0	1	0	0	0	2	0	0	0	4	0	0	0	0	0	14
OTU502	0	0	0	0	0	1	0	0	1	2	2	1	0	1	0	0	2	0	0	0	0	0	1	0	0	0	2	1	0	0	14
OTU503	0	0	0	1	0	1	0	0	2	1	0	0	0	0	0	0	0	0	0	0	0	0	0	1	0	4	3	1	0	0	14
OTU504	0	0	0	0	0	0	0	0	0	2	1	0	0	0	0	0	0	0	1	0	0	2	0	4	2	2	0	0	0	0	14
OTU505	0	4	2	0	0	0	0	1	0	2	0	3	0	0	0	0	0	0	0	0	0	2	0	0	0	0	0	0	0	0	14
OTU506	1	0	0	0	0	0	0	0	1	1	0	1	0	0	0	0	0	0	1	0	3	0	0	3	0	0	1	0	1	1	14
OTU507	0	0	1	0	0	0	0	0	0	1	0	0	0	0	0	0	0	0	0	0	2	1	1	0	1	0	0	1	1	5	14
OTU508	0	0	0	0	0	0	0	1	0	0	0	0	0	1	0	1	1	1	4	0	0	0	3	0	0	0	0	1	1	0	14
OTU509	0	1	0	0	2	0	1	0	2	0	0	0	0	1	0	0	0	0	0	0	0	1	0	2	1	2	0	1	0	0	14

OTU510	0	0	1	0	1	0	3	0	0	0	0	0	1	0	0	4	0	0	1	1	1	0	0	0	0	0	0	0	0	0	13
OTU511	0	1	1	0	0	3	0	0	3	1	0	0	0	0	1	0	1	0	0	0	0	0	0	0	0	0	1	0	0	1	13
OTU512	3	0	0	1	0	0	0	4	0	0	1	0	0	0	0	2	1	0	1	0	0	0	0	0	0	0	0	0	0	0	13
OTU513	5	0	1	0	0	2	0	0	0	0	0	0	1	0	0	1	0	0	1	1	0	0	0	0	0	0	1	0	0	0	13
OTU514	0	0	0	0	0	1	0	0	1	4	0	0	1	0	0	0	0	0	0	0	0	0	0	2	3	0	0	1	0	0	13
OTU515	2	0	0	7	0	0	0	0	0	0	0	0	2	0	0	0	0	0	0	0	0	0	0	0	0	0	0	2	0	0	13
OTU516	0	0	5	0	0	0	0	0	8	0	0	0	0	0	0	0	0	0	0	0	0	0	0	0	0	0	0	0	0	0	13
OTU517	0	0	0	0	0	0	2	0	0	0	0	0	0	0	6	0	0	0	0	0	0	0	5	0	0	0	0	0	0	0	13
OTU518	1	0	0	0	0	0	0	0	1	2	0	0	0	0	0	0	0	1	0	6	0	0	0	0	0	0	0	0	2	0	13
OTU519	1	1	0	0	1	0	1	0	1	0	1	1	2	0	1	2	0	0	0	0	0	0	0	0	1	0	0	0	0	0	13
OTU520	0	2	0	0	0	0	0	0	0	0	0	0	0	2	1	2	0	0	2	1	0	0	0	1	0	0	0	0	0	2	13
OTU521	1	2	0	2	0	1	0	0	0	7	0	0	0	0	0	0	0	0	0	0	0	0	0	0	0	0	0	0	0	0	13
OTU522	0	0	0	0	0	0	1	7	1	0	0	0	0	0	1	0	0	2	0	0	0	0	0	0	0	0	0	1	0	0	13
OTU523	0	1	0	0	0	1	0	1	0	1	0	2	0	1	0	0	0	1	0	0	3	0	0	0	1	0	0	1	0	0	13
OTU524	2	1	0	1	0	1	0	1	2	0	0	2	0	0	0	0	0	0	0	0	1	0	0	0	1	0	0	0	1	0	13
OTU525	0	0	1	0	0	0	0	0	0	1	0	0	0	1	0	0	0	0	0	2	1	0	0	0	2	0	1	0	3	1	13
OTU526	0	0	0	0	0	0	0	2	3	0	0	0	0	0	0	2	1	0	0	1	0	0	0	1	0	0	0	0	0	3	13
OTU527	0	0	0	0	0	1	0	0	1	0	0	3	0	1	1	0	0	1	0	0	0	0	1	0	0	0	1	3	0	0	13
OTU528	5	0	0	0	0	8	0	0	0	0	0	0	0	0	0	0	0	0	0	0	0	0	0	0	0	0	0	0	0	0	13
OTU529	0	0	0	0	0	0	0	0	0	0	0	7	0	0	0	0	0	0	0	0	0	0	0	3	0	0	0	0	0	3	13
OTU530	0	0	0	0	0	0	0	0	0	0	0	0	0	0	0	0	0	0	0	0	0	0	0	0	12	0	0	1	0	0	13
OTU531	0	7	1	0	0	0	0	0	0	0	0	3	0	0	0	2	0	0	0	0	0	0	0	0	0	0	0	0	0	0	13
OTU532	0	0	0	0	0	0	1	0	0	0	0	3	0	0	0	0	0	0	0	0	0	1	6	0	0	0	1	0	0	0	12
OTU533	4	0	0	0	0	0	0	0	0	0	0	0	0	0	0	0	0	0	0	0	0	0	5	0	0	0	0	3	0	0	12
OTU534	0	0	1	0	0	1	1	0	0	3	0	2	0	2	0	0	1	0	0	0	0	0	1	0	0	0	0	0	0	0	12
OTU535	1	0	0	1	0	1	0	0	0	0	0	0	0	2	0	1	1	0	0	1	1	0	1	0	1	1	0	0	0	0	12

OTU536	0	0	0	3	0	0	0	1	0	0	0	1	1	0	0	1	0	0	0	0	1	0	0	0	3	0	0	1	0	0	12
OTU537	0	0	0	4	0	0	0	0	0	0	0	1	0	0	0	1	0	0	3	0	0	0	0	2	0	1	0	0	0	0	12
OTU538	0	0	1	0	0	1	2	0	0	0	1	0	0	1	0	0	0	0	0	1	0	0	0	0	0	1	0	2	0	2	12
OTU539	0	0	0	0	0	0	0	0	0	1	0	4	0	0	0	0	0	0	0	0	0	0	0	0	3	1	0	3	0	0	12
OTU540	0	0	0	0	0	2	0	0	1	0	0	0	1	4	0	2	0	0	1	1	0	0	0	0	0	0	0	0	0	0	12
OTU541	0	1	1	0	0	1	0	0	0	0	0	0	0	1	0	3	1	1	1	0	0	0	1	0	0	0	0	0	1	0	12
OTU542	1	0	0	2	0	0	0	0	1	0	2	0	1	0	0	0	1	0	1	1	0	0	0	0	0	0	0	1	1	0	12
OTU543	0	0	0	0	1	1	0	0	0	0	1	1	0	0	1	1	0	1	0	0	0	0	0	0	2	0	0	1	1	1	12
OTU544	2	3	0	0	0	1	0	0	1	0	1	1	0	0	0	0	0	0	0	0	0	0	0	0	0	0	0	2	1	0	12
OTU545	0	0	1	0	2	0	0	1	3	2	0	0	0	0	0	0	1	0	0	0	0	0	0	1	0	1	0	0	0	0	12
OTU546	0	0	0	0	0	0	1	1	0	0	0	4	2	0	0	0	0	0	0	0	0	0	1	0	0	0	0	0	0	3	12
OTU547	0	0	0	0	0	1	0	0	0	0	0	1	1	0	1	3	0	0	0	0	0	0	0	0	2	0	0	0	1	2	12
OTU548	0	0	0	0	1	0	0	0	1	1	0	0	0	0	0	1	4	1	0	0	1	0	0	0	0	0	0	0	2	0	12
OTU549	0	1	0	0	0	0	0	0	1	0	1	1	0	2	0	0	0	2	0	0	0	0	0	0	1	0	1	1	0	1	12
OTU550	0	0	0	0	1	0	0	1	0	0	0	0	2	0	0	1	3	0	0	0	0	0	2	0	0	1	0	0	0	1	12
OTU551	0	0	0	0	0	0	1	1	0	0	0	0	1	0	0	0	0	0	0	0	0	0	2	0	0	5	0	0	2	0	12
OTU552	0	0	1	1	0	0	0	0	0	0	0	0	1	4	0	1	1	0	0	0	0	0	0	0	1	1	0	0	1	0	12
OTU553	1	0	0	3	0	0	1	0	1	0	2	0	1	0	0	1	0	0	0	0	1	0	0	0	0	0	0	0	0	1	12
OTU554	0	0	0	0	0	0	1	0	0	0	0	1	0	0	0	0	0	0	0	3	1	4	0	0	0	0	0	1	0	1	12
OTU555	0	0	0	0	0	0	0	0	0	0	3	0	0	0	0	0	0	0	0	8	0	0	0	1	0	0	0	0	0	0	12
OTU556	10	0	0	0	0	0	0	0	0	0	0	1	0	0	0	0	0	0	0	0	0	0	0	0	0	1	0	0	0	0	12
OTU557	0	2	0	0	1	2	0	0	0	0	0	0	0	0	0	0	0	0	3	1	0	0	0	0	2	0	0	0	0	0	11
OTU558	0	0	0	0	0	0	0	4	0	0	0	0	0	0	0	0	0	0	0	0	0	0	0	0	0	0	1	0	3	3	11
OTU559	2	0	0	0	0	2	0	0	0	1	1	0	0	0	0	2	0	1	0	0	0	0	0	0	0	0	0	1	1	0	11
OTU560	1	0	0	1	0	0	0	0	0	0	0	0	0	1	1	0	0	0	2	2	2	0	1	0	0	0	0	0	0	0	11
OTU561	0	0	0	0	0	1	0	0	0	1	0	1	0	2	1	0	0	0	1	1	0	3	0	0	0	0	0	0	0	0	11

OTU562	0	1	0	0	2	0	0	2	0	0	0	0	0	1	0	1	0	0	1	0	0	0	0	0	1	0	0	1	0	1	11
OTU563	0	0	0	0	0	1	0	0	1	1	1	0	0	2	0	1	0	0	0	0	2	1	0	0	0	0	1	0	0	0	11
OTU564	0	0	0	0	0	0	0	0	0	0	0	1	0	0	1	2	0	1	0	1	1	0	2	0	0	0	1	0	1	0	11
OTU565	1	0	0	0	1	0	0	1	0	0	0	1	0	0	3	1	0	1	1	1	0	0	0	0	0	0	0	0	0	0	11
OTU566	1	2	0	0	0	0	0	0	0	0	0	0	1	3	0	0	0	1	0	0	2	0	0	0	0	0	0	1	0	0	11
OTU567	1	0	0	0	0	0	0	0	0	1	0	0	1	0	2	0	0	0	0	0	3	0	0	1	0	0	0	0	1	1	11
OTU568	0	0	0	0	0	3	0	0	2	0	0	0	1	1	1	0	0	0	0	0	0	0	0	0	0	0	0	3	0	0	11
OTU569	0	0	1	0	0	0	0	0	0	0	0	0	0	0	0	0	0	0	0	0	0	0	0	0	0	0	2	0	5	3	11
OTU570	0	0	0	0	0	2	0	0	0	0	0	0	1	0	1	1	2	0	1	0	0	0	0	0	2	0	0	0	1	0	11
OTU571	0	0	0	0	0	0	1	1	0	2	0	0	0	0	1	0	5	0	1	0	0	0	0	0	0	0	0	0	0	0	11
OTU572	0	0	0	0	0	0	1	0	0	0	0	0	0	0	1	0	0	0	1	1	0	1	4	0	0	0	1	1	0	0	11
OTU573	1	0	1	0	0	0	0	0	0	0	0	2	0	0	1	2	0	1	0	0	1	0	0	0	0	0	0	0	1	1	11
OTU574	0	0	0	0	0	0	0	1	0	3	0	6	0	0	0	0	0	0	0	0	0	0	0	1	0	0	0	0	0	0	11
OTU575	0	0	0	0	0	0	0	0	0	0	0	0	0	0	0	0	0	0	2	0	0	0	0	0	0	4	0	5	0	0	11
OTU576	0	0	0	0	0	1	0	3	0	0	0	0	0	0	0	0	0	1	1	0	0	3	0	0	0	0	2	0	0	0	11
OTU577	0	0	0	0	0	0	0	0	1	0	0	0	0	0	0	0	0	0	0	0	4	0	5	0	0	1	0	0	0	0	11
OTU578	0	0	0	0	0	0	0	0	2	0	0	0	0	0	0	0	0	0	0	0	0	1	0	0	2	2	0	2	1	1	11
OTU579	0	0	0	2	0	0	0	0	0	0	2	0	0	0	1	2	0	0	0	0	0	3	0	0	0	0	0	1	0	0	11
OTU580	0	0	0	0	0	0	1	0	1	0	1	0	0	1	0	1	3	0	0	1	1	0	0	0	0	1	0	0	0	0	11
OTU581	0	0	0	0	0	0	0	0	0	0	0	0	0	0	5	0	0	0	0	0	0	0	0	0	0	6	0	0	0	0	11
OTU582	0	0	0	0	0	0	0	0	0	0	0	4	0	0	0	0	0	0	6	0	0	0	0	0	0	0	0	1	0	0	11
OTU583	1	0	0	1	0	2	0	0	0	0	0	2	0	0	0	0	0	0	0	0	0	3	1	0	0	0	0	0	0	1	11
OTU584	0	0	0	0	0	1	0	0	0	1	0	0	0	0	0	0	0	0	1	2	3	1	0	1	1	0	0	0	0	0	11
OTU585	2	0	0	0	0	1	0	0	0	0	0	0	0	0	0	0	1	0	3	1	0	1	0	0	1	0	0	0	0	0	10
OTU586	0	0	0	0	0	0	0	0	0	2	0	0	0	0	0	0	0	0	0	8	0	0	0	0	0	0	0	0	0	0	10
OTU587	0	0	0	0	0	3	0	0	3	2	0	0	0	1	0	1	0	0	0	0	0	0	0	0	0	0	0	0	0	0	10

OTU588	0	0	0	0	0	0	0	0	0	0	0	1	0	0	0	2	0	0	0	1	0	2	3	0	0	1	0	0	0	0	10
OTU589	0	0	1	0	0	1	0	0	0	0	0	0	0	1	0	1	1	1	1	0	0	0	0	0	0	0	2	1	0	0	10
OTU590	3	0	0	0	0	1	1	0	0	0	1	2	0	1	0	0	0	0	0	0	1	0	0	0	0	0	0	0	0	0	10
OTU591	0	1	0	1	1	0	0	0	0	0	0	0	0	0	0	0	1	0	0	1	0	0	3	0	0	1	0	0	0	1	10
OTU592	0	0	0	0	0	0	0	0	0	0	0	0	0	0	0	0	5	0	0	0	0	0	0	0	0	0	5	0	0	0	10
OTU593	2	0	0	0	0	0	0	0	0	4	0	1	0	0	0	0	0	0	0	0	1	0	2	0	0	0	0	0	0	0	10
OTU594	0	1	0	0	1	0	1	0	0	0	0	0	0	2	0	0	0	0	1	2	1	0	0	0	0	0	0	0	1	0	10
OTU595	0	0	0	0	0	0	0	0	0	3	0	0	4	0	0	0	0	0	1	0	0	0	1	0	0	0	1	0	0	0	10
OTU596	0	0	0	0	0	0	3	0	0	0	0	2	0	0	0	0	0	0	0	0	0	0	3	1	1	0	0	0	0	0	10
OTU597	0	0	0	1	0	0	1	0	0	0	0	0	0	0	0	0	5	0	0	0	0	0	2	1	0	0	0	0	0	0	10
OTU598	0	0	0	0	0	0	0	0	0	0	0	0	0	0	0	0	0	0	0	5	0	0	0	0	0	0	0	0	0	5	10
OTU599	0	0	0	10	0	0	0	0	0	0	0	0	0	0	0	0	0	0	0	0	0	0	0	0	0	0	0	0	0	0	10
OTU600	0	0	0	0	1	0	0	0	0	2	0	0	0	0	2	1	0	0	0	0	0	3	0	0	0	0	0	0	1	0	10
OTU601	0	0	0	0	0	0	0	1	2	0	0	1	0	1	1	1	0	0	1	1	0	0	0	1	0	0	0	0	0	0	10
OTU602	0	0	0	0	0	0	0	0	0	0	0	0	0	0	0	0	0	0	0	0	7	0	0	0	0	0	0	0	2	1	10
OTU603	0	0	0	0	0	0	0	0	0	3	0	0	0	0	0	0	0	0	0	0	0	6	0	0	0	0	0	0	1	0	10
OTU604	0	0	0	0	0	0	0	1	2	2	0	0	0	0	0	0	1	0	0	0	0	0	0	1	0	0	0	0	2	1	10
OTU605	1	1	1	0	0	0	0	1	0	0	1	0	0	0	0	0	0	0	0	0	1	0	1	0	3	0	0	0	0	0	10
OTU606	9	0	0	0	0	0	0	0	0	0	0	0	0	0	0	0	0	0	0	0	0	0	0	0	0	0	0	0	0	0	9
OTU607	1	0	1	0	0	0	0	0	0	0	0	0	2	0	1	0	0	0	1	0	1	0	0	0	0	0	0	0	0	2	9
OTU608	0	0	0	0	0	0	0	0	0	0	0	1	2	1	0	0	0	0	1	0	0	2	0	0	1	0	0	0	0	1	9
OTU609	0	0	1	0	2	2	0	0	1	0	0	0	0	0	0	1	1	0	0	1	0	0	0	0	0	0	0	0	0	0	9
OTU610	0	0	0	0	0	2	0	0	2	0	3	0	1	0	1	0	0	0	0	0	0	0	0	0	0	0	0	0	0	0	9
OTU611	0	0	0	0	1	0	0	0	0	2	0	0	0	0	2	0	0	0	1	0	0	0	0	0	1	0	0	2	0	0	9
OTU612	2	0	0	0	0	0	1	0	0	0	0	0	0	0	0	1	1	0	0	1	0	3	0	0	0	0	0	0	0	0	9
OTU613	0	0	3	0	0	0	0	1	0	0	0	1	0	0	0	0	0	0	0	0	0	0	0	0	0	2	0	0	2	0	9

OTU614	0	0	0	0	1	0	0	0	0	0	0	0	0	0	0	0	0	3	0	0	0	0	0	0	1	0	0	0	4	0	9
OTU615	0	0	0	0	0	1	0	1	1	0	0	1	0	0	1	0	0	0	3	0	0	0	0	0	0	0	0	1	0	0	9
OTU616	2	0	0	0	1	0	0	0	1	0	0	0	0	1	0	0	0	0	0	0	3	0	0	0	0	0	0	0	1	0	9
OTU617	0	0	0	0	1	0	0	0	0	0	0	0	2	0	1	0	0	0	0	0	0	0	0	1	0	1	0	1	2	0	9
OTU618	0	0	0	0	0	0	0	0	0	2	0	0	0	0	0	0	0	0	0	0	0	7	0	0	0	0	0	0	0	0	9
OTU619	0	0	0	0	1	0	0	0	0	0	0	0	4	0	0	0	0	0	1	0	0	0	0	1	0	0	0	2	0	0	9
OTU620	0	0	0	0	0	1	2	0	1	0	0	0	0	0	2	0	0	1	0	1	0	0	0	0	0	0	1	0	0	0	9
OTU621	3	0	0	0	0	0	1	0	0	4	0	0	0	0	0	0	0	0	0	0	0	1	0	0	0	0	0	0	0	0	9
OTU622	0	0	0	0	0	0	3	0	0	1	0	0	0	0	2	0	3	0	0	0	0	0	0	0	0	0	0	0	0	0	9
OTU623	0	0	0	0	0	0	0	0	0	0	0	1	3	1	1	1	0	1	0	1	0	0	0	0	0	0	0	0	0	0	9
OTU624	0	2	0	1	0	0	0	0	0	0	0	0	0	0	0	0	0	0	0	0	0	0	0	0	0	0	0	0	6	0	9
OTU625	0	0	0	0	0	0	0	0	1	0	1	0	0	0	2	0	1	0	0	1	1	1	0	0	0	0	0	0	1	0	9
OTU626	1	0	0	0	2	0	0	0	0	0	0	0	0	0	4	0	0	0	0	1	0	0	0	0	1	0	0	0	0	0	9
OTU627	0	0	0	0	0	0	0	0	0	0	0	0	0	0	0	0	2	0	0	1	0	0	0	0	3	0	0	0	0	3	9
OTU628	0	0	0	0	0	3	0	0	2	0	0	0	0	0	0	0	4	0	0	0	0	0	0	0	0	0	0	0	0	0	9
OTU629	0	0	0	0	0	0	0	1	0	1	0	0	0	0	0	0	0	0	2	0	0	0	0	0	0	0	0	1	4	0	9
OTU630	0	0	0	0	0	0	0	0	0	0	0	4	0	0	0	0	0	0	0	0	3	0	0	2	0	0	0	0	0	0	9
OTU631	0	0	0	0	0	0	3	0	1	0	3	2	0	0	0	0	0	0	0	0	0	0	0	0	0	0	0	0	0	0	9
OTU632	0	0	0	0	1	0	0	0	0	0	2	0	0	0	0	0	0	0	0	0	1	0	1	0	0	1	0	0	3	0	9
OTU633	0	0	0	0	0	0	0	0	0	0	0	0	0	2	0	0	0	0	0	0	0	0	0	0	7	0	0	0	0	0	9
OTU634	1	0	0	0	3	0	0	0	0	0	0	0	0	1	0	0	1	2	0	0	0	0	0	0	0	0	0	1	0	0	9
OTU635	0	0	0	0	0	0	0	3	2	0	0	0	0	1	0	1	0	0	0	0	0	0	0	0	1	0	1	0	0	0	9
OTU636	0	0	0	0	0	0	0	0	0	1	1	0	0	0	4	0	3	0	0	0	0	0	0	0	0	0	0	0	0	0	9
OTU637	0	0	0	0	0	0	0	0	0	0	0	0	0	0	0	0	0	0	0	0	0	0	0	0	0	0	0	0	0	9	9
OTU638	0	0	0	2	0	0	1	1	0	0	0	0	1	0	0	0	2	0	0	1	0	0	0	0	1	0	0	0	0	0	9
OTU639	0	0	0	0	0	0	0	4	1	1	0	1	0	0	0	1	0	0	0	0	0	0	1	0	0	0	0	0	0	0	9

OTU640	0	0	0	0	7	0	0	0	0	0	0	1	0	0	0	0	0	0	0	0	0	0	0	0	0	0	0	0	0	0	8
OTU641	0	0	1	1	1	0	0	0	0	0	0	0	0	0	0	1	2	0	0	0	0	0	0	0	0	0	1	0	0	1	8
OTU642	1	0	0	0	2	1	0	0	0	0	0	0	0	0	0	2	0	0	0	0	1	0	0	1	0	0	0	0	0	0	8
OTU643	0	0	0	1	1	0	0	0	0	0	0	0	0	0	0	0	0	2	3	0	0	0	0	0	0	0	0	1	0	0	8
OTU644	0	0	0	0	1	2	0	0	0	0	0	0	0	0	0	2	1	0	0	2	0	0	0	0	0	0	0	0	0	0	8
OTU645	0	0	0	1	0	0	0	0	0	0	0	0	0	0	1	0	0	0	0	0	1	2	0	1	1	0	0	0	1	0	8
OTU646	1	0	2	0	0	0	0	0	0	1	0	1	0	0	0	1	0	0	1	0	0	0	0	0	1	0	0	0	0	0	8
OTU647	0	0	0	0	0	0	0	1	0	0	0	1	0	0	0	0	0	0	0	0	0	0	0	0	1	0	2	1	0	2	8
OTU648	0	1	1	0	0	0	0	0	0	0	0	0	0	1	0	0	0	0	1	1	0	1	0	0	0	0	2	0	0	0	8
OTU649	2	0	0	0	0	0	0	0	0	0	0	0	0	0	0	0	0	0	0	0	0	0	6	0	0	0	0	0	0	0	8
OTU650	0	0	0	0	0	0	0	0	0	0	0	0	0	0	0	0	0	8	0	0	0	0	0	0	0	0	0	0	0	0	8
OTU651	0	0	0	0	0	0	0	4	4	0	0	0	0	0	0	0	0	0	0	0	0	0	0	0	0	0	0	0	0	0	8
OTU652	0	1	0	0	0	1	0	0	0	0	0	0	0	0	1	0	1	0	0	0	2	0	1	0	0	1	0	0	0	0	8
OTU653	1	0	0	0	1	0	0	0	0	1	0	1	0	0	0	0	3	0	0	0	0	0	0	0	1	0	0	0	0	0	8
OTU654	1	0	0	0	0	2	0	1	0	0	1	0	0	0	0	0	0	1	0	1	0	0	0	1	0	0	0	0	0	0	8
OTU655	0	0	0	1	0	0	0	0	1	0	0	0	0	0	0	1	0	0	1	1	0	0	0	0	2	0	0	0	0	1	8
OTU656	0	0	0	0	0	0	0	0	0	0	0	0	0	0	0	0	0	0	0	0	8	0	0	0	0	0	0	0	0	0	8
OTU657	0	0	0	0	0	0	0	0	7	0	0	0	0	0	0	0	0	0	0	0	0	0	0	0	0	0	1	0	0	0	8
OTU658	0	0	0	1	0	0	0	0	1	0	0	0	0	1	1	0	0	3	0	0	0	0	0	0	0	0	0	1	0	0	8
OTU659	0	0	0	0	0	0	0	0	0	0	3	2	0	0	2	0	0	0	0	0	1	0	0	0	0	0	0	0	0	0	8
OTU660	0	0	1	0	0	0	0	0	0	0	0	0	0	1	0	1	0	0	1	0	0	1	0	0	2	1	0	0	0	0	8
OTU661	0	3	0	0	0	0	0	1	0	0	0	0	0	0	1	0	0	0	0	0	0	0	0	0	1	0	2	0	0	0	8
OTU662	1	0	0	0	0	0	0	0	1	1	0	0	1	1	0	0	0	1	0	1	0	0	0	0	0	0	1	0	0	0	8
OTU663	0	1	0	0	0	0	0	1	2	0	0	0	0	0	0	0	0	0	0	2	1	1	0	0	0	0	0	0	0	0	8
OTU664	1	0	1	0	0	0	1	0	0	0	2	0	0	0	0	0	0	1	0	0	0	0	0	0	0	0	0	2	0	0	8
OTU665	0	0	0	0	1	0	1	4	0	0	0	0	1	0	0	0	0	0	0	0	0	0	0	0	0	1	0	0	0	0	8

OTU666	0	0	0	0	0	0	0	0	0	0	0	0	0	0	0	0	0	0	0	0	0	0	0	0	0	0	0	8	0	0	8
OTU667	0	0	1	0	1	1	0	0	0	1	0	0	0	0	0	0	0	0	0	0	1	0	0	1	1	0	0	0	1	0	8
OTU668	0	5	0	0	3	0	0	0	0	0	0	0	0	0	0	0	0	0	0	0	0	0	0	0	0	0	0	0	0	0	8
OTU669	1	1	0	0	0	0	0	1	0	0	0	0	0	0	1	0	0	0	2	0	0	0	0	0	0	0	0	1	0	1	8
OTU670	0	0	0	0	0	0	0	0	0	0	0	0	0	0	0	4	0	0	0	0	0	0	0	0	4	0	0	0	0	0	8
OTU671	0	1	0	0	0	0	0	0	0	0	1	0	0	1	0	0	1	0	0	0	0	0	2	0	1	0	0	0	0	1	8
OTU672	0	1	0	0	0	0	0	1	0	0	0	0	0	0	0	0	0	0	1	0	3	0	1	0	0	1	0	0	0	0	8
OTU673	0	0	0	0	0	0	0	0	1	1	0	1	0	0	0	0	1	0	0	0	0	1	0	0	0	1	0	0	1	1	8
OTU674	0	1	0	2	0	0	0	0	0	0	0	0	0	0	0	0	0	0	0	0	0	1	1	0	0	0	1	0	2	0	8
OTU675	1	0	0	0	0	0	0	0	0	2	0	1	0	0	1	0	0	0	1	0	0	0	0	0	0	1	0	0	1	0	8
OTU676	0	0	0	0	0	0	0	0	0	0	0	0	0	0	0	0	0	5	3	0	0	0	0	0	0	0	0	0	0	0	8
OTU677	0	0	0	1	0	1	0	0	0	0	1	2	0	0	0	0	1	0	0	0	0	0	0	0	0	1	1	0	0	0	8
OTU678	2	0	0	0	0	0	0	0	0	3	0	3	0	0	0	0	0	0	0	0	0	0	0	0	0	0	0	0	0	0	8
OTU679	0	0	0	0	3	0	0	0	0	0	0	0	0	0	0	2	0	0	0	0	0	0	0	0	1	1	1	0	0	0	8
OTU680	1	0	0	0	0	0	0	0	0	0	2	0	1	1	3	0	0	0	0	0	0	0	0	0	0	0	0	0	0	0	8
OTU681	0	0	1	0	0	0	0	0	0	0	0	3	0	1	0	0	0	0	0	0	0	0	0	0	0	0	0	3	0	0	8
OTU682	0	0	0	1	0	1	0	0	0	0	0	0	0	0	0	0	0	0	1	0	0	0	0	1	0	0	4	0	0	0	8
OTU683	0	0	0	0	0	0	0	0	0	0	0	0	0	1	1	3	0	0	1	0	0	2	0	0	0	0	0	0	0	0	8
OTU684	1	7	0	0	0	0	0	0	0	0	0	0	0	0	0	0	0	0	0	0	0	0	0	0	0	0	0	0	0	0	8
OTU685	0	0	6	0	0	0	0	0	0	0	0	0	0	0	0	0	0	0	0	0	0	2	0	0	0	0	0	0	0	0	8
OTU686	0	0	0	0	0	0	0	0	0	0	0	3	0	0	0	0	0	0	0	0	1	0	2	0	0	1	0	1	0	0	8
OTU687	2	0	0	0	0	0	0	0	0	0	0	0	0	0	0	0	0	0	0	0	0	3	0	2	0	0	0	0	0	0	7
OTU688	0	0	0	0	0	1	0	1	0	0	0	0	0	1	0	0	0	0	0	0	0	0	0	0	0	0	0	4	0	0	7
OTU689	0	0	0	0	0	0	0	5	0	0	0	0	0	0	0	0	0	0	0	0	0	0	0	0	2	0	0	0	0	0	7
OTU690	0	0	0	0	0	1	0	0	0	4	0	0	0	0	1	1	0	0	0	0	0	0	0	0	0	0	0	0	0	0	7
OTU691	1	1	2	0	0	0	0	0	0	0	0	0	0	0	0	1	0	0	0	1	0	0	0	0	1	0	0	0	0	0	7

OTU692	0	1	0	0	0	0	0	0	0	0	0	0	0	0	0	0	0	0	0	1	0	0	0	0	0	1	3	0	1	0	7
OTU693	0	0	1	0	0	0	0	0	1	0	0	0	0	1	2	0	0	0	0	0	0	0	0	0	0	0	0	0	1	1	7
OTU694	0	0	0	0	0	0	0	0	0	0	0	0	0	0	0	0	0	0	2	0	0	0	2	3	0	0	0	0	0	0	7
OTU695	1	0	0	0	0	1	0	1	1	0	0	0	0	0	1	0	1	0	0	1	0	0	0	0	0	0	0	0	0	0	7
OTU696	1	0	0	0	0	1	0	0	0	0	0	1	1	0	0	0	0	0	0	0	0	0	0	0	3	0	0	0	0	0	7
OTU697	0	0	0	0	0	0	0	0	3	0	0	1	0	0	0	1	0	0	0	0	0	1	0	0	0	0	0	0	0	1	7
OTU698	1	0	0	0	0	0	0	4	0	1	0	0	0	0	0	0	0	0	0	0	1	0	0	0	0	0	0	0	0	0	7
OTU699	1	0	0	0	0	0	0	1	0	0	0	0	0	0	0	0	0	1	1	0	0	0	0	0	1	0	0	0	0	2	7
OTU700	0	0	0	1	0	0	0	0	0	0	0	0	0	0	0	0	4	0	2	0	0	0	0	0	0	0	0	0	0	0	7
OTU701	2	0	0	0	0	1	0	0	1	0	0	0	0	0	0	1	1	0	0	0	0	0	0	0	0	0	1	0	0	0	7
OTU702	0	0	0	0	0	0	1	0	5	0	0	0	0	0	0	0	0	0	0	0	0	0	0	0	0	0	0	0	0	1	7
OTU703	0	0	0	0	0	0	0	0	0	0	0	0	0	0	0	0	0	0	0	0	0	3	0	0	0	0	4	0	0	0	7
OTU704	3	0	1	0	0	0	0	0	1	1	0	0	0	0	0	0	0	0	0	0	0	1	0	0	0	0	0	0	0	0	7
OTU705	0	0	0	0	0	0	1	0	0	0	0	0	0	0	0	2	0	0	1	0	0	0	0	0	0	0	2	1	0	0	7
OTU706	0	0	0	0	0	0	0	0	0	0	2	1	0	0	1	0	1	0	0	2	0	0	0	0	0	0	0	0	0	0	7
OTU707	2	0	0	0	0	0	0	0	2	1	1	0	0	0	0	1	0	0	0	0	0	0	0	0	0	0	0	0	0	0	7
OTU708	0	0	2	0	0	0	0	0	0	0	0	0	0	0	0	0	1	0	0	4	0	0	0	0	0	0	0	0	0	0	7
OTU709	0	0	1	0	0	0	1	0	0	0	0	0	0	0	0	0	4	0	1	0	0	0	0	0	0	0	0	0	0	0	7
OTU710	0	0	0	0	0	0	0	0	0	0	0	1	1	0	0	0	1	0	1	1	0	0	0	0	0	1	0	0	1	0	7
OTU711	0	0	0	0	0	0	0	0	0	0	0	0	0	0	0	0	0	0	0	0	0	0	0	0	0	0	0	0	7	0	7
OTU712	1	0	4	0	2	0	0	0	0	0	0	0	0	0	0	0	0	0	0	0	0	0	0	0	0	0	0	0	0	0	7
OTU713	0	0	1	0	0	0	0	1	0	1	1	0	0	1	1	0	0	0	0	0	0	0	0	0	0	0	0	1	0	0	7
OTU714	0	0	0	0	0	0	0	0	0	1	0	0	0	0	0	0	0	0	0	0	1	0	0	0	0	0	0	0	2	3	7
OTU715	0	0	0	0	0	2	0	0	0	0	0	0	0	0	0	0	0	0	0	0	0	0	0	0	0	3	0	0	2	0	7
OTU716	1	0	0	0	0	1	0	0	0	0	0	1	0	0	1	0	0	0	1	0	0	0	0	0	1	0	1	0	0	0	7
OTU717	0	0	0	0	0	0	0	0	0	0	0	0	0	0	0	0	0	0	1	0	3	0	0	0	0	0	3	0	0	0	7

OTU718	0	0	0	0	0	0	3	0	0	1	0	0	0	0	0	0	1	0	0	2	0	0	0	0	0	0	0	0	0	0	7
OTU719	1	0	0	0	0	2	0	0	0	1	0	0	0	1	0	0	0	0	0	2	0	0	0	0	0	0	0	0	0	0	7
OTU720	0	0	0	0	1	0	0	0	0	0	0	0	0	1	0	0	0	0	0	0	0	3	0	0	0	0	1	1	0	0	7
OTU721	0	0	0	0	0	0	0	0	0	0	0	0	0	3	0	0	0	0	0	0	0	0	0	0	0	0	1	0	0	3	7
OTU722	0	1	0	2	0	1	0	0	0	0	0	0	0	1	1	0	0	0	0	0	0	0	0	0	0	0	0	1	0	0	7
OTU723	1	1	0	0	1	0	0	0	1	0	0	0	0	0	0	0	0	0	1	0	1	0	0	0	0	0	1	0	0	0	7
OTU724	1	0	0	0	0	0	0	0	0	0	0	0	0	0	0	0	0	0	0	0	0	0	0	0	0	0	0	1	0	5	7
OTU725	0	0	0	0	0	0	0	0	0	0	0	0	0	0	0	0	0	0	0	0	0	0	0	0	0	0	0	0	7	0	7
OTU726	0	0	0	0	0	0	2	1	0	0	0	0	0	0	1	1	0	0	1	0	0	0	0	0	1	0	0	0	0	0	7
OTU727	0	0	0	0	0	0	0	0	0	0	0	0	0	0	0	0	0	0	0	0	0	0	6	0	0	0	0	0	0	0	6
OTU728	0	0	0	0	0	0	0	2	1	0	0	0	0	2	0	0	0	1	0	0	0	0	0	0	0	0	0	0	0	0	6
OTU729	0	4	0	0	0	0	0	0	0	0	0	0	0	0	2	0	0	0	0	0	0	0	0	0	0	0	0	0	0	0	6
OTU730	0	0	0	0	4	0	0	0	0	0	0	0	0	0	0	1	0	0	0	1	0	0	0	0	0	0	0	0	0	0	6
OTU731	5	0	0	0	0	0	0	0	0	0	0	0	0	0	0	0	0	0	0	0	0	0	0	0	1	0	0	0	0	0	6
OTU732	0	1	0	0	0	0	0	0	0	0	1	0	1	0	0	1	0	0	0	0	0	0	0	0	0	2	0	0	0	0	6
OTU733	0	0	0	0	0	0	0	0	0	0	0	0	0	0	0	0	0	0	0	3	0	0	0	0	3	0	0	0	0	0	6
OTU734	0	0	0	0	0	0	0	0	0	0	0	0	0	3	0	0	0	0	1	0	0	1	0	1	0	0	0	0	0	0	6
OTU735	0	0	0	0	1	0	0	0	1	0	0	0	1	0	0	0	0	0	1	0	0	0	0	0	0	0	0	0	2	0	6
OTU736	0	0	0	0	0	0	0	0	0	0	0	5	0	0	0	0	0	0	0	0	1	0	0	0	0	0	0	0	0	0	6
OTU737	1	1	0	1	0	0	1	0	0	0	0	0	0	0	0	0	0	1	1	0	0	0	0	0	0	0	0	0	0	0	6
OTU738	0	0	0	0	1	0	0	0	0	0	0	0	0	0	1	0	1	0	0	1	0	0	0	0	1	0	0	1	0	0	6
OTU739	0	1	0	0	0	0	0	0	0	0	0	0	0	4	0	0	0	0	0	0	0	0	0	0	1	0	0	0	0	0	6
OTU740	0	0	0	0	0	0	0	0	0	0	0	1	0	0	0	0	0	0	0	0	0	0	0	0	0	0	0	0	0	5	6
OTU741	0	0	1	3	0	0	0	0	0	0	0	0	0	0	0	0	0	0	2	0	0	0	0	0	0	0	0	0	0	0	6
OTU742	0	1	0	0	0	0	1	0	0	0	0	0	0	0	1	0	0	0	0	0	0	1	0	1	0	0	0	0	1	0	6
OTU743	1	0	0	0	1	0	0	0	0	0	0	0	0	1	0	0	1	0	0	0	1	0	0	0	0	0	0	0	0	1	6

OTU744	0	0	0	0	0	0	0	0	0	0	0	1	0	0	0	0	0	0	2	0	0	1	0	0	0	1	0	0	0	1	6
OTU745	0	0	0	0	0	0	0	0	0	0	0	0	0	1	0	0	0	0	0	0	0	4	1	0	0	0	0	0	0	0	6
OTU746	0	0	0	0	0	0	0	0	0	3	0	1	0	0	0	0	0	0	0	0	0	0	0	1	0	0	1	0	0	0	6
OTU747	0	0	0	0	0	0	0	0	0	0	0	0	0	6	0	0	0	0	0	0	0	0	0	0	0	0	0	0	0	0	6
OTU748	0	0	0	0	1	0	0	0	0	0	0	0	1	0	1	0	0	0	0	0	0	0	0	1	0	1	0	0	0	1	6
OTU749	0	0	0	0	0	0	0	0	0	0	0	4	0	0	0	0	0	0	0	0	0	0	2	0	0	0	0	0	0	0	6
OTU750	0	0	0	0	0	0	0	0	0	0	0	0	0	0	0	0	0	0	0	0	2	0	0	0	0	0	1	3	0	0	6
OTU751	0	0	0	0	0	0	0	0	0	1	1	0	1	0	1	2	0	0	0	0	0	0	0	0	0	0	0	0	0	0	6
OTU752	0	0	1	0	0	1	0	0	2	0	0	0	0	0	0	0	0	0	0	0	0	0	0	0	1	0	0	0	1	0	6
OTU753	0	0	0	0	0	0	0	0	0	0	0	0	0	0	0	0	0	0	0	5	0	0	0	0	0	0	0	1	0	0	6
OTU754	0	1	0	0	1	0	0	0	0	0	2	0	0	0	0	0	0	0	0	1	0	0	0	0	0	1	0	0	0	0	6
OTU755	0	0	0	1	0	0	0	0	0	0	0	0	0	0	0	0	1	0	0	1	0	2	0	0	0	0	0	0	0	1	6
OTU756	0	0	0	1	0	0	1	0	0	0	0	0	0	0	0	0	1	0	0	0	1	0	0	0	0	0	0	1	1	0	6
OTU757	0	0	0	0	0	0	0	0	0	0	0	0	0	0	0	0	0	1	0	0	0	0	2	2	0	1	0	0	0	0	6
OTU758	0	0	0	0	0	0	0	0	0	0	0	0	0	0	0	0	0	0	0	0	0	0	0	0	0	0	0	6	0	0	6
OTU759	0	0	0	1	0	0	1	1	0	0	0	0	1	0	0	1	0	0	1	0	0	0	0	0	0	0	0	0	0	0	6
OTU760	0	0	0	0	0	0	0	0	0	0	0	0	0	0	0	0	2	4	0	0	0	0	0	0	0	0	0	0	0	0	6
OTU761	0	0	0	0	0	1	0	0	0	0	1	0	0	0	0	0	0	0	0	0	0	2	1	0	1	0	0	0	0	0	6
OTU762	0	0	0	0	0	0	0	0	1	0	0	0	0	0	0	0	0	0	0	0	0	0	0	0	0	4	0	1	0	0	6
OTU763	0	0	1	0	0	2	1	1	0	0	0	0	0	0	0	0	0	0	0	0	0	0	0	0	0	0	0	0	0	0	5
OTU764	0	0	0	0	0	0	1	0	0	0	0	0	0	0	4	0	0	0	0	0	0	0	0	0	0	0	0	0	0	0	5
OTU765	0	0	0	0	0	0	0	0	0	0	0	0	0	0	0	0	0	0	0	0	0	0	0	0	0	0	0	0	0	5	5
OTU766	0	0	0	0	0	0	0	0	0	0	0	0	0	5	0	0	0	0	0	0	0	0	0	0	0	0	0	0	0	0	5
OTU767	0	0	0	0	1	0	0	1	0	0	0	0	0	0	0	0	0	0	0	0	0	0	0	0	0	1	0	0	0	2	5
OTU768	0	0	0	0	0	0	0	0	3	0	0	0	0	0	0	0	0	0	0	0	1	0	0	0	0	0	0	0	0	1	5
OTU769	0	1	0	0	2	0	0	0	0	0	0	0	0	0	1	0	0	0	0	0	1	0	0	0	0	0	0	0	0	0	5

OTU770	0	0	0	0	0	0	0	1	0	0	0	1	0	1	0	0	0	0	0	0	0	0	0	0	1	0	0	0	0	1	5
OTU771	0	0	0	0	0	0	0	0	0	0	0	0	0	0	0	0	0	0	0	5	0	0	0	0	0	0	0	0	0	0	5
OTU772	0	0	2	0	0	0	0	0	0	0	0	0	0	0	0	0	0	1	2	0	0	0	0	0	0	0	0	0	0	0	5
OTU773	1	1	0	0	0	0	0	0	0	0	0	0	0	1	0	0	0	0	0	0	0	0	0	0	1	0	0	1	0	0	5
OTU774	0	0	0	0	0	0	0	0	0	0	0	0	0	0	0	2	0	0	0	0	0	0	1	0	0	0	0	0	1	1	5
OTU775	0	5	0	0	0	0	0	0	0	0	0	0	0	0	0	0	0	0	0	0	0	0	0	0	0	0	0	0	0	0	5
OTU776	1	0	0	0	0	0	0	0	0	0	0	0	1	0	0	0	0	0	2	0	0	0	0	0	0	0	1	0	0	0	5
OTU777	0	1	0	0	0	0	0	0	0	0	0	1	0	0	0	0	0	1	0	1	0	0	0	0	0	0	0	0	0	1	5
OTU778	0	1	0	0	0	0	0	0	1	0	0	0	2	0	0	0	0	0	0	0	0	0	0	0	0	1	0	0	0	0	5
OTU779	0	0	0	0	0	0	1	0	0	0	0	0	0	0	0	0	0	0	0	1	0	1	0	1	0	0	0	0	0	1	5
OTU780	0	0	0	0	0	0	0	0	0	0	0	0	0	0	1	0	0	0	1	0	0	0	0	0	0	0	0	0	2	1	5
OTU781	0	0	0	0	0	1	0	0	0	0	0	4	0	0	0	0	0	0	0	0	0	0	0	0	0	0	0	0	0	0	5
OTU782	0	0	0	0	0	0	0	0	0	0	0	1	1	0	1	0	0	0	0	0	1	0	0	0	0	0	0	0	1	0	5
OTU783	1	0	1	0	0	0	0	0	3	0	0	0	0	0	0	0	0	0	0	0	0	0	0	0	0	0	0	0	0	0	5
OTU784	0	0	0	0	0	1	0	0	0	0	1	0	0	0	1	0	0	0	0	1	0	0	1	0	0	0	0	0	0	0	5
OTU785	0	0	0	0	1	0	0	0	1	0	0	1	1	1	0	0	0	0	0	0	0	0	0	0	0	0	0	0	0	0	5
OTU786	1	0	0	0	0	0	0	0	0	0	0	0	0	0	0	0	0	0	1	0	0	0	0	0	3	0	0	0	0	0	5
OTU787	0	0	0	0	0	0	0	2	0	0	0	0	0	0	1	0	0	0	0	0	1	0	0	0	0	0	0	0	0	1	5
OTU788	0	0	0	0	0	0	0	0	0	0	0	0	0	1	0	0	0	0	0	0	0	1	0	0	1	0	0	0	2	0	5
OTU789	2	0	0	0	0	0	0	0	0	1	0	0	0	0	0	0	0	0	0	0	0	0	0	1	0	0	0	0	0	1	5
OTU790	0	0	1	0	0	0	0	0	0	0	0	0	0	0	0	0	0	0	0	1	2	0	0	0	0	0	1	0	0	0	5
OTU791	0	0	0	0	0	0	0	0	0	3	0	0	0	0	0	0	0	0	0	0	0	0	0	0	0	0	2	0	0	0	5
OTU792	3	0	0	0	0	0	0	0	0	0	0	0	0	0	0	0	0	0	0	0	0	0	1	0	0	0	0	0	0	1	5
OTU793	0	0	1	0	0	0	0	0	0	0	0	1	0	0	0	0	1	0	0	0	0	0	1	0	0	1	0	0	0	0	5
OTU794	0	0	0	0	0	0	0	0	0	0	0	0	0	0	5	0	0	0	0	0	0	0	0	0	0	0	0	0	0	0	5
OTU795	5	0	0	0	0	0	0	0	0	0	0	0	0	0	0	0	0	0	0	0	0	0	0	0	0	0	0	0	0	0	5

OTU796	0	0	1	0	1	0	1	0	0	0	0	0	0	0	0	0	0	0	0	0	2	0	0	0	0	0	0	0	0	0	5
OTU797	0	0	4	0	0	0	0	0	0	0	0	0	0	0	0	0	1	0	0	0	0	0	0	0	0	0	0	0	0	0	5
OTU798	0	1	0	0	1	0	1	0	0	0	0	0	0	0	0	1	0	0	0	0	1	0	0	0	0	0	0	0	0	0	5
OTU799	0	0	0	2	0	0	0	1	1	1	0	0	0	0	0	0	0	0	0	0	0	0	0	0	0	0	0	0	0	0	5
OTU800	0	0	0	0	0	0	0	0	0	0	0	0	0	0	0	0	0	0	0	0	0	5	0	0	0	0	0	0	0	0	5
OTU801	0	0	0	0	0	0	0	0	0	1	1	1	0	0	0	0	0	0	1	0	0	0	1	0	0	0	0	0	0	0	5
OTU802	0	0	0	0	0	1	0	0	0	0	0	0	0	0	0	1	0	0	0	1	0	0	0	0	0	0	0	0	1	1	5
OTU803	0	0	0	0	0	0	0	0	0	0	0	1	0	0	0	0	0	0	0	0	0	0	0	0	0	4	0	0	0	0	5
OTU804	0	0	0	0	0	0	0	0	0	0	0	0	0	0	0	0	0	0	4	0	0	0	0	0	0	0	0	0	1	0	5
OTU805	0	0	0	0	0	0	0	0	0	0	0	0	0	0	0	0	0	0	0	3	0	0	0	0	0	2	0	0	0	0	5
OTU806	0	0	0	0	0	0	0	0	0	1	0	0	0	0	0	0	0	1	1	0	0	0	0	1	0	1	0	0	0	0	5
OTU807	0	0	1	1	1	0	0	0	0	0	0	0	0	0	1	0	0	0	0	0	0	0	0	0	0	1	0	0	0	0	5
OTU808	0	0	0	0	2	0	0	0	0	0	0	0	0	0	0	1	0	1	0	0	0	0	0	1	0	0	0	0	0	0	5
OTU809	0	0	0	0	0	0	0	0	0	2	0	0	0	1	0	0	0	2	0	0	0	0	0	0	0	0	0	0	0	0	5
OTU810	0	0	0	0	0	1	0	1	0	0	0	0	0	0	0	0	0	0	0	0	0	0	1	2	0	0	0	0	0	0	5
OTU811	0	0	0	2	0	0	0	0	0	0	0	0	0	0	0	0	0	0	0	0	0	0	0	0	1	2	0	0	0	0	5
OTU812	0	0	0	0	0	0	0	0	0	0	0	0	0	0	0	0	0	0	0	0	0	0	0	2	0	0	0	1	0	2	5
OTU813	0	0	0	0	0	0	0	0	0	0	0	0	3	0	0	0	0	0	0	0	0	1	0	0	0	0	1	0	0	0	5
OTU814	0	0	0	2	3	0	0	0	0	0	0	0	0	0	0	0	0	0	0	0	0	0	0	0	0	0	0	0	0	0	5
OTU815	0	0	0	0	0	1	1	0	0	0	0	2	0	1	0	0	0	0	0	0	0	0	0	0	0	0	0	0	0	0	5
OTU816	0	0	0	1	0	0	0	0	0	2	0	0	0	0	0	0	1	0	0	0	0	0	0	0	1	0	0	0	0	0	5
OTU817	0	0	0	0	0	0	80	5	12	762	943	0	193	0	36	12	0	3	0	0	0	0	0	0	0	0	0	0	0	0	2046
OTU818	35	35	24	45	29	51	19	36	38	23	9	17	7	14	7	11	10	3	15	5	18	2	6	0	1	14	32	33	36	66	641
OTU819	0	3	11	0	1	0	1	0	10	1	172	38	15	3	0	0	0	16	1	3	0	7	5	29	16	1	10	2	144	103	592
OTU820	0	0	0	0	0	0	1	0	0	0	0	1	2	17	0	0	0	2	51	1	57	11	5	1	10	9	4	4	56	267	499
OTU821	4	12	0	14	4	2	0	0	0	0	0	0	0	0	0	0	0	0	0	0	8	14	344	6	4	29	4	22	18	4	489

OTU822	0	0	0	0	0	0	0	2	1	0	2	0	0	0	0	0	1	373	3	2	2	0	0	0	1	0	0	0	0	0	387
OTU823	15	17	10	6	4	9	2	1	1	0	0	0	7	0	0	0	0	1	0	0	5	18	6	0	183	5	12	2	0	0	304
OTU824	0	0	0	0	0	0	3	1	0	14	1	1	1	0	1	0	0	240	1	0	0	0	1	7	1	0	0	0	0	0	272
OTU825	0	0	0	0	0	0	0	0	0	0	0	0	0	6	0	0	0	0	38	1	0	0	1	217	0	1	0	0	0	0	264
OTU826	0	0	0	0	0	0	10	2	0	105	13	0	9	0	0	4	0	101	0	0	0	0	0	12	0	1	0	0	0	0	257
OTU827	0	0	0	0	0	0	0	0	0	0	0	0	0	35	0	0	0	0	15	55	0	0	6	0	144	0	0	0	0	0	255
OTU828	0	3	0	0	0	3	0	0	0	0	0	0	0	1	0	0	0	6	6	0	6	181	25	0	3	4	5	1	5	0	249
OTU829	0	0	0	0	0	0	0	0	0	0	0	0	0	0	0	0	0	0	0	0	0	0	0	227	0	8	0	0	1	8	244
OTU830	0	0	0	0	0	0	0	0	0	0	0	0	0	0	0	1	0	240	0	0	0	0	0	0	0	0	0	0	0	0	241
OTU831	0	0	0	0	0	0	0	0	0	0	0	0	0	1	0	0	0	81	18	0	3	3	1	57	8	3	0	1	8	57	241
OTU832	4	1	195	1	29	0	2	0	0	0	0	0	0	0	0	0	0	0	0	0	0	0	0	0	0	0	0	0	0	0	232
OTU833	0	0	4	0	0	0	1	0	4	0	84	9	3	2	0	0	0	12	1	1	0	4	0	8	5	1	4	3	43	22	211
OTU834	12	10	5	2	6	5	12	10	5	23	3	17	7	11	5	4	7	10	8	16	4	8	6	1	2	1	2	0	1	4	207
OTU835	0	0	0	0	0	0	0	0	0	0	0	0	0	23	0	0	3	0	7	20	0	0	22	0	124	0	0	0	0	0	199
OTU836	6	6	2	0	6	3	5	1	1	0	0	3	2	11	0	0	1	8	0	19	8	14	19	20	4	10	26	6	6	12	199
OTU837	1	103	2	0	0	2	8	17	0	3	5	0	48	0	1	0	0	0	0	0	2	0	0	5	0	0	1	0	0	0	198
OTU838	2	1	127	1	13	0	0	0	0	6	15	0	0	0	0	0	0	0	0	0	0	0	6	0	0	0	0	0	0	0	171
OTU839	5	4	2	4	2	4	11	7	7	7	5	7	7	8	10	4	4	1	3	8	4	6	9	1	2	3	2	6	2	15	160
OTU840	0	0	0	0	0	0	0	0	0	0	0	0	0	3	0	0	0	1	0	0	0	15	1	92	1	32	0	0	0	7	152
OTU841	0	0	0	0	0	0	5	0	106	0	0	6	8	0	16	4	0	2	0	0	0	0	0	0	2	0	0	0	0	0	149
OTU842	14	2	11	0	4	1	0	9	0	8	8	17	1	3	0	0	7	6	10	10	8	0	0	3	11	1	10	0	1	4	149
OTU843	0	76	0	1	0	1	8	8	0	2	2	0	35	0	0	0	0	0	0	0	3	0	1	1	1	0	0	0	0	0	139
OTU844	0	0	0	0	0	0	5	6	2	4	8	6	62	1	13	3	0	15	4	0	0	1	0	0	2	0	0	0	0	0	132
OTU845	0	0	2	0	0	0	3	0	5	3	2	13	9	0	0	1	0	88	1	0	0	0	0	0	2	0	0	0	0	0	129
OTU846	0	0	0	0	0	0	0	0	0	0	0	0	0	0	0	0	0	126	1	0	0	0	0	0	0	0	1	0	0	0	128
OTU847	0	0	0	0	0	0	35	2	1	48	12	0	21	0	1	0	0	3	0	0	0	0	0	0	0	0	0	0	0	0	123

OTU848	0	0	0	0	0	0	0	0	0	4	1	0	0	0	2	0	0	0	20	0	2	0	18	67	0	0	1	0	0	1	116
OTU849	0	0	0	0	0	0	0	0	0	29	10	0	2	0	0	0	0	68	0	0	0	0	2	4	0	0	0	0	0	0	115
OTU850	0	0	0	0	0	0	0	0	0	2	0	4	0	0	0	18	0	87	0	0	0	0	0	0	0	0	0	0	0	0	111
OTU851	0	3	0	21	13	16	1	2	0	0	0	2	0	8	0	0	2	0	4	0	8	0	15	3	1	4	6	0	2	0	111
OTU852	0	54	1	0	0	1	5	8	0	5	4	0	25	0	0	0	0	0	2	0	0	0	0	1	0	0	0	0	0	0	106
OTU853	0	0	0	0	0	0	1	0	2	9	14	0	18	8	0	0	0	14	3	18	0	4	7	3	2	1	0	0	2	0	106
OTU854	2	4	0	2	3	1	1	2	6	3	0	0	2	3	7	5	5	1	3	5	6	8	3	1	3	1	7	4	7	5	100
OTU855	1	2	2	3	1	1	2	6	4	0	1	16	1	2	3	3	1	1	1	3	4	4	5	1	2	1	1	4	2	17	95
OTU856	60	2	0	15	6	5	0	1	0	0	0	0	0	0	0	0	0	0	0	0	0	0	0	1	0	0	0	0	0	1	91
OTU857	0	0	0	0	0	0	0	0	0	0	0	0	0	2	0	0	0	4	11	0	0	3	3	51	1	1	0	0	0	12	88
OTU858	0	0	0	0	0	0	7	0	0	57	0	0	17	0	3	0	0	0	0	0	0	0	0	0	0	0	0	0	0	0	84
OTU859	0	0	0	0	0	0	0	0	66	3	8	4	0	0	0	0	0	0	0	0	0	2	0	0	0	0	0	0	0	0	83
OTU860	0	0	0	0	0	0	10	3	5	9	3	8	17	0	4	2	1	13	7	0	0	0	0	0	1	0	0	0	0	0	83
OTU861	0	0	1	1	0	1	0	1	0	0	3	2	0	1	4	5	9	6	8	8	9	4	6	0	2	2	0	5	0	2	80
OTU862	0	0	0	0	0	0	0	0	0	12	12	0	8	0	11	1	0	11	0	0	0	2	0	14	7	0	0	0	0	0	78
OTU863	0	0	0	0	0	0	0	0	0	0	0	0	0	24	0	0	0	0	2	0	5	3	3	13	1	4	3	0	6	14	78
OTU864	6	7	3	1	2	9	2	5	9	2	4	6	1	7	9	1	0	0	0	0	0	1	1	0	0	0	0	0	1	0	77
OTU865	0	0	0	0	0	0	0	0	0	0	0	0	0	0	0	2	0	74	0	0	0	0	0	0	0	0	0	0	0	0	76
OTU866	0	0	0	0	0	0	0	0	0	0	0	1	0	4	0	0	0	0	8	0	8	0	0	0	0	1	0	0	2	51	75
OTU867	0	0	0	0	0	0	0	0	0	0	0	0	0	20	0	0	0	0	0	11	0	0	5	0	37	0	0	0	0	0	73
OTU868	0	0	0	0	0	0	0	0	2	2	6	1	10	1	1	0	0	7	2	10	0	7	13	1	1	1	4	0	3	0	72
OTU869	0	0	0	0	0	0	0	0	3	3	18	1	9	0	7	0	0	4	0	0	0	0	6	14	2	0	1	0	0	0	68
OTU870	0	0	0	0	0	0	0	0	0	0	0	0	0	0	0	0	0	2	5	1	0	1	1	49	1	0	0	0	0	8	68
OTU871	1	0	0	0	4	0	0	0	0	0	0	3	1	2	0	2	0	0	1	0	1	0	2	45	0	1	0	0	0	5	68
OTU872	30	1	6	4	0	1	1	1	0	7	0	1	5	0	0	0	0	0	2	2	1	1	1	1	0	0	0	0	0	2	67
OTU873	0	0	0	0	0	0	0	0	0	0	0	0	1	2	0	0	0	1	12	0	7	0	2	2	3	0	0	0	1	36	67

OTU874	0	0	0	0	0	0	0	0	0	5	1	25	0	0	0	11	0	22	0	0	0	0	0	0	0	0	0	0	0	0	64
OTU875	3	0	0	0	0	0	4	5	3	4	0	3	2	1	9	1	0	4	2	2	0	0	9	6	0	5	0	0	1	0	64
OTU876	13	0	25	0	5	3	0	0	0	0	2	0	3	8	0	0	0	0	0	0	0	0	0	0	0	1	0	0	0	0	60
OTU877	0	0	0	0	0	0	0	0	0	0	0	0	0	2	0	0	0	0	0	0	1	0	2	28	0	0	0	1	1	24	59
OTU878	0	0	0	0	0	0	0	0	0	0	0	0	0	0	0	0	0	0	17	1	16	1	3	2	0	1	0	0	8	10	59
OTU879	0	0	0	0	0	0	0	0	0	0	0	0	0	0	0	0	0	22	6	0	0	0	0	18	1	1	0	0	2	8	58
OTU880	0	0	0	0	0	0	0	0	0	0	0	0	0	0	0	0	0	0	0	0	1	9	7	1	0	0	32	1	5	2	58
OTU881	0	0	0	0	0	12	0	42	0	1	0	0	0	0	0	0	0	0	0	0	0	0	0	0	0	0	0	0	0	0	55
OTU882	8	1	4	0	1	0	0	0	0	3	2	4	0	3	1	1	5	1	1	6	0	0	0	0	0	8	1	1	1	3	55
OTU883	0	0	0	0	0	0	0	0	0	0	0	0	0	9	0	0	0	0	0	9	0	0	2	0	34	0	0	0	0	0	54
OTU884	0	0	0	0	0	0	0	0	0	0	0	17	8	6	0	0	0	0	0	0	0	9	4	0	0	0	1	1	2	5	53
OTU885	0	1	5	0	0	0	0	0	0	0	0	0	0	0	0	3	12	0	2	0	0	3	0	0	1	0	4	3	10	8	52
OTU886	0	0	0	0	0	0	5	3	0	16	9	0	9	0	1	0	0	7	0	0	0	0	0	0	0	0	0	0	0	0	50
OTU887	0	0	0	0	0	0	0	0	0	0	0	0	0	2	0	0	0	0	1	0	1	0	8	28	1	0	0	1	0	8	50
OTU888	1	1	0	1	13	0	1	0	0	0	0	1	0	0	0	0	0	0	0	0	2	0	0	0	0	0	0	4	2	23	49
OTU889	2	6	1	1	3	2	0	0	0	0	1	2	0	2	0	0	0	3	0	1	0	9	7	2	1	2	0	0	0	1	46
OTU890	1	3	3	2	0	0	0	0	1	3	0	0	0	0	10	0	2	0	7	0	1	4	0	1	0	1	4	1	0	2	46
OTU891	0	0	0	8	0	0	0	0	0	0	0	0	0	0	0	0	20	0	0	0	0	0	0	0	4	0	0	0	2	11	45
OTU892	0	0	0	0	0	0	0	0	0	2	0	0	0	2	0	0	0	2	4	0	0	0	3	10	0	2	1	0	5	14	45
OTU893	37	0	0	3	0	0	0	0	0	0	0	0	0	0	0	0	0	0	0	0	0	0	0	0	1	0	0	0	0	0	41
OTU894	0	0	0	0	0	0	0	0	0	0	0	0	0	0	0	0	0	0	0	0	0	0	8	31	0	0	0	0	2	0	41
OTU895	0	0	2	0	0	3	0	4	0	0	0	3	0	0	0	0	0	6	3	0	0	0	0	0	19	0	1	0	0	0	41
OTU896	0	0	0	1	1	0	0	0	2	2	0	4	0	0	0	0	0	2	0	0	4	3	3	11	0	0	0	0	0	8	41
OTU897	0	0	0	0	0	0	0	0	0	0	0	0	0	0	0	0	0	0	0	0	0	0	0	40	0	0	0	0	0	0	40
OTU898	2	2	1	0	0	0	0	4	1	1	1	0	3	0	0	1	2	1	4	1	1	0	1	0	1	1	5	1	1	4	39
OTU899	5	0	0	0	0	0	0	0	0	20	5	2	3	0	0	1	0	3	0	0	0	0	0	0	0	0	0	0	0	0	39

OTU900	0	1	0	0	5	3	0	0	2	0	1	2	0	6	1	0	0	4	0	1	0	1	6	0	0	1	0	3	1	1	39
OTU901	0	0	0	0	13	8	1	0	0	6	10	0	0	0	0	0	0	0	0	0	0	0	0	0	0	0	0	0	0	0	38
OTU902	0	0	0	0	0	0	0	0	0	0	0	0	0	0	0	0	0	0	0	0	0	0	0	32	0	2	0	0	0	4	38
OTU903	0	0	0	0	0	0	1	0	0	2	3	0	8	10	0	0	0	0	0	0	0	0	0	11	3	0	0	0	0	0	38
OTU904	35	0	0	0	0	0	0	0	0	0	0	0	0	0	0	0	0	0	0	0	0	2	0	1	0	0	0	0	0	0	38
OTU905	0	0	0	0	11	0	1	0	0	0	0	24	0	0	0	0	0	0	0	0	0	0	0	0	0	0	0	0	0	0	36
OTU906	0	0	0	0	0	0	0	0	0	0	0	0	0	0	1	0	0	35	0	0	0	0	0	0	0	0	0	0	0	0	36
OTU907	0	4	1	0	0	0	5	0	3	0	1	0	0	0	4	2	0	0	0	5	6	0	0	0	0	0	0	0	0	5	36
OTU908	16	1	0	1	0	0	0	0	1	3	1	1	3	0	0	0	0	0	1	0	0	0	4	2	0	0	0	0	0	1	35
OTU909	0	0	0	0	0	0	0	0	0	0	0	0	1	1	0	0	0	1	4	0	0	0	4	19	0	2	0	0	0	3	35
OTU910	0	0	0	0	0	0	0	0	0	0	0	0	0	0	0	0	7	0	4	0	0	0	0	6	18	0	0	0	0	0	35
OTU911	0	4	1	2	1	0	7	0	1	5	0	0	1	0	0	0	0	1	0	0	3	4	2	0	0	0	3	0	0	0	35
OTU912	0	0	0	0	0	0	0	1	0	0	0	3	3	6	0	0	0	14	8	0	0	0	0	0	0	0	0	0	0	0	35
OTU913	3	0	0	0	0	0	2	12	0	7	1	1	6	0	0	0	0	1	0	0	0	1	0	0	1	0	0	0	0	0	35
OTU914	2	2	2	0	0	3	0	0	0	5	1	0	1	4	0	0	0	0	0	0	2	0	2	0	0	6	1	0	1	3	35
OTU915	6	0	2	4	0	1	0	1	0	2	0	0	0	0	6	0	3	0	1	0	1	2	0	0	0	0	2	2	0	1	34
OTU916	0	0	0	0	0	0	0	0	0	0	0	0	0	0	0	0	0	34	0	0	0	0	0	0	0	0	0	0	0	0	34
OTU917	10	1	0	1	2	1	0	0	3	4	0	2	1	0	2	0	0	0	0	2	0	0	0	0	0	0	1	3	0	1	34
OTU918	0	0	0	0	1	0	0	0	1	0	0	4	0	0	0	0	0	0	1	0	0	0	1	14	1	1	3	0	1	6	34
OTU919	0	0	3	0	6	0	0	0	0	5	1	0	0	0	0	0	0	19	0	0	0	0	0	0	0	0	0	0	0	0	34
OTU920	0	0	0	0	0	0	0	0	0	2	1	0	7	1	0	0	0	18	0	1	0	2	1	1	0	0	0	0	0	0	34
OTU921	0	0	0	0	0	0	1	0	0	0	0	1	0	3	0	0	0	0	0	1	1	0	0	14	5	2	0	0	0	6	34
OTU922	0	12	0	0	0	0	3	3	0	0	2	0	13	0	0	0	0	0	0	0	0	0	0	0	0	0	0	0	0	0	33
OTU923	5	0	0	4	7	4	0	0	0	0	0	0	0	11	0	0	0	0	0	0	0	0	2	0	0	0	0	0	0	0	33
OTU924	2	4	0	5	5	0	0	0	0	0	0	0	0	0	0	0	0	0	0	0	3	0	0	3	0	4	0	2	1	4	33
OTU925	9	1	0	1	4	3	0	1	0	0	0	1	0	0	0	0	0	5	1	0	0	0	1	5	0	0	0	0	0	0	32

OTU926	1	0	1	0	2	0	1	1	0	1	0	2	3	0	1	1	0	0	1	3	1	2	1	0	0	1	1	1	2	5	32
OTU927	0	0	0	0	0	0	0	0	0	0	1	0	0	0	0	1	0	28	0	0	0	0	0	0	2	0	0	0	0	0	32
OTU928	0	0	0	0	0	0	0	1	0	10	1	0	0	0	0	0	0	19	0	0	0	0	0	1	0	0	0	0	0	0	32
OTU929	0	0	0	1	2	2	3	0	1	1	0	0	0	0	2	11	0	1	0	1	0	0	0	0	2	0	1	1	1	2	32
OTU930	17	0	0	6	6	0	0	0	0	0	0	0	0	0	0	0	0	0	0	0	0	0	0	0	0	1	0	0	2	0	32
OTU931	4	0	0	0	1	0	0	0	0	0	0	1	0	4	0	0	1	0	1	3	3	0	3	3	1	1	6	0	0	0	32
OTU932	0	0	0	0	0	0	0	0	0	0	0	0	0	0	0	0	0	0	0	0	0	4	5	0	0	0	19	0	3	1	32
OTU933	0	0	22	0	1	3	0	0	0	1	4	0	0	0	0	0	0	0	0	0	0	0	0	0	0	0	0	0	0	0	31
OTU934	0	0	0	0	0	0	0	0	0	0	0	0	0	0	0	3	0	27	0	1	0	0	0	0	0	0	0	0	0	0	31
OTU935	0	4	0	1	6	0	0	0	0	0	0	1	0	0	0	0	0	0	1	0	0	1	0	7	0	9	0	0	0	1	31
OTU936	1	1	0	0	1	0	0	0	0	0	0	0	0	1	0	0	0	0	0	0	4	0	5	7	0	3	5	0	0	3	31
OTU937	0	0	0	0	1	0	0	0	0	0	0	0	0	0	0	0	0	0	1	1	0	0	13	1	2	1	2	0	2	7	31
OTU938	0	0	0	0	0	0	0	0	0	0	0	0	0	0	0	0	0	0	0	0	0	0	0	13	1	17	0	0	0	0	31
OTU939	0	2	1	2	1	2	0	2	0	0	0	2	1	0	0	2	1	1	2	2	2	0	0	0	0	3	0	1	1	2	30
OTU940	0	0	0	11	0	0	2	0	1	0	0	6	5	0	0	0	0	0	1	0	0	0	0	1	3	0	0	0	0	0	30
OTU941	0	0	0	0	0	1	0	0	0	0	8	3	0	2	0	0	2	1	0	2	0	0	1	6	2	2	0	0	0	0	30
OTU942	0	0	0	0	0	0	0	0	0	0	0	0	0	0	0	0	0	0	0	0	0	0	7	3	0	0	0	0	0	20	30
OTU943	0	0	4	0	0	1	0	0	0	1	0	1	1	0	0	2	0	3	1	0	1	5	2	1	1	2	3	1	0	0	30
OTU944	0	1	0	0	0	0	0	0	0	0	0	0	0	19	0	0	0	1	0	0	0	3	0	1	3	0	0	0	2	0	30
OTU945	11	0	0	0	0	0	0	0	0	6	0	0	0	2	0	0	5	0	0	0	0	2	3	0	0	0	0	0	0	0	29
OTU946	0	0	0	0	2	0	4	5	0	12	0	0	0	0	0	0	0	0	0	0	0	0	0	5	0	1	0	0	0	0	29
OTU947	0	1	0	0	0	0	0	0	0	0	0	2	0	1	0	0	0	0	0	2	2	1	4	3	1	4	7	1	0	0	29
OTU948	0	0	0	0	0	0	0	0	0	0	0	0	0	3	0	0	0	0	0	7	0	0	1	0	18	0	0	0	0	0	29
OTU949	0	0	0	0	0	0	0	0	0	0	0	0	0	0	0	0	0	0	0	2	0	0	17	0	4	0	0	0	4	1	28
OTU950	0	0	0	0	0	0	0	0	0	0	0	0	0	0	0	0	0	2	0	0	0	0	0	26	0	0	0	0	0	0	28
OTU951	0	0	0	0	0	1	4	1	0	0	1	2	0	1	0	0	0	1	0	0	0	2	4	3	4	1	1	0	0	2	28

OTU952	0	0	0	0	0	0	0	0	0	0	0	0	0	0	0	0	0	0	0	0	0	0	1	14	13	0	0	0	0	0	28
OTU953	0	8	7	0	0	0	0	0	4	0	0	0	4	0	0	0	0	0	0	0	0	0	0	0	0	4	0	0	0	0	27
OTU954	0	0	0	0	1	2	4	0	0	15	1	0	1	0	0	0	0	0	0	0	0	0	0	3	0	0	0	0	0	0	27
OTU955	0	0	0	3	0	0	0	0	0	0	0	1	0	1	0	0	0	0	0	1	3	5	2	0	2	1	8	0	0	0	27
OTU956	0	0	0	0	0	0	0	0	0	0	0	0	0	0	0	0	0	0	0	0	0	0	0	5	0	0	0	0	0	22	27
OTU957	22	2	0	0	0	0	0	0	0	0	0	1	0	1	0	0	0	0	0	0	0	0	0	0	0	0	0	0	0	1	27
OTU958	0	0	0	0	0	0	0	0	0	0	1	0	0	0	0	0	0	26	0	0	0	0	0	0	0	0	0	0	0	0	27
OTU959	5	1	1	0	1	4	0	0	0	2	0	0	0	0	0	0	0	0	0	0	1	2	0	7	0	0	1	1	0	0	26
OTU960	0	0	0	5	0	0	0	13	0	0	0	0	0	0	0	0	0	0	2	0	0	0	0	0	5	1	0	0	0	0	26
OTU961	0	2	2	0	0	0	0	0	0	0	0	0	1	0	2	1	0	5	0	0	2	0	0	0	2	1	0	5	0	3	26
OTU962	0	0	0	0	0	0	0	0	0	0	0	0	0	0	0	0	0	2	0	0	0	0	1	23	0	0	0	0	0	0	26
OTU963	0	0	0	0	0	0	0	0	0	0	0	0	0	0	0	0	0	0	0	0	0	0	4	10	9	0	0	0	3	0	26
OTU964	3	4	4	1	0	1	4	1	0	7	0	0	1	0	0	0	0	0	0	0	0	0	0	0	0	0	0	0	0	0	26
OTU965	0	0	0	0	0	0	0	0	0	0	0	0	0	0	0	0	0	26	0	0	0	0	0	0	0	0	0	0	0	0	26
OTU966	0	0	0	0	0	0	0	0	0	0	0	0	0	0	0	0	0	0	12	6	0	1	6	0	0	0	0	0	1	0	26
OTU967	0	0	2	0	0	0	0	0	0	2	2	2	1	0	1	4	1	2	0	1	3	1	1	0	0	0	1	1	0	0	25
OTU968	0	0	4	0	0	0	2	4	0	0	0	0	0	0	0	0	0	2	0	0	0	0	0	2	0	0	0	3	0	8	25
OTU969	0	3	3	1	0	3	6	2	3	4	0	0	0	0	0	0	0	0	0	0	0	0	0	0	0	0	0	0	0	0	25
OTU970	0	0	0	0	0	0	0	0	0	0	0	0	25	0	0	0	0	0	0	0	0	0	0	0	0	0	0	0	0	0	25
OTU971	0	1	0	0	0	0	0	0	0	1	0	1	0	2	0	0	0	2	0	0	3	3	2	2	1	3	4	0	0	0	25
OTU972	0	0	0	0	0	0	0	0	2	0	0	9	0	6	0	0	0	0	1	0	1	0	3	1	0	1	0	0	1	0	25
OTU973	2	0	0	0	12	0	0	0	0	0	2	0	0	2	0	0	0	0	0	0	0	0	0	0	0	0	5	0	1	0	24
OTU974	0	0	0	4	0	0	5	2	0	3	0	0	0	1	0	0	0	0	0	0	0	0	0	0	0	1	2	0	6	0	24
OTU975	0	5	0	0	2	2	0	0	1	13	0	0	0	0	0	0	0	0	0	0	0	0	0	0	0	0	1	0	0	0	24
OTU976	0	0	0	0	0	3	0	0	0	0	0	0	0	1	9	10	0	0	0	0	0	0	0	0	0	0	0	1	0	0	24
OTU977	0	0	2	0	0	0	0	0	0	0	0	0	0	0	0	0	0	0	2	0	0	0	0	2	0	0	0	0	3	15	24

OTU978	0	0	0	0	0	0	0	0	0	0	0	1	0	3	0	0	0	0	2	0	3	6	0	8	0	0	0	0	0	0	23
OTU979	1	0	0	0	0	0	0	0	0	0	0	0	0	4	0	0	0	0	2	1	6	0	4	3	0	0	2	0	0	0	23
OTU980	0	0	0	0	0	0	0	0	0	0	0	0	0	0	0	0	0	0	0	0	0	0	0	5	0	2	0	1	2	13	23
OTU981	0	0	0	0	0	0	0	0	0	0	0	0	1	0	0	0	0	0	5	0	2	1	1	0	0	0	0	0	1	12	23
OTU982	0	3	0	0	0	0	1	2	7	0	0	1	0	2	0	0	1	0	0	2	1	0	0	0	1	0	0	0	0	2	23
OTU983	7	1	9	0	0	2	0	0	0	0	0	0	0	0	1	0	0	0	2	0	0	0	0	0	0	0	0	0	0	0	22
OTU984	3	1	12	0	0	1	2	0	0	0	0	0	0	3	0	0	0	0	0	0	0	0	0	0	0	0	0	0	0	0	22
OTU985	0	2	0	0	2	1	0	0	0	3	0	4	0	0	0	1	0	3	0	0	0	3	0	1	0	1	0	0	0	1	22
OTU986	0	0	0	3	0	0	0	0	1	0	0	0	0	0	0	1	0	0	0	0	0	1	2	5	4	2	3	0	0	0	22
OTU987	4	4	0	0	1	1	0	0	0	0	0	1	0	0	0	0	0	0	0	4	0	2	0	0	4	0	0	0	1	0	22
OTU988	0	0	0	0	0	0	0	0	0	4	0	1	1	0	0	0	0	14	0	0	0	0	0	1	0	0	0	0	0	1	22
OTU989	0	0	0	0	1	0	0	0	0	0	0	0	0	0	0	0	0	0	0	0	0	3	1	10	0	1	0	1	1	4	22
OTU990	0	3	0	0	0	0	5	0	4	0	0	0	0	0	1	3	0	0	0	3	0	0	0	0	0	0	0	3	0	0	22
OTU991	0	0	0	0	0	0	0	0	0	0	19	0	0	0	0	2	0	0	0	0	0	0	0	0	0	0	0	0	0	0	21
OTU992	0	0	1	0	2	0	0	0	1	1	0	1	0	0	0	1	0	0	2	0	4	0	5	0	0	1	0	1	1	0	21
OTU993	0	0	21	0	0	0	0	0	0	0	0	0	0	0	0	0	0	0	0	0	0	0	0	0	0	0	0	0	0	0	21
OTU994	9	3	0	2	1	2	0	0	0	0	0	0	0	0	0	0	0	0	2	0	0	0	0	0	0	2	0	0	0	0	21
OTU995	0	0	0	0	0	0	0	0	0	0	0	0	0	0	0	0	0	0	0	0	0	1	0	20	0	0	0	0	0	0	21
OTU996	0	0	0	0	0	1	0	0	2	3	0	0	0	3	0	0	0	0	0	1	2	4	2	1	1	0	0	0	0	1	21
OTU997	0	0	0	0	0	0	0	1	1	3	2	0	3	0	4	0	0	1	0	0	0	0	1	0	3	1	0	0	0	0	20
OTU998	0	1	0	0	2	0	0	0	0	0	0	0	0	7	0	0	0	0	0	0	0	2	0	6	0	0	0	0	2	0	20
OTU999	0	0	0	0	0	0	0	0	0	0	0	2	0	0	0	0	0	0	5	1	0	1	3	3	0	3	2	0	0	0	20
OTU1000	7	1	0	0	4	0	0	0	0	0	0	0	0	0	0	0	0	0	1	0	1	1	0	3	0	1	0	0	0	1	20
OTU1001	0	0	0	0	0	0	0	0	0	0	0	0	0	0	0	0	0	0	0	0	0	0	0	19	0	0	0	1	0	0	20
OTU1002	0	0	0	3	0	0	0	0	0	0	0	0	0	3	0	0	0	1	0	0	0	0	2	1	0	0	2	0	0	8	20
OTU1003	0	0	0	6	2	0	0	0	0	0	0	0	0	0	0	0	0	0	0	0	0	1	4	3	0	0	1	0	2	0	19

OTU1004	0	0	0	0	0	0	0	0	0	0	0	0	0	0	0	0	0	18	0	0	0	0	0	0	0	0	0	0	0	0	18
OTU1005	0	0	0	0	0	0	0	0	0	0	0	0	0	0	0	0	0	0	0	0	9	0	0	0	0	0	0	0	0	9	18
OTU1006	0	0	0	0	0	0	0	0	0	0	0	0	0	0	0	0	0	15	0	0	0	0	1	2	0	0	0	0	0	0	18
OTU1007	0	0	6	0	0	0	2	2	0	2	1	0	0	0	0	0	0	0	1	1	0	0	0	0	2	0	0	0	0	0	17
OTU1008	0	0	0	0	0	0	1	0	0	4	10	0	1	0	0	1	0	0	0	0	0	0	0	0	0	0	0	0	0	0	17
OTU1009	0	0	0	0	0	0	0	0	0	0	5	0	10	0	2	0	0	0	0	0	0	0	0	0	0	0	0	0	0	0	17
OTU1010	0	0	0	0	0	0	0	0	0	0	0	0	0	5	0	0	0	0	1	3	0	0	1	0	7	0	0	0	0	0	17
OTU1011	0	0	0	0	1	0	0	0	1	0	0	0	0	2	1	0	0	0	2	1	1	1	2	2	0	3	0	0	0	0	17
OTU1012	0	0	0	0	0	0	0	0	0	0	0	0	0	0	0	0	0	1	0	0	1	0	0	13	0	0	0	1	0	1	17
OTU1013	0	0	0	0	0	0	0	1	0	2	0	0	0	0	0	0	0	2	1	0	1	1	0	5	0	1	0	0	1	2	17
OTU1014	0	0	0	0	0	0	0	0	0	1	0	0	0	1	0	0	1	0	0	3	2	1	2	1	0	0	2	1	2	0	17
OTU1015	0	0	0	0	1	0	0	0	0	0	0	0	0	0	0	0	0	0	0	0	0	0	0	0	0	0	0	2	8	6	17
OTU1016	0	0	0	2	1	0	0	0	0	0	0	0	0	8	0	0	0	0	0	0	4	0	0	0	0	0	0	0	0	2	17
OTU1017	0	0	0	0	0	0	0	0	0	0	0	0	0	0	0	0	0	17	0	0	0	0	0	0	0	0	0	0	0	0	17
OTU1018	9	0	0	0	0	0	0	1	0	0	0	1	0	2	0	0	0	0	0	0	2	0	0	0	2	0	0	0	0	0	17
OTU1019	0	1	0	0	0	0	3	4	0	0	1	1	0	0	0	2	0	0	2	1	0	0	0	0	1	0	0	0	0	0	16
OTU1020	0	0	0	0	1	2	1	0	0	0	0	1	0	0	0	2	0	0	0	2	0	0	1	0	4	0	1	0	0	1	16
OTU1021	0	0	0	0	0	3	0	0	3	0	1	0	6	0	0	0	0	1	0	0	0	0	2	0	0	0	0	0	0	0	16
OTU1022	0	1	0	2	0	0	0	0	0	0	0	1	0	7	0	0	0	0	0	0	3	0	1	0	0	0	0	0	0	1	16
OTU1023	0	0	0	0	0	0	0	0	0	0	0	0	9	0	0	1	0	5	1	0	0	0	0	0	0	0	0	0	0	0	16
OTU1024	0	0	0	0	0	0	0	0	0	0	0	4	0	0	0	0	0	0	0	4	0	0	0	0	0	1	0	0	7	0	16
OTU1025	0	0	0	0	0	0	0	0	0	0	6	0	1	0	0	0	0	1	0	0	0	0	0	0	1	0	0	0	4	3	16
OTU1026	7	0	0	1	0	0	0	0	0	0	0	0	1	1	0	0	0	0	0	0	1	4	0	1	0	0	0	0	0	0	16
OTU1027	0	0	0	0	0	0	0	0	0	0	0	0	0	0	0	0	0	3	0	0	0	0	3	0	0	0	4	0	6	0	16
OTU1028	0	0	0	0	0	0	0	0	0	0	0	0	0	0	0	0	0	0	0	2	0	2	0	2	0	10	0	0	0	0	16
OTU1029	0	0	0	0	0	0	0	0	0	0	0	0	0	5	0	0	0	0	1	0	0	0	4	0	6	0	0	0	0	0	16

OTU1030	0	1	0	3	0	0	0	0	0	0	0	0	0	0	0	0	0	1	0	0	0	5	1	1	0	1	2	1	0	0	16
OTU1031	0	0	0	0	0	0	0	0	0	0	0	0	0	0	0	0	0	0	0	0	0	0	0	7	3	6	0	0	0	0	16
OTU1032	2	4	0	0	3	1	0	0	0	4	0	0	0	0	0	0	0	0	0	0	0	0	0	1	0	0	0	0	0	0	15
OTU1033	0	0	1	0	0	0	1	1	0	1	0	1	0	1	2	1	0	0	1	1	0	0	1	0	0	1	1	0	0	1	15
OTU1034	0	0	0	0	0	0	0	0	0	0	0	0	0	0	0	0	0	0	0	0	0	10	5	0	0	0	0	0	0	0	15
OTU1035	0	0	0	0	0	0	0	0	0	0	0	0	0	0	0	0	0	0	0	0	0	0	0	15	0	0	0	0	0	0	15
OTU1036	0	0	0	0	0	0	0	0	0	0	0	3	0	0	0	0	0	5	0	1	0	0	2	1	0	2	1	0	0	0	15
OTU1037	0	9	0	0	0	0	0	0	0	0	0	1	0	0	0	0	0	0	0	0	0	4	0	0	0	1	0	0	0	0	15
OTU1038	2	2	2	0	4	3	0	0	0	0	0	0	0	0	0	0	0	0	0	0	0	0	1	0	0	0	0	1	0	0	15
OTU1039	0	0	1	0	0	0	1	4	0	0	0	0	1	0	0	0	0	3	0	4	0	0	0	0	1	0	0	0	0	0	15
OTU1040	1	1	0	0	0	0	0	2	0	3	1	0	0	0	0	0	0	0	0	0	0	0	7	0	0	0	0	0	0	0	15
OTU1041	0	0	0	0	0	1	2	3	0	5	0	0	0	0	4	0	0	0	0	0	0	0	0	0	0	0	0	0	0	0	15
OTU1042	0	0	0	0	0	0	0	0	0	0	0	1	0	0	0	0	0	14	0	0	0	0	0	0	0	0	0	0	0	0	15
OTU1043	5	0	0	0	2	0	0	0	0	2	0	0	0	0	3	2	0	0	0	0	0	0	0	0	0	0	0	0	0	0	14
OTU1044	0	0	0	0	1	0	0	2	1	0	1	1	0	0	0	1	0	0	1	0	0	1	0	0	1	2	1	0	0	1	14
OTU1045	0	0	0	0	0	0	0	0	0	0	0	0	13	0	0	1	0	0	0	0	0	0	0	0	0	0	0	0	0	0	14
OTU1046	0	0	0	0	0	0	0	0	0	0	0	0	0	0	0	0	0	0	0	0	0	0	2	1	0	0	5	2	0	4	14
OTU1047	6	0	0	6	0	0	0	0	0	0	0	0	0	0	0	0	0	0	2	0	0	0	0	0	0	0	0	0	0	0	14
OTU1048	2	0	0	0	0	3	1	0	0	0	0	0	3	0	0	0	0	3	0	0	1	1	0	0	0	0	0	0	0	0	14
OTU1049	0	0	0	0	0	0	0	0	0	0	0	0	0	6	4	0	0	0	0	0	0	0	1	3	0	0	0	0	0	0	14
OTU1050	1	0	0	0	0	2	0	0	0	1	0	0	0	0	1	0	0	0	0	1	0	3	0	1	0	0	3	0	0	1	14
OTU1051	0	0	0	0	0	0	0	0	0	0	0	0	0	0	0	0	0	0	0	0	0	0	0	14	0	0	0	0	0	0	14
OTU1052	0	0	0	0	0	0	0	0	0	0	0	0	0	6	0	0	0	0	0	3	0	0	1	0	4	0	0	0	0	0	14
OTU1053	0	0	0	0	0	0	0	0	0	0	0	0	0	0	0	0	0	1	0	1	0	0	0	3	0	8	0	0	0	1	14
OTU1054	0	0	0	2	0	1	0	2	0	1	0	1	0	0	0	0	0	0	2	0	0	1	0	0	0	1	1	0	0	1	13
OTU1055	0	2	1	0	2	2	0	0	0	0	0	0	0	0	0	0	0	1	0	0	0	1	2	2	0	0	0	0	0	0	13

OTU1056	3	2	0	0	7	0	0	0	0	0	0	0	0	0	0	0	0	1	0	0	0	0	0	0	0	0	0	0	0	0	13
OTU1057	0	0	0	0	0	0	1	1	1	1	1	0	4	0	0	0	1	1	1	0	0	0	0	0	1	0	0	0	0	0	13
OTU1058	0	0	0	1	0	0	0	1	1	2	2	1	0	0	0	0	0	0	0	0	0	0	1	0	0	0	1	1	2	0	13
OTU1059	0	2	0	1	0	1	0	0	0	0	1	1	0	0	1	0	1	0	1	0	0	1	1	0	1	0	0	1	0	0	13
OTU1060	0	0	0	0	0	0	0	0	0	0	0	0	0	1	0	0	1	0	0	3	0	0	1	0	7	0	0	0	0	0	13
OTU1061	0	2	0	0	2	0	0	0	0	0	0	0	0	0	0	0	0	2	0	0	3	0	0	1	0	0	0	0	0	3	13
OTU1062	0	0	0	0	0	0	0	0	0	0	0	0	0	0	0	0	0	11	2	0	0	0	0	0	0	0	0	0	0	0	13
OTU1063	0	0	0	0	0	0	0	0	0	0	0	0	0	0	0	0	0	13	0	0	0	0	0	0	0	0	0	0	0	0	13
OTU1064	0	0	0	0	0	0	0	0	0	0	0	0	0	0	0	0	0	0	1	3	0	3	1	0	0	0	4	0	1	0	13
OTU1065	0	0	0	0	0	0	0	0	0	0	0	0	0	1	0	1	0	1	0	0	0	5	0	2	2	0	0	0	0	1	13
OTU1066	0	0	0	0	0	0	0	0	0	0	0	0	0	0	0	0	0	0	0	0	0	0	0	2	0	0	0	0	0	11	13
OTU1067	1	3	0	0	0	1	0	0	0	0	0	0	0	0	0	0	0	0	0	0	0	0	0	0	6	1	1	0	0	0	13
OTU1068	12	0	0	0	1	0	0	0	0	0	0	0	0	0	0	0	0	0	0	0	0	0	0	0	0	0	0	0	0	0	13
OTU1069	8	1	0	0	0	3	0	1	0	0	0	0	0	0	0	0	0	0	0	0	0	0	0	0	0	0	0	0	0	0	13
OTU1070	3	0	0	0	0	0	0	1	1	3	1	0	2	0	0	0	0	0	0	0	0	0	0	0	1	1	0	0	0	0	13
OTU1071	0	0	0	0	0	0	0	0	0	0	0	0	0	0	0	0	0	0	0	1	1	4	2	1	0	0	0	0	4	0	13
OTU1072	0	1	8	0	0	0	0	0	0	0	3	0	0	0	0	0	0	0	0	0	0	0	0	0	0	0	0	0	0	0	12
OTU1073	0	0	0	12	0	0	0	0	0	0	0	0	0	0	0	0	0	0	0	0	0	0	0	0	0	0	0	0	0	0	12
OTU1074	0	0	0	0	0	0	5	1	2	1	0	0	2	0	0	0	0	0	1	0	0	0	0	0	0	0	0	0	0	0	12
OTU1075	0	0	1	0	2	0	0	4	0	1	0	1	0	0	0	0	0	0	1	0	0	0	0	0	0	0	0	1	0	1	12
OTU1076	0	0	0	0	0	0	0	0	0	0	0	0	0	2	0	0	0	0	3	4	0	3	0	0	0	0	0	0	0	0	12
OTU1077	0	0	0	0	0	0	0	0	0	0	0	0	0	2	0	0	0	6	0	0	2	0	1	0	0	0	1	0	0	0	12
OTU1078	0	0	0	0	0	0	0	0	0	0	0	0	0	0	0	0	0	1	0	0	0	0	0	2	0	0	0	0	0	9	12
OTU1079	0	0	0	1	0	0	0	0	0	0	0	0	0	0	0	0	0	0	0	0	2	0	1	2	0	0	3	3	0	0	12
OTU1080	0	0	0	0	0	0	0	0	0	0	0	0	0	0	0	0	0	1	2	0	4	0	0	1	0	0	0	0	3	1	12
OTU1081	0	0	0	0	0	0	0	0	0	0	0	0	0	0	0	0	0	0	0	0	0	0	0	0	0	1	0	0	2	9	12

OTU1082	0	0	0	0	0	0	0	0	0	0	0	0	0	0	0	0	0	2	0	0	1	1	3	4	0	0	0	1	0	0	12
OTU1083	0	1	0	0	0	0	0	0	0	0	0	0	0	0	0	0	0	0	0	0	1	1	0	0	0	0	1	8	0	0	12
OTU1084	7	0	0	1	3	0	0	0	0	0	0	0	0	0	0	0	0	0	0	0	0	0	0	0	0	0	0	0	0	0	11
OTU1085	11	0	0	0	0	0	0	0	0	0	0	0	0	0	0	0	0	0	0	0	0	0	0	0	0	0	0	0	0	0	11
OTU1086	0	0	1	0	1	0	0	0	0	0	2	0	1	0	0	0	0	1	0	0	0	0	0	1	0	0	0	0	3	1	11
OTU1087	1	0	0	0	8	0	0	0	2	0	0	0	0	0	0	0	0	0	0	0	0	0	0	0	0	0	0	0	0	0	11
OTU1088	1	0	1	2	0	2	1	0	3	0	0	0	0	0	0	0	0	0	0	0	0	0	0	1	0	0	0	0	0	0	11
OTU1089	0	0	0	0	0	5	0	0	0	1	5	0	0	0	0	0	0	0	0	0	0	0	0	0	0	0	0	0	0	0	11
OTU1090	0	0	4	0	0	0	0	0	1	0	0	0	0	0	0	0	0	0	0	0	2	0	0	0	0	0	0	0	1	3	11
OTU1091	0	0	0	0	1	0	0	0	0	0	0	2	0	0	0	0	0	0	0	0	1	0	1	1	0	0	4	0	0	1	11
OTU1092	0	0	0	0	0	0	0	0	0	0	0	0	0	0	7	0	0	0	0	0	2	0	0	2	0	0	0	0	0	0	11
OTU1093	0	0	0	0	0	0	0	0	0	6	0	0	2	0	0	1	0	2	0	0	0	0	0	0	0	0	0	0	0	0	11
OTU1094	0	0	0	0	0	0	0	0	0	0	0	0	0	0	0	0	0	1	0	0	0	0	0	3	2	5	0	0	0	0	11
OTU1095	0	0	0	0	0	0	0	0	0	0	0	4	0	2	0	0	0	0	0	0	0	2	0	0	1	2	0	0	0	0	11
OTU1096	1	0	0	0	0	0	0	0	0	0	0	0	0	0	0	0	0	0	0	0	0	0	0	0	0	0	2	7	0	1	11
OTU1097	0	0	0	0	0	0	0	0	0	0	0	0	0	0	0	0	0	0	0	0	5	0	1	1	0	0	0	0	4	0	11
OTU1098	0	0	0	0	0	0	0	0	0	0	0	0	0	2	0	0	0	0	5	1	0	0	0	0	0	0	0	0	1	2	11
OTU1099	3	1	0	0	0	4	0	1	0	0	0	0	1	0	0	0	0	1	0	0	0	0	0	0	0	0	0	0	0	0	11
OTU1100	0	0	0	0	2	0	0	1	0	0	0	0	0	1	0	2	0	0	5	0	0	0	0	0	0	0	0	0	0	0	11
OTU1101	0	1	0	1	0	1	0	0	2	0	0	0	0	0	0	0	0	0	0	1	0	3	1	1	0	0	0	0	0	0	11
OTU1102	0	0	0	0	0	0	0	0	0	0	0	0	0	0	0	0	0	11	0	0	0	0	0	0	0	0	0	0	0	0	11
OTU1103	0	0	0	0	0	0	0	0	0	5	3	0	2	0	0	0	0	0	1	0	0	0	0	0	0	0	0	0	0	0	11
OTU1104	0	0	0	0	0	0	0	0	0	0	0	0	0	0	0	0	0	0	0	3	0	2	1	2	0	0	2	0	1	0	11
OTU1105	0	0	0	0	0	0	0	0	1	0	0	0	0	0	0	0	0	0	0	1	0	0	1	1	0	0	0	5	2	0	11
OTU1106	0	0	0	0	0	0	0	0	0	0	0	0	0	0	0	0	0	0	0	0	0	0	11	0	0	0	0	0	0	0	11
OTU1107	0	0	0	0	0	0	0	0	0	0	0	0	0	0	0	0	0	0	0	0	0	0	9	1	0	1	0	0	0	0	11

OTU1108	0	0	0	0	0	0	0	0	0	0	0	0	0	10	0	0	0	0	0	0	0	0	0	1	0	0	0	0	0	0	11
OTU1109	0	0	0	0	0	0	0	0	0	0	0	0	0	0	0	0	0	0	0	1	0	0	0	0	10	0	0	0	0	0	11
OTU1110	0	0	0	0	0	0	0	0	0	0	0	0	0	0	0	0	0	0	0	1	0	0	6	0	0	0	0	0	0	4	11
OTU1111	7	1	2	0	0	0	0	0	0	0	0	0	0	0	0	0	0	0	0	0	0	0	0	0	0	0	0	0	0	0	10
OTU1112	4	1	0	0	0	0	1	0	0	0	1	0	1	0	0	0	0	0	0	0	0	0	1	0	0	1	0	0	0	0	10
OTU1113	4	0	0	0	0	0	0	2	1	0	0	1	0	0	0	0	0	0	0	0	0	1	0	0	0	0	0	0	1	0	10
OTU1114	0	10	0	0	0	0	0	0	0	0	0	0	0	0	0	0	0	0	0	0	0	0	0	0	0	0	0	0	0	0	10
OTU1115	0	0	9	0	0	0	0	0	0	0	0	0	0	0	0	0	0	0	0	0	0	0	0	1	0	0	0	0	0	0	10
OTU1116	0	0	1	0	4	1	0	1	0	0	0	1	0	0	0	1	0	0	1	0	0	0	0	0	0	0	0	0	0	0	10
OTU1117	0	0	0	0	0	0	0	0	0	3	0	0	0	0	0	2	0	0	0	0	0	0	1	4	0	0	0	0	0	0	10
OTU1118	0	0	0	0	0	3	0	0	0	1	0	0	0	6	0	0	0	0	0	0	0	0	0	0	0	0	0	0	0	0	10
OTU1119	0	0	0	0	0	2	0	0	0	0	8	0	0	0	0	0	0	0	0	0	0	0	0	0	0	0	0	0	0	0	10
OTU1120	0	0	0	0	0	0	0	0	0	0	8	0	0	0	0	2	0	0	0	0	0	0	0	0	0	0	0	0	0	0	10
OTU1121	0	0	0	0	0	0	0	0	0	0	0	0	0	6	0	0	0	0	0	0	0	0	2	1	0	0	0	1	0	0	10
OTU1122	0	0	0	0	0	0	0	0	0	0	0	0	0	0	0	0	1	0	0	0	0	2	2	3	0	0	1	1	0	0	10
OTU1123	0	0	0	0	0	0	0	0	0	0	0	0	0	0	0	0	0	0	0	0	0	2	4	3	0	0	0	0	0	1	10
OTU1124	0	0	3	0	0	0	0	0	0	0	0	0	0	0	0	0	0	0	0	0	0	0	7	0	0	0	0	0	0	0	10
OTU1125	0	0	0	0	0	0	0	0	0	0	0	0	0	0	0	0	0	0	0	0	0	0	0	7	3	0	0	0	0	0	10
OTU1126	0	0	0	1	0	0	0	0	3	0	0	1	0	0	2	0	0	0	1	0	0	0	0	1	0	0	0	1	0	0	10
OTU1127	0	0	0	0	0	5	0	0	0	0	0	2	0	0	0	0	0	0	0	0	0	0	0	0	0	0	0	2	0	1	10
OTU1128	10	0	0	0	0	0	0	0	0	0	0	0	0	0	0	0	0	0	0	0	0	0	0	0	0	0	0	0	0	0	10
OTU1129	1	0	0	5	3	0	0	0	0	0	0	0	0	0	0	0	0	0	0	0	0	0	0	1	0	0	0	0	0	0	10
OTU1130	0	0	0	0	1	0	0	0	0	0	0	0	0	0	0	0	0	3	0	0	0	0	3	0	0	1	0	0	0	2	10
OTU1131	5	0	0	0	1	0	0	0	0	0	0	0	1	0	0	0	0	0	0	0	2	0	0	0	0	0	0	0	0	1	10
OTU1132	0	0	0	0	0	0	0	0	6	0	0	3	0	1	0	0	0	0	0	0	0	0	0	0	0	0	0	0	0	0	10
OTU1133	0	0	0	0	0	0	0	0	0	0	0	0	0	0	0	1	0	4	5	0	0	0	0	0	0	0	0	0	0	0	10

OTU1134	0	0	0	0	0	0	0	0	0	0	0	0	0	0	0	0	0	3	3	2	0	0	0	0	2	0	0	0	0	0	10
OTU1135	0	0	0	0	0	0	0	0	0	0	0	0	0	0	0	0	0	0	0	0	0	3	0	2	1	0	4	0	0	0	10
OTU1136	0	0	0	0	0	0	0	0	0	0	0	0	0	0	0	0	0	0	0	0	2	0	0	6	0	0	0	0	1	1	10
OTU1137	0	0	0	0	0	0	0	0	0	0	0	0	0	2	0	0	0	0	1	0	1	0	1	0	0	0	0	1	3	1	10
OTU1138	2	1	0	0	2	0	0	0	0	1	0	0	0	0	0	0	0	1	0	0	0	2	0	0	0	0	0	0	0	0	9
OTU1139	1	0	0	1	0	0	0	0	0	0	0	0	0	0	0	0	0	2	0	0	0	1	1	0	1	0	0	0	0	2	9
OTU1140	0	0	2	0	0	0	1	0	1	0	0	0	2	0	1	0	0	1	1	0	0	0	0	0	0	0	0	0	0	0	9
OTU1141	0	0	8	0	1	0	0	0	0	0	0	0	0	0	0	0	0	0	0	0	0	0	0	0	0	0	0	0	0	0	9
OTU1142	0	0	0	1	0	0	0	0	0	0	0	0	0	0	0	0	0	4	0	0	0	0	0	4	0	0	0	0	0	0	9
OTU1143	2	0	0	1	0	3	2	0	1	0	0	0	0	0	0	0	0	0	0	0	0	0	0	0	0	0	0	0	0	0	9
OTU1144	0	0	0	0	0	0	0	0	8	0	0	1	0	0	0	0	0	0	0	0	0	0	0	0	0	0	0	0	0	0	9
OTU1145	0	0	0	0	0	0	2	0	0	7	0	0	0	0	0	0	0	0	0	0	0	0	0	0	0	0	0	0	0	0	9
OTU1146	0	0	0	0	0	0	0	0	0	0	0	0	0	2	0	0	2	1	0	0	0	0	4	0	0	0	0	0	0	0	9
OTU1147	0	0	0	1	1	0	0	0	0	0	0	1	0	0	2	0	0	0	2	0	0	0	0	0	0	0	1	1	0	0	9
OTU1148	0	0	0	1	0	0	0	0	0	0	0	0	0	0	0	0	8	0	0	0	0	0	0	0	0	0	0	0	0	0	9
OTU1149	0	0	0	0	0	0	0	0	0	0	0	0	0	0	0	0	0	9	0	0	0	0	0	0	0	0	0	0	0	0	9
OTU1150	0	0	0	0	0	0	0	0	0	0	0	0	0	0	0	0	0	8	0	0	0	0	0	0	1	0	0	0	0	0	9
OTU1151	0	0	0	0	0	0	0	0	0	0	0	0	0	0	1	0	0	1	1	3	0	0	1	0	0	0	0	0	2	0	9
OTU1152	0	0	0	0	0	1	0	0	0	1	0	0	0	0	0	1	0	0	0	0	0	0	0	2	0	4	0	0	0	0	9
OTU1153	0	0	0	0	0	0	0	0	0	0	0	0	0	0	1	0	0	0	0	0	0	0	0	0	0	0	8	0	0	0	9
OTU1154	0	0	0	0	0	0	0	0	0	0	0	0	0	0	0	0	0	0	2	0	0	0	0	3	0	0	0	3	0	1	9
OTU1155	0	0	0	0	0	0	0	0	0	0	0	0	0	3	0	0	0	0	0	0	1	0	0	0	0	0	0	0	0	5	9
OTU1156	0	0	0	0	0	0	0	0	0	0	0	0	0	1	0	0	0	0	0	0	0	0	0	0	0	0	0	1	0	7	9
OTU1157	0	0	0	0	0	0	0	0	0	0	0	0	0	0	0	0	0	0	0	0	0	0	0	0	0	0	0	0	2	7	9
OTU1158	0	0	9	0	0	0	0	0	0	0	0	0	0	0	0	0	0	0	0	0	0	0	0	0	0	0	0	0	0	0	9
OTU1159	0	0	0	0	0	0	0	0	1	5	0	0	3	0	0	0	0	0	0	0	0	0	0	0	0	0	0	0	0	0	9

OTU1160	3	0	1	0	0	0	0	0	1	0	1	0	0	0	2	0	0	0	0	1	0	0	0	0	0	0	0	0	0	0	9
OTU1161	0	0	0	0	0	0	0	0	7	0	0	0	0	0	0	0	0	0	2	0	0	0	0	0	0	0	0	0	0	0	9
OTU1162	4	0	0	2	0	0	0	1	0	0	0	0	0	0	0	0	0	0	0	0	0	2	0	0	0	0	0	0	0	0	9
OTU1163	0	0	0	0	0	0	0	0	0	0	0	0	0	0	0	0	0	0	1	0	0	0	0	4	0	0	0	0	0	4	9
OTU1164	0	0	0	0	1	0	0	0	0	0	0	0	0	0	0	0	0	0	0	0	0	0	0	0	0	0	0	0	0	8	9
OTU1165	0	0	0	0	0	0	0	0	0	0	0	0	0	0	0	0	0	0	0	0	0	0	0	0	0	0	0	0	0	9	9
OTU1166	0	1	1	0	0	0	1	0	0	3	0	0	0	0	0	0	0	0	0	0	0	2	0	0	0	0	0	0	0	0	8
OTU1167	0	0	8	0	0	0	0	0	0	0	0	0	0	0	0	0	0	0	0	0	0	0	0	0	0	0	0	0	0	0	8
OTU1168	0	0	0	8	0	0	0	0	0	0	0	0	0	0	0	0	0	0	0	0	0	0	0	0	0	0	0	0	0	0	8
OTU1169	0	0	0	0	0	0	0	1	0	7	0	0	0	0	0	0	0	0	0	0	0	0	0	0	0	0	0	0	0	0	8
OTU1170	0	0	0	0	0	0	0	0	0	0	0	1	0	1	0	0	0	0	0	2	0	3	0	1	0	0	0	0	0	0	8
OTU1171	0	0	0	0	0	0	0	0	2	2	0	0	4	0	0	0	0	0	0	0	0	0	0	0	0	0	0	0	0	0	8
OTU1172	0	1	0	0	0	0	0	0	0	0	0	0	0	3	1	0	0	0	0	2	0	0	0	0	0	0	1	0	0	0	8
OTU1173	2	0	0	0	3	0	0	0	0	0	0	0	0	1	0	1	0	0	0	0	0	0	0	0	0	1	0	0	0	0	8
OTU1174	0	0	0	0	0	0	0	0	0	0	0	0	0	0	8	0	0	0	0	0	0	0	0	0	0	0	0	0	0	0	8
OTU1175	0	0	0	0	1	0	0	0	0	1	2	0	0	0	1	0	0	0	3	0	0	0	0	0	0	0	0	0	0	0	8
OTU1176	0	0	0	0	0	0	0	0	0	0	0	0	0	0	0	0	0	4	0	0	0	0	0	2	0	0	0	0	1	1	8
OTU1177	0	0	0	0	0	0	0	0	0	0	0	0	0	0	0	0	0	8	0	0	0	0	0	0	0	0	0	0	0	0	8
OTU1178	0	0	0	0	0	0	0	0	0	0	0	0	0	0	0	0	0	2	0	4	0	0	0	0	0	0	0	0	0	2	8
OTU1179	0	1	0	0	0	0	0	0	2	0	0	0	0	0	0	2	1	0	0	2	0	0	0	0	0	0	0	0	0	0	8
OTU1180	0	0	0	0	0	0	0	0	0	0	0	0	0	0	0	0	0	0	0	0	0	0	0	7	0	1	0	0	0	0	8
OTU1181	0	0	0	0	0	0	0	0	0	0	0	0	0	0	0	0	0	5	0	0	0	2	0	1	0	0	0	0	0	0	8
OTU1182	0	0	0	0	0	0	0	0	0	0	0	1	0	0	0	0	0	0	0	0	0	1	0	3	0	0	0	0	0	3	8
OTU1183	0	0	0	0	0	0	0	0	0	0	0	0	0	0	0	0	0	0	0	0	0	0	0	8	0	0	0	0	0	0	8
OTU1184	0	0	0	0	0	0	0	0	0	0	0	0	0	0	0	0	0	0	0	0	0	0	0	0	0	0	8	0	0	0	8
OTU1185	0	0	0	0	0	0	0	0	0	0	0	0	0	0	0	0	0	0	0	1	0	0	4	0	0	1	1	0	1	0	8

OTU1186	0	0	0	0	0	0	0	0	0	0	0	0	0	0	0	0	0	0	0	0	3	0	0	0	0	0	0	3	2	0	8
OTU1187	0	3	0	0	4	1	0	0	0	0	0	0	0	0	0	0	0	0	0	0	0	0	0	0	0	0	0	0	0	0	8
OTU1188	0	0	0	0	0	0	0	4	0	4	0	0	0	0	0	0	0	0	0	0	0	0	0	0	0	0	0	0	0	0	8
OTU1189	0	0	0	0	0	0	0	0	2	0	0	2	0	0	4	0	0	0	0	0	0	0	0	0	0	0	0	0	0	0	8
OTU1190	0	0	0	0	0	0	0	0	0	0	0	0	8	0	0	0	0	0	0	0	0	0	0	0	0	0	0	0	0	0	8
OTU1191	0	0	0	0	0	0	0	0	0	0	0	0	0	0	0	4	0	0	0	0	0	0	4	0	0	0	0	0	0	0	8
OTU1192	0	0	0	0	0	0	0	0	0	0	0	0	0	0	0	0	0	8	0	0	0	0	0	0	0	0	0	0	0	0	8
OTU1193	0	0	0	0	0	0	0	0	0	0	0	1	5	0	0	0	0	2	0	0	0	0	0	0	0	0	0	0	0	0	8
OTU1194	0	0	0	0	0	0	0	0	0	0	0	0	0	0	0	0	0	2	0	0	0	0	0	6	0	0	0	0	0	0	8
OTU1195	0	0	0	0	0	0	0	0	0	0	0	0	0	0	0	3	0	5	0	0	0	0	0	0	0	0	0	0	0	0	8
OTU1196	0	0	0	0	0	0	0	0	0	1	0	1	0	0	0	0	0	0	2	0	0	0	1	2	0	0	0	1	0	0	8
OTU1197	0	0	0	0	0	0	0	0	0	0	0	0	0	0	0	0	0	0	0	0	0	0	0	8	0	0	0	0	0	0	8
OTU1198	0	0	0	0	0	0	0	0	0	0	0	0	0	0	0	0	0	0	0	0	0	0	0	8	0	0	0	0	0	0	8
OTU1199	0	0	0	0	0	0	0	0	0	0	0	0	0	0	0	0	0	0	0	0	0	0	0	8	0	0	0	0	0	0	8
OTU1200	0	0	0	0	0	0	0	0	0	0	0	0	0	0	0	0	0	0	0	0	0	0	0	3	0	0	4	0	1	0	8
OTU1201	4	0	0	2	1	0	0	0	0	0	0	0	0	0	0	0	0	0	0	0	0	0	0	0	0	0	0	0	0	0	7
OTU1202	5	0	0	0	2	0	0	0	0	0	0	0	0	0	0	0	0	0	0	0	0	0	0	0	0	0	0	0	0	0	7
OTU1203	5	1	0	0	0	0	0	0	0	0	0	1	0	0	0	0	0	0	0	0	0	0	0	0	0	0	0	0	0	0	7
OTU1204	0	1	0	0	1	0	1	0	0	0	0	1	0	0	1	0	0	0	0	1	1	0	0	0	0	0	0	0	0	0	7
OTU1205	0	0	0	0	0	1	0	0	0	1	0	0	0	0	0	0	0	2	0	0	0	0	1	2	0	0	0	0	0	0	7
OTU1206	0	0	1	1	1	0	1	0	0	0	0	0	0	0	0	0	0	0	0	0	0	0	0	1	2	0	0	0	0	0	7
OTU1207	0	0	1	0	0	0	0	1	3	0	0	0	0	0	0	0	0	0	0	0	0	0	0	2	0	0	0	0	0	0	7
OTU1208	0	0	0	0	0	0	0	0	1	0	0	4	0	0	0	1	0	0	0	0	0	0	0	0	0	1	0	0	0	0	7
OTU1209	0	0	0	0	0	0	0	0	0	6	1	0	0	0	0	0	0	0	0	0	0	0	0	0	0	0	0	0	0	0	7
OTU1210	0	0	5	0	0	0	0	0	0	2	0	0	0	0	0	0	0	0	0	0	0	0	0	0	0	0	0	0	0	0	7
OTU1211	0	0	0	0	0	0	0	0	1	1	0	0	0	0	0	0	0	0	0	0	0	0	5	0	0	0	0	0	0	0	7

OTU1212	0	0	0	0	0	0	0	0	0	0	0	0	7	0	0	0	0	0	0	0	0	0	0	0	0	0	0	0	0	0	7
OTU1213	0	0	0	0	0	0	0	0	0	0	0	0	0	1	0	0	0	0	0	0	0	0	0	3	0	0	2	0	1	0	7
OTU1214	0	0	0	0	0	0	0	0	0	0	0	1	0	0	0	0	0	4	0	0	0	0	0	2	0	0	0	0	0	0	7
OTU1215	0	0	0	0	0	0	0	0	0	0	0	0	0	0	0	0	0	6	0	1	0	0	0	0	0	0	0	0	0	0	7
OTU1216	0	0	0	0	0	0	0	0	0	0	0	0	0	0	0	0	0	4	0	0	0	0	0	3	0	0	0	0	0	0	7
OTU1217	0	0	0	0	0	0	0	0	0	0	0	0	0	0	0	0	0	0	7	0	0	0	0	0	0	0	0	0	0	0	7
OTU1218	0	0	0	0	0	0	0	0	0	1	0	0	0	1	0	0	0	0	3	1	0	0	0	0	1	0	0	0	0	0	7
OTU1219	0	0	0	0	0	0	0	0	0	0	0	1	0	0	0	1	0	1	4	0	0	0	0	0	0	0	0	0	0	0	7
OTU1220	0	0	0	0	0	0	0	0	0	0	0	0	0	0	0	0	0	0	0	0	7	0	0	0	0	0	0	0	0	0	7
OTU1221	3	0	0	0	0	0	0	0	0	0	0	0	0	0	0	0	0	2	0	0	0	2	0	0	0	0	0	0	0	0	7
OTU1222	1	0	0	0	0	0	0	0	0	0	0	0	0	0	0	0	0	0	0	0	0	2	2	2	0	0	0	0	0	0	7
OTU1223	0	0	0	0	0	0	0	0	1	0	0	0	2	0	0	0	0	0	1	0	0	0	2	1	0	0	0	0	0	0	7
OTU1224	0	0	0	0	0	0	0	0	0	0	0	0	0	0	0	0	0	0	0	0	0	0	0	6	1	0	0	0	0	0	7
OTU1225	0	0	0	0	0	0	0	0	0	0	0	0	0	0	0	0	0	0	0	0	0	0	0	7	0	0	0	0	0	0	7
OTU1226	0	1	0	0	0	0	0	0	0	0	0	0	0	1	0	0	0	0	1	0	0	0	0	1	3	0	0	0	0	0	7
OTU1227	3	1	0	1	0	0	0	0	0	0	0	0	0	0	0	0	0	0	0	0	0	0	0	1	0	0	0	0	0	1	7
OTU1228	0	0	0	0	0	0	0	0	0	0	0	0	0	0	0	0	0	0	0	0	0	0	0	0	0	0	7	0	0	0	7
OTU1229	0	0	0	0	0	0	0	0	2	0	0	1	0	0	0	0	0	0	0	0	0	1	0	0	0	0	2	1	0	0	7
OTU1230	0	0	0	0	2	0	0	0	0	0	0	0	0	0	0	0	0	0	0	0	0	3	0	0	0	0	2	0	0	0	7
OTU1231	0	0	0	0	0	0	0	0	0	0	4	0	0	0	0	0	0	0	0	0	0	0	0	1	0	0	0	0	2	0	7
OTU1232	0	0	0	0	1	0	2	0	0	0	0	0	0	0	0	0	0	0	0	0	0	0	0	2	0	2	0	0	0	0	7
OTU1233	0	4	0	0	0	0	0	1	0	1	0	0	0	0	0	0	0	0	0	1	0	0	0	0	0	0	0	0	0	0	7
OTU1234	0	0	2	0	0	0	0	0	5	0	0	0	0	0	0	0	0	0	0	0	0	0	0	0	0	0	0	0	0	0	7
OTU1235	0	0	0	0	0	2	0	1	0	3	0	0	0	0	0	0	0	0	1	0	0	0	0	0	0	0	0	0	0	0	7
OTU1236	0	0	0	0	0	0	0	0	0	0	7	0	0	0	0	0	0	0	0	0	0	0	0	0	0	0	0	0	0	0	7
OTU1237	0	0	0	0	0	0	0	0	0	0	0	0	7	0	0	0	0	0	0	0	0	0	0	0	0	0	0	0	0	0	7

OTU1238	0	0	0	0	0	0	0	0	0	0	0	0	0	0	0	0	0	0	0	5	0	0	0	0	2	0	0	0	0	0	7
OTU1239	0	1	0	0	0	0	0	0	0	0	0	1	0	0	0	0	0	0	0	0	1	1	3	0	0	0	0	0	0	0	7
OTU1240	0	0	0	0	0	0	0	0	0	2	0	0	1	0	0	0	0	1	0	0	0	0	1	0	2	0	0	0	0	0	7
OTU1241	0	0	0	0	0	0	0	0	0	0	0	0	0	0	0	0	0	0	3	0	0	0	2	1	0	0	0	0	0	1	7
OTU1242	5	0	0	0	0	1	0	0	0	0	0	0	0	0	0	0	0	0	0	0	0	0	1	0	0	0	0	0	0	0	7
OTU1243	0	0	0	0	0	0	0	0	0	0	0	0	0	0	0	0	0	0	0	0	0	0	0	5	2	0	0	0	0	0	7
OTU1244	0	0	0	0	0	0	0	0	0	0	0	0	0	0	0	0	0	0	0	0	0	0	0	7	0	0	0	0	0	0	7
OTU1245	0	0	0	0	0	0	0	0	0	0	0	1	0	2	0	0	0	0	0	0	0	0	0	2	0	1	1	0	0	0	7
OTU1246	0	0	0	0	2	0	0	0	0	0	0	0	0	1	0	0	0	0	1	0	1	0	0	0	0	0	2	0	0	0	7
OTU1247	0	0	6	0	0	0	0	0	0	0	0	0	0	0	0	0	0	0	0	0	0	0	0	0	0	0	0	1	0	0	7
OTU1248	1	0	0	0	0	0	0	0	1	0	1	0	0	0	0	0	0	0	0	0	0	1	0	2	0	0	0	0	0	1	7
OTU1249	0	1	0	0	0	0	2	0	2	0	0	0	0	0	0	0	0	0	0	1	0	0	0	0	0	0	0	0	0	1	7
OTU1250	1	2	0	0	0	2	0	0	0	1	0	0	0	0	0	0	0	0	0	0	0	0	0	0	0	0	0	0	0	0	6
OTU1251	0	5	0	0	1	0	0	0	0	0	0	0	0	0	0	0	0	0	0	0	0	0	0	0	0	0	0	0	0	0	6
OTU1252	0	0	6	0	0	0	0	0	0	0	0	0	0	0	0	0	0	0	0	0	0	0	0	0	0	0	0	0	0	0	6
OTU1253	0	0	6	0	0	0	0	0	0	0	0	0	0	0	0	0	0	0	0	0	0	0	0	0	0	0	0	0	0	0	6
OTU1254	0	0	0	2	0	0	0	0	0	0	0	0	0	0	0	0	0	0	0	0	1	0	1	1	0	1	0	0	0	0	6
OTU1255	0	0	0	4	0	0	0	0	0	0	0	0	0	0	0	0	0	0	0	0	1	0	0	0	0	1	0	0	0	0	6
OTU1256	0	1	0	0	2	0	0	0	0	0	0	0	0	0	0	0	0	0	0	2	0	1	0	0	0	0	0	0	0	0	6
OTU1257	0	0	0	0	0	1	1	0	0	0	1	0	0	0	0	0	0	1	0	0	0	0	0	1	0	1	0	0	0	0	6
OTU1258	0	0	0	0	0	0	0	4	0	0	2	0	0	0	0	0	0	0	0	0	0	0	0	0	0	0	0	0	0	0	6
OTU1259	0	0	0	0	0	0	0	0	4	0	0	0	2	0	0	0	0	0	0	0	0	0	0	0	0	0	0	0	0	0	6
OTU1260	0	0	0	0	0	0	0	0	2	0	0	0	0	0	0	0	0	4	0	0	0	0	0	0	0	0	0	0	0	0	6
OTU1261	0	0	0	0	0	0	0	0	6	0	0	0	0	0	0	0	0	0	0	0	0	0	0	0	0	0	0	0	0	0	6
OTU1262	0	0	0	0	0	0	0	0	6	0	0	0	0	0	0	0	0	0	0	0	0	0	0	0	0	0	0	0	0	0	6
OTU1263	0	0	0	0	0	0	0	0	0	0	3	0	0	0	0	0	0	3	0	0	0	0	0	0	0	0	0	0	0	0	6

OTU1264	0	0	0	0	0	0	0	0	0	0	3	0	3	0	0	0	0	0	0	0	0	0	0	0	0	0	0	0	0	0	6
OTU1265	0	0	0	0	0	0	0	0	0	0	2	0	0	0	3	0	0	0	0	0	0	0	0	1	0	0	0	0	0	0	6
OTU1266	0	0	0	0	0	0	0	0	0	0	5	0	0	0	0	1	0	0	0	0	0	0	0	0	0	0	0	0	0	0	6
OTU1267	1	0	0	0	0	0	0	0	0	2	2	0	0	0	0	0	1	0	0	0	0	0	0	0	0	0	0	0	0	0	6
OTU1268	0	0	0	0	0	0	0	0	0	0	0	0	0	0	0	0	0	6	0	0	0	0	0	0	0	0	0	0	0	0	6
OTU1269	0	0	0	0	0	0	0	0	0	0	0	0	0	0	0	0	0	6	0	0	0	0	0	0	0	0	0	0	0	0	6
OTU1270	0	0	0	0	0	0	0	0	0	0	0	0	0	0	0	0	0	5	1	0	0	0	0	0	0	0	0	0	0	0	6
OTU1271	0	0	0	0	0	0	0	0	0	0	0	0	0	0	0	0	0	0	3	0	0	0	0	0	0	0	3	0	0	0	6
OTU1272	0	0	0	0	0	0	0	0	0	0	0	0	0	4	0	0	0	0	1	0	0	0	1	0	0	0	0	0	0	0	6
OTU1273	0	0	0	0	0	0	0	0	0	0	0	0	0	0	0	0	0	0	0	0	6	0	0	0	0	0	0	0	0	0	6
OTU1274	0	0	0	0	0	0	0	0	0	0	0	0	0	0	0	0	0	0	1	1	0	2	0	2	0	0	0	0	0	0	6
OTU1275	0	0	0	0	0	0	0	0	0	0	0	0	0	2	0	0	0	0	0	0	0	0	3	1	0	0	0	0	0	0	6
OTU1276	0	4	0	0	0	0	0	0	0	0	0	0	0	0	0	0	0	0	0	0	0	0	0	0	2	0	0	0	0	0	6
OTU1277	0	0	0	0	0	0	0	0	0	0	0	0	0	0	0	0	0	0	0	0	0	2	0	0	1	0	0	3	0	0	6
OTU1278	0	0	0	0	0	0	0	0	0	1	0	0	0	0	0	0	0	0	0	0	0	0	0	0	0	0	0	2	0	3	6
OTU1279	0	2	0	0	0	0	0	0	0	0	0	0	0	1	0	0	0	0	0	0	0	0	1	0	0	0	0	0	0	2	6
OTU1280	0	0	0	0	0	0	0	0	0	0	0	0	0	0	0	0	0	1	0	0	0	0	0	0	0	0	0	0	1	4	6
OTU1281	0	0	0	0	0	0	0	0	0	0	0	0	0	0	0	0	0	0	0	0	0	1	0	0	1	0	3	0	0	1	6
OTU1282	0	0	0	0	0	0	0	0	0	0	0	1	0	0	0	0	0	0	0	0	0	0	0	0	0	0	0	0	0	5	6
OTU1283	3	0	1	0	0	0	0	0	0	0	0	1	0	0	0	0	0	0	1	0	0	0	0	0	0	0	0	0	0	0	6
OTU1284	5	1	0	0	0	0	0	0	0	0	0	0	0	0	0	0	0	0	0	0	0	0	0	0	0	0	0	0	0	0	6
OTU1285	5	1	0	0	0	0	0	0	0	0	0	0	0	0	0	0	0	0	0	0	0	0	0	0	0	0	0	0	0	0	6
OTU1286	3	0	0	0	0	0	1	0	0	0	0	1	0	0	0	1	0	0	0	0	0	0	0	0	0	0	0	0	0	0	6
OTU1287	0	0	6	0	0	0	0	0	0	0	0	0	0	0	0	0	0	0	0	0	0	0	0	0	0	0	0	0	0	0	6
OTU1288	0	0	6	0	0	0	0	0	0	0	0	0	0	0	0	0	0	0	0	0	0	0	0	0	0	0	0	0	0	0	6
OTU1289	0	0	0	0	0	0	1	0	0	1	1	0	2	0	0	0	0	1	0	0	0	0	0	0	0	0	0	0	0	0	6

OTU1290	0	2	0	0	0	2	1	0	0	1	0	0	0	0	0	0	0	0	0	0	0	0	0	0	0	0	0	0	0	0	6
OTU1291	0	1	1	0	0	0	0	0	0	2	0	0	0	0	2	0	0	0	0	0	0	0	0	0	0	0	0	0	0	0	6
OTU1292	0	0	0	0	0	4	0	0	0	1	1	0	0	0	0	0	0	0	0	0	0	0	0	0	0	0	0	0	0	0	6
OTU1293	2	0	0	0	0	0	0	0	0	0	0	0	4	0	0	0	0	0	0	0	0	0	0	0	0	0	0	0	0	0	6
OTU1294	0	0	0	0	0	0	0	0	0	0	0	0	0	5	0	0	0	0	0	0	0	0	0	0	0	0	1	0	0	0	6
OTU1295	0	0	0	0	0	0	0	0	0	0	0	0	0	3	0	0	2	0	0	0	0	0	0	0	0	0	0	0	0	1	6
OTU1296	0	0	0	0	0	0	0	0	0	0	0	0	1	0	0	0	0	3	0	0	0	0	0	0	2	0	0	0	0	0	6
OTU1297	0	0	0	0	0	0	0	0	0	0	0	0	0	0	0	0	0	0	0	0	0	1	2	0	0	1	0	2	0	0	6
OTU1298	3	0	0	0	0	0	0	0	0	0	0	0	0	0	0	0	0	0	0	0	0	0	2	0	0	1	0	0	0	0	6
OTU1299	0	3	1	0	1	0	0	0	0	0	0	0	0	0	0	0	0	0	0	0	0	0	1	0	0	0	0	0	0	0	6
OTU1300	0	0	0	0	0	0	0	0	0	0	0	0	0	0	0	0	0	0	0	0	0	0	0	6	0	0	0	0	0	0	6
OTU1301	0	0	0	0	0	0	0	0	0	0	0	0	0	0	0	0	0	1	0	0	0	0	0	5	0	0	0	0	0	0	6
OTU1302	0	0	0	0	0	0	0	0	0	0	0	0	0	0	0	1	0	4	0	0	0	0	0	0	1	0	0	0	0	0	6
OTU1303	0	0	0	0	0	0	0	0	0	0	0	0	0	0	0	0	0	0	2	0	0	0	0	0	0	3	0	0	0	1	6
OTU1304	0	0	0	0	0	0	0	0	0	0	0	0	0	1	0	0	0	0	0	0	2	0	0	0	0	0	0	1	0	2	6
OTU1305	2	0	0	0	0	0	0	0	0	0	0	0	0	0	0	0	0	0	0	0	0	0	0	0	0	0	0	0	0	4	6
OTU1306	0	0	0	0	0	0	0	0	0	0	0	0	0	0	0	0	0	0	0	0	0	0	0	3	0	0	0	0	0	3	6
OTU1307	3	0	0	0	0	1	0	0	0	0	0	0	0	0	0	0	0	0	0	0	0	0	0	0	0	0	0	0	1	0	5
OTU1308	1	0	3	0	0	0	0	0	0	0	0	0	0	1	0	0	0	0	0	0	0	0	0	0	0	0	0	0	0	0	5
OTU1309	0	0	0	0	5	0	0	0	0	0	0	0	0	0	0	0	0	0	0	0	0	0	0	0	0	0	0	0	0	0	5
OTU1310	0	0	2	0	3	0	0	0	0	0	0	0	0	0	0	0	0	0	0	0	0	0	0	0	0	0	0	0	0	0	5
OTU1311	0	0	0	0	5	0	0	0	0	0	0	0	0	0	0	0	0	0	0	0	0	0	0	0	0	0	0	0	0	0	5
OTU1312	0	0	0	0	4	0	0	0	0	0	0	0	0	0	0	0	0	0	1	0	0	0	0	0	0	0	0	0	0	0	5
OTU1313	0	0	0	0	1	1	0	0	0	0	0	0	0	3	0	0	0	0	0	0	0	0	0	0	0	0	0	0	0	0	5
OTU1314	0	0	1	0	0	0	1	0	0	3	0	0	0	0	0	0	0	0	0	0	0	0	0	0	0	0	0	0	0	0	5
OTU1315	0	0	0	0	0	0	0	5	0	0	0	0	0	0	0	0	0	0	0	0	0	0	0	0	0	0	0	0	0	0	5

OTU1316	0	0	0	0	0	0	0	2	3	0	0	0	0	0	0	0	0	0	0	0	0	0	0	0	0	0	0	0	0	0	5
OTU1317	0	0	0	0	0	0	0	0	0	5	0	0	0	0	0	0	0	0	0	0	0	0	0	0	0	0	0	0	0	0	5
OTU1318	0	0	0	0	0	0	0	0	0	3	0	0	0	0	0	0	0	0	0	0	0	0	0	0	0	0	0	0	1	1	5
OTU1319	0	0	0	0	0	0	0	0	0	5	0	0	0	0	0	0	0	0	0	0	0	0	0	0	0	0	0	0	0	0	5
OTU1320	0	0	0	0	0	0	0	0	0	3	0	1	0	0	0	0	0	0	0	0	0	1	0	0	0	0	0	0	0	0	5
OTU1321	0	0	0	0	0	0	0	0	0	5	0	0	0	0	0	0	0	0	0	0	0	0	0	0	0	0	0	0	0	0	5
OTU1322	0	0	0	0	0	0	0	0	0	5	0	0	0	0	0	0	0	0	0	0	0	0	0	0	0	0	0	0	0	0	5
OTU1323	0	0	0	0	0	0	0	0	0	0	0	2	0	0	0	0	0	2	0	0	0	0	0	1	0	0	0	0	0	0	5
OTU1324	0	0	0	0	0	0	0	0	0	0	0	0	5	0	0	0	0	0	0	0	0	0	0	0	0	0	0	0	0	0	5
OTU1325	0	0	0	0	0	0	0	0	0	0	0	0	5	0	0	0	0	0	0	0	0	0	0	0	0	0	0	0	0	0	5
OTU1326	0	0	1	0	0	1	0	0	0	0	0	0	0	1	0	0	0	0	0	0	0	0	0	2	0	0	0	0	0	0	5
OTU1327	0	0	0	0	0	0	0	0	0	0	0	0	0	2	0	0	0	0	1	0	0	2	0	0	0	0	0	0	0	0	5
OTU1328	0	1	0	0	0	0	1	1	0	0	0	0	0	0	1	0	0	1	0	0	0	0	0	0	0	0	0	0	0	0	5
OTU1329	0	0	0	0	0	0	0	0	0	0	0	0	0	0	0	2	0	0	0	0	0	0	0	3	0	0	0	0	0	0	5
OTU1330	0	0	0	0	0	0	0	0	0	1	0	0	3	0	0	0	0	1	0	0	0	0	0	0	0	0	0	0	0	0	5
OTU1331	0	0	0	0	0	0	0	0	0	2	0	0	0	0	0	0	0	3	0	0	0	0	0	0	0	0	0	0	0	0	5
OTU1332	0	0	0	0	1	0	0	0	0	0	0	0	0	0	0	0	0	1	0	0	0	0	0	0	0	1	0	1	0	1	5
OTU1333	0	0	0	0	0	0	0	0	0	0	0	0	0	0	1	0	0	4	0	0	0	0	0	0	0	0	0	0	0	0	5
OTU1334	0	0	0	0	0	0	0	0	0	0	0	0	0	0	0	0	0	1	4	0	0	0	0	0	0	0	0	0	0	0	5
OTU1335	0	0	0	0	0	0	0	0	0	0	0	0	0	0	0	0	0	0	5	0	0	0	0	0	0	0	0	0	0	0	5
OTU1336	0	0	0	0	0	0	0	0	0	0	0	0	0	0	0	0	0	0	0	5	0	0	0	0	0	0	0	0	0	0	5
OTU1337	0	0	0	0	0	0	0	0	0	0	0	0	0	0	0	0	0	0	0	0	2	0	0	0	0	3	0	0	0	0	5
OTU1338	0	0	0	0	0	0	0	0	0	0	0	0	0	0	0	0	0	0	0	0	4	0	0	0	0	0	1	0	0	0	5
OTU1339	0	0	1	0	0	0	0	0	0	0	0	0	0	0	0	0	0	0	0	0	0	1	2	1	0	0	0	0	0	0	5
OTU1340	0	0	0	0	0	0	0	0	0	0	0	0	0	0	0	0	0	0	0	0	0	0	5	0	0	0	0	0	0	0	5
OTU1341	0	0	0	0	0	0	0	0	0	0	0	0	0	0	0	0	0	0	0	0	0	0	1	4	0	0	0	0	0	0	5

OTU1342	0	0	0	0	0	0	0	0	0	0	0	0	0	0	0	0	0	0	0	0	0	0	0	5	0	0	0	0	0	0	5
OTU1343	0	0	0	0	0	0	0	0	0	0	0	0	0	0	0	0	0	0	0	0	0	0	0	1	0	0	0	0	4	0	5
OTU1344	0	0	0	0	0	0	0	0	0	0	0	0	0	0	0	0	0	0	0	0	0	0	0	5	0	0	0	0	0	0	5
OTU1345	0	0	0	0	0	0	0	0	0	0	0	0	0	0	0	0	0	0	0	0	0	0	0	5	0	0	0	0	0	0	5
OTU1346	0	0	0	0	0	0	0	0	0	0	0	0	0	0	0	0	0	0	0	0	0	0	0	0	4	1	0	0	0	0	5
OTU1347	0	0	0	0	0	0	0	0	0	0	0	0	0	1	0	0	1	0	0	0	0	0	0	0	3	0	0	0	0	0	5
OTU1348	0	0	0	0	0	0	0	0	0	0	0	0	0	0	0	0	0	0	0	1	0	0	0	0	3	0	1	0	0	0	5
OTU1349	0	0	0	0	0	0	0	0	0	0	0	0	0	0	0	0	0	0	0	1	0	0	0	0	4	0	0	0	0	0	5
OTU1350	0	0	0	0	0	0	0	0	0	0	0	0	0	0	0	0	0	0	0	0	0	0	0	1	4	0	0	0	0	0	5
OTU1351	0	0	0	0	0	0	0	0	0	0	0	0	0	0	0	0	0	0	0	0	0	0	0	0	0	5	0	0	0	0	5
OTU1352	0	0	0	0	0	0	0	0	0	0	0	0	0	3	0	0	0	0	0	0	0	0	0	0	0	0	2	0	0	0	5
OTU1353	0	0	0	0	0	0	0	0	0	0	0	0	0	0	0	0	0	0	0	3	0	0	0	0	0	0	0	2	0	0	5
OTU1354	0	0	0	0	0	0	0	0	0	0	0	0	0	0	0	0	0	0	0	0	0	0	0	0	0	0	0	5	0	0	5
OTU1355	0	0	0	0	0	0	0	0	0	0	0	0	0	0	0	0	0	0	0	0	0	0	0	0	0	0	0	0	5	0	5
OTU1356	0	0	0	0	1	0	0	0	0	0	0	0	0	0	0	0	0	0	0	0	0	0	1	0	0	0	0	0	0	3	5
OTU1357	0	0	0	0	0	0	0	0	0	0	0	0	0	0	0	0	0	0	0	0	0	0	0	0	0	0	0	0	0	5	5
OTU1358	0	0	0	0	0	0	0	0	0	0	0	0	0	0	0	0	0	0	0	0	0	0	1	3	0	0	0	0	0	1	5
OTU1359	0	0	0	0	0	0	0	0	0	0	0	0	0	0	0	0	0	0	4	0	0	0	0	0	0	0	0	0	0	1	5
OTU1360	1	1	0	2	0	0	0	0	0	0	0	0	0	0	0	0	0	0	1	0	0	0	0	0	0	0	0	0	0	0	5
OTU1361	5	0	0	0	0	0	0	0	0	0	0	0	0	0	0	0	0	0	0	0	0	0	0	0	0	0	0	0	0	0	5
OTU1362	0	0	1	0	0	0	0	0	1	0	0	0	0	0	0	0	0	1	0	0	0	0	1	1	0	0	0	0	0	0	5
OTU1363	1	0	0	4	0	0	0	0	0	0	0	0	0	0	0	0	0	0	0	0	0	0	0	0	0	0	0	0	0	0	5
OTU1364	0	3	0	0	0	0	1	1	0	0	0	0	0	0	0	0	0	0	0	0	0	0	0	0	0	0	0	0	0	0	5
OTU1365	0	0	0	0	0	0	2	2	0	1	0	0	0	0	0	0	0	0	0	0	0	0	0	0	0	0	0	0	0	0	5
OTU1366	1	0	0	0	0	0	0	0	0	0	0	0	0	4	0	0	0	0	0	0	0	0	0	0	0	0	0	0	0	0	5
OTU1367	0	0	0	0	0	0	0	0	0	0	0	0	0	3	0	2	0	0	0	0	0	0	0	0	0	0	0	0	0	0	5

OTU1368	1	0	0	0	0	0	0	1	0	0	0	0	0	1	0	0	0	0	0	0	0	2	0	0	0	0	0	0	0	0	5
OTU1369	3	0	0	0	0	0	0	0	0	0	0	0	0	1	0	0	0	0	0	0	0	0	1	0	0	0	0	0	0	0	5
OTU1370	0	0	0	0	0	0	0	0	0	0	0	0	0	0	0	0	1	0	0	3	0	1	0	0	0	0	0	0	0	0	5
OTU1371	0	0	0	0	0	0	0	0	0	0	0	0	0	0	0	0	0	5	0	0	0	0	0	0	0	0	0	0	0	0	5
OTU1372	0	0	0	0	0	0	0	0	0	0	0	0	0	0	0	0	0	5	0	0	0	0	0	0	0	0	0	0	0	0	5
OTU1373	0	0	0	0	0	0	3	0	0	0	0	0	0	0	0	0	0	2	0	0	0	0	0	0	0	0	0	0	0	0	5
OTU1374	0	0	0	0	0	0	0	0	0	0	0	0	0	0	0	0	0	5	0	0	0	0	0	0	0	0	0	0	0	0	5
OTU1375	0	0	0	0	0	0	0	0	0	0	0	0	0	0	0	0	0	3	2	0	0	0	0	0	0	0	0	0	0	0	5
OTU1376	0	0	0	0	0	0	0	0	0	0	0	0	0	0	0	0	0	4	1	0	0	0	0	0	0	0	0	0	0	0	5
OTU1377	0	0	1	0	0	0	0	0	0	3	0	0	0	0	0	0	0	1	0	0	0	0	0	0	0	0	0	0	0	0	5
OTU1378	0	0	0	0	0	0	0	0	0	0	0	0	0	0	0	0	0	0	0	0	4	0	0	0	0	1	0	0	0	0	5
OTU1379	0	0	0	0	0	0	0	0	0	0	0	0	0	0	0	0	0	0	0	0	3	0	0	1	0	0	1	0	0	0	5
OTU1380	0	0	0	0	0	0	0	0	0	0	0	0	0	2	0	0	0	1	0	0	0	0	1	0	0	0	0	0	0	1	5
OTU1381	0	0	0	0	0	0	0	0	0	0	0	0	0	0	0	0	0	0	0	0	0	0	0	4	1	0	0	0	0	0	5
OTU1382	0	0	0	0	0	0	0	0	0	0	0	0	0	0	0	0	0	0	0	0	0	0	0	5	0	0	0	0	0	0	5
OTU1383	0	0	0	0	0	0	0	0	0	0	0	1	0	1	0	0	0	0	0	0	0	0	0	2	0	0	0	1	0	0	5
OTU1384	0	0	0	0	0	0	0	0	0	0	0	0	0	0	0	0	0	0	0	0	0	0	4	0	0	1	0	0	0	0	5
OTU1385	0	0	0	0	0	0	0	0	0	0	0	0	0	0	0	0	0	0	1	0	0	0	0	0	0	1	0	0	1	2	5
OTU1386	0	0	0	0	0	0	0	0	0	0	0	0	0	0	0	0	0	0	0	0	0	0	0	2	0	0	2	0	1	0	5
OTU1387	1	0	0	0	0	0	0	0	0	0	0	0	0	0	0	0	0	0	0	0	2	0	1	0	0	0	1	0	0	0	5
OTU1388	0	0	0	0	0	0	0	0	0	0	0	0	0	0	0	0	0	0	0	0	0	0	0	0	0	0	1	0	0	4	5
OTU1389	0	0	0	0	0	0	0	0	0	0	0	0	0	0	0	0	0	0	0	0	0	0	0	0	0	0	0	0	0	5	5
OTU1390	0	0	0	0	0	0	0	0	0	0	0	0	0	0	0	0	0	0	0	0	0	0	0	0	0	0	0	1	0	4	5
OTU1391	0	0	0	0	0	0	0	0	0	0	0	0	0	0	0	0	0	0	0	0	0	1	0	2	0	0	0	0	0	2	5

Supplementary Table S3. Information of OTUs annotation.

OTUs	Reference sequence	Kindom	Phylum	Class	Order	Family	Genus	E-value	Bitscore	Length	Similarity	Gapopen	Gaps
OTU162	JX014309	Fungi	Ascomycota	Dothideomycetes	Capnodiales	Capnodiaceae	*Capnodium*	2E-84	313	174	98.28	1	1
OTU277	JF817339	Fungi	Ascomycota	Dothideomycetes	Capnodiales	Capnodiaceae	*Capnodium*	8E-61	234	134	97.76	1	1
OTU281	KM282290	Fungi	Ascomycota	Dothideomycetes	Capnodiales	Capnodiaceae	unidentified	4E-82	305	181	97.24	2	3
OTU463	KJ463541	Fungi	Ascomycota	Dothideomycetes	Capnodiales	Capnodiaceae	unidentified	8E-96	351	189	98.94	1	1
OTU499	JF495181	Fungi	Ascomycota	Dothideomycetes	Capnodiales	Capnodiaceae	unidentified	5E-56	218	138	95.65	1	1
OTU152	GU981736	Fungi	Ascomycota	Dothideomycetes	Capnodiales	Capnodiaceae	unidentified	3E-95	349	215	96.74	3	4
OTU258	GQ999538	Fungi	Ascomycota	Dothideomycetes	Capnodiales	Capnodiaceae	unidentified	2E-106	387	252	95.63	4	7
OTU508	LC011422	Fungi	Ascomycota	Dothideomycetes	Capnodiales	Cladosporiaceae	*Cladosporium*	7E-93	341	184	98.91	1	1
OTU35	DQ914681	Fungi	Ascomycota	Dothideomycetes	Capnodiales	Cladosporiaceae	*Cladosporium*	6E-53	208	157	92.36	1	1
OTU320	KF146177	Fungi	Ascomycota	Dothideomycetes	Capnodiales	Cladosporiaceae	*Cladosporium*	1E-103	377	198	98.99	0	0
OTU275	AJ582964	Fungi	Ascomycota	Dothideomycetes	Capnodiales	Cladosporiaceae	*Cladosporium*	6E-100	365	226	96.46	3	5
OTU542	FJ757587	Fungi	Ascomycota	Dothideomycetes	Capnodiales	Cladosporiaceae	*Cladosporium*	2E-93	343	197	98.48	3	3
OTU398	KM265497	Fungi	Ascomycota	Dothideomycetes	Capnodiales	Cladosporiaceae	*Cladosporium*	2E-64	246	140	98.57	2	2
OTU446	KC109759	Fungi	Ascomycota	Dothideomycetes	Capnodiales	Cladosporiaceae	*Cladosporium*	3E-76	285	163	98.16	2	3
OTU443	EU035415	Fungi	Ascomycota	Dothideomycetes	Capnodiales	Cladosporiaceae	*Cladosporium*	4E-48	192	197	88.32	3	7
OTU503	KJ817302	Fungi	Ascomycota	Dothideomycetes	Capnodiales	Cladosporiaceae	*Cladosporium*	3E-133	476	252	99.21	1	1
OTU65	JX014309	Fungi	Ascomycota	Dothideomycetes	Capnodiales	Davidiellaceae	*Davidiella*	3E-92	339	226	95.13	3	4
OTU361	EU041803	Fungi	Ascomycota	Dothideomycetes	Capnodiales	Dissoconiaceae	*Uwebraunia*	3E-33	143	151	91.39	7	8
OTU322	KM111293	Fungi	Ascomycota	Dothideomycetes	Capnodiales	Mycosphaerellaceae	*Cercospora*	7E-96	351	181	99.45	0	0
OTU76	KF800162	Fungi	Ascomycota	Dothideomycetes	Capnodiales	Mycosphaerellaceae	*Mycosphaerella*	5E-122	438	240	98.33	1	2
OTU254	DQ530216	Fungi	Ascomycota	Dothideomycetes	Capnodiales	Mycosphaerellaceae	*Mycosphaerella*	1E-44	180	147	92.52	4	8
OTU351	DQ530216	Fungi	Ascomycota	Dothideomycetes	Capnodiales	Mycosphaerellaceae	*Mycosphaerella*	4E-35	149	140	90.71	4	7
OTU465	NR_073218	Fungi	Ascomycota	Dothideomycetes	Capnodiales	Mycosphaerellaceae	*Mycosphaerella*	2E-51	202	110	98.18	0	0

OTU317	HQ625466	Fungi	Ascomycota	Dothideomycetes	Capnodiales	Mycosphaerellaceae	*Mycosphaerella*	5E-69	262	172	96.51	4	4
OTU371	HM992800	Fungi	Ascomycota	Dothideomycetes	Capnodiales	Mycosphaerellaceae	*Mycosphaerella*	9E-93	341	227	95.59	4	5
OTU66	JX875930	Fungi	Ascomycota	Dothideomycetes	Capnodiales	Mycosphaerellaceae	*Mycosphaerella*	1E-75	283	143	100	0	0
OTU505	KM113749	Fungi	Ascomycota	Dothideomycetes	Capnodiales	Mycosphaerellaceae	*Mycosphaerella*	5E-78	291	155	98.71	0	0
OTU130	DQ914681	Fungi	Ascomycota	Dothideomycetes	Capnodiales	Mycosphaerellaceae	*Mycosphaerella*	1E-57	224	157	93.63	1	1
OTU184	GQ203545	Fungi	Ascomycota	Dothideomycetes	Capnodiales	Mycosphaerellaceae	*Mycosphaerella*	3E-95	349	204	97.06	1	1
OTU344	JX998717	Fungi	Ascomycota	Dothideomycetes	Capnodiales	Mycosphaerellaceae	*Mycosphaerella*	1E-66	254	151	97.35	2	3
OTU510	AB255305	Fungi	Ascomycota	Dothideomycetes	Capnodiales	Mycosphaerellaceae	*Mycosphaerella*	2E-55	216	125	97.6	1	1
OTU318	HQ649891	Fungi	Ascomycota	Dothideomycetes	Capnodiales	Mycosphaerellaceae	*Mycosphaerella*	7E-93	341	176	99.43	0	0
OTU37	AY604831	Fungi	Ascomycota	Dothideomycetes	Capnodiales	Mycosphaerellaceae	*Mycosphaerella*	6E-62	238	155	94.84	1	2
OTU349	KM217176	Fungi	Ascomycota	Dothideomycetes	Capnodiales	Mycosphaerellaceae	*Mycosphaerella*	6E-52	204	114	98.25	1	2
OTU90	KF146177	Fungi	Ascomycota	Dothideomycetes	Capnodiales	Mycosphaerellaceae	*Mycosphaerella*	8E-102	371	199	98.49	0	0
OTU545	GU981736	Fungi	Ascomycota	Dothideomycetes	Capnodiales	Mycosphaerellaceae	*Mycosphaerella*	5E-97	355	215	97.21	3	3
OTU350	AF078889	Fungi	Ascomycota	Dothideomycetes	Capnodiales	Mycosphaerellaceae	*Mycosphaerella*	6E-87	321	186	98.39	3	3
OTU121	KC978007	Fungi	Ascomycota	Dothideomycetes	Capnodiales	Mycosphaerellaceae	*Mycosphaerella*	8E-87	321	220	95.45	5	7
OTU507	KP131523	Fungi	Ascomycota	Dothideomycetes	Capnodiales	Mycosphaerellaceae	*Passalora*	7E-71	268	151	98.01	1	1
OTU268	AB998373	Fungi	Ascomycota	Dothideomycetes	Capnodiales	Mycosphaerellaceae	*Passalora*	3E-89	329	181	98.34	1	2
OTU129	GU981736	Fungi	Ascomycota	Dothideomycetes	Capnodiales	Mycosphaerellaceae	*Pseudocercospora*	1E-103	377	210	98.1	1	1
OTU280	KJ869117	Fungi	Ascomycota	Dothideomycetes	Capnodiales	Mycosphaerellaceae	*Pseudocercosporella*	9E-64	244	139	97.84	1	1
OTU553	KM051384	Fungi	Ascomycota	Dothideomycetes	Capnodiales	Mycosphaerellaceae	*Ramichloridium*	5E-69	262	168	97.02	4	4
OTU186	HQ630983	Fungi	Ascomycota	Dothideomycetes	Capnodiales	Mycosphaerellaceae	*Ramichloridium*	3E-57	222	143	96.5	3	4
OTU261	KC485065	Fungi	Ascomycota	Dothideomycetes	Capnodiales	Mycosphaerellaceae	*Ramichloridium*	1E-57	224	179	92.18	4	10
OTU435	KC305358	Fungi	Ascomycota	Dothideomycetes	Capnodiales	Mycosphaerellaceae	*Ramichloridium*	9E-55	214	163	94.48	5	6
OTU82	HM992800	Fungi	Ascomycota	Dothideomycetes	Capnodiales	Mycosphaerellaceae	*Ramichloridium*	2E-118	426	223	99.1	0	0
OTU492	FJ627262	Fungi	Ascomycota	Dothideomycetes	Capnodiales	Mycosphaerellaceae	*Ramichloridium*	2E-68	260	209	91.87	4	10
OTU326	UDB019589	Fungi	Ascomycota	Dothideomycetes	Capnodiales	Mycosphaerellaceae	*Ramichloridium*	8E-121	434	243	98.77	3	3

OTU299	HM992800	Fungi	Ascomycota	Dothideomycetes	Capnodiales	Mycosphaerellaceae	*Ramichloridium*	9E-93	341	227	95.15	3	4
OTU138	FJ820729	Fungi	Ascomycota	Dothideomycetes	Capnodiales	Mycosphaerellaceae	*Ramichloridium*	3E-64	246	166	95.18	3	5
OTU543	GQ999538	Fungi	Ascomycota	Dothideomycetes	Capnodiales	Mycosphaerellaceae	*Septoria*	7E-38	159	80	100	0	0
OTU478	KM051384	Fungi	Ascomycota	Dothideomycetes	Capnodiales	Mycosphaerellaceae	unidentified	2E-74	280	153	98.69	1	1
OTU255	EU035415	Fungi	Ascomycota	Dothideomycetes	Capnodiales	Mycosphaerellaceae	unidentified	1E-38	161	201	88.06	7	11
OTU303	AJ582964	Fungi	Ascomycota	Dothideomycetes	Capnodiales	Mycosphaerellaceae	unidentified	3E-111	402	223	97.76	0	0
OTU257	KF286985	Fungi	Ascomycota	Dothideomycetes	Capnodiales	Mycosphaerellaceae	unidentified	3E-30	133	127	91.34	4	4
OTU535	DQ530216	Fungi	Ascomycota	Dothideomycetes	Capnodiales	Mycosphaerellaceae	unidentified	1E-35	151	128	92.19	3	3
OTU462	KJ186791	Fungi	Ascomycota	Dothideomycetes	Capnodiales	Teratosphaeriaceae	*Devriesia*	3E-73	276	139	100	0	0
OTU428	KP131662	Fungi	Ascomycota	Dothideomycetes	Capnodiales	Teratosphaeriaceae	*Devriesia*	4E-69	262	140	99.29	1	1
OTU413	AB769904	Fungi	Ascomycota	Dothideomycetes	Capnodiales	Teratosphaeriaceae	unidentified	3E-92	339	236	94.49	4	7
OTU749	KC438382	Fungi	Ascomycota	Dothideomycetes	Capnodiales	Teratosphaeriaceae	unidentified	1E-65	250	141	97.87	1	2
OTU431	GU981736	Fungi	Ascomycota	Dothideomycetes	Capnodiales	Teratosphaeriaceae	unidentified	2E-90	333	217	95.85	4	7
OTU502	KC857282	Fungi	Ascomycota	Dothideomycetes	Capnodiales	Teratosphaeriaceae	unidentified	4E-63	242	155	95.48	2	5
OTU518	FJ791130	Fungi	Ascomycota	Dothideomycetes	Capnodiales	Teratosphaeriaceae	unidentified	7E-93	341	180	99.44	1	1
OTU549	KC978007	Fungi	Ascomycota	Dothideomycetes	Capnodiales	Teratosphaeriaceae	unidentified	3E-83	309	220	93.64	2	2
OTU208	AB255241	Fungi	Ascomycota	Dothideomycetes	Capnodiales	Trichomeriaceae	*Trichomerium*	6E-62	238	136	97.79	1	1
OTU114	KC354795	Fungi	Ascomycota	Dothideomycetes	Capnodiales	Trichomeriaceae	*Trichomerium*	1E-56	220	155	94.19	2	2
OTU166	JF819170	Fungi	Ascomycota	Dothideomycetes	Capnodiales	Trichomeriaceae	*Trichomerium*	1E-59	230	156	94.23	1	1
OTU282	AB507841	Fungi	Ascomycota	Dothideomycetes	Capnodiales	Trichomeriaceae	*Trichomerium*	2E-38	161	117	94.87	3	3
OTU298	FJ778154	Fungi	Ascomycota	Dothideomycetes	Capnodiales	Trichomeriaceae	*Trichomerium*	4E-66	252	159	96.86	3	3
OTU321	KJ881377	Fungi	Ascomycota	Dothideomycetes	Capnodiales	Trichomeriaceae	*Trichomerium*	7E-90	331	189	97.35	1	3
OTU11	JX014309	Fungi	Ascomycota	Dothideomycetes	Capnodiales	Trichomeriaceae	*Trichomerium*	8E-121	434	223	99.55	0	0
OTU80	KM246756	Fungi	Ascomycota	Dothideomycetes	Capnodiales	Trichomeriaceae	*Trichomerium*	6E-81	301	176	96.59	0	0
OTU110	JX998717	Fungi	Ascomycota	Dothideomycetes	Capnodiales	Trichomeriaceae	*Trichomerium*	5E-56	218	152	95.39	4	6
OTU133	JQ825256	Fungi	Ascomycota	Dothideomycetes	Capnodiales	Trichomeriaceae	*Trichomerium*	2E-51	202	110	99.09	1	1

OTU30	FJ757039	Fungi	Ascomycota	Dothideomycetes	Capnodiales	Trichomeriaceae	*Trichomerium*	2E-86	319	161	100	0	0
OTU33	NR_073231	Fungi	Ascomycota	Dothideomycetes	Capnodiales	Trichomeriaceae	*Trichomerium*	6E-49	194	114	97.37	1	1
OTU159	KM527126	Fungi	Ascomycota	Dothideomycetes	Capnodiales	Trichomeriaceae	*Trichomerium*	6E-65	248	147	97.28	2	4
OTU400	AB615578	Fungi	Ascomycota	Dothideomycetes	Capnodiales	Trichomeriaceae	*Trichomerium*	7E-36	151	76	100	0	0
OTU63	KM246756	Fungi	Ascomycota	Dothideomycetes	Capnodiales	Trichomeriaceae	*Trichomerium*	1E-75	283	179	97.21	4	4
OTU117	KF286985	Fungi	Ascomycota	Dothideomycetes	Capnodiales	Trichomeriaceae	*Trichomerium*	5E-47	188	151	92.72	3	3
OTU287	JF749183	Fungi	Ascomycota	Dothideomycetes	Capnodiales	Trichomeriaceae	*Trichomerium*	1E-20	101	97	88.66	1	3
OTU434	JX268526	Fungi	Ascomycota	Dothideomycetes	Capnodiales	Trichomeriaceae	*Trichomerium*	1E-53	210	126	96.83	1	1
OTU544	GQ999538	Fungi	Ascomycota	Dothideomycetes	Capnodiales	Trichomeriaceae	*Trichomerium*	1E-45	184	101	98.02	0	0
OTU188	AB922855	Fungi	Ascomycota	Dothideomycetes	Capnodiales	Trichomeriaceae	*Trichomerium*	6E-49	194	110	98.18	1	1
OTU219	JQ905732	Fungi	Ascomycota	Dothideomycetes	Capnodiales	Trichomeriaceae	*Trichomerium*	1E-56	220	135	97.04	2	2
OTU267	AB507841	Fungi	Ascomycota	Dothideomycetes	Capnodiales	Trichomeriaceae	*Trichomerium*	8E-41	168	109	95.41	1	1
OTU331	JF817339	Fungi	Ascomycota	Dothideomycetes	Capnodiales	Trichomeriaceae	*Trichomerium*	8E-58	224	137	97.08	2	2
OTU61	JF945390	Fungi	Ascomycota	Dothideomycetes	Capnodiales	Trichomeriaceae	*Trichomerium*	7E-49	194	133	95.49	3	4
OTU98	KC857282	Fungi	Ascomycota	Dothideomycetes	Capnodiales	Trichomeriaceae	*Trichomerium*	7E-74	278	152	98.03	0	0
OTU96	GQ999538	Fungi	Ascomycota	Dothideomycetes	Capnodiales	Trichomeriaceae	*Trichomerium*	3E-43	176	105	97.14	1	1
OTU263	AB998515	Fungi	Ascomycota	Dothideomycetes	Capnodiales	Trichomeriaceae	*Trichomerium*	7E-58	224	124	98.39	1	2
OTU295	KJ775453	Fungi	Ascomycota	Dothideomycetes	Capnodiales	Trichomeriaceae	*Trichomerium*	5E-78	291	155	98.71	0	0
OTU54	JX966555	Fungi	Ascomycota	Dothideomycetes	Capnodiales	Trichomeriaceae	*Trichomerium*	2E-74	280	199	93.97	3	5
OTU218	GQ999538	Fungi	Ascomycota	Dothideomycetes	Capnodiales	Trichomeriaceae	*Trichomerium*	7E-38	159	80	100	0	0
OTU269	AM712269	Fungi	Ascomycota	Dothideomycetes	Capnodiales	unidentified	unidentified	4E-92	339	235	94.89	4	4
OTU429	U65613	Fungi	Ascomycota	Dothideomycetes	Capnodiales	unidentified	unidentified	8E-55	214	145	94.48	2	5
OTU472	HM484701	Fungi	Ascomycota	Dothideomycetes	Capnodiales	unidentified	unidentified	8E-83	307	155	100	0	0
OTU556	Z81427	Fungi	Ascomycota	Dothideomycetes	Capnodiales	unidentified	unidentified	6E-31	135	124	89.52	1	1
OTU363	FJ820729	Fungi	Ascomycota	Dothideomycetes	Capnodiales	unidentified	unidentified	8E-43	174	170	91.76	7	9
OTU224	AM901803	Fungi	Ascomycota	Dothideomycetes	Capnodiales	unidentified	unidentified	8E-55	214	148	95.27	3	3

OTU210	KM100712	Fungi	Ascomycota	Dothideomycetes	Capnodiales	unidentified	unidentified	4E-85	315	191	97.38	3	3
OTU375	KF901810	Fungi	Ascomycota	Dothideomycetes	Dothideales	Saccotheciaceae	*Aureobasidium*	5E-47	188	151	92.72	3	3
OTU89	FJ770078	Fungi	Ascomycota	Dothideomycetes	Dothideales	Saccotheciaceae	*Aureobasidium*	3E-23	109	142	88.03	5	6
OTU470	FJ778154	Fungi	Ascomycota	Dothideomycetes	Incertae	Eremomycetaceae	*Arthrographis*	4E-60	232	164	95.12	4	5
OTU83	GU981736	Fungi	Ascomycota	Dothideomycetes	Incertae	Incertae	*Zymoseptoria*	1E-91	337	214	96.73	4	4
OTU506	KM527126	Fungi	Ascomycota	Dothideomycetes	Incertae	Incertae	*Zymoseptoria*	3E-70	266	142	99.3	1	1
OTU139	JF817339	Fungi	Ascomycota	Dothideomycetes	Incertae	Incertae	*Zymoseptoria*	2E-67	256	133	99.25	0	0
OTU460	KC193597	Fungi	Ascomycota	Dothideomycetes	Incertae	Incertae	*Zymoseptoria*	6E-49	194	110	98.18	1	1
OTU496	FJ820729	Fungi	Ascomycota	Dothideomycetes	Incertae	Incertae	*Zymoseptoria*	1E-50	200	168	92.86	5	6
OTU447	KC491368	Fungi	Ascomycota	Dothideomycetes	Incertae	Incertae	*Zymoseptoria*	1E-113	410	242	96.69	1	2
OTU360	AY254161	Fungi	Ascomycota	Dothideomycetes	Myriangiales	Myriangiaceae	*Myriangium*	4E-23	109	103	90.29	2	2
OTU103	KM527126	Fungi	Ascomycota	Dothideomycetes	Myriangiales	Myriangiaceae	*Myriangium*	1E-62	240	145	97.24	2	2
OTU441	U65613	Fungi	Ascomycota	Dothideomycetes	Myriangiales	Myriangiaceae	*Myriangium*	6E-65	248	141	97.16	0	0
OTU201	GQ999538	Fungi	Ascomycota	Dothideomycetes	Pleosporales	Coniothyriaceae	*Coniothyrium*	5E-42	172	107	97.2	2	2
OTU751	KC857282	Fungi	Ascomycota	Dothideomycetes	Pleosporales	Cucurbitariaceae	*Pyrenochaetopsis*	2E-58	226	159	94.97	4	7
OTU204	KM225886	Fungi	Ascomycota	Dothideomycetes	Pleosporales	Cucurbitariaceae	*Pyrenochaetopsis*	1E-107	391	225	98.22	3	3
OTU259	JX014309	Fungi	Ascomycota	Dothideomycetes	Pleosporales	Didymellaceae	*Didymella*	1E-97	357	228	96.49	4	4
OTU356	KF146177	Fungi	Ascomycota	Dothideomycetes	Pleosporales	Didymellaceae	*Didymella*	5E-103	375	197	98.98	0	0
OTU306	GQ999538	Fungi	Ascomycota	Dothideomycetes	Pleosporales	Didymellaceae	*Didymella*	1E-119	430	249	97.59	2	2
OTU581	NR_073218	Fungi	Ascomycota	Dothideomycetes	Pleosporales	Didymellaceae	*Leptosphaerulina*	8E-39	161	105	94.29	0	0
OTU562	DQ825984	Fungi	Ascomycota	Dothideomycetes	Pleosporales	Didymosphaeriaceae	*Didymosphaeria*	2E-43	176	124	94.35	2	3
OTU16	KC354795	Fungi	Ascomycota	Dothideomycetes	Pleosporales	Didymosphaeriaceae	*Didymosphaeria*	1E-59	230	152	94.74	1	1
OTU42	FJ757768	Fungi	Ascomycota	Dothideomycetes	Pleosporales	Didymosphaeriaceae	*Didymosphaeria*	3E-82	305	166	98.8	1	1
OTU18	KM246232	Fungi	Ascomycota	Dothideomycetes	Pleosporales	Didymosphaeriaceae	*Didymosphaeria*	4E-44	178	122	95.08	2	2
OTU452	KM520128	Fungi	Ascomycota	Dothideomycetes	Pleosporales	Incertae	*Phoma*	7E-87	321	209	96.65	5	6
OTU783	HM487014	Fungi	Ascomycota	Dothideomycetes	Pleosporales	Incertae	*Phoma*	2E-37	157	145	92.41	6	8

OTU367	KC485065	Fungi	Ascomycota	Dothideomycetes	Pleosporales	Lentitheciaceae	*Keissleriella*	9E-83	307	171	98.83	2	2
OTU523	JQ692165	Fungi	Ascomycota	Dothideomycetes	Pleosporales	Leptosphaeriaceae	*Leptosphaeria*	8E-87	321	178	98.88	2	2
OTU164	GU053830	Fungi	Ascomycota	Dothideomycetes	Pleosporales	Leptosphaeriaceae	*Leptosphaeria*	2E-50	200	204	89.71	5	6
OTU466	AJ875349	Fungi	Ascomycota	Dothideomycetes	Pleosporales	Leptosphaeriaceae	*Leptosphaeria*	4E-69	262	140	98.57	0	0
OTU271	FJ917556	Fungi	Ascomycota	Dothideomycetes	Pleosporales	Leptosphaeriaceae	*Leptosphaeria*	4E-85	315	194	96.91	3	4
OTU414	AB998515	Fungi	Ascomycota	Dothideomycetes	Pleosporales	Leptosphaeriaceae	*Leptosphaeria*	1E-59	230	124	98.39	0	0
OTU461	KC857282	Fungi	Ascomycota	Dothideomycetes	Pleosporales	Leptosphaeriaceae	*Leptosphaeria*	2E-71	270	152	97.37	0	0
OTU113	FJ627262	Fungi	Ascomycota	Dothideomycetes	Pleosporales	Leptosphaeriaceae	*Leptosphaeria*	4E-79	295	205	96.1	6	6
OTU155	KJ188708	Fungi	Ascomycota	Dothideomycetes	Pleosporales	Leptosphaeriaceae	*Leptosphaeria*	9E-64	244	139	97.12	0	0
OTU217	KJ572145	Fungi	Ascomycota	Dothideomycetes	Pleosporales	Lophiostomataceae	*Lophiostoma*	9E-64	244	149	95.97	1	3
OTU387	KM051384	Fungi	Ascomycota	Dothideomycetes	Pleosporales	Lophiostomataceae	*Lophiostoma*	2E-77	289	162	98.15	1	1
OTU213	HQ631046	Fungi	Ascomycota	Dothideomycetes	Pleosporales	Massarinaceae	*Massarina*	3E-50	198	108	98.15	0	0
OTU347	KJ525663	Fungi	Ascomycota	Dothideomycetes	Pleosporales	Montagnulaceae	*Paraconiothyrium*	2E-71	270	144	99.31	1	1
OTU316	GU936114	Fungi	Ascomycota	Dothideomycetes	Pleosporales	Montagnulaceae	*Paraconiothyrium*	6E-84	311	181	98.34	3	3
OTU232	GU721442	Fungi	Ascomycota	Dothideomycetes	Pleosporales	Montagnulaceae	*Paraconiothyrium*	7E-71	268	155	96.77	0	0
OTU491	DQ914681	Fungi	Ascomycota	Dothideomycetes	Pleosporales	Montagnulaceae	*Paraconiothyrium*	2E-43	176	169	89.94	3	3
OTU339	KM051384	Fungi	Ascomycota	Dothideomycetes	Pleosporales	Phaeosphaeriaceae	*Ophiosphaerella*	3E-55	216	153	96.08	5	5
OTU112	EF504325	Fungi	Ascomycota	Dothideomycetes	Pleosporales	Phaeosphaeriaceae	*Phaeosphaeria*	9E-51	200	109	98.17	0	0
OTU274	AB255241	Fungi	Ascomycota	Dothideomycetes	Pleosporales	Phaeosphaeriaceae	*Phaeosphaeria*	2E-55	216	149	95.97	4	4
OTU225	HE584827	Fungi	Ascomycota	Dothideomycetes	Pleosporales	Phaeosphaeriaceae	*Phaeosphaeria*	3E-21	103	60	96.67	0	0
OTU329	EU035415	Fungi	Ascomycota	Dothideomycetes	Pleosporales	Phaeosphaeriaceae	*Phaeosphaeria*	3E-58	226	194	91.24	4	8
OTU405	JX967403	Fungi	Ascomycota	Dothideomycetes	Pleosporales	Phaeosphaeriaceae	*Phaeosphaeriopsis*	1E-38	161	157	89.81	4	8
OTU134	JX535092	Fungi	Ascomycota	Dothideomycetes	Pleosporales	Phaeosphaeriaceae	*Phaeosphaeriopsis*	2E-21	103	72	94.44	1	1
OTU445	GQ511957	Fungi	Ascomycota	Dothideomycetes	Pleosporales	Phaeosphaeriaceae	*Phaeosphaeriopsis*	5E-44	178	166	90.96	4	4
OTU353	DQ530216	Fungi	Ascomycota	Dothideomycetes	Pleosporales	Phaeosphaeriaceae	*Sclerostagonospora*	2E-24	113	145	89.66	7	7
OTU764	AB255262	Fungi	Ascomycota	Dothideomycetes	Pleosporales	Phaeosphaeriaceae	*Stagonospora*	7E-74	278	152	98.68	1	1

OTU716	KC978007	Fungi	Ascomycota	Dothideomycetes	Pleosporales	Pleosporaceae	*Alternaria*	3E-74	280	149	99.33	1	1
OTU780	FJ781312	Fungi	Ascomycota	Dothideomycetes	Pleosporales	Pleosporaceae	*Alternaria*	1E-66	254	163	96.32	3	4
OTU715	KC753419	Fungi	Ascomycota	Dothideomycetes	Pleosporales	Pleosporaceae	*Alternaria*	1E-38	161	147	91.16	4	6
OTU386	KJ780809	Fungi	Ascomycota	Dothideomycetes	Pleosporales	Pleosporaceae	*Bipolaris*	1E-72	274	154	98.05	1	1
OTU722	KM215637	Fungi	Ascomycota	Dothideomycetes	Pleosporales	Pleosporaceae	*Epicoccum*	1E-53	210	161	95.03	6	7
OTU307	JF945390	Fungi	Ascomycota	Dothideomycetes	Pleosporales	Pleosporaceae	*Epicoccum*	2E-55	216	133	97.74	3	3
OTU283	FJ627262	Fungi	Ascomycota	Dothideomycetes	Pleosporales	Shiraiaceae	*Shiraia*	4E-88	325	200	95.5	0	0
OTU157	AF314984	Fungi	Ascomycota	Dothideomycetes	Pleosporales	Shiraiaceae	*Shiraia*	1E-85	317	187	96.79	1	2
OTU1	AB255241	Fungi	Ascomycota	Dothideomycetes	Pleosporales	Shiraiaceae	*Shiraia*	2E-61	236	147	96.6	2	2
OTU74	KM246756	Fungi	Ascomycota	Dothideomycetes	Pleosporales	Shiraiaceae	*Shiraia*	2E-77	289	174	97.13	2	2
OTU199	KJ677255	Fungi	Ascomycota	Dothideomycetes	Pleosporales	Shiraiaceae	*Shiraia*	2E-77	289	154	98.7	0	0
OTU40	KM023784	Fungi	Ascomycota	Dothideomycetes	Pleosporales	Shiraiaceae	*Shiraia*	6E-71	268	147	97.96	0	0
OTU20	KM246232	Fungi	Ascomycota	Dothideomycetes	Pleosporales	Shiraiaceae	*Shiraia*	1E-44	180	123	95.12	2	2
OTU266	KM923758	Fungi	Ascomycota	Dothideomycetes	Pleosporales	Shiraiaceae	*Shiraia*	4E-66	252	147	97.96	2	2
OTU313	AB998271	Fungi	Ascomycota	Dothideomycetes	Pleosporales	Shiraiaceae	*Shiraia*	4E-88	325	180	98.33	1	1
OTU75	AJ875349	Fungi	Ascomycota	Dothideomycetes	Pleosporales	Shiraiaceae	*Shiraia*	2E-68	260	147	97.96	1	1
OTU181	KF901501	Fungi	Ascomycota	Dothideomycetes	Pleosporales	Shiraiaceae	*Shiraia*	2E-65	250	150	97.33	2	2
OTU451	KM232438	Fungi	Ascomycota	Dothideomycetes	Pleosporales	Shiraiaceae	*Shiraia*	2E-74	280	157	98.73	2	2
OTU24	GQ999538	Fungi	Ascomycota	Dothideomycetes	Pleosporales	Shiraiaceae	*Shiraia*	5E-51	202	106	99.06	0	0
OTU403	HM992800	Fungi	Ascomycota	Dothideomycetes	Pleosporales	Shiraiaceae	*Shiraia*	4E-107	389	227	96.92	1	2
OTU193	JX524287	Fungi	Ascomycota	Dothideomycetes	Pleosporales	Shiraiaceae	*Shiraia*	4E-63	242	158	95.57	2	2
OTU170	EU035415	Fungi	Ascomycota	Dothideomycetes	Pleosporales	Shiraiaceae	*Shiraia*	2E-53	210	201	90.05	5	10
OTU58	GQ999538	Fungi	Ascomycota	Dothideomycetes	Pleosporales	Shiraiaceae	*Shiraia*	4E-39	163	105	96.19	2	3
OTU148	KM246756	Fungi	Ascomycota	Dothideomycetes	Pleosporales	Shiraiaceae	*Shiraia*	2E-80	299	179	97.21	2	2
OTU408	KM232438	Fungi	Ascomycota	Dothideomycetes	Pleosporales	Shiraiaceae	*Shiraia*	3E-70	266	161	97.52	3	4
OTU55	JQ009310	Fungi	Ascomycota	Dothideomycetes	Pleosporales	Shiraiaceae	*Shiraia*	3E-33	143	88	95.45	0	0

OTU88	KM265911	Fungi	Ascomycota	Dothideomycetes	Pleosporales	Shiraiaceae	*Shiraia*	4E-94	345	182	99.45	1	1
OTU479	KM052900	Fungi	Ascomycota	Dothideomycetes	Pleosporales	Shiraiaceae	*Shiraia*	2E-64	246	148	96.62	1	1
OTU140	DQ914681	Fungi	Ascomycota	Dothideomycetes	Pleosporales	Shiraiaceae	*Shiraia*	3E-45	182	170	90.59	4	6
OTU528	KC491368	Fungi	Ascomycota	Dothideomycetes	Pleosporales	Shiraiaceae	*Shiraia*	1E-33	145	176	86.93	3	4
OTU548	KC857282	Fungi	Ascomycota	Dothideomycetes	Pleosporales	Shiraiaceae	*Shiraia*	9E-61	234	149	95.3	1	2
OTU526	JX556228	Fungi	Ascomycota	Dothideomycetes	Pleosporales	Sporormiaceae	*Preussia*	2E-77	289	162	98.77	2	2
OTU454	NR_073231	Fungi	Ascomycota	Dothideomycetes	Pleosporales	Sporormiaceae	*Preussia*	1E-56	220	111	100	0	0
OTU288	JX998699	Fungi	Ascomycota	Dothideomycetes	Pleosporales	unidentified	unidentified	8E-58	224	145	95.17	1	1
OTU246	FJ627262	Fungi	Ascomycota	Dothideomycetes	Pleosporales	unidentified	unidentified	3E-95	349	200	98	2	2
OTU554	KP132479	Fungi	Ascomycota	Dothideomycetes	Pleosporales	unidentified	unidentified	5E-75	281	166	96.39	0	0
OTU262	KM051384	Fungi	Ascomycota	Dothideomycetes	Pleosporales	unidentified	unidentified	4E-66	252	167	96.41	4	4
OTU345	JX998717	Fungi	Ascomycota	Dothideomycetes	Pleosporales	unidentified	unidentified	6E-68	258	146	97.95	1	1
OTU412	AB036065	Fungi	Ascomycota	Dothideomycetes	Pleosporales	unidentified	unidentified	6E-71	268	143	98.6	0	0
OTU146	JX268524	Fungi	Ascomycota	Dothideomycetes	Pleosporales	unidentified	unidentified	7E-68	258	162	96.3	2	2
OTU292	GU981606	Fungi	Ascomycota	Dothideomycetes	Pleosporales	unidentified	unidentified	6E-90	331	175	99.43	1	1
OTU13	FJ770078	Fungi	Ascomycota	Dothideomycetes	Pleosporales	unidentified	unidentified	6E-28	125	139	88.49	3	3
OTU97	JF694932	Fungi	Ascomycota	Dothideomycetes	Pleosporales	unidentified	unidentified	1E-59	230	154	95.45	3	5
OTU333	JX984735	Fungi	Ascomycota	Dothideomycetes	Pleosporales	unidentified	unidentified	6E-62	238	148	96.62	2	2
OTU10	FJ820729	Fungi	Ascomycota	Dothideomycetes	Pleosporales	unidentified	unidentified	7E-65	248	165	95.76	3	3
OTU237	GQ999534	Fungi	Ascomycota	Dothideomycetes	Pleosporales	unidentified	unidentified	6E-65	248	152	96.71	2	3
OTU365	JN904871	Fungi	Ascomycota	Dothideomycetes	Pleosporales	unidentified	unidentified	6E-21	101	71	94.37	1	1
OTU106	HQ696058	Fungi	Ascomycota	Dothideomycetes	Pleosporales	unidentified	unidentified	1E-59	230	148	97.3	4	4
OTU362	FJ025287	Fungi	Ascomycota	Dothideomycetes	Pleosporales	unidentified	unidentified	1E-78	293	152	99.34	0	0
OTU123	GQ999538	Fungi	Ascomycota	Dothideomycetes	Pleosporales	unidentified	unidentified	1E-48	194	106	99.06	1	1
OTU439	KM272006	Fungi	Ascomycota	Dothideomycetes	unidentified	unidentified	unidentified	9E-130	464	242	99.17	0	0
OTU740	GU214635	Fungi	Ascomycota	Dothideomycetes	Venturiales	Venturiaceae	unidentified	1E-29	131	137	89.78	4	5

OTU290	AY737765	Fungi	Ascomycota	Dothideomycetes	Venturiales	Venturiaceae	*Xenomeris*	1E-100	367	201	98.51	1	1
OTU573	JX436317	Fungi	Ascomycota	Eurotiomycetes	Chaetothyriales	Chaetothyriaceae	*Camptophora*	2E-55	216	169	94.08	5	5
OTU122	KM052887	Fungi	Ascomycota	Eurotiomycetes	Chaetothyriales	Cyphellophoraceae	*Cyphellophora*	2E-64	246	151	96.69	2	3
OTU291	GQ999538	Fungi	Ascomycota	Eurotiomycetes	Chaetothyriales	Cyphellophoraceae	*Cyphellophora*	9E-53	208	105	100	0	0
OTU385	KF156293	Fungi	Ascomycota	Eurotiomycetes	Chaetothyriales	Cyphellophoraceae	*Cyphellophora*	1E-50	200	148	95.27	5	6
OTU236	EU035415	Fungi	Ascomycota	Eurotiomycetes	Chaetothyriales	Cyphellophoraceae	*Cyphellophora*	6E-44	178	202	89.6	8	12
OTU424	KC965494	Fungi	Ascomycota	Eurotiomycetes	Chaetothyriales	Cyphellophoraceae	*Cyphellophora*	3E-20	99.6	50	100	0	0
OTU120	FJ798594	Fungi	Ascomycota	Eurotiomycetes	Chaetothyriales	Cyphellophoraceae	*Cyphellophora*	7E-74	278	144	99.31	0	0
OTU175	DQ914681	Fungi	Ascomycota	Eurotiomycetes	Chaetothyriales	Cyphellophoraceae	*Cyphellophora*	2E-62	240	172	93.6	2	3
OTU392	HE584827	Fungi	Ascomycota	Eurotiomycetes	Chaetothyriales	Cyphellophoraceae	*Cyphellophora*	8E-25	115	176	86.36	6	8
OTU406	KF435574	Fungi	Ascomycota	Eurotiomycetes	Chaetothyriales	Cyphellophoraceae	*Cyphellophora*	4E-66	252	155	97.42	3	3
OTU511	AB980801	Fungi	Ascomycota	Eurotiomycetes	Chaetothyriales	Cyphellophoraceae	unidentified	8E-74	278	176	96.59	3	3
OTU473	HM992800	Fungi	Ascomycota	Eurotiomycetes	Chaetothyriales	Cyphellophoraceae	unidentified	3E-83	309	233	93.56	5	8
OTU185	JX014309	Fungi	Ascomycota	Eurotiomycetes	Chaetothyriales	Cyphellophoraceae	unidentified	1E-101	371	227	96.48	2	2
OTU309	JX998699	Fungi	Ascomycota	Eurotiomycetes	Chaetothyriales	Cyphellophoraceae	unidentified	1E-53	210	142	95.07	2	2
OTU376	Z81427	Fungi	Ascomycota	Eurotiomycetes	Chaetothyriales	Cyphellophoraceae	unidentified	4E-26	119	96	91.67	1	1
OTU391	AY598859	Fungi	Ascomycota	Eurotiomycetes	Chaetothyriales	Cyphellophoraceae	unidentified	4E-44	178	122	93.44	0	0
OTU171	FJ820729	Fungi	Ascomycota	Eurotiomycetes	Chaetothyriales	Cyphellophoraceae	unidentified	2E-46	186	106	98.11	1	1
OTU512	DQ530216	Fungi	Ascomycota	Eurotiomycetes	Chaetothyriales	Cyphellophoraceae	unidentified	6E-59	228	135	97.04	1	1
OTU310	KF222008	Fungi	Ascomycota	Eurotiomycetes	Chaetothyriales	Herpotrichiellaceae	*Phialophora*	2E-24	113	93	92.47	2	2
OTU173	FJ438386	Fungi	Ascomycota	Eurotiomycetes	Chaetothyriales	Herpotrichiellaceae	*Phialophora*	3E-98	359	217	97.7	4	4
OTU487	AB520352	Fungi	Ascomycota	Eurotiomycetes	Chaetothyriales	Herpotrichiellaceae	*Phialophora*	2E-58	226	118	99.15	0	0
OTU520	FJ820729	Fungi	Ascomycota	Eurotiomycetes	Chaetothyriales	Herpotrichiellaceae	*Phialophora*	4E-60	232	153	96.08	3	3
OTU64	DQ682585	Fungi	Ascomycota	Eurotiomycetes	Chaetothyriales	Herpotrichiellaceae	*Phialophora*	1E-56	220	130	97.69	2	3
OTU109	HQ631017	Fungi	Ascomycota	Eurotiomycetes	Chaetothyriales	Herpotrichiellaceae	*Phialophora*	2E-49	196	139	94.96	3	3
OTU380	EU686935	Fungi	Ascomycota	Eurotiomycetes	Chaetothyriales	Herpotrichiellaceae	*Phialophora*	3E-20	99.6	94	89.36	1	1

OTU125	DQ680687	Fungi	Ascomycota	Eurotiomycetes	Chaetothyriales	Herpotrichiellaceae	*Phialophora*	1E-94	347	175	100	0	0
OTU92	FJ757040	Fungi	Ascomycota	Eurotiomycetes	Chaetothyriales	Herpotrichiellaceae	*Phialophora*	8E-64	244	135	97.78	0	0
OTU39	JX014309	Fungi	Ascomycota	Eurotiomycetes	Chaetothyriales	Herpotrichiellaceae	*Phialophora*	7E-112	404	220	99.09	2	2
OTU102	KM246186	Fungi	Ascomycota	Eurotiomycetes	Chaetothyriales	Herpotrichiellaceae	*Phialophora*	5E-62	238	120	100	0	0
OTU197	JQ009310	Fungi	Ascomycota	Eurotiomycetes	Chaetothyriales	Incertae	*Strelitziana*	1E-35	151	88	96.59	0	0
OTU44	JX624261	Fungi	Ascomycota	Eurotiomycetes	Chaetothyriales	Incertae	*Strelitziana*	2E-71	270	148	98.65	1	1
OTU100	DQ914681	Fungi	Ascomycota	Eurotiomycetes	Chaetothyriales	Incertae	*Strelitziana*	6E-47	188	171	91.23	4	4
OTU229	KM527126	Fungi	Ascomycota	Eurotiomycetes	Chaetothyriales	Incertae	*Strelitziana*	5E-59	228	143	95.8	1	1
OTU209	FJ798594	Fungi	Ascomycota	Eurotiomycetes	Chaetothyriales	unidentified	unidentified	6E-65	248	149	97.32	2	2
OTU384	KF143800	Fungi	Ascomycota	Eurotiomycetes	Chaetothyriales	unidentified	unidentified	5E-59	228	139	97.12	2	2
OTU433	JN904871	Fungi	Ascomycota	Eurotiomycetes	Chaetothyriales	unidentified	unidentified	2E-20	99.6	74	93.24	1	1
OTU67	EU686935	Fungi	Ascomycota	Eurotiomycetes	Chaetothyriales	unidentified	unidentified	3E-20	99.6	94	89.36	1	1
OTU558	AF444483	Fungi	Ascomycota	Eurotiomycetes	Chaetothyriales	unidentified	unidentified	4E-25	115	74	95.95	1	1
OTU86	KF435574	Fungi	Ascomycota	Eurotiomycetes	Chaetothyriales	unidentified	unidentified	7E-74	278	152	98.03	0	0
OTU187	KM063247	Fungi	Ascomycota	Eurotiomycetes	Chaetothyriales	unidentified	unidentified	2E-71	270	144	99.31	1	1
OTU48	GU981734	Fungi	Ascomycota	Eurotiomycetes	Chaetothyriales	unidentified	unidentified	2E-102	373	216	98.15	3	3
OTU396	KF435574	Fungi	Ascomycota	Eurotiomycetes	Chaetothyriales	unidentified	unidentified	7E-74	278	152	98.03	0	0
OTU432	HE584882	Fungi	Ascomycota	Eurotiomycetes	Chaetothyriales	unidentified	unidentified	2E-46	186	132	93.94	2	4
OTU17	EU686935	Fungi	Ascomycota	Eurotiomycetes	Chaetothyriales	unidentified	unidentified	1E-22	107	94	90.43	1	1
OTU49	HQ696058	Fungi	Ascomycota	Eurotiomycetes	Chaetothyriales	unidentified	unidentified	3E-60	232	145	97.24	3	3
OTU41	GU461502	Fungi	Ascomycota	Eurotiomycetes	Chaetothyriales	unidentified	unidentified	2E-33	143	124	89.52	1	5
OTU163	EF694652	Fungi	Ascomycota	Eurotiomycetes	Chaetothyriales	unidentified	unidentified	1E-75	283	155	98.71	1	1
OTU305	EF504325	Fungi	Ascomycota	Eurotiomycetes	Chaetothyriales	unidentified	unidentified	9E-54	210	106	100	0	0
OTU200	AF444483	Fungi	Ascomycota	Eurotiomycetes	Chaetothyriales	unidentified	unidentified	2E-20	99.6	74	93.24	1	1
OTU359	AB255305	Fungi	Ascomycota	Eurotiomycetes	Chaetothyriales	unidentified	unidentified	8E-52	204	130	96.15	2	3
OTU527	JX998714	Fungi	Ascomycota	Eurotiomycetes	Chaetothyriales	unidentified	unidentified	4E-29	129	81	95.06	0	0

OTU567	HE584882	Fungi	Ascomycota	Eurotiomycetes	Chaetothyriales	unidentified	unidentified	7E-43	174	141	92.2	3	6
OTU169	HQ731642	Fungi	Ascomycota	Eurotiomycetes	Chaetothyriales	unidentified	unidentified	4E-69	262	148	97.97	1	1
OTU346	KF296406	Fungi	Ascomycota	Eurotiomycetes	Chaetothyriales	unidentified	unidentified	6E-90	331	175	99.43	1	1
OTU69	KF735008	Fungi	Ascomycota	Eurotiomycetes	Chaetothyriales	unidentified	unidentified	2E-64	246	144	97.22	1	1
OTU145	HF558655	Fungi	Ascomycota	Eurotiomycetes	Chaetothyriales	unidentified	unidentified	4E-88	325	164	100	0	0
OTU477	KF313112	Fungi	Ascomycota	Eurotiomycetes	Chaetothyriales	unidentified	unidentified	4E-85	315	206	95.63	3	4
OTU486	U65613	Fungi	Ascomycota	Eurotiomycetes	Chaetothyriales	unidentified	unidentified	3E-54	212	145	95.17	3	5
OTU223	KJ608106	Fungi	Ascomycota	Eurotiomycetes	Chaetothyriales	unidentified	unidentified	3E-60	232	121	99.17	0	0
OTU221	GQ518230	Fungi	Ascomycota	Eurotiomycetes	Chaetothyriales	unidentified	unidentified	3E-32	139	122	90.98	2	2
OTU6	GQ999538	Fungi	Ascomycota	Eurotiomycetes	Chaetothyriales	unidentified	unidentified	5E-42	172	99	97.98	1	1
OTU377	AB693792	Fungi	Ascomycota	Eurotiomycetes	Chaetothyriales	unidentified	unidentified	1E-62	240	137	97.81	1	1
OTU409	NR_073337	Fungi	Ascomycota	Eurotiomycetes	Chaetothyriales	unidentified	unidentified	1E-25	117	101	91.09	2	4
OTU273	KM205065	Fungi	Ascomycota	Eurotiomycetes	Chaetothyriales	unidentified	unidentified	2E-68	260	163	96.32	2	2
OTU514	DQ682585	Fungi	Ascomycota	Eurotiomycetes	Chaetothyriales	unidentified	unidentified	1E-56	220	130	97.69	2	3
OTU68	KM051384	Fungi	Ascomycota	Eurotiomycetes	Chaetothyriales	unidentified	unidentified	2E-74	280	165	96.97	1	1
OTU265	KM246756	Fungi	Ascomycota	Eurotiomycetes	Chaetothyriales	unidentified	unidentified	9E-83	307	179	97.77	2	2
OTU285	HM992800	Fungi	Ascomycota	Eurotiomycetes	Chaetothyriales	unidentified	unidentified	3E-83	309	230	93.91	5	7
OTU94	KF146177	Fungi	Ascomycota	Eurotiomycetes	Chaetothyriales	unidentified	unidentified	8E-102	371	199	98.49	0	0
OTU206	GQ999538	Fungi	Ascomycota	Eurotiomycetes	Chaetothyriales	unidentified	unidentified	2E-44	180	107	98.13	2	2
OTU147	KM246756	Fungi	Ascomycota	Eurotiomycetes	Chaetothyriales	unidentified	unidentified	2E-77	289	174	97.13	2	2
OTU340	KM051384	Fungi	Ascomycota	Eurotiomycetes	Chaetothyriales	unidentified	unidentified	1E-72	274	154	98.05	1	1
OTU116	AB998432	Fungi	Ascomycota	Eurotiomycetes	Chaetothyriales	unidentified	unidentified	3E-47	188	111	98.2	2	2
OTU453	NR_073231	Fungi	Ascomycota	Eurotiomycetes	Chaetothyriales	unidentified	unidentified	2E-51	202	114	98.25	1	1
OTU108	AB036065	Fungi	Ascomycota	Eurotiomycetes	Chaetothyriales	unidentified	unidentified	1E-69	264	145	97.93	0	0
OTU426	KJ652493	Fungi	Ascomycota	Eurotiomycetes	Chaetothyriales	unidentified	unidentified	2E-99	363	183	100	0	0
OTU177	EF504325	Fungi	Ascomycota	Eurotiomycetes	Chaetothyriales	unidentified	unidentified	9E-51	200	109	98.17	0	0

OTU397	KJ935008	Fungi	Ascomycota	Eurotiomycetes	Chaetothyriales	unidentified	unidentified	3E-89	329	190	97.89	2	2
OTU43	KM215637	Fungi	Ascomycota	Eurotiomycetes	Chaetothyriales	unidentified	unidentified	6E-65	248	157	96.82	3	3
OTU2	GQ999538	Fungi	Ascomycota	Eurotiomycetes	Chaetothyriales	unidentified	unidentified	2E-50	200	105	99.05	0	0
OTU8	GQ999538	Fungi	Ascomycota	Eurotiomycetes	Chaetothyriales	unidentified	unidentified	2E-50	200	105	99.05	0	0
OTU22	KJ572145	Fungi	Ascomycota	Eurotiomycetes	Chaetothyriales	unidentified	unidentified	6E-68	258	146	97.26	0	0
OTU87	KF146177	Fungi	Ascomycota	Eurotiomycetes	Chaetothyriales	unidentified	unidentified	8E-102	371	199	98.49	0	0
OTU497	GQ999538	Fungi	Ascomycota	Eurotiomycetes	Chaetothyriales	unidentified	unidentified	1E-42	174	104	97.12	1	1
OTU485	KP099830	Fungi	Ascomycota	Eurotiomycetes	Chaetothyriales	unidentified	unidentified	1E-106	387	211	98.58	1	1
OTU154	KF496199	Fungi	Ascomycota	Eurotiomycetes	Chaetothyriales	unidentified	unidentified	4E-66	252	154	96.75	2	3
OTU156	AB693792	Fungi	Ascomycota	Eurotiomycetes	Chaetothyriales	unidentified	unidentified	1E-65	250	138	98.55	1	1
OTU178	HF680223	Fungi	Ascomycota	Eurotiomycetes	Chaetothyriales	unidentified	unidentified	2E-61	236	119	100	0	0
OTU212	HE584827	Fungi	Ascomycota	Eurotiomycetes	Chaetothyriales	unidentified	unidentified	5E-32	139	176	87.5	5	7
OTU228	JQ599382	Fungi	Ascomycota	Eurotiomycetes	Chaetothyriales	unidentified	unidentified	9E-89	327	165	100	0	0
OTU379	AF444483	Fungi	Ascomycota	Eurotiomycetes	Chaetothyriales	unidentified	unidentified	9E-23	107	74	94.59	1	1
OTU399	AB255241	Fungi	Ascomycota	Eurotiomycetes	Chaetothyriales	unidentified	unidentified	6E-59	228	150	96.67	4	5
OTU427	KM036093	Fungi	Ascomycota	Eurotiomycetes	Chaetothyriales	unidentified	unidentified	1E-62	240	145	97.93	3	3
OTU459	JX014309	Fungi	Ascomycota	Eurotiomycetes	Chaetothyriales	unidentified	unidentified	9E-99	361	225	95.56	1	2
OTU513	DQ530216	Fungi	Ascomycota	Eurotiomycetes	Chaetothyriales	unidentified	unidentified	1E-35	151	141	91.49	5	8
OTU516	FJ372393	Fungi	Ascomycota	Eurotiomycetes	Chaetothyriales	unidentified	unidentified	8E-99	361	190	99.47	1	1
OTU448	KF143783	Fungi	Ascomycota	Eurotiomycetes	Eurotiales	Trichocomaceae	*Aspergillus*	4E-66	252	159	97.48	4	4
OTU233	HM992800	Fungi	Ascomycota	Eurotiomycetes	Eurotiales	Trichocomaceae	*Aspergillus*	2E-38	161	101	97.03	2	2
OTU253	AY254153	Fungi	Ascomycota	Eurotiomycetes	Eurotiales	Trichocomaceae	*Aspergillus*	2E-74	280	161	98.14	2	2
OTU216	KJ531966	Fungi	Ascomycota	Eurotiomycetes	Eurotiales	Trichocomaceae	*Aspergillus*	1E-62	240	129	99.22	1	1
OTU442	AB255305	Fungi	Ascomycota	Eurotiomycetes	Eurotiales	Trichocomaceae	*Aspergillus*	2E-67	256	141	98.58	1	1
OTU402	GQ999538	Fungi	Ascomycota	Eurotiomycetes	Eurotiales	Trichocomaceae	*Aspergillus*	5E-48	192	105	98.1	0	0
OTU336	HQ010680	Fungi	Ascomycota	Eurotiomycetes	Eurotiales	Trichocomaceae	*Aspergillus*	5E-31	135	127	92.91	6	7

OTU348	KM051384	Fungi	Ascomycota	Eurotiomycetes	Eurotiales	Trichocomaceae	*Aspergillus*	1E-81	303	161	98.76	0	0
OTU234	JX875930	Fungi	Ascomycota	Eurotiomycetes	Eurotiales	Trichocomaceae	*Aspergillus*	2E-71	270	148	98.65	1	1
OTU547	JX624261	Fungi	Ascomycota	Eurotiomycetes	Eurotiales	Trichocomaceae	*Aspergillus*	3E-57	222	151	96.03	4	5
OTU135	HM992800	Fungi	Ascomycota	Eurotiomycetes	Eurotiales	Trichocomaceae	*Eurotium*	8E-90	331	227	95.59	5	5
OTU519	FJ820729	Fungi	Ascomycota	Eurotiomycetes	Eurotiales	Trichocomaceae	*Neosartorya*	3E-67	256	157	96.82	2	2
OTU357	KF675509	Fungi	Ascomycota	Eurotiomycetes	Eurotiales	Trichocomaceae	*Neosartorya*	9E-58	224	149	94.63	1	1
OTU521	GU461502	Fungi	Ascomycota	Eurotiomycetes	Eurotiales	Trichocomaceae	*Penicillium*	8E-30	131	126	88.89	2	6
OTU480	KM056275	Fungi	Ascomycota	Eurotiomycetes	Eurotiales	Trichocomaceae	*Penicillium*	2E-74	280	157	98.73	2	2
OTU21	KM246756	Fungi	Ascomycota	Eurotiomycetes	Eurotiales	Trichocomaceae	*Penicillium*	6E-84	311	177	97.74	1	1
OTU62	KM246756	Fungi	Ascomycota	Eurotiomycetes	Eurotiales	Trichocomaceae	*Penicillium*	3E-76	285	179	96.65	3	4
OTU358	KM246204	Fungi	Ascomycota	Eurotiomycetes	Eurotiales	Trichocomaceae	*Penicillium*	5E-56	218	150	96	4	4
OTU561	AY853194	Fungi	Ascomycota	Eurotiomycetes	Eurotiales	Trichocomaceae	*Penicillium*	4E-66	252	139	98.56	1	1
OTU51	KM246232	Fungi	Ascomycota	Eurotiomycetes	Eurotiales	Trichocomaceae	*Penicillium*	4E-44	178	122	95.08	2	2
OTU168	EU035415	Fungi	Ascomycota	Eurotiomycetes	Eurotiales	Trichocomaceae	*Penicillium*	2E-56	220	199	91.46	6	10
OTU60	JX624261	Fungi	Ascomycota	Eurotiomycetes	Eurotiales	Trichocomaceae	*Penicillium*	3E-73	276	143	99.3	0	0
OTU104	EU686935	Fungi	Ascomycota	Eurotiomycetes	Eurotiales	Trichocomaceae	*Penicillium*	6E-22	105	93	90.32	1	1
OTU32	KM051384	Fungi	Ascomycota	Eurotiomycetes	Eurotiales	Trichocomaceae	*Penicillium*	1E-78	293	164	97.56	0	0
OTU517	FJ770078	Fungi	Ascomycota	Eurotiomycetes	Eurotiales	Trichocomaceae	*Penicillium*	8E-52	204	139	94.96	2	2
OTU52	GQ999538	Fungi	Ascomycota	Eurotiomycetes	Eurotiales	Trichocomaceae	*Penicillium*	1E-48	194	102	99.02	0	0
OTU105	AB255241	Fungi	Ascomycota	Eurotiomycetes	Eurotiales	Trichocomaceae	*Penicillium*	4E-63	242	146	97.26	2	2
OTU404	HQ631052	Fungi	Ascomycota	Eurotiomycetes	Eurotiales	Trichocomaceae	*Penicillium*	1E-29	131	165	86.67	3	4
OTU335	DQ682585	Fungi	Ascomycota	Eurotiomycetes	Eurotiales	Trichocomaceae	*Penicillium*	1E-41	170	141	92.91	4	5
OTU560	AJ582964	Fungi	Ascomycota	Eurotiomycetes	Eurotiales	Trichocomaceae	*Penicillium*	9E-99	361	228	96.05	3	5
OTU12	KM246756	Fungi	Ascomycota	Eurotiomycetes	Eurotiales	Trichocomaceae	*Penicillium*	9E-86	317	176	97.73	0	0
OTU19	GQ999538	Fungi	Ascomycota	Eurotiomycetes	Eurotiales	Trichocomaceae	*Penicillium*	3E-43	176	105	96.19	0	0
OTU194	KC753419	Fungi	Ascomycota	Eurotiomycetes	Eurotiales	Trichocomaceae	*Penicillium*	1E-38	161	144	90.97	3	4

OTU286	KC491368	Fungi	Ascomycota	Eurotiomycetes	Eurotiales	Trichocomaceae	*Penicillium*	4E-92	339	242	93.39	2	3
OTU411	AB036065	Fungi	Ascomycota	Eurotiomycetes	Eurotiales	Trichocomaceae	*Penicillium*	6E-71	268	139	99.28	0	0
OTU230	JX014309	Fungi	Ascomycota	Eurotiomycetes	Eurotiales	Trichocomaceae	*Penicillium*	2E-93	343	224	95.98	4	5
OTU532	AB540574	Fungi	Ascomycota	Eurotiomycetes	Eurotiales	Trichocomaceae	*Penicillium*	5E-75	281	166	96.99	1	1
OTU449	KJ572145	Fungi	Ascomycota	Eurotiomycetes	Eurotiales	Trichocomaceae	*Penicillium*	9E-64	244	147	97.28	2	2
OTU564	EU035415	Fungi	Ascomycota	Eurotiomycetes	Eurotiales	Trichocomaceae	*Penicillium*	2E-65	250	194	92.78	4	8
OTU214	KJ619992	Fungi	Ascomycota	Eurotiomycetes	Eurotiales	Trichocomaceae	*Penicillium*	4E-66	252	150	97.33	2	3
OTU250	KJ572145	Fungi	Ascomycota	Eurotiomycetes	Eurotiales	Trichocomaceae	*Penicillium*	2E-64	246	148	97.3	2	2
OTU354	HE584827	Fungi	Ascomycota	Eurotiomycetes	Eurotiales	Trichocomaceae	*Penicillium*	6E-35	149	169	87.57	3	5
OTU370	GQ999538	Fungi	Ascomycota	Eurotiomycetes	Eurotiales	Trichocomaceae	*Penicillium*	5E-48	192	105	98.1	0	0
OTU418	FJ820729	Fungi	Ascomycota	Eurotiomycetes	Eurotiales	Trichocomaceae	*Penicillium*	3E-61	236	166	94.58	3	4
OTU207	JF945390	Fungi	Ascomycota	Eurotiomycetes	Eurotiales	Trichocomaceae	*Talaromyces*	3E-48	192	133	96.24	4	4
OTU563	EU035415	Fungi	Ascomycota	Eurotiomycetes	Eurotiales	Trichocomaceae	*Talaromyces*	1E-42	174	205	88.78	8	15
OTU415	EF114689	Fungi	Ascomycota	Eurotiomycetes	Eurotiales	Trichocomaceae	*Talaromyces*	7E-43	174	147	90.48	1	2
OTU278	JX624261	Fungi	Ascomycota	Eurotiomycetes	Eurotiales	Trichocomaceae	*Talaromyces*	5E-59	228	147	96.6	3	3
OTU582	NR_073279	Fungi	Ascomycota	Eurotiomycetes	Eurotiales	Trichocomaceae	*Talaromyces*	5E-78	291	159	98.11	0	0
OTU401	AB998368	Fungi	Ascomycota	Eurotiomycetes	Eurotiales	Trichocomaceae	*Talaromyces*	2E-93	343	181	99.45	1	1
OTU531	KP131815	Fungi	Ascomycota	Eurotiomycetes	Eurotiales	Trichocomaceae	*Talaromyces*	1E-68	260	139	99.28	1	1
OTU546	HQ649891	Fungi	Ascomycota	Eurotiomycetes	Onygenales	Onygenaceae	*Chrysosporium*	7E-59	228	168	92.86	2	5
OTU551	KJ909773	Fungi	Ascomycota	Eurotiomycetes	Onygenales	Onygenaceae	*Chrysosporium*	1E-88	327	173	99.42	1	1
OTU509	NR_073337	Fungi	Ascomycota	Eurotiomycetes	Onygenales	Onygenaceae	*Chrysosporium*	2E-21	103	98	89.8	2	4
OTU468	EU041803	Fungi	Ascomycota	Eurotiomycetes	Onygenales	Onygenaceae	*Rhinocladiella*	1E-38	161	149	93.29	7	7
OTU45	DQ530216	Fungi	Ascomycota	Eurotiomycetes	Onygenales	Onygenaceae	*Rhinocladiella*	3E-42	172	143	93.01	4	4
OTU243	EU035415	Fungi	Ascomycota	Eurotiomycetes	Onygenales	Onygenaceae	*Rhinocladiella*	3E-58	226	186	91.94	4	8
OTU38	KM066541	Fungi	Ascomycota	Eurotiomycetes	Onygenales	Onygenaceae	*Rhinocladiella*	6E-81	301	176	97.16	1	1
OTU450	KM100715	Fungi	Ascomycota	Eurotiomycetes	Onygenales	Onygenaceae	*Rhinocladiella*	3E-67	256	156	97.44	3	4

OTU482	KM246756	Fungi	Ascomycota	Eurotiomycetes	Onygenales	Onygenaceae	*Rhinocladiella*	5E-78	291	171	97.66	2	2
OTU481	KM100715	Fungi	Ascomycota	Eurotiomycetes	Onygenales	Onygenaceae	*Rhinocladiella*	4E-69	262	154	97.4	2	4
OTU77	JQ520177	Fungi	Ascomycota	Incertae	Incertae	Incertae	*Acrodontium*	1E-88	327	201	97.01	3	3
OTU557	AB615578	Fungi	Ascomycota	Incertae	Incertae	Incertae	*Acrodontium*	2E-26	119	76	94.74	0	0
OTU247	GQ999538	Fungi	Ascomycota	Incertae	Incertae	Incertae	*Acrodontium*	1E-51	204	103	100	0	0
OTU249	JN198444	Fungi	Ascomycota	Incertae	Incertae	Incertae	*Acrodontium*	5E-75	281	158	98.73	2	2
OTU294	KF646151	Fungi	Ascomycota	Incertae	Incertae	Pseudeurotiaceae	*Pseudeurotium*	3E-37	155	78	100	0	0
OTU476	KF143800	Fungi	Ascomycota	Incertae				4E-66	252	139	98.56	1	1
OTU773	DQ914681	Fungi	Ascomycota	Leotiomycetes	Helotiales	Incertae	*Cystodendron*	5E-32	139	173	87.28	5	10
OTU256	HE584882	Fungi	Ascomycota	Leotiomycetes	Helotiales	Incertae	*Hyalodendriella*	3E-39	163	140	91.43	3	5
OTU115	JX014309	Fungi	Ascomycota	Leotiomycetes	Helotiales	Sclerotiniaceae	*Ciboria*	3E-108	392	222	97.3	0	0
OTU270	DQ914681	Fungi	Ascomycota	Leotiomycetes	Helotiales	Sclerotiniaceae	*Ciboria*	9E-49	194	172	90.7	3	5
OTU407	KJ957774	Fungi	Ascomycota	Leotiomycetes	Helotiales	Sclerotiniaceae	unidentified	3E-73	276	163	98.16	3	3
OTU393	JN395828	Fungi	Ascomycota	Leotiomycetes	Helotiales	Sclerotiniaceae	unidentified	2E-44	180	228	86.4	5	12
OTU231	KC785567	Fungi	Ascomycota	Leotiomycetes	Incertae	Myxotrichaceae	*Oidiodendron*	4E-75	281	142	100	0	0
OTU308	JN904871	Fungi	Ascomycota	Saccharomycetes	Saccharomycetales	Incertae	*Candida*	1E-22	107	74	94.59	1	1
OTU369	KJ572145	Fungi	Ascomycota	Saccharomycetes	Saccharomycetales	Incertae	*Candida*	4E-66	252	147	97.28	1	1
OTU490	DQ530216	Fungi	Ascomycota	Saccharomycetes	Saccharomycetales	Incertae		1E-41	170	145	93.1	5	6
OTU533	AB693818	Fungi	Ascomycota	Saccharomycetes	Saccharomycetales	Metschnikowiaceae	*Metschnikowia*	6E-87	321	170	99.41	1	1
OTU81	AB036065	Fungi	Ascomycota	Saccharomycetes	Saccharomycetales	Metschnikowiaceae	*Metschnikowia*	4E-72	272	145	98.62	0	0
OTU279	KJ699113	Fungi	Ascomycota	Saccharomycetes	Saccharomycetales	Metschnikowiaceae	*Metschnikowia*	4E-69	262	140	99.29	1	1
OTU227	KC978007	Fungi	Ascomycota	Saccharomycetes	Saccharomycetales	Metschnikowiaceae	*Metschnikowia*	4E-98	359	216	96.3	1	2
OTU196	FJ762687	Fungi	Ascomycota	Saccharomycetes	Saccharomycetales	Metschnikowiaceae	*Metschnikowia*	4E-66	252	143	97.9	1	1
OTU190	FJ425662	Fungi	Ascomycota	Saccharomycetes	Saccharomycetales	Metschnikowiaceae	*Metschnikowia*	1E-41	170	134	93.28	3	3
OTU289	AF383955	Fungi	Ascomycota	Saccharomycetes	Saccharomycetales	Metschnikowiaceae	*Metschnikowia*	1E-50	200	155	92.9	3	5
OTU417	FJ757656	Fungi	Ascomycota	Saccharomycetes	Saccharomycetales	Metschnikowiaceae	*Metschnikowia*	2E-80	299	167	98.8	2	2

OTU151	GQ999538	Fungi	Ascomycota	Saccharomycetes	Saccharomycetales	Metschnikowiaceae	*Metschnikowia*	1E-33	145	81	98.77	1	1
OTU515	EU002878	Fungi	Ascomycota	Saccharomycetes	Saccharomycetales	Metschnikowiaceae	*Metschnikowia*	4E-66	252	147	97.28	1	1
OTU284	GQ999538	Fungi	Ascomycota	Saccharomycetes	Saccharomycetales	Metschnikowiaceae	*Metschnikowia*	1E-48	194	106	98.11	0	0
OTU215	GQ999538	Fungi	Ascomycota	Saccharomycetes	Saccharomycetales	Pichiaceae	*Pichia*	8E-47	188	95	100	0	0
OTU410	UDB015281	Fungi	Ascomycota	Saccharomycetes	Saccharomycetales	Pichiaceae	*Pichia*	1E-91	337	234	94.44	3	3
OTU323	KM199317	Fungi	Ascomycota	Sordariomycetes	Diaporthales	Diaporthaceae	*Phomopsis*	1E-106	387	195	100	0	0
OTU180	EU686935	Fungi	Ascomycota	Sordariomycetes	Hypocreales	Bionectriaceae	*Bionectria*	1E-22	107	94	90.43	1	1
OTU457	DQ530216	Fungi	Ascomycota	Sordariomycetes	Hypocreales	Bionectriaceae	unidentified	5E-44	178	139	92.81	3	6
OTU471	FJ820729	Fungi	Ascomycota	Sordariomycetes	Hypocreales	Clavicipitaceae	*Metacordyceps*	1E-66	254	166	95.18	2	4
OTU300	HM992800	Fungi	Ascomycota	Sordariomycetes	Hypocreales	Clavicipitaceae	*Villosiclava*	3E-92	339	230	95.22	4	5
OTU198	KC354795	Fungi	Ascomycota	Sordariomycetes	Hypocreales	Cordycipitaceae	*Beauveria*	2E-62	240	149	95.97	1	1
OTU251	NR_073337	Fungi	Ascomycota	Sordariomycetes	Hypocreales	Cordycipitaceae	*Beauveria*	1E-25	117	105	90.48	2	4
OTU160	JX014309	Fungi	Ascomycota	Sordariomycetes	Hypocreales	Cordycipitaceae	*Cordyceps*	1E-107	391	229	98.25	4	4
OTU242	KM051384	Fungi	Ascomycota	Sordariomycetes	Hypocreales	Hypocreaceae	*Gliocladium*	2E-74	280	165	96.97	1	1
OTU390	AB998390	Fungi	Ascomycota	Sordariomycetes	Hypocreales	Hypocreaceae	*Gliocladium*	9E-48	190	112	98.21	2	2
OTU53	FJ917556	Fungi	Ascomycota	Sordariomycetes	Hypocreales	Hypocreaceae	*Gliocladium*	2E-96	353	194	98.97	2	2
OTU352	DQ530216	Fungi	Ascomycota	Sordariomycetes	Hypocreales	Hypocreaceae	*Gliocladium*	2E-25	117	124	88.71	3	6
OTU372	JX043123	Fungi	Ascomycota	Sordariomycetes	Hypocreales	Hypocreaceae	*Gliocladium*	2E-21	103	87	90.8	1	2
OTU131	GQ999538	Fungi	Ascomycota	Sordariomycetes	Hypocreales	Hypocreaceae	*Hypocrea*	3E-46	186	106	98.11	1	1
OTU455	AM901803	Fungi	Ascomycota	Sordariomycetes	Hypocreales	Hypocreaceae	*Hypocrea*	4E-66	252	139	97.84	0	0
OTU211	AB998390	Fungi	Ascomycota	Sordariomycetes	Hypocreales	Hypocreaceae	*Hypocrea*	1E-52	206	108	99.07	0	0
OTU304	AY604831	Fungi	Ascomycota	Sordariomycetes	Hypocreales	Hypocreaceae	*Hypocrea*	8E-46	184	157	92.99	5	5
OTU420	HM992800	Fungi	Ascomycota	Sordariomycetes	Hypocreales	Hypocreaceae	*Trichoderma*	2E-93	343	227	94.71	2	4
OTU330	FJ820729	Fungi	Ascomycota	Sordariomycetes	Hypocreales	Hypocreaceae	*Trichoderma*	3E-64	246	167	95.81	4	5
OTU144	GU981736	Fungi	Ascomycota	Sordariomycetes	Hypocreales	Hypocreaceae	*Trichoderma*	9E-102	371	211	98.58	3	3
OTU416	EU035415	Fungi	Ascomycota	Sordariomycetes	Hypocreales	Hypocreaceae	*Trichoderma*	3E-49	196	187	90.37	5	9

OTU301	JX875935	Fungi	Ascomycota	Sordariomycetes	Hypocreales	Hypocreaceae	*Trichoderma*	1E-94	347	183	99.45	1	1
OTU522	HM162138	Fungi	Ascomycota	Sordariomycetes	Hypocreales	Incertae	*Acremonium*	9E-64	244	147	95.92	0	0
OTU422	JX014309	Fungi	Ascomycota	Sordariomycetes	Hypocreales	Incertae	*Acremonium*	3E-108	392	226	97.35	1	1
OTU127	FJ790250	Fungi	Ascomycota	Sordariomycetes	Hypocreales	Incertae	*Acremonium*	2E-28	127	96	94.79	3	3
OTU165	HM992800	Fungi	Ascomycota	Sordariomycetes	Hypocreales	Incertae	*Acremonium*	3E-77	289	230	94.35	8	8
OTU239	NR_077114	Fungi	Ascomycota	Sordariomycetes	Hypocreales	Incertae	*Myrothecium*	2E-67	256	133	99.25	0	0
OTU183	DQ530216	Fungi	Ascomycota	Sordariomycetes	Hypocreales	Incertae	*Myrothecium*	7E-34	145	145	92.41	7	7
OTU772	DQ530216	Fungi	Ascomycota	Sordariomycetes	Hypocreales	Incertae	*Sarocladium*	4E-23	109	138	89.86	7	8
OTU803	KF036591	Fungi	Ascomycota	Sordariomycetes	Hypocreales	Incertae	*Sarocladium*	4E-28	125	71	97.18	0	0
OTU732	EU035415	Fungi	Ascomycota	Sordariomycetes	Hypocreales	Incertae	*Sarocladium*	1E-32	141	196	87.24	8	15
OTU800	KC460868	Fungi	Ascomycota	Sordariomycetes	Hypocreales	Incertae	*Sarocladium*	7E-59	228	155	93.55	0	0
OTU752	KC978007	Fungi	Ascomycota	Sordariomycetes	Hypocreales	Incertae	*Sarocladium*	9E-99	361	218	96.79	2	2
OTU714	KC354795	Fungi	Ascomycota	Sordariomycetes	Hypocreales	Incertae	*Sarocladium*	2E-55	216	155	94.19	3	5
OTU297	KM527126	Fungi	Ascomycota	Sordariomycetes	Hypocreales	Incertae	*Sarocladium*	5E-59	228	143	95.8	1	1
OTU315	GQ999538	Fungi	Ascomycota	Sordariomycetes	Hypocreales	Incertae	*Sarocladium*	5E-48	192	97	100	0	0
OTU723	KM246756	Fungi	Ascomycota	Sordariomycetes	Hypocreales	Incertae	*Sarocladium*	2E-74	280	180	96.11	3	4
OTU731	DQ530216	Fungi	Ascomycota	Sordariomycetes	Hypocreales	Incertae	*Sarocladium*	7E-43	174	137	92.7	3	6
OTU805	KJ706360	Fungi	Ascomycota	Sordariomycetes	Hypocreales	Incertae	*Sarocladium*	6E-68	258	146	98.63	2	2
OTU95	GQ999538	Fungi	Ascomycota	Sordariomycetes	Hypocreales	Incertae		1E-45	184	105	98.1	1	1
OTU374	KF222008	Fungi	Ascomycota	Sordariomycetes	Hypocreales	Incertae		7E-46	184	132	93.18	1	2
OTU355	JN905220	Fungi	Ascomycota	Sordariomycetes	Hypocreales	Incertae		1E-28	127	120	92.5	5	5
OTU421	JQ747510	Fungi	Ascomycota	Sordariomycetes	Hypocreales	Nectriaceae	*Cylindrocarpon*	1E-91	337	182	98.9	1	1
OTU534	AB980801	Fungi	Ascomycota	Sordariomycetes	Hypocreales	Nectriaceae	*Fusarium*	6E-93	341	172	100	0	0
OTU366	JX984735	Fungi	Ascomycota	Sordariomycetes	Hypocreales	Nectriaceae	*Fusarium*	8E-52	204	147	93.88	2	2
OTU334	KC485065	Fungi	Ascomycota	Sordariomycetes	Hypocreales	Nectriaceae	*Fusarium*	5E-75	281	170	96.47	1	1
OTU85	DQ530216	Fungi	Ascomycota	Sordariomycetes	Hypocreales	Nectriaceae	*Fusarium*	2E-37	157	143	92.31	5	5

OTU158	HE584882	Fungi	Ascomycota	Sordariomycetes	Hypocreales	Nectriaceae	*Fusarium*	3E-45	182	144	92.36	2	2
OTU149	FJ820729	Fungi	Ascomycota	Sordariomycetes	Hypocreales	Nectriaceae	*Fusarium*	3E-70	266	165	96.36	2	3
OTU153	JX014309	Fungi	Ascomycota	Sordariomycetes	Hypocreales	Nectriaceae	*Fusarium*	4E-104	379	227	97.36	3	3
OTU440	KM923758	Fungi	Ascomycota	Sordariomycetes	Hypocreales	Nectriaceae	*Fusarium*	4E-72	272	149	98.66	1	1
OTU70	AB255241	Fungi	Ascomycota	Sordariomycetes	Hypocreales	Nectriaceae	*Fusarium*	2E-64	246	136	98.53	1	1
OTU34	KF269234	Fungi	Ascomycota	Sordariomycetes	Hypocreales	Nectriaceae	*Fusarium*	2E-68	260	155	98.06	3	3
OTU84	JQ936142	Fungi	Ascomycota	Sordariomycetes	Hypocreales	Nectriaceae	*Fusarium*	7E-77	287	157	98.73	1	1
OTU444	FJ791145	Fungi	Ascomycota	Sordariomycetes	Hypocreales	Nectriaceae	*Fusarium*	3E-24	113	65	96.92	0	0
OTU101	JQ905732	Fungi	Ascomycota	Sordariomycetes	Hypocreales	Nectriaceae	*Fusidium*	7E-68	258	142	97.89	0	0
OTU325	KF212203	Fungi	Ascomycota	Sordariomycetes	Hypocreales	Nectriaceae	*Fusidium*	3E-73	276	155	98.71	2	2
OTU161	Z81427	Fungi	Ascomycota	Sordariomycetes	Hypocreales	Nectriaceae	*Fusidium*	1E-41	170	130	91.54	0	0
OTU260	KC485065	Fungi	Ascomycota	Sordariomycetes	Hypocreales	Nectriaceae	*Nectria*	3E-70	266	170	95.88	2	2
OTU328	EF504325	Fungi	Ascomycota	Sordariomycetes	Hypocreales	Nectriaceae	*Nectria*	8E-45	180	111	97.3	2	2
OTU538	EU035415	Fungi	Ascomycota	Sordariomycetes	Hypocreales	Niessliaceae	*Myrmaecium*	1E-39	165	205	88.78	9	15
OTU150	GU721805	Fungi	Ascomycota	Sordariomycetes	Hypocreales	Ophiocordycipitaceae	*Purpureocillium*	3E-76	285	152	98.68	0	0
OTU302	AB733121	Fungi	Ascomycota	Sordariomycetes	Hypocreales	Stachybotriaceae	*Stachybotrys*	5E-97	355	203	98.03	2	2
OTU458	DQ914681	Fungi	Ascomycota	Sordariomycetes	Hypocreales	unidentified	unidentified	6E-50	198	168	91.67	3	3
OTU119	DQ068346	Fungi	Ascomycota	Sordariomycetes	Hypocreales	unidentified	unidentified	3E-39	163	82	100	0	0
OTU493	FJ627262	Fungi	Ascomycota	Sordariomycetes	Hypocreales	unidentified	unidentified	4E-82	305	202	96.04	4	4
OTU755	KJ911228	Fungi	Ascomycota	Sordariomycetes	Incertae	Apiosporaceae	*Arthrinium*	9E-108	391	209	99.04	1	1
OTU132	KF435380	Fungi	Ascomycota	Sordariomycetes	Incertae	Apiosporaceae	*Arthrinium*	3E-39	163	126	93.65	3	3
OTU541	FJ627262	Fungi	Ascomycota	Sordariomycetes	Incertae	Cephalothecaceae	*Phialemonium*	7E-84	311	205	96.59	5	5
OTU464	KJ608106	Fungi	Ascomycota	Sordariomycetes	Incertae	Cephalothecaceae	*Phialemonium*	2E-58	226	118	99.15	0	0
OTU475	KC485065	Fungi	Ascomycota	Sordariomycetes	Incertae	Cephalothecaceae	*Phialemonium*	2E-77	289	170	97.06	1	1
OTU222	KJ598810	Fungi	Ascomycota	Sordariomycetes	Incertae	Cephalothecaceae	*Phialemonium*	2E-93	343	181	99.45	1	1
OTU525	JX268524	Fungi	Ascomycota	Sordariomycetes	Incertae	Cephalothecaceae	*Phialemonium*	7E-59	228	174	93.1	3	4

OTU79	AF325173	Fungi	Ascomycota	Sordariomycetes	Incertae	Cephalothecaceae	*Phialemonium*	2E-42	172	111	96.4	2	2
OTU495	FJ790250	Fungi	Ascomycota	Sordariomycetes	Incertae	Cephalothecaceae	*Phialemonium*	1E-22	107	94	92.55	3	3
OTU571	JN905318	Fungi	Ascomycota	Sordariomycetes	Incertae	Cephalothecaceae	*Phialemonium*	5E-59	228	115	100	0	0
OTU14	FJ820729	Fungi	Ascomycota	Sordariomycetes	Incertae	Cephalothecaceae	*Phialemonium*	3E-67	256	164	95.12	1	2
OTU31	KC685630	Fungi	Ascomycota	Sordariomycetes	Incertae	Cephalothecaceae	*Phialemonium*	8E-58	224	129	98.45	2	2
OTU438	KF746124	Fungi	Ascomycota	Sordariomycetes	Incertae	Cephalothecaceae	*Phialemonium*	5E-97	355	187	99.47	1	1
OTU4	KC354795	Fungi	Ascomycota	Sordariomycetes	Incertae	Cephalothecaceae	*Phialemonium*	6E-65	248	153	95.42	0	0
OTU143	NR_073218	Fungi	Ascomycota	Sordariomycetes	Incertae	Cephalothecaceae	*Phialemonium*	2E-51	202	110	98.18	0	0
OTU124	KJ572145	Fungi	Ascomycota	Sordariomycetes	Incertae	Cephalothecaceae	*Phialemonium*	3E-73	276	139	100	0	0
OTU220	JX014309	Fungi	Ascomycota	Sordariomycetes	Incertae	Glomerellaceae	*Colletotrichum*	1E-101	371	203	98.52	1	1
OTU389	AB615460	Fungi	Ascomycota	Sordariomycetes	Incertae	Incertae	*Myrmecridium*	3E-39	163	124	91.94	2	8
OTU765	AB615578	Fungi	Ascomycota	Sordariomycetes	Incertae	Plectosphaerellaceae	*Plectosphaerella*	4E-31	135	68	100	0	0
OTU167	AB520352	Fungi	Ascomycota	Sordariomycetes	Incertae	Plectosphaerellaceae	*Verticillium*	4E-53	208	117	98.29	1	1
OTU489	AJ582964	Fungi	Ascomycota	Sordariomycetes	Microascales	Halosphaeriaceae	unidentified	2E-106	387	223	98.21	3	3
OTU191	JN905220	Fungi	Ascomycota	Sordariomycetes	Microascales	Microascaceae	*Kernia*	1E-31	137	116	91.38	2	3
OTU189	KM100715	Fungi	Ascomycota	Sordariomycetes	Microascales	Microascaceae	*Kernia*	6E-68	258	154	97.4	2	2
OTU241	GU981736	Fungi	Ascomycota	Sordariomycetes	Microascales	Microascaceae	*Kernia*	1E-91	337	214	96.26	3	3
OTU327	AY254153	Fungi	Ascomycota	Sordariomycetes	Microascales	Microascaceae	*Kernia*	1E-57	224	169	95.86	7	7
OTU264	KJ572145	Fungi	Ascomycota	Sordariomycetes	Microascales	Microascaceae	*Kernia*	3E-73	276	147	98.64	0	0
OTU501	JQ936271	Fungi	Ascomycota	Sordariomycetes	Microascales	Microascaceae	*Kernia*	1E-75	283	195	93.85	2	6
OTU381	GQ999538	Fungi	Ascomycota	Sordariomycetes	Microascales	Microascaceae	*Microascus*	2E-44	180	103	98.06	1	1
OTU423	JX524287	Fungi	Ascomycota	Sordariomycetes	Microascales	Microascaceae	*Microascus*	8E-52	204	155	93.55	3	3
OTU235	NR_073231	Fungi	Ascomycota	Sordariomycetes	Microascales	Microascaceae	*Microascus*	2E-51	202	110	98.18	0	0
OTU26	AB255241	Fungi	Ascomycota	Sordariomycetes	Phyllachorales	Phyllachoraceae	*Phyllachora*	9E-64	244	147	97.28	2	2
OTU311	KJ572129	Fungi	Ascomycota	Sordariomycetes	Sordariales	Chaetomiaceae	*Chaetomium*	5E-59	228	142	97.18	3	4
OTU312	KJ572205	Fungi	Ascomycota	Sordariomycetes	Sordariales	Chaetomiaceae	*Chaetomium*	3E-114	412	208	100	0	0

OTU500	JF945390	Fungi	Ascomycota	Sordariomycetes	Sordariales	Chaetomiaceae	*Chaetomium*	2E-55	216	125	98.4	2	2
OTU524	JX014309	Fungi	Ascomycota	Sordariomycetes	Sordariales	Chaetomiaceae	*Chaetomium*	1E-73	278	176	96.59	3	3
OTU364	HM992800	Fungi	Ascomycota	Sordariomycetes	Sordariales	Lasiosphaeriaceae	*Cercophora*	5E-91	335	228	94.3	2	3
OTU337	JX966555	Fungi	Ascomycota	Sordariomycetes	Sordariales	Sordariaceae	*Neurospora*	2E-68	260	175	94.29	1	1
OTU784	HM992800	Fungi	Ascomycota	Sordariomycetes	Trichosphaeriales	Incertae	*Nigrospora*	1E-88	327	225	94.67	3	3
OTU529	KC507224	Fungi	Ascomycota	Sordariomycetes	Trichosphaeriales	Incertae	*Nigrospora*	5E-65	248	137	97.81	0	0
OTU552	KJ957778	Fungi	Ascomycota	Sordariomycetes	Trichosphaeriales	Incertae	*Nigrospora*	9E-64	244	150	97.33	3	4
OTU814	NR_073264	Fungi	Ascomycota	Sordariomycetes	Trichosphaeriales	Incertae	*Nigrospora*	3E-20	99.6	102	88.24	1	1
OTU767	AB980801	Fungi	Ascomycota	Sordariomycetes	Trichosphaeriales	Incertae	*Nigrospora*	1E-84	313	162	99.38	0	0
OTU793	JX649198	Fungi	Ascomycota	Sordariomycetes	Trichosphaeriales	Incertae	*Nigrospora*	4E-23	109	118	88.14	2	3
OTU71	KF146177	Fungi	Ascomycota	Sordariomycetes	Trichosphaeriales	Incertae	*Nigrospora*	8E-102	371	199	98.49	0	0
OTU172	KC354795	Fungi	Ascomycota	Sordariomycetes	Trichosphaeriales	Incertae	*Nigrospora*	3E-67	256	157	96.18	1	1
OTU111	AB769904	Fungi	Ascomycota	Sordariomycetes	Trichosphaeriales	Incertae	*Nigrospora*	2E-106	387	234	96.58	2	3
OTU136	HM992800	Fungi	Ascomycota	Sordariomycetes	Trichosphaeriales	Incertae	*Nigrospora*	3E-89	329	229	94.76	4	5
OTU719	KF269234	Fungi	Ascomycota	Sordariomycetes	Trichosphaeriales	Incertae	*Nigrospora*	3E-79	295	149	100	0	0
OTU779	FJ776691	Fungi	Ascomycota	Sordariomycetes	Trichosphaeriales	Incertae	*Nigrospora*	2E-62	240	157	96.82	4	4
OTU797	KC193592	Fungi	Ascomycota	Sordariomycetes	Trichosphaeriales	Incertae	*Nigrospora*	2E-87	323	202	96.53	3	4
OTU550	KF156293	Fungi	Ascomycota	Sordariomycetes	Trichosphaeriales	Incertae	*Nigrospora*	3E-73	276	143	99.3	0	0
OTU338	JX967397	Fungi	Ascomycota	Sordariomycetes	Trichosphaeriales	Incertae	*Nigrospora*	3E-47	188	95	100	0	0
OTU314	FJ917556	Fungi	Ascomycota	Sordariomycetes	Trichosphaeriales	Incertae	*Nigrospora*	1E-97	357	188	98.94	0	0
OTU107	DQ530216	Fungi	Ascomycota	Sordariomycetes	Trichosphaeriales	Incertae	*Nigrospora*	4E-38	159	140	90.71	3	7
OTU23	KM246756	Fungi	Ascomycota	Sordariomycetes	Trichosphaeriales	Incertae	*Nigrospora*	1E-78	293	176	96.59	1	1
OTU736	FN565296	Fungi	Ascomycota	Sordariomycetes	Trichosphaeriales	Incertae	*Nigrospora*	1E-62	240	149	96.64	2	2
OTU745	JQ038351	Fungi	Ascomycota	Sordariomycetes	Trichosphaeriales	Incertae	*Nigrospora*	6E-68	258	144	97.22	0	0
OTU750	KC491368	Fungi	Ascomycota	Sordariomycetes	Trichosphaeriales	Incertae	*Nigrospora*	3E-40	167	204	87.75	5	5
OTU794	JX976056	Fungi	Ascomycota	Sordariomycetes	Trichosphaeriales	Incertae	*Nigrospora*	3E-92	339	195	97.95	2	2

OTU28	DQ914681	Fungi	Ascomycota	Sordariomycetes	Trichosphaeriales	Incertae	*Nigrospora*	4E-51	202	162	92.59	3	3
OTU142	KJ608106	Fungi	Ascomycota	Sordariomycetes	Trichosphaeriales	Incertae	*Nigrospora*	4E-56	218	126	98.41	2	2
OTU555	NR_073338	Fungi	Ascomycota	Sordariomycetes	Trichosphaeriales	Incertae	*Nigrospora*	1E-28	127	118	90.68	3	5
OTU747	JX280781	Fungi	Ascomycota	Sordariomycetes	Trichosphaeriales	Incertae	*Nigrospora*	8E-86	317	160	100	0	0
OTU47	JQ825256	Fungi	Ascomycota	Sordariomycetes	Trichosphaeriales	Incertae	*Nigrospora*	1E-49	196	99	100	0	0
OTU504	KM063249	Fungi	Ascomycota	Sordariomycetes	Trichosphaeriales	Incertae	*Nigrospora*	1E-72	274	142	99.3	0	0
OTU3	EU686935	Fungi	Ascomycota	Sordariomycetes	unidentified	unidentified	unidentified	3E-20	99.6	94	89.36	1	1
OTU9	EU686935	Fungi	Ascomycota	Sordariomycetes	unidentified	unidentified	unidentified	3E-20	99.6	94	89.36	1	1
OTU56	KJ471524	Fungi	Ascomycota	Sordariomycetes	unidentified	unidentified	unidentified	2E-80	299	155	99.35	0	0
OTU272	JN905220	Fungi	Ascomycota	Sordariomycetes	unidentified	unidentified	unidentified	5E-40	165	115	93.04	0	0
OTU73	JX624261	Fungi	Ascomycota	Sordariomycetes	unidentified	unidentified	unidentified	2E-68	260	135	99.26	0	0
OTU15	JX436244	Fungi	Ascomycota	Sordariomycetes	unidentified	unidentified	unidentified	3E-73	276	167	97.6	3	3
OTU128	EF488417	Fungi	Ascomycota	Sordariomycetes	unidentified	unidentified	unidentified	1E-88	327	181	98.34	1	1
OTU174	JN905220	Fungi	Ascomycota	Sordariomycetes	unidentified	unidentified	unidentified	8E-39	161	117	94.02	2	2
OTU456	DQ530216	Fungi	Ascomycota	Sordariomycetes	unidentified	unidentified	unidentified	4E-35	149	146	91.78	6	7
OTU118	UDB018208	Fungi	Ascomycota	Sordariomycetes	unidentified	unidentified	unidentified	3E-123	442	239	98.74	1	1
OTU238	GU053830	Fungi	Ascomycota	Sordariomycetes	Xylariales	Amphisphaeriaceae	*Pestalotiopsis*	1E-39	165	159	90.57	4	4
OTU583	UDB019703	Fungi	Ascomycota	Sordariomycetes	Xylariales	Diatrypaceae	*Eutypa*	7E-103	375	237	96.62	4	4
OTU244	JX014309	Fungi	Ascomycota	Sordariomycetes	Xylariales	Diatrypaceae	*Eutypa*	5E-48	192	101	99.01	0	0
OTU530	KJ869112	Fungi	Ascomycota	Sordariomycetes	Xylariales	Diatrypaceae	*Eutypa*	2E-64	246	155	95.48	1	2
OTU192	JQ520177	Fungi	Ascomycota	Sordariomycetes	Xylariales	Diatrypaceae	*Eutypa*	8E-99	361	194	98.97	1	1
OTU57	GQ999538	Fungi	Ascomycota	Sordariomycetes	Xylariales	Diatrypaceae	*Eutypa*	4E-39	163	101	96.04	1	2
OTU176	JQ520177	Fungi	Ascomycota	Sordariomycetes	Xylariales	Diatrypaceae	*Eutypa*	1E-88	327	204	97.06	4	5
OTU419	HM589327	Fungi	Ascomycota	Sordariomycetes	Xylariales	Diatrypaceae	*Eutypa*	8E-80	297	162	98.77	1	1
OTU388	KM458799	Fungi	Ascomycota	Sordariomycetes	Xylariales	Diatrypaceae	*Eutypa*	1E-91	337	178	99.44	1	1
OTU27	HE584882	Fungi	Ascomycota	Sordariomycetes	Xylariales	Diatrypaceae	*Eutypa*	2E-30	133	141	90.07	5	7

OTU245	AB615578	Fungi	Ascomycota	Sordariomycetes	Xylariales	Diatrypaceae	*Eutypa*	3E-32	139	74	98.65	0	0
OTU276	AM749926	Fungi	Ascomycota	Sordariomycetes	Xylariales	Diatrypaceae	*Eutypa*	3E-114	412	240	97.08	1	1
OTU559	AF492086	Fungi	Ascomycota	Sordariomycetes	Xylariales	Diatrypaceae	*Eutypa*	7E-93	341	176	99.43	0	0
OTU332	JQ348934	Fungi	Ascomycota	Sordariomycetes	Xylariales	Diatrypaceae	*Eutypa*	4E-85	315	183	98.36	3	3
OTU252	AB900869	Fungi	Ascomycota	Sordariomycetes	Xylariales	Diatrypaceae	*Eutypa*	2E-73	276	139	100	0	0
OTU373	KF146177	Fungi	Ascomycota	Sordariomycetes	Xylariales	Diatrypaceae	*Eutypella*	7E-90	331	203	97.54	4	4
OTU324	JF495181	Fungi	Ascomycota	Sordariomycetes	Xylariales	Diatrypaceae	*Libertella*	7E-52	204	139	94.24	1	1
OTU99	KM527126	Fungi	Ascomycota	Sordariomycetes	Xylariales	Diatrypaceae	*Libertella*	6E-65	248	141	98.58	2	2
OTU537	EF419902	Fungi	Ascomycota	Sordariomycetes	Xylariales	Diatrypaceae	*Libertella*	2E-87	323	182	97.8	1	2
OTU195	AB255241	Fungi	Ascomycota	Sordariomycetes	Xylariales	Diatrypaceae	*Libertella*	1E-66	254	136	99.26	1	1
OTU72	U65613	Fungi	Ascomycota	Sordariomycetes	Xylariales	Diatrypaceae	*Libertella*	4E-69	262	140	98.57	0	0
OTU319	JQ936271	Fungi	Ascomycota	Sordariomycetes	Xylariales	Diatrypaceae	*Libertella*	4E-91	335	192	97.92	2	3
OTU29	FJ770078	Fungi	Ascomycota	Sordariomycetes	Xylariales	Diatrypaceae	*Libertella*	3E-23	109	139	86.33	2	2
OTU25	JN596348	Fungi	Ascomycota	Sordariomycetes	Xylariales	Diatrypaceae	*Libertella*	7E-74	278	152	98.68	1	1
OTU126	KJ170305	Fungi	Ascomycota	Sordariomycetes	Xylariales	Diatrypaceae	*Libertella*	5E-59	228	142	95.77	1	2
OTU93	KC857282	Fungi	Ascomycota	Sordariomycetes	Xylariales	Diatrypaceae	*Libertella*	3E-70	266	154	98.05	2	2
OTU536	DQ682585	Fungi	Ascomycota	Sordariomycetes	Xylariales	Diatrypaceae	unidentified	3E-57	222	131	97.71	2	3
OTU494	FJ776641	Fungi	Ascomycota	Sordariomycetes	Xylariales	Diatrypaceae	unidentified	1E-81	303	157	99.36	0	0
OTU341	FJ627262	Fungi	Ascomycota	Sordariomycetes	Xylariales	Diatrypaceae	unidentified	7E-87	321	198	96.46	2	2
OTU425	KF705019	Fungi	Ascomycota	Sordariomycetes	Xylariales	Diatrypaceae	unidentified	2E-112	406	217	99.08	1	1
OTU182	AB693819	Fungi	Ascomycota	Sordariomycetes	Xylariales	Incertae	*Dinemasporium*	4E-110	398	209	99.04	0	0
OTU498	HQ846974	Fungi	Ascomycota	Sordariomycetes	Xylariales	unidentified	unidentified	8E-115	414	236	97.46	1	2
OTU296	KJ957778	Fungi	Ascomycota	Sordariomycetes	Xylariales	Xylariaceae	*Annulohypoxylon*	9E-61	234	150	97.33	4	4
OTU484	KM527126	Fungi	Ascomycota	Sordariomycetes	Xylariales	Xylariaceae	*Hypoxylon*	8E-55	214	139	94.96	1	2
OTU46	KP132554	Fungi	Ascomycota	Sordariomycetes	Xylariales	Xylariaceae	*Hypoxylon*	1E-66	254	176	94.32	2	2
OTU141	FJ917556	Fungi	Ascomycota	Sordariomycetes	Xylariales	Xylariaceae	*Hypoxylon*	8E-102	371	191	99.48	0	0

OTU469	FJ596861	Fungi	Ascomycota	Sordariomycetes	Xylariales	Xylariaceae	*Hypoxylon*	1E-91	337	198	97.47	2	2
OTU474	JX162758	Fungi	Ascomycota	Sordariomycetes	Xylariales	Xylariaceae	*Hypoxylon*	1E-100	367	193	98.96	0	0
OTU202	HE584882	Fungi	Ascomycota	Sordariomycetes	Xylariales	Xylariaceae	*Hypoxylon*	3E-45	182	147	91.84	2	3
OTU483	KM457634	Fungi	Ascomycota	Sordariomycetes	Xylariales	Xylariaceae	*Hypoxylon*	1E-103	377	190	100	0	0
OTU293	KF212308	Fungi	Ascomycota	Sordariomycetes	Xylariales	Xylariaceae	*Hypoxylon*	1E-75	283	159	98.74	2	2
OTU488	AF191548	Fungi	Ascomycota	Sordariomycetes	Xylariales	Xylariaceae	*Hypoxylon*	1E-84	313	166	99.4	1	1
OTU430	GQ511904	Fungi	Ascomycota	Sordariomycetes	Xylariales	Xylariaceae	*Hypoxylon*	8E-42	170	114	94.74	1	1
OTU137	KM246204	Fungi	Ascomycota	Sordariomycetes	Xylariales	Xylariaceae	*Xylaria*	1E-69	264	148	97.97	1	2
OTU437	KF286985	Fungi	Ascomycota	Sordariomycetes	Xylariales	Xylariaceae	*Xylaria*	6E-25	115	122	89.34	3	3
OTU467	DQ914681	Fungi	Ascomycota	Sordariomycetes	Xylariales	Xylariaceae	*Xylaria*	2E-43	176	169	90.53	4	4
OTU382	HE584827	Fungi	Ascomycota	Sordariomycetes	Xylariales	Xylariaceae	*Xylaria*	3E-21	103	60	96.67	0	0
OTU203	HQ631032	Fungi	Ascomycota	Sordariomycetes	Xylariales	Xylariaceae	*Xylaria*	4E-69	262	156	96.79	1	1
OTU91	KC978007	Fungi	Ascomycota	Sordariomycetes	Xylariales	Xylariaceae	*Xylaria*	3E-86	319	220	94.55	3	4
OTU36	JX014309	Fungi	Ascomycota	Sordariomycetes	Xylariales	Xylariaceae	*Xylaria*	3E-108	392	226	97.79	2	2
OTU59	KC857282	Fungi	Ascomycota	Sordariomycetes	Xylariales	Xylariaceae	*Xylaria*	1E-69	264	153	97.39	1	1
OTU50	GQ999538	Fungi	Ascomycota	Sordariomycetes	Xylariales	Xylariaceae	*Xylaria*	2E-35	151	108	96.3	4	4
OTU436	KC978007	Fungi	Ascomycota	Sordariomycetes	Xylariales	Xylariaceae	*Xylaria*	8E-93	341	219	96.35	4	5
OTU395	KC491368	Fungi	Ascomycota	Taphrinomycetes	Taphrinales	Taphrinaceae	*Taphrina*	3E-74	280	225	92.44	4	4
OTU575	KF516949	Fungi	Ascomycota	unidentified	unidentified	unidentified	unidentified	4E-72	272	161	97.52	2	2
OTU368	KF212308	Fungi	Ascomycota	unidentified	unidentified	unidentified	unidentified	8E-74	278	168	97.02	2	2
OTU652	GQ999538	Fungi	Basidiomycota	Agaricomycetes	Agaricales	Psathyrellaceae	*Coprinellus*	7E-38	159	80	100	0	0
OTU718	KF212196	Fungi	Basidiomycota	Agaricomycetes	Agaricales	Psathyrellaceae	*Coprinellus*	6E-109	394	199	100	0	0
OTU658	HQ631017	Fungi	Basidiomycota	Agaricomycetes	Agaricales	Psathyrellaceae	*Coprinopsis*	1E-38	161	139	92.09	4	6
OTU654	HE584827	Fungi	Basidiomycota	Agaricomycetes	Agaricales	Psathyrellaceae	*Coprinopsis*	2E-22	107	164	86.59	6	8
OTU622	JX317505	Fungi	Basidiomycota	Agaricomycetes	Agaricales	Psathyrellaceae	*Psathyrella*	1E-22	107	113	89.38	3	4
OTU589	FJ820729	Fungi	Basidiomycota	Agaricomycetes	Agaricales	Strophariaceae	*Hypholoma*	4E-57	222	169	93.49	4	7

OTU629	KC978007	Fungi	Basidiomycota	Agaricomycetes	Agaricales	Strophariaceae	*Hypholoma*	1E-107	391	221	98.64	3	3
OTU645	AF443929	Fungi	Basidiomycota	Agaricomycetes	Agaricales	Strophariaceae	*Psilocybe*	2E-96	353	209	97.61	3	4
OTU671	KC191577	Fungi	Basidiomycota	Agaricomycetes	Agaricales	Strophariaceae	*Stropharia*	1E-32	141	178	87.64	5	6
OTU676	KF222008	Fungi	Basidiomycota	Agaricomycetes	Agaricales	Strophariaceae	*Stropharia*	5E-22	105	53	100	0	0
OTU667	JX966555	Fungi	Basidiomycota	Agaricomycetes	Agaricales	Strophariaceae	*Stropharia*	6E-69	262	199	93.47	4	5
OTU640	AB218068	Fungi	Basidiomycota	Agaricomycetes	Boletales	Rhizopogonaceae	*Rhizopogon*	6E-90	331	179	98.88	1	1
OTU587	EU035415	Fungi	Basidiomycota	Agaricomycetes	Boletales	Rhizopogonaceae	*Rhizopogon*	1E-60	234	198	91.41	4	8
OTU700	HM437870	Fungi	Basidiomycota	Agaricomycetes	Corticiales	Corticiaceae	*Limonomyces*	1E-84	313	158	100	0	0
OTU687	AF324170	Fungi	Basidiomycota	Agaricomycetes	Phallales	Phallaceae	*Phallus*	4E-54	212	146	95.21	3	4
OTU709	JX162767	Fungi	Basidiomycota	Agaricomycetes	Polyporales	Ganodermataceae	*Ganoderma*	4E-88	325	200	96.5	2	2
OTU577	KF984795	Fungi	Basidiomycota	Agaricomycetes	Polyporales	Ganodermataceae	*Ganoderma*	7E-96	351	181	99.45	0	0
OTU613	GU366712	Fungi	Basidiomycota	Agaricomycetes	Polyporales	Ganodermataceae	*Ganoderma*	1E-81	303	153	100	0	0
OTU635	KM111293	Fungi	Basidiomycota	Agaricomycetes	Polyporales	Ganodermataceae	*Ganoderma*	1E-66	254	189	93.12	3	6
OTU625	JX624261	Fungi	Basidiomycota	Agaricomycetes	Polyporales	Ganodermataceae	*Ganoderma*	2E-49	196	150	93.33	3	4
OTU572	JX014309	Fungi	Basidiomycota	Agaricomycetes	Polyporales	Ganodermataceae	*Ganoderma*	6E-97	355	203	98.03	2	2
OTU580	KM246756	Fungi	Basidiomycota	Agaricomycetes	Polyporales	Ganodermataceae	*Ganoderma*	1E-78	293	179	97.21	3	4
OTU592	JF945479	Fungi	Basidiomycota	Agaricomycetes	Polyporales	Ganodermataceae	*Ganoderma*	8E-37	155	173	87.86	3	4
OTU593	JN906117	Fungi	Basidiomycota	Agaricomycetes	Polyporales	Ganodermataceae	*Ganoderma*	1E-62	240	145	97.93	3	3
OTU646	DQ068346	Fungi	Basidiomycota	Agaricomycetes	Polyporales	Ganodermataceae	*Ganoderma*	2E-40	167	92	97.83	0	0
OTU637	KM265670	Fungi	Basidiomycota	Agaricomycetes	Polyporales	Ganodermataceae	*Ganoderma*	9E-61	234	142	96.48	1	1
OTU633	KM016925	Fungi	Basidiomycota	Agaricomycetes	Polyporales	Ganodermataceae	*Ganoderma*	4E-75	281	150	99.33	1	1
OTU627	KC478551	Fungi	Basidiomycota	Agaricomycetes	Polyporales	Ganodermataceae	*Ganoderma*	1E-72	274	142	99.3	0	0
OTU706	JQ520177	Fungi	Basidiomycota	Agaricomycetes	Polyporales	Ganodermataceae	*Ganoderma*	8E-102	371	195	99.49	1	1
OTU342	FJ972957	Fungi	Basidiomycota	Agaricomycetes	Polyporales	Polyporaceae	*Perenniporia*	1E-78	293	164	98.17	1	1
OTU394	JX014309	Fungi	Basidiomycota	Agaricomycetes	Polyporales	Polyporaceae	*Perenniporia*	8E-90	331	225	94.67	3	5
OTU205	FJ820729	Fungi	Basidiomycota	Agaricomycetes	Polyporales	Polyporaceae	*Perenniporia*	6E-56	218	166	94.58	5	5

OTU7	FJ627262	Fungi	Basidiomycota	Agaricomycetes	Polyporales	Polyporaceae	*Perenniporia*	1E-79	297	202	95.54	4	4
OTU383	JN905220	Fungi	Basidiomycota	Agaricomycetes	Polyporales	Polyporaceae	*Perenniporia*	2E-33	143	116	92.24	2	2
OTU343	JX043185	Fungi	Basidiomycota	Agaricomycetes	Polyporales	Polyporaceae	*Perenniporia*	7E-28	125	134	88.06	2	3
OTU78	KF212308	Fungi	Basidiomycota	Agaricomycetes	Polyporales	Polyporaceae	*Perenniporia*	2E-86	319	165	99.39	0	0
OTU539	EU035415	Fungi	Basidiomycota	Agaricomycetes	Polyporales	Polyporaceae	*Perenniporia*	3E-55	216	196	90.82	5	10
OTU248	HQ631032	Fungi	Basidiomycota	Agaricomycetes	Polyporales	Polyporaceae	*Perenniporia*	3E-73	276	158	98.1	2	3
OTU540	FJ627262	Fungi	Basidiomycota	Agaricomycetes	Polyporales	Polyporaceae	*Perenniporia*	1E-82	307	202	95.54	3	4
OTU378	AF444483	Fungi	Basidiomycota	Agaricomycetes	Polyporales	Polyporaceae	*Perenniporia*	2E-20	99.6	74	93.24	1	1
OTU179	KF916572	Fungi	Basidiomycota	Agaricomycetes	Polyporales	Polyporaceae	*Perenniporia*	3E-50	198	108	99.07	1	1
OTU240	EU686935	Fungi	Basidiomycota	Agaricomycetes	Polyporales	Polyporaceae	*Perenniporia*	1E-22	107	94	90.43	1	1
OTU5	FJ627262	Fungi	Basidiomycota	Agaricomycetes	Polyporales	Polyporaceae	*Perenniporia*	8E-99	361	202	98.51	2	2
OTU226	JQ418381	Fungi	Basidiomycota	Agaricomycetes	Russulales	Peniophoraceae	*Peniophora*	1E-50	200	137	94.89	2	2
OTU681	KJ572145	Fungi	Basidiomycota	Agaricomycetes	Russulales	unidentified	unidentified	4E-75	281	142	100	0	0
OTU683	KM246756	Fungi	Basidiomycota	Agaricomycetes	Sebacinales	Sebacinaceae	*Sebacina*	5E-75	281	181	96.13	3	4
OTU675	KC978007	Fungi	Basidiomycota	Agaricomycetes	Sebacinales	Sebacinaceae	*Sebacina*	4E-104	379	219	97.26	1	1
OTU615	HM992800	Fungi	Basidiomycota	Agaricomycetes	Trechisporales	Hydnodontaceae	*Trechispora*	2E-66	254	178	94.38	3	5
OTU603	KP131527	Fungi	Basidiomycota	Agaricomycetes	Trechisporales	Hydnodontaceae	unidentified	1E-75	283	167	98.2	3	3
OTU712	JX974764	Fungi	Basidiomycota	Agaricomycetes	unidentified	unidentified	unidentified	9E-23	107	81	92.59	1	2
OTU648	EU686935	Fungi	Basidiomycota	Agaricomycetes	unidentified	unidentified	unidentified	1E-22	107	94	90.43	1	1
OTU665	JX624261	Fungi	Basidiomycota	Agaricostilbomycetes	Agaricostilbales	Agaricostilbaceae	*Bensingtonia*	8E-55	214	149	93.96	2	5
OTU685	KP132554	Fungi	Basidiomycota	Cystobasidiomycetes	Cystobasidiales	Cystobasidiaceae	*Occultifur*	2E-96	353	194	98.97	2	2
OTU586	AB998278	Fungi	Basidiomycota	Cystobasidiomycetes	Cystobasidiales	Cystobasidiaceae	*Occultifur*	1E-75	283	154	98.7	1	2
OTU566	GQ999538	Fungi	Basidiomycota	Cystobasidiomycetes	Erythrobasidiales	Erythrobasidiaceae	*Erythrobasidium*	5E-48	192	101	99.01	0	0
OTU620	JQ905732	Fungi	Basidiomycota	Cystobasidiomycetes	Erythrobasidiales	Erythrobasidiaceae	*Erythrobasidium*	2E-61	236	139	97.84	2	2
OTU639	NR_073218	Fungi	Basidiomycota	Cystobasidiomycetes	Erythrobasidiales	Erythrobasidiaceae	*Erythrobasidium*	3E-47	188	103	98.06	0	0
OTU663	JX014309	Fungi	Basidiomycota	Cystobasidiomycetes	Erythrobasidiales	Erythrobasidiaceae	*Erythrobasidium*	2E-81	303	157	99.36	0	0

OTU601	KM063240	Fungi	Basidiomycota	Cystobasidiomycetes	Erythrobasidiales	Erythrobasidiaceae	*Erythrobasidium*	5E-57	222	207	92.75	9	10
OTU668	JX978429	Fungi	Basidiomycota	Cystobasidiomycetes	Erythrobasidiales	Erythrobasidiaceae	*Erythrobasidium*	1E-54	214	175	92	3	4
OTU651	GQ999325	Fungi	Basidiomycota	Cystobasidiomycetes	Erythrobasidiales	Erythrobasidiaceae	*Erythrobasidium*	3E-92	339	187	98.93	2	2
OTU611	GQ999538	Fungi	Basidiomycota	Cystobasidiomycetes	Erythrobasidiales	Erythrobasidiaceae	*Erythrobasidium*	7E-38	159	80	100	0	0
OTU696	GQ518230	Fungi	Basidiomycota	Cystobasidiomycetes	Erythrobasidiales	Erythrobasidiaceae	*Erythrobasidium*	3E-29	129	124	90.32	3	4
OTU634	KM103661	Fungi	Basidiomycota	Cystobasidiomycetes	Erythrobasidiales	Erythrobasidiaceae	*Erythrobasidium*	7E-77	287	153	98.69	0	0
OTU576	KF800162	Fungi	Basidiomycota	Cystobasidiomycetes	Erythrobasidiales	Erythrobasidiaceae	*Erythrobasidium*	2E-103	377	190	100	0	0
OTU692	EF419971	Fungi	Basidiomycota	Cystobasidiomycetes	Erythrobasidiales	Erythrobasidiaceae	*Erythrobasidium*	4E-69	262	144	97.92	0	0
OTU585	AB255241	Fungi	Basidiomycota	Cystobasidiomycetes	Erythrobasidiales	Erythrobasidiaceae	*Erythrobasidium*	9E-58	224	137	97.81	3	3
OTU595	KF143787	Fungi	Basidiomycota	Cystobasidiomycetes	Erythrobasidiales	Erythrobasidiaceae	*Erythrobasidium*	5E-94	345	194	98.45	2	2
OTU684	KP099830	Fungi	Basidiomycota	Cystobasidiomycetes	Erythrobasidiales	Erythrobasidiaceae	*Erythrobasidium*	6E-20	99.6	58	96.55	0	0
OTU643	AB693792	Fungi	Basidiomycota	Cystobasidiomycetes	Erythrobasidiales	Erythrobasidiaceae	*Erythrobasidium*	3E-63	242	134	98.51	1	1
OTU672	KC354795	Fungi	Basidiomycota	Cystobasidiomycetes	Erythrobasidiales	Erythrobasidiaceae	*Erythrobasidium*	2E-46	186	147	92.52	3	6
OTU699	HE584827	Fungi	Basidiomycota	Cystobasidiomycetes	Erythrobasidiales	Erythrobasidiaceae	*Erythrobasidium*	3E-30	133	173	87.28	5	7
OTU666	JX898596	Fungi	Basidiomycota	Cystobasidiomycetes	Erythrobasidiales	Erythrobasidiaceae	*Erythrobasidium*	5E-75	281	170	95.88	0	0
OTU703	JN205932	Fungi	Basidiomycota	Cystobasidiomycetes	Erythrobasidiales	Erythrobasidiaceae	*Erythrobasidium*	1E-107	391	209	99.04	1	1
OTU579	KJ572145	Fungi	Basidiomycota	Cystobasidiomycetes	Erythrobasidiales	Erythrobasidiaceae	*Erythrobasidium*	4E-69	262	144	98.61	1	1
OTU679	KF675509	Fungi	Basidiomycota	Cystobasidiomycetes	Erythrobasidiales	Erythrobasidiaceae	*Erythrobasidium*	9E-61	234	146	95.89	1	1
OTU628	KC753419	Fungi	Basidiomycota	Cystobasidiomycetes	Erythrobasidiales	unidentified	unidentified	4E-44	178	142	92.25	2	2
OTU653	GU214635	Fungi	Basidiomycota	Cystobasidiomycetes	Erythrobasidiales	unidentified	unidentified	4E-35	149	135	91.11	3	3
OTU644	AF314984	Fungi	Basidiomycota	Microbotryomycetes	Sporidiobolales	Incertae	*Rhodotorula*	2E-87	323	186	97.85	2	3
OTU647	DQ530216	Fungi	Basidiomycota	Microbotryomycetes	Sporidiobolales	Incertae	*Rhodotorula*	2E-31	137	145	91.72	7	7
OTU590	GU214635	Fungi	Basidiomycota	Microbotryomycetes	Sporidiobolales	Incertae	*Rhodotorula*	1E-22	107	144	86.81	4	6
OTU612	GU214635	Fungi	Basidiomycota	Microbotryomycetes	Sporidiobolales	Incertae	*Rhodotorula*	3E-36	153	136	91.18	3	4
OTU624	JX624261	Fungi	Basidiomycota	Microbotryomycetes	Sporidiobolales	Incertae	*Rhodotorula*	3E-70	266	142	99.3	1	1
OTU686	KP132554	Fungi	Basidiomycota	Microbotryomycetes	Sporidiobolales	Incertae	*Rhodotorula*	2E-80	299	175	96.57	0	0

OTU678	KF293978	Fungi	Basidiomycota	Microbotryomycetes	Sporidiobolales	Incertae	*Rhodotorula*	2E-68	260	143	98.6	1	1
OTU799	KC455241	Fungi	Basidiomycota	Microbotryomycetes	Sporidiobolales	Incertae	*Rhodotorula*	5E-60	232	185	92.43	4	8
OTU605	UDB015716	Fungi	Basidiomycota	Microbotryomycetes	Sporidiobolales	Incertae	*Rhodotorula*	1E-85	317	244	93.44	5	5
OTU602	KM248556	Fungi	Basidiomycota	Microbotryomycetes	Sporidiobolales	Incertae	*Rhodotorula*	2E-55	216	136	95.59	1	2
OTU621	JX268524	Fungi	Basidiomycota	Microbotryomycetes	Sporidiobolales	Incertae	*Rhodotorula*	2E-71	270	176	96.59	4	4
OTU649	FJ969800	Fungi	Basidiomycota	Microbotryomycetes	Sporidiobolales	Incertae	*Rhodotorula*	5E-32	139	156	89.1	5	7
OTU720	KF901810	Fungi	Basidiomycota	Microbotryomycetes	Sporidiobolales	Incertae	*Rhodotorula*	8E-49	194	150	92.67	2	2
OTU670	KC182055	Fungi	Basidiomycota	Microbotryomycetes	Sporidiobolales	Incertae	*Rhodotorula*	1E-57	224	161	96.27	6	6
OTU574	KC525844	Fungi	Basidiomycota	Microbotryomycetes	Sporidiobolales	Incertae	*Rhodotorula*	2E-74	280	141	100	0	0
OTU591	HE584882	Fungi	Basidiomycota	Microbotryomycetes	Sporidiobolales	Incertae	*Rhodotorula*	7E-40	165	126	93.65	3	4
OTU655	HE998744	Fungi	Basidiomycota	Microbotryomycetes	Sporidiobolales	Incertae	*Rhodotorula*	8E-49	194	152	92.11	2	4
OTU659	HQ914866	Fungi	Basidiomycota	Microbotryomycetes	Sporidiobolales	Incertae	*Sporobolomyces*	3E-54	212	135	96.3	2	2
OTU669	JX998699	Fungi	Basidiomycota	Microbotryomycetes	Sporidiobolales	Incertae	*Sporobolomyces*	5E-50	198	147	93.2	2	3
OTU702	JF718366	Fungi	Basidiomycota	Microbotryomycetes	Sporidiobolales	Incertae	*Sporobolomyces*	4E-88	325	172	99.42	1	1
OTU616	HM992800	Fungi	Basidiomycota	Microbotryomycetes	Sporidiobolales	Incertae	*Sporobolomyces*	5E-88	325	203	96.06	2	3
OTU708	JQ906769	Fungi	Basidiomycota	Pucciniomycetes	Septobasidiales	Septobasidiaceae	unidentified	5E-29	129	85	94.12	0	0
OTU726	U65610	Fungi	Basidiomycota	Pucciniomycetes	Septobasidiales	Septobasidiaceae	unidentified	3E-32	139	127	92.13	5	8
OTU739	GQ999538	Fungi	Basidiomycota	Tremellomycetes	Cystofilobasidiales	Cystofilobasidiaceae	*Guehomyces*	1E-51	204	103	100	0	0
OTU695	FJ438386	Fungi	Basidiomycota	Tremellomycetes	Cystofilobasidiales	Cystofilobasidiaceae	*Mrakia*	8E-90	331	218	96.33	5	6
OTU619	JN890113	Fungi	Basidiomycota	Tremellomycetes	Tremellales	Cuniculitremaceae	*Fellomyces*	9E-21	101	138	86.96	4	4
OTU661	JQ009310	Fungi	Basidiomycota	Tremellomycetes	Tremellales	Cuniculitremaceae	*Fellomyces*	1E-35	151	88	96.59	0	0
OTU673	KC857282	Fungi	Basidiomycota	Tremellomycetes	Tremellales	Cuniculitremaceae	*Fellomyces*	8E-49	194	155	92.26	3	6
OTU725	NR_073259	Fungi	Basidiomycota	Tremellomycetes	Tremellales	Cuniculitremaceae	*Fellomyces*	7E-27	121	97	91.75	1	1
OTU707	JQ747716	Fungi	Basidiomycota	Tremellomycetes	Tremellales	Cuniculitremaceae	unidentified	2E-90	333	180	98.33	0	0
OTU689	DQ322127	Fungi	Basidiomycota	Tremellomycetes	Tremellales	Cuniculitremaceae	unidentified	7E-38	159	92	97.83	1	1
OTU694	EU707864	Fungi	Basidiomycota	Tremellomycetes	Tremellales	Cuniculitremaceae	unidentified	4E-29	129	125	89.6	2	2

OTU656	HG004557	Fungi	Basidiomycota	Tremellomycetes	Tremellales	Cuniculitremaceae	unidentified	4E-78	291	147	100	0	0
OTU704	JN942309	Fungi	Basidiomycota	Tremellomycetes	Tremellales	Incertae	*Derxomyces*	2E-93	343	181	99.45	1	1
OTU604	KP132440	Fungi	Basidiomycota	Tremellomycetes	Tremellales	Incertae	*Hannaella*	2E-90	333	192	98.44	3	3
OTU618	JF945479	Fungi	Basidiomycota	Tremellomycetes	Tremellales	Incertae	*Hannaella*	7E-28	125	177	85.88	4	6
OTU650	FN548160	Fungi	Basidiomycota	Tremellomycetes	Tremellales	Incertae	*Hannaella*	4E-20	99.6	90	92.22	3	3
OTU594	JX998699	Fungi	Basidiomycota	Tremellomycetes	Tremellales	Incertae	*Hannaella*	2E-43	176	145	92.41	3	3
OTU570	JF502456	Fungi	Basidiomycota	Tremellomycetes	Tremellales	Incertae	*Hannaella*	1E-56	220	139	95.68	1	1
OTU598	KJ706359	Fungi	Basidiomycota	Tremellomycetes	Tremellales	Incertae	*Hannaella*	6E-71	268	143	99.3	1	1
OTU698	GU721275	Fungi	Basidiomycota	Tremellomycetes	Tremellales	Incertae	*Hannaella*	2E-54	212	107	100	0	0
OTU606	AM901803	Fungi	Basidiomycota	Tremellomycetes	Tremellales	Incertae		2E-68	260	143	97.9	0	0
OTU636	KM246756	Fungi	Basidiomycota	Tremellomycetes	Tremellales	Incertae		1E-84	313	166	99.4	1	1
OTU638	KP132554	Fungi	Basidiomycota	Tremellomycetes	Tremellales	Incertae		2E-74	280	177	96.05	2	2
OTU578	KJ082097	Fungi	Basidiomycota	Tremellomycetes	Tremellales	Tremellaceae	*Bullera*	1E-78	293	156	98.72	0	0
OTU600	KM051384	Fungi	Basidiomycota	Tremellomycetes	Tremellales	Tremellaceae	*Bullera*	8E-77	287	165	96.97	0	0
OTU569	HQ631022	Fungi	Basidiomycota	Tremellomycetes	Tremellales	Tremellaceae	*Bullera*	5E-100	365	200	98.5	1	1
OTU677	KF286985	Fungi	Basidiomycota	Tremellomycetes	Tremellales	Tremellaceae	*Bullera*	4E-20	99.6	126	88.1	4	4
OTU688	AF325173	Fungi	Basidiomycota	Tremellomycetes	Tremellales	Tremellaceae	*Bullera*	9E-48	190	104	98.08	0	0
OTU610	GQ999538	Fungi	Basidiomycota	Tremellomycetes	Tremellales	Tremellaceae	*Bullera*	8E-44	178	98	98.98	1	1
OTU614	HE774495	Fungi	Basidiomycota	Tremellomycetes	Tremellales	Tremellaceae	*Bullera*	1E-116	420	216	99.54	0	0
OTU680	KJ028784	Fungi	Basidiomycota	Tremellomycetes	Tremellales	Tremellaceae	*Bullera*	1E-113	410	247	97.57	4	4
OTU682	KM246204	Fungi	Basidiomycota	Tremellomycetes	Tremellales	Tremellaceae	*Bullera*	2E-55	216	141	95.04	1	1
OTU664	JX014309	Fungi	Basidiomycota	Tremellomycetes	Tremellales	Tremellaceae	*Cryptococcus*	1E-94	347	202	97.52	2	3
OTU660	JQ009310	Fungi	Basidiomycota	Tremellomycetes	Tremellales	Tremellaceae	*Cryptococcus*	1E-35	151	88	96.59	0	0
OTU607	DQ914681	Fungi	Basidiomycota	Tremellomycetes	Tremellales	Tremellaceae	*Cryptococcus*	5E-41	168	169	89.94	4	4
OTU657	HQ154258	Fungi	Basidiomycota	Tremellomycetes	Tremellales	Tremellaceae	*Cryptococcus*	9E-83	307	179	96.65	0	0
OTU630	KJ186952	Fungi	Basidiomycota	Tremellomycetes	Tremellales	Tremellaceae	*Cryptococcus*	4E-69	262	140	99.29	1	1

OTU701	HM487014	Fungi	Basidiomycota	Tremellomycetes	Tremellales	Tremellaceae	*Cryptococcus*	3E-36	153	144	92.36	6	7
OTU588	FJ810799	Fungi	Basidiomycota	Tremellomycetes	Tremellales	Tremellaceae	*Cryptococcus*	9E-83	307	171	98.25	1	1
OTU608	GQ511904	Fungi	Basidiomycota	Tremellomycetes	Tremellales	Tremellaceae	*Cryptococcus*	3E-29	129	115	92.17	4	6
OTU674	KC858999	Fungi	Basidiomycota	Tremellomycetes	Tremellales	Tremellaceae	*Cryptococcus*	4E-32	139	94	94.68	1	1
OTU617	HM992800	Fungi	Basidiomycota	Tremellomycetes	Tremellales	Tremellaceae	*Cryptococcus*	2E-109	396	228	98.25	3	3
OTU597	KJ619990	Fungi	Basidiomycota	Tremellomycetes	Tremellales	Tremellaceae	*Cryptococcus*	1E-97	357	184	99.46	0	0
OTU642	AB255305	Fungi	Basidiomycota	Tremellomycetes	Tremellales	Tremellaceae	*Cryptococcus*	7E-46	184	129	95.35	3	3
OTU641	AB255277	Fungi	Basidiomycota	Tremellomycetes	Tremellales	Tremellaceae	*Cryptococcus*	9E-64	244	150	97.33	3	4
OTU724	NR_073218	Fungi	Basidiomycota	Tremellomycetes	Tremellales	Tremellaceae	*Cryptococcus*	1E-49	196	103	99.03	0	0
OTU609	GQ522634	Fungi	Basidiomycota	Tremellomycetes	Tremellales	Tremellaceae	*Cryptococcus*	2E-118	426	247	96.76	0	0
OTU599	KM013444	Fungi	Basidiomycota	Tremellomycetes	Tremellales	Tremellaceae	*Cryptococcus*	1E-28	127	64	100	0	0
OTU705	JQ009308	Fungi	Basidiomycota	Tremellomycetes	Tremellales	Tremellaceae	*Cryptococcus*	2E-118	426	231	98.7	1	1
OTU626	KC354795	Fungi	Basidiomycota	Tremellomycetes	Tremellales	Tremellaceae	*Cryptococcus*	9E-58	224	149	95.3	2	2
OTU631	KJ775456	Fungi	Basidiomycota	Tremellomycetes	Tremellales	Tremellaceae	*Dioszegia*	5E-78	291	155	98.71	0	0
OTU623	JX502176	Fungi	Basidiomycota	Tremellomycetes	Tremellales	Tremellaceae	*Dioszegia*	5E-41	168	149	92.62	5	5
OTU717	KF001699	Fungi	Basidiomycota	Tremellomycetes	Tremellales	Tremellaceae	*Dioszegia*	7E-99	361	182	100	0	0
OTU596	KF984784	Fungi	Basidiomycota	Tremellomycetes	Tremellales	Tremellaceae	*Dioszegia*	2E-93	343	184	98.91	1	2
OTU697	GQ999423	Fungi	Basidiomycota	Tremellomycetes	Tremellales	Tremellaceae	*Trimorphomyces*	1E-23	111	190	87.37	10	12
OTU662	JQ009310	Fungi	Basidiomycota	Tremellomycetes	Tremellales	Tremellaceae	*Trimorphomyces*	2E-28	127	88	94.32	1	1
OTU568	HM992800	Fungi	Basidiomycota	Tremellomycetes	Tremellales	Tremellaceae	unidentified	2E-72	274	233	91.85	6	11
OTU693	EU520130	Fungi	Basidiomycota	Tremellomycetes	Tremellales	Tremellaceae	unidentified	6E-84	311	173	98.84	2	2
OTU584	Z81427	Fungi	Basidiomycota	Tremellomycetes	Tremellales	Tremellaceae	unidentified	4E-26	119	131	87.79	2	3
OTU565	FJ627262	Fungi	Basidiomycota	Tremellomycetes	Tremellales	Tremellaceae	unidentified	9E-71	268	205	93.66	5	7
OTU690	DQ530216	Fungi	Basidiomycota	Tremellomycetes	Tremellales	Tremellaceae	unidentified	4E-38	159	144	92.36	5	5
OTU691	DQ914681	Fungi	Basidiomycota	Tremellomycetes	Tremellales	Tremellaceae	unidentified	5E-35	149	170	88.24	4	5
OTU721	KJ494633	Fungi	Basidiomycota	Wallemiomycetes	Wallemiales	Wallemiaceae	*Wallemia*	6E-52	204	111	98.2	0	0

OTU632	KJ780808	Fungi	Basidiomycota	Wallemiomycetes	Wallemiales	Wallemiaceae	*Wallemia*	5E-81	301	156	99.36	0	0
OTU711	JX502176	Fungi	Chytridiomycota	Monoblepharidomycetes	Monoblepharidales	unidentified	unidentified	5E-38	159	132	93.18	4	4
OTU710	JX496034	Fungi	Chytridiomycota	unidentified	unidentified	unidentified	unidentified	2E-102	373	208	98.56	2	2
OTU713	KC311472	Fungi	Rozellomycota	unidentified	unidentified	unidentified	unidentified	8E-21	101	59	96.61	0	0
OTU782	HE998744	Fungi	unidentified	unidentified	unidentified	unidentified	unidentified	2E-34	147	149	90.6	5	6
OTU758	KM100715	Fungi	unidentified	unidentified	unidentified	unidentified	unidentified	2E-71	270	152	97.37	0	0
OTU763	AB255241	Fungi	unidentified	unidentified	unidentified	unidentified	unidentified	1E-66	254	136	99.26	1	1
OTU792	JX502176	Fungi	unidentified	unidentified	unidentified	unidentified	unidentified	6E-28	125	151	90.73	8	8
OTU774	DQ914681	Fungi	unidentified	unidentified	unidentified	unidentified	unidentified	5E-44	178	170	90.59	4	4
OTU816	U65610	Fungi	unidentified	unidentified	unidentified	unidentified	unidentified	2E-30	133	122	90.98	3	4
OTU787	JF497127	Fungi	unidentified	unidentified	unidentified	unidentified	unidentified	2E-96	353	178	100	0	0
OTU770	AF444483	Fungi	unidentified	unidentified	unidentified	unidentified	unidentified	1E-22	107	74	93.24	0	0
OTU735	FJ770078	Fungi	unidentified	unidentified	unidentified	unidentified	unidentified	4E-32	139	146	89.73	4	4
OTU761	KM265911	Fungi	unidentified	unidentified	unidentified	unidentified	unidentified	2E-53	210	106	100	0	0
OTU757	KM066193	Fungi	unidentified	unidentified	unidentified	unidentified	unidentified	7E-96	351	177	100	0	0
OTU776	FJ627262	Fungi	unidentified	unidentified	unidentified	unidentified	unidentified	2E-87	323	205	96.1	3	5
OTU756	KM051384	Fungi	unidentified	unidentified	unidentified	unidentified	unidentified	7E-65	248	157	96.18	2	2
OTU759	KM215637	Fungi	unidentified	unidentified	unidentified	unidentified	unidentified	4E-72	272	157	98.09	2	2
OTU738	GQ999538	Fungi	unidentified	unidentified	unidentified	unidentified	unidentified	2E-47	190	104	98.08	0	0
OTU743	HF680223	Fungi	unidentified	unidentified	unidentified	unidentified	unidentified	4E-47	188	119	95.8	1	1
OTU788	JF691121	Fungi	unidentified	unidentified	unidentified	unidentified	unidentified	6E-34	145	148	89.86	4	5
OTU741	GU966493	Fungi	unidentified	unidentified	unidentified	unidentified	unidentified	4E-29	129	97	93.81	2	2
OTU728	AB255241	Fungi	unidentified	unidentified	unidentified	unidentified	unidentified	1E-62	240	129	99.22	1	1
OTU754	KF269234	Fungi	unidentified	unidentified	unidentified	unidentified	unidentified	2E-52	206	154	94.81	5	7
OTU766	AB908129	Fungi	unidentified	unidentified	unidentified	unidentified	unidentified	8E-86	317	160	100	0	0
OTU730	AM176691	Fungi	unidentified	unidentified	unidentified	unidentified	unidentified	8E-61	234	141	97.16	2	3

OTU753	KF221297	Fungi	unidentified	unidentified	unidentified	unidentified	unidentified	3E-24	113	57	100	0	0
OTU748	KC354795	Fungi	unidentified	unidentified	unidentified	unidentified	unidentified	1E-50	200	160	93.12	4	5
OTU737	GQ999538	Fungi	unidentified	unidentified	unidentified	unidentified	unidentified	1E-51	204	103	100	0	0
OTU746	JQ418381	Fungi	unidentified	unidentified	unidentified	unidentified	unidentified	1E-44	180	139	94.24	4	4
OTU791	JQ666632	Fungi	unidentified	unidentified	unidentified	unidentified	unidentified	4E-41	168	132	93.18	3	4
OTU768	AB998515	Fungi	unidentified	unidentified	unidentified	unidentified	unidentified	1E-50	200	125	96	1	1
OTU729	AB998384	Fungi	unidentified	unidentified	unidentified	unidentified	unidentified	6E-68	258	134	99.25	0	0
OTU762	NR_073337	Fungi	unidentified	unidentified	unidentified	unidentified	unidentified	3E-23	109	105	89.52	2	4
OTU786	HQ914933	Fungi	unidentified	unidentified	unidentified	unidentified	unidentified	2E-68	260	178	95.51	4	5
OTU789	JF718364	Fungi	unidentified	unidentified	unidentified	unidentified	unidentified	1E-69	264	179	96.09	5	7
OTU734	FJ770078	Fungi	unidentified	unidentified	unidentified	unidentified	unidentified	2E-30	133	139	89.21	3	3
OTU801	KC978007	Fungi	unidentified	unidentified	unidentified	unidentified	unidentified	1E-88	327	216	95.37	3	4
OTU771	AM901803	Fungi	unidentified	unidentified	unidentified	unidentified	unidentified	2E-64	246	144	97.92	2	2
OTU777	FJ627262	Fungi	unidentified	unidentified	unidentified	unidentified	unidentified	4E-85	315	198	96.46	3	4
OTU802	KC978007	Fungi	unidentified	unidentified	unidentified	unidentified	unidentified	2E-96	353	221	96.83	4	5
OTU790	JN596348	Fungi	unidentified	unidentified	unidentified	unidentified	unidentified	5E-56	218	153	94.77	3	4
OTU733	EU035415	Fungi	unidentified	unidentified	unidentified	unidentified	unidentified	3E-49	196	199	89.95	6	10
OTU744	JN904871	Fungi	unidentified	unidentified	unidentified	unidentified	unidentified	4E-25	115	74	95.95	1	1
OTU727	AB042224	Fungi	unidentified	unidentified	unidentified	unidentified	unidentified	3E-20	99.6	103	88.35	2	5
OTU775	EU516831	Fungi	unidentified	unidentified	unidentified	unidentified	unidentified	2E-74	280	145	99.31	0	0
OTU813	NR_073218	Fungi	unidentified	unidentified	unidentified	unidentified	unidentified	1E-52	206	104	100	0	0
OTU760	KM246166	Fungi	unidentified	unidentified	unidentified	unidentified	unidentified	3E-23	109	144	86.81	4	7
OTU796	JX998717	Fungi	unidentified	unidentified	unidentified	unidentified	unidentified	1E-56	220	150	96	4	5
OTU742	GU981736	Fungi	unidentified	unidentified	unidentified	unidentified	unidentified	3E-95	349	208	96.63	1	1
OTU778	FJ758367	Fungi	unidentified	unidentified	unidentified	unidentified	unidentified	2E-67	256	144	97.92	1	2
OTU781	GQ515633	Fungi	unidentified	unidentified	unidentified	unidentified	unidentified	1E-75	283	163	96.93	0	0

OTU769	AF437776	Fungi	unidentified	unidentified	unidentified	unidentified	unidentified	5E-47	188	155	91.61	3	7
OTU785	HQ731642	Fungi	unidentified	unidentified	unidentified	unidentified	unidentified	5E-56	218	150	96	4	4
OTU795	JX998699	Fungi	unidentified	unidentified	unidentified	unidentified	unidentified	5E-53	208	141	94.33	1	1
OTU798	KC354795	Fungi	unidentified	unidentified	unidentified	unidentified	unidentified	1E-56	220	143	94.41	0	0
OTU804	KF445073	Fungi	Zygomycota	Incertae	Mortierellales	Mortierellaceae	*Mortierella*	8E-96	351	216	96.76	3	4
OTU811	KM246232	Fungi	Zygomycota	Incertae	Mortierellales	Mortierellaceae	*Mortierella*	3E-42	172	123	94.31	2	2
OTU815	NR_103620	Fungi	Zygomycota	Incertae	Mortierellales	Mortierellaceae	*Mortierella*	1E-88	327	173	99.42	1	1
OTU806	KJ775706	Fungi	Zygomycota	Incertae	Mortierellales	Mortierellaceae	*Mortierella*	2E-93	343	177	99.44	0	0
OTU808	KM051384	Fungi	Zygomycota	Incertae	Mortierellales	Mortierellaceae	*Mortierella*	2E-77	289	162	98.15	1	1
OTU812	KM458799	Fungi	Zygomycota	Incertae	Mortierellales	Mortierellaceae	*Mortierella*	1E-85	317	175	98.29	1	2
OTU807	KM051384	Fungi	Zygomycota	Incertae	Mortierellales	Mortierellaceae	*Mortierella*	4E-51	202	157	94.27	5	6
OTU809	KM189445	Fungi	Zygomycota	Incertae	Mucorales	Mucoraceae	*Mucor*	4E-82	305	174	97.7	1	1
OTU810	KM222495	Fungi	Zygomycota	Incertae	Mucorales	Mucoraceae	*Mucor*	5E-78	291	178	96.63	2	3
OTU817		unknown											
OTU818		unknown											
OTU819		unknown											
OTU820		unknown											
OTU821		unknown											
OTU822		unknown											
OTU823		unknown											
OTU824		unknown											
OTU825		unknown											
OTU826		unknown											
OTU827		unknown											
OTU828		unknown											
OTU829		unknown											

OTU830	unknown
OTU831	unknown
OTU832	unknown
OTU833	unknown
OTU834	unknown
OTU835	unknown
OTU836	unknown
OTU837	unknown
OTU838	unknown
OTU839	unknown
OTU840	unknown
OTU841	unknown
OTU842	unknown
OTU843	unknown
OTU844	unknown
OTU845	unknown
OTU846	unknown
OTU847	unknown
OTU848	unknown
OTU849	unknown
OTU850	unknown
OTU851	unknown
OTU852	unknown
OTU853	unknown
OTU854	unknown
OTU855	unknown

OTU856	unknown
OTU857	unknown
OTU858	unknown
OTU859	unknown
OTU860	unknown
OTU861	unknown
OTU862	unknown
OTU863	unknown
OTU864	unknown
OTU865	unknown
OTU866	unknown
OTU867	unknown
OTU868	unknown
OTU869	unknown
OTU870	unknown
OTU871	unknown
OTU872	unknown
OTU873	unknown
OTU874	unknown
OTU875	unknown
OTU876	unknown
OTU877	unknown
OTU878	unknown
OTU879	unknown
OTU880	unknown
OTU881	unknown

OTU882	unknown
OTU883	unknown
OTU884	unknown
OTU885	unknown
OTU886	unknown
OTU887	unknown
OTU888	unknown
OTU889	unknown
OTU890	unknown
OTU891	unknown
OTU892	unknown
OTU893	unknown
OTU894	unknown
OTU895	unknown
OTU896	unknown
OTU897	unknown
OTU898	unknown
OTU899	unknown
OTU900	unknown
OTU901	unknown
OTU902	unknown
OTU903	unknown
OTU904	unknown
OTU905	unknown
OTU906	unknown
OTU907	unknown

OTU908	unknown
OTU909	unknown
OTU910	unknown
OTU911	unknown
OTU912	unknown
OTU913	unknown
OTU914	unknown
OTU915	unknown
OTU916	unknown
OTU917	unknown
OTU918	unknown
OTU919	unknown
OTU920	unknown
OTU921	unknown
OTU922	unknown
OTU923	unknown
OTU924	unknown
OTU925	unknown
OTU926	unknown
OTU927	unknown
OTU928	unknown
OTU929	unknown
OTU930	unknown
OTU931	unknown
OTU932	unknown
OTU933	unknown

OTU934	unknown
OTU935	unknown
OTU936	unknown
OTU937	unknown
OTU938	unknown
OTU939	unknown
OTU940	unknown
OTU941	unknown
OTU942	unknown
OTU943	unknown
OTU944	unknown
OTU945	unknown
OTU946	unknown
OTU947	unknown
OTU948	unknown
OTU949	unknown
OTU950	unknown
OTU951	unknown
OTU952	unknown
OTU953	unknown
OTU954	unknown
OTU955	unknown
OTU956	unknown
OTU957	unknown
OTU958	unknown
OTU959	unknown

OTU960	unknown
OTU961	unknown
OTU962	unknown
OTU963	unknown
OTU964	unknown
OTU965	unknown
OTU966	unknown
OTU967	unknown
OTU968	unknown
OTU969	unknown
OTU970	unknown
OTU971	unknown
OTU972	unknown
OTU973	unknown
OTU974	unknown
OTU975	unknown
OTU976	unknown
OTU977	unknown
OTU978	unknown
OTU979	unknown
OTU980	unknown
OTU981	unknown
OTU982	unknown
OTU983	unknown
OTU984	unknown
OTU985	unknown

OTU986	unknown
OTU987	unknown
OTU988	unknown
OTU989	unknown
OTU990	unknown
OTU991	unknown
OTU992	unknown
OTU993	unknown
OTU994	unknown
OTU995	unknown
OTU996	unknown
OTU997	unknown
OTU998	unknown
OTU999	unknown
OTU1000	unknown
OTU1001	unknown
OTU1002	unknown
OTU1003	unknown
OTU1004	unknown
OTU1005	unknown
OTU1006	unknown
OTU1007	unknown
OTU1008	unknown
OTU1009	unknown
OTU1010	unknown
OTU1011	unknown

OTU1012	unknown
OTU1013	unknown
OTU1014	unknown
OTU1015	unknown
OTU1016	unknown
OTU1017	unknown
OTU1018	unknown
OTU1019	unknown
OTU1020	unknown
OTU1021	unknown
OTU1022	unknown
OTU1023	unknown
OTU1024	unknown
OTU1025	unknown
OTU1026	unknown
OTU1027	unknown
OTU1028	unknown
OTU1029	unknown
OTU1030	unknown
OTU1031	unknown
OTU1032	unknown
OTU1033	unknown
OTU1034	unknown
OTU1035	unknown
OTU1036	unknown
OTU1037	unknown

OTU1038	unknown
OTU1039	unknown
OTU1040	unknown
OTU1041	unknown
OTU1042	unknown
OTU1043	unknown
OTU1044	unknown
OTU1045	unknown
OTU1046	unknown
OTU1047	unknown
OTU1048	unknown
OTU1049	unknown
OTU1050	unknown
OTU1051	unknown
OTU1052	unknown
OTU1053	unknown
OTU1054	unknown
OTU1055	unknown
OTU1056	unknown
OTU1057	unknown
OTU1058	unknown
OTU1059	unknown
OTU1060	unknown
OTU1061	unknown
OTU1062	unknown
OTU1063	unknown

OTU1064	unknown
OTU1065	unknown
OTU1066	unknown
OTU1067	unknown
OTU1068	unknown
OTU1069	unknown
OTU1070	unknown
OTU1071	unknown
OTU1072	unknown
OTU1073	unknown
OTU1074	unknown
OTU1075	unknown
OTU1076	unknown
OTU1077	unknown
OTU1078	unknown
OTU1079	unknown
OTU1080	unknown
OTU1081	unknown
OTU1082	unknown
OTU1083	unknown
OTU1084	unknown
OTU1085	unknown
OTU1086	unknown
OTU1087	unknown
OTU1088	unknown
OTU1089	unknown

OTU1090	unknown
OTU1091	unknown
OTU1092	unknown
OTU1093	unknown
OTU1094	unknown
OTU1095	unknown
OTU1096	unknown
OTU1097	unknown
OTU1098	unknown
OTU1099	unknown
OTU1100	unknown
OTU1101	unknown
OTU1102	unknown
OTU1103	unknown
OTU1104	unknown
OTU1105	unknown
OTU1106	unknown
OTU1107	unknown
OTU1108	unknown
OTU1109	unknown
OTU1110	unknown
OTU1111	unknown
OTU1112	unknown
OTU1113	unknown
OTU1114	unknown
OTU1115	unknown

OTU1116	unknown
OTU1117	unknown
OTU1118	unknown
OTU1119	unknown
OTU1120	unknown
OTU1121	unknown
OTU1122	unknown
OTU1123	unknown
OTU1124	unknown
OTU1125	unknown
OTU1126	unknown
OTU1127	unknown
OTU1128	unknown
OTU1129	unknown
OTU1130	unknown
OTU1131	unknown
OTU1132	unknown
OTU1133	unknown
OTU1134	unknown
OTU1135	unknown
OTU1136	unknown
OTU1137	unknown
OTU1138	unknown
OTU1139	unknown
OTU1140	unknown
OTU1141	unknown

OTU1142	unknown
OTU1143	unknown
OTU1144	unknown
OTU1145	unknown
OTU1146	unknown
OTU1147	unknown
OTU1148	unknown
OTU1149	unknown
OTU1150	unknown
OTU1151	unknown
OTU1152	unknown
OTU1153	unknown
OTU1154	unknown
OTU1155	unknown
OTU1156	unknown
OTU1157	unknown
OTU1158	unknown
OTU1159	unknown
OTU1160	unknown
OTU1161	unknown
OTU1162	unknown
OTU1163	unknown
OTU1164	unknown
OTU1165	unknown
OTU1166	unknown
OTU1167	unknown

OTU1168	unknown
OTU1169	unknown
OTU1170	unknown
OTU1171	unknown
OTU1172	unknown
OTU1173	unknown
OTU1174	unknown
OTU1175	unknown
OTU1176	unknown
OTU1177	unknown
OTU1178	unknown
OTU1179	unknown
OTU1180	unknown
OTU1181	unknown
OTU1182	unknown
OTU1183	unknown
OTU1184	unknown
OTU1185	unknown
OTU1186	unknown
OTU1187	unknown
OTU1188	unknown
OTU1189	unknown
OTU1190	unknown
OTU1191	unknown
OTU1192	unknown
OTU1193	unknown

OTU1194	unknown
OTU1195	unknown
OTU1196	unknown
OTU1197	unknown
OTU1198	unknown
OTU1199	unknown
OTU1200	unknown
OTU1201	unknown
OTU1202	unknown
OTU1203	unknown
OTU1204	unknown
OTU1205	unknown
OTU1206	unknown
OTU1207	unknown
OTU1208	unknown
OTU1209	unknown
OTU1210	unknown
OTU1211	unknown
OTU1212	unknown
OTU1213	unknown
OTU1214	unknown
OTU1215	unknown
OTU1216	unknown
OTU1217	unknown
OTU1218	unknown
OTU1219	unknown

OTU1220	unknown
OTU1221	unknown
OTU1222	unknown
OTU1223	unknown
OTU1224	unknown
OTU1225	unknown
OTU1226	unknown
OTU1227	unknown
OTU1228	unknown
OTU1229	unknown
OTU1230	unknown
OTU1231	unknown
OTU1232	unknown
OTU1233	unknown
OTU1234	unknown
OTU1235	unknown
OTU1236	unknown
OTU1237	unknown
OTU1238	unknown
OTU1239	unknown
OTU1240	unknown
OTU1241	unknown
OTU1242	unknown
OTU1243	unknown
OTU1244	unknown
OTU1245	unknown

OTU1246	unknown
OTU1247	unknown
OTU1248	unknown
OTU1249	unknown
OTU1250	unknown
OTU1251	unknown
OTU1252	unknown
OTU1253	unknown
OTU1254	unknown
OTU1255	unknown
OTU1256	unknown
OTU1257	unknown
OTU1258	unknown
OTU1259	unknown
OTU1260	unknown
OTU1261	unknown
OTU1262	unknown
OTU1263	unknown
OTU1264	unknown
OTU1265	unknown
OTU1266	unknown
OTU1267	unknown
OTU1268	unknown
OTU1269	unknown
OTU1270	unknown
OTU1271	unknown

OTU1272	unknown
OTU1273	unknown
OTU1274	unknown
OTU1275	unknown
OTU1276	unknown
OTU1277	unknown
OTU1278	unknown
OTU1279	unknown
OTU1280	unknown
OTU1281	unknown
OTU1282	unknown
OTU1283	unknown
OTU1284	unknown
OTU1285	unknown
OTU1286	unknown
OTU1287	unknown
OTU1288	unknown
OTU1289	unknown
OTU1290	unknown
OTU1291	unknown
OTU1292	unknown
OTU1293	unknown
OTU1294	unknown
OTU1295	unknown
OTU1296	unknown
OTU1297	unknown

OTU1298	unknown
OTU1299	unknown
OTU1300	unknown
OTU1301	unknown
OTU1302	unknown
OTU1303	unknown
OTU1304	unknown
OTU1305	unknown
OTU1306	unknown
OTU1307	unknown
OTU1308	unknown
OTU1309	unknown
OTU1310	unknown
OTU1311	unknown
OTU1312	unknown
OTU1313	unknown
OTU1314	unknown
OTU1315	unknown
OTU1316	unknown
OTU1317	unknown
OTU1318	unknown
OTU1319	unknown
OTU1320	unknown
OTU1321	unknown
OTU1322	unknown
OTU1323	unknown

OTU1324	unknown
OTU1325	unknown
OTU1326	unknown
OTU1327	unknown
OTU1328	unknown
OTU1329	unknown
OTU1330	unknown
OTU1331	unknown
OTU1332	unknown
OTU1333	unknown
OTU1334	unknown
OTU1335	unknown
OTU1336	unknown
OTU1337	unknown
OTU1338	unknown
OTU1339	unknown
OTU1340	unknown
OTU1341	unknown
OTU1342	unknown
OTU1343	unknown
OTU1344	unknown
OTU1345	unknown
OTU1346	unknown
OTU1347	unknown
OTU1348	unknown
OTU1349	unknown

OTU1350	unknown
OTU1351	unknown
OTU1352	unknown
OTU1353	unknown
OTU1354	unknown
OTU1355	unknown
OTU1356	unknown
OTU1357	unknown
OTU1358	unknown
OTU1359	unknown
OTU1360	unknown
OTU1361	unknown
OTU1362	unknown
OTU1363	unknown
OTU1364	unknown
OTU1365	unknown
OTU1366	unknown
OTU1367	unknown
OTU1368	unknown
OTU1369	unknown
OTU1370	unknown
OTU1371	unknown
OTU1372	unknown
OTU1373	unknown
OTU1374	unknown
OTU1375	unknown

OTU1376	unknown
OTU1377	unknown
OTU1378	unknown
OTU1379	unknown
OTU1380	unknown
OTU1381	unknown
OTU1382	unknown
OTU1383	unknown
OTU1384	unknown
OTU1385	unknown
OTU1386	unknown
OTU1387	unknown
OTU1388	unknown
OTU1389	unknown
OTU1390	unknown
OTU1391	unknown

Supplementary Table S4. Relative abundances of read and OTU counts per site at class level.

**Relative abundances of read counts**																															
**Phylum**	Class	1	2	3	4	5	6	7	8	9	10	11	12	13	14	15	16	17	18	19	20	21	22	23	24	25	26	27	28	29	30
**Ascomycota**	Dothideomycetes	33.06	32.40	30.30	30.37	29.71	31.06	29.35	28.87	26.87	32.84	23.25	33.33	28.97	26.64	26.56	25.88	27.67	29.04	27.94	30.22	25.66	27.81	32.14	27.37	32.63	26.07	27.65	30.18	30.05	37.53
**Ascomycota**	Eurotiomycetes	26.50	29.14	26.77	28.66	26.42	27.29	26.59	29.66	30.58	24.85	32.61	22.35	29.10	31.27	30.55	31.38	27.57	31.81	29.75	27.14	30.44	28.21	25.05	26.78	25.41	27.55	32.09	29.73	27.98	22.87
**Ascomycota**	Incertae	0.00	0.00	0.00	0.00	0.05	0.00	0.00	0.00	0.03	0.00	0.00	0.03	0.04	0.03	0.00	0.00	0.00	0.00	0.00	0.00	0.00	0.00	0.00	0.00	0.00	0.00	0.00	0.00	0.00	0.00
**Ascomycota**	Leotiomycetes	0.23	0.14	0.00	0.00	0.00	0.11	0.00	0.28	0.07	0.07	0.47	0.13	0.00	0.00	0.07	0.03	0.13	0.00	0.15	0.17	0.03	0.04	0.00	0.00	0.19	0.00	0.00	0.00	0.12	0.06
**Ascomycota**	Saccharomycetes	0.73	1.93	0.39	1.60	0.90	0.83	1.70	1.13	1.28	0.85	0.94	0.60	0.79	0.42	0.83	0.99	2.13	0.43	1.14	0.97	0.38	0.84	0.38	1.05	1.64	1.76	1.29	0.77	1.00	1.01

**Ascomycota**	Sordariomycetes	17.74	17.35	20.91	15.80	21.78	22.43	20.79	18.38	18.32	19.16	19.89	19.55	19.88	19.62	21.58	17.36	19.91	19.30	20.19	18.71	18.94	17.55	18.69	17.19	18.00	20.57	18.38	18.42	18.10	17.21
**Ascomycota**	Taphrinomycetes	0.33	0.14	0.34	0.18	0.18	0.15	0.18	0.18	0.16	0.17	0.00	0.03	0.25	0.09	0.07	0.10	0.23	0.06	0.06	0.17	0.16	0.04	0.04	0.12	0.11	0.14	0.11	0.18	0.17	0.09
**Ascomycota**	A_unidentified	0.35	0.05	0.06	0.07	0.18	0.04	0.18	0.06	0.07	0.14	0.12	0.22	0.04	0.12	0.13	0.03	0.10	0.18	0.20	0.06	0.10	0.09	0.04	0.00	0.04	0.23	0.11	0.15	0.21	0.15
**Basidiomycota**	Agaricomycetes	2.05	2.29	2.85	2.83	2.34	3.32	2.67	2.60	3.35	1.49	2.35	2.36	4.09	3.02	2.66	2.49	3.92	2.22	1.93	3.22	2.42	2.89	2.56	2.22	1.26	1.85	2.09	1.95	2.57	1.48
**Basidiomycota**	Agaricostilbomycetes	0.08	0.00	0.00	0.00	0.05	0.00	0.09	0.00	0.00	0.00	0.00	0.00	0.08	0.06	0.00	0.03	0.00	0.00	0.15	0.00	0.00	0.00	0.17	0.00	0.11	0.00	0.08	0.04	0.00	0.00
**Basidiomycota**	Cystobasidiomycetes	1.23	1.28	1.17	0.87	1.94	0.87	1.89	2.08	0.82	1.08	1.65	1.40	1.54	1.34	1.66	2.12	1.28	2.22	1.37	2.05	2.49	2.27	2.52	1.29	2.48	2.96	1.41	1.81	2.49	1.84
**Basidiomycota**	Microbotryomycetes	1.70	1.79	1.06	1.20	2.12	1.70	1.79	1.07	1.87	2.47	1.29	3.18	2.88	2.99	1.86	3.08	1.70	1.91	2.48	2.74	2.26	1.73	2.60	1.99	1.07	1.16	0.42	1.44	0.95	1.63
**Basidiomycota**	Pucciniomycetes	0.00	0.00	0.06	0.07	0.00	0.00	0.00	0.00	0.00	0.00	0.12	0.10	0.00	0.03	0.03	0.07	0.00	0.00	0.00	0.00	0.00	0.13	0.00	0.00	0.00	0.00	0.00	0.15	0.00	0.00
**Basidiomycota**	Tremellomycetes	2.28	2.43	3.58	6.90	5.50	2.79	4.55	4.86	4.74	5.72	5.53	5.28	3.00	3.32	3.13	3.15	2.58	2.90	3.10	3.02	2.87	4.18	2.56	7.25	4.17	4.11	2.47	2.76	2.99	2.81
**Basidiomycota**	unidentified	0.13	0.41	0.11	0.00	0.14	0.00	0.05	0.28	0.07	0.07	0.00	0.10	0.00	0.15	0.00	0.07	0.13	0.25	0.00	0.00	0.00	0.13	0.04	0.00	0.04	0.00	0.08	0.04	0.00	0.09
**Basidiomycota**	Wallemiomycetes	0.00	0.05	0.00	0.04	0.00	0.00	0.05	0.03	0.00	0.14	0.00	0.00	0.00	0.03	0.00	0.00	0.03	0.12	0.00	0.19	0.29	0.00	0.00	0.00	0.00	0.09	0.15	0.00	0.00	0.03
**Chytridiomycota**	Monoblepharidomycetes	0.00	0.00	0.00	0.00	0.00	0.00	0.00	0.00	0.00	0.00	0.00	0.00	0.00	0.09	0.00	0.30	0.23	0.00	0.15	0.00	0.00	0.00	0.04	0.00	0.15	0.00	0.11	0.00	0.00	0.18
**Chytridiomycota**	unidentified	0.60	0.37	0.61	0.91	0.14	0.49	0.23	0.31	0.72	0.71	0.35	0.45	0.25	0.24	0.40	0.17	0.52	0.37	0.32	0.25	0.38	0.40	0.13	0.35	0.34	0.88	0.99	0.33	0.66	0.36
**Rozellomycota**	unidentified	0.00	0.05	0.00	0.07	0.00	0.04	0.00	0.00	0.00	0.00	0.00	0.00	0.00	0.03	0.03	0.00	0.00	0.00	0.00	0.00	0.00	0.00	0.00	0.00	0.00	0.00	0.00	0.04	0.00	0.00
**unidentified**	unidentified	12.36	9.87	11.29	9.95	8.48	8.56	9.89	9.54	10.98	9.92	11.18	10.76	8.84	10.30	9.97	12.19	11.81	8.82	10.78	10.17	13.20	12.88	12.59	14.27	11.96	12.53	12.19	11.53	12.16	12.17
**Zygomycota**	Incertae	0.65	0.32	0.50	0.47	0.09	0.34	0.00	0.67	0.07	0.34	0.24	0.13	0.25	0.21	0.47	0.56	0.07	0.37	0.29	0.91	0.38	0.80	0.43	0.12	0.38	0.09	0.38	0.48	0.54	0.50
**Total**		100	100	100	100	100	100	100	100	100	100	100	100	100	100	100	100	100	100	100	100	100	100	100	100	100	100	100	100	100	100
**Relative abundances of read counts**																															
**Phylum**	Class	1	2	3	4	5	6	7	8	9	10	11	12	13	14	15	16	17	18	19	20	21	22	23	24	25	26	27	28	29	30
**Ascomycota**	Dothideomycetes	24.55	23.21	24.31	24.40	22.09	25.72	21.47	22.44	23.10	20.76	22.09	22.52	20.54	23.35	22.99	21.41	22.29	19.22	22.89	24.38	22.16	20.00	22.88	18.48	22.74	22.01	21.36	20.72	21.57	24.73
**Ascomycota**	Eurotiomycetes	28.64	29.01	25.35	24.70	27.91	27.75	27.24	26.42	28.26	24.56	27.13	26.27	29.17	27.47	29.02	28.46	26.57	32.38	26.32	25.75	26.42	25.81	24.45	23.22	24.20	22.96	26.41	24.03	26.89	25.27
**Ascomycota**	Incertae	0.00	0.00	0.00	0.00	0.29	0.00	0.00	0.00	0.27	0.00	0.00	0.27	0.30	0.27	0.00	0.00	0.00	0.00	0.00	0.00	0.00	0.00	0.00	0.00	0.00	0.00	0.00	0.00	0.00	0.00
**Ascomycota**	Leotiomycetes	1.02	0.34	0.00	0.00	0.00	0.87	0.00	0.28	0.27	0.58	1.16	0.80	0.00	0.00	0.29	0.27	0.29	0.00	0.53	0.55	0.28	0.32	0.00	0.00	0.58	0.00	0.00	0.00	0.56	0.27
**Ascomycota**	Saccharomycetes	1.53	2.05	0.35	1.81	1.45	1.73	2.24	2.27	1.63	2.05	1.16	1.07	1.79	1.10	1.72	1.90	2.57	0.71	2.37	1.37	1.42	1.61	1.57	2.37	3.21	2.20	2.08	1.66	2.24	1.86
**Ascomycota**	Sordariomycetes	18.93	19.80	22.22	17.77	21.22	20.81	21.15	19.32	19.84	21.64	19.77	21.18	20.24	18.96	22.41	17.89	19.71	20.28	18.68	21.37	20.45	20.97	21.32	20.38	20.12	18.24	21.07	20.99	20.17	19.68
**Ascomycota**	Taphrinomycetes	0.26	0.34	0.35	0.30	0.29	0.29	0.32	0.28	0.27	0.29	0.00	0.27	0.30	0.27	0.29	0.27	0.29	0.36	0.26	0.27	0.28	0.32	0.31	0.47	0.29	0.31	0.30	0.28	0.28	0.27

**Ascomycota**	unidentified	0.51	0.34	0.35	0.30	0.58	0.29	0.64	0.57	0.54	0.88	0.78	1.07	0.30	0.82	0.86	0.27	0.29	0.71	1.05	0.55	0.57	0.32	0.31	0.00	0.29	0.94	0.59	1.10	0.56	0.80
**Basidiomycota**	Agaricomycetes	2.56	3.41	4.17	4.22	2.91	3.76	3.85	3.13	2.99	2.92	3.10	4.83	4.17	3.02	3.45	4.34	4.57	2.49	3.95	4.38	3.98	2.58	3.13	4.74	2.62	4.72	3.86	2.21	3.92	2.66
**Basidiomycota**	Agaricostilbomycetes	0.26	0.00	0.00	0.00	0.29	0.00	0.32	0.00	0.00	0.00	0.00	0.00	0.30	0.27	0.00	0.27	0.00	0.00	0.26	0.00	0.00	0.00	0.31	0.00	0.29	0.00	0.30	0.28	0.00	0.00
**Basidiomycota**	Cystobasidiomycetes	3.07	2.39	2.43	2.11	3.78	2.60	3.21	2.27	2.45	2.92	3.88	3.22	2.68	2.75	2.59	3.25	3.14	3.91	3.42	2.19	3.69	4.52	3.45	3.32	3.50	4.40	3.56	4.42	3.64	2.93
**Basidiomycota**	Microbotryomycetes	2.30	2.39	2.78	2.11	2.91	2.31	2.24	1.70	2.45	2.63	2.33	1.61	2.68	2.75	2.30	2.44	2.00	2.14	2.63	1.92	2.27	1.29	1.57	2.37	2.33	2.20	2.08	2.49	1.96	2.39
**Basidiomycota**	Pucciniomycetes	0.00	0.00	0.35	0.30	0.00	0.00	0.00	0.00	0.00	0.00	0.39	0.27	0.00	0.27	0.29	0.27	0.00	0.00	0.00	0.00	0.00	0.32	0.00	0.00	0.00	0.00	0.00	0.55	0.00	0.00
**Basidiomycota**	Tremellomycetes	4.35	4.10	4.17	10.24	6.69	4.62	5.77	6.25	6.52	7.89	5.81	5.09	5.95	6.04	3.74	5.96	5.71	6.41	5.00	6.03	5.11	6.77	6.58	9.95	6.41	8.49	4.75	6.91	7.00	6.65
**Basidiomycota**	unidentified	0.51	0.68	0.35	0.00	0.29	0.00	0.32	0.85	0.27	0.29	0.00	0.27	0.00	0.82	0.00	0.54	0.57	0.71	0.00	0.00	0.00	0.65	0.31	0.00	0.29	0.00	0.59	0.28	0.00	0.27
**Basidiomycota**	Wallemiomycetes	0.00	0.34	0.00	0.30	0.00	0.00	0.32	0.28	0.00	0.58	0.00	0.00	0.00	0.27	0.00	0.00	0.29	0.36	0.00	0.27	0.28	0.00	0.00	0.00	0.00	0.31	0.30	0.00	0.00	0.27
**Chytridiomycota**	Monoblepharidomycetes	0.00	0.00	0.00	0.00	0.00	0.00	0.00	0.00	0.00	0.00	0.00	0.00	0.00	0.27	0.00	0.27	0.29	0.00	0.26	0.00	0.00	0.00	0.31	0.00	0.29	0.00	0.30	0.00	0.00	0.27
**Chytridiomycota**	unidentified	0.26	0.34	0.35	0.30	0.29	0.29	0.32	0.28	0.27	0.29	0.39	0.27	0.30	0.27	0.29	0.27	0.29	0.36	0.26	0.27	0.28	0.32	0.31	0.47	0.29	0.31	0.30	0.28	0.28	0.27
**Rozellomycota**	unidentified	0.00	0.34	0.00	0.30	0.00	0.29	0.00	0.00	0.00	0.00	0.00	0.00	0.00	0.27	0.29	0.00	0.00	0.00	0.00	0.00	0.00	0.00	0.00	0.00	0.00	0.00	0.00	0.28	0.00	0.00
**unidentified**	unidentified	9.72	10.24	11.11	9.64	8.72	7.23	10.58	12.22	10.60	10.82	11.24	10.19	10.42	10.44	8.62	11.11	10.86	9.25	11.05	9.59	11.36	12.90	12.23	13.74	11.37	12.26	11.57	12.43	10.08	10.37
**Zygomycota**	Incertae	1.53	0.68	1.39	1.20	0.29	1.45	0.00	1.42	0.27	0.88	0.78	0.80	0.89	0.27	0.86	0.81	0.29	0.71	1.05	1.10	1.42	1.29	0.94	0.47	1.17	0.63	0.59	1.10	0.84	1.06
**Total**		100	100	100	100	100	100	100	100	100	100	100	100	100	100	100	100	100	100	100	100	100	100	100	100	100	100	100	100	100	100

Supplementary Table S5. Read and OTU numbers (and relative abundance) of 130 identified genera.

**Genus**	**OTUs**	**Sequences**	**percent of OTUs**	**percent of Sequences**
Shiraia	25	7052	3.06372549	8.831890992
Perenniporia	14	4914	1.715686275	6.154270041
Penicillium	31	4619	3.799019608	5.784813456
Phialemonium	14	4417	1.715686275	5.531829624
Trichomerium	30	3904	3.676470588	4.889350884
Nigrospora	28	2875	3.431372549	3.600636217
Didymosphaeria	4	1893	0.490196078	2.370784125
Erythrobasidium	22	1645	2.696078431	2.060190114
Libertella	10	1528	1.225490196	1.913659875
Sarocladium	11	1320	1.348039216	1.653161672
Ganoderma	14	1207	1.715686275	1.511641013
Rhodotorula	17	1191	2.083333333	1.49160269
Fusarium	15	1128	1.838235294	1.412701792
Mycosphaerella	19	1114	2.328431373	1.395168259
Xylaria	10	1077	1.225490196	1.348829637
Eutypa	14	997	1.715686275	1.24863802
Phialophora	11	962	1.348039216	1.204804188
Alternaria	3	961	0.367647059	1.203551793
Bullera	9	930	1.102941176	1.164727541
Hannaella	7	740	0.857843137	0.926772452
Rhinocladiella	7	702	0.857843137	0.879181434
Cryptococcus	18	698	2.205882353	0.874171854
Dioszegia	4	526	0.490196078	0.658759878
Phyllachora	1	525	0.12254902	0.657507483
Hypoxylon	10	465	1.225490196	0.582363771
Strelitziana	4	464	0.490196078	0.581111376
Cladosporium	9	441	1.102941176	0.552306286
Metschnikowia	11	368	1.348039216	0.460881436
Gliocladium	5	305	0.612745098	0.381980538
Ramichloridium	9	303	1.102941176	0.379475747
Cyphellophora	9	277	1.102941176	0.346913472
Epicoccum	2	250	0.245098039	0.313098801
Arthrinium	2	247	0.245098039	0.309341616
Leptosphaeria	8	236	0.980392157	0.295565269
Zymoseptoria	6	211	0.735294118	0.264255388
Davidiella	1	199	0.12254902	0.249226646
Acrodontium	4	185	0.490196078	0.231693113

Aspergillus	10	183	1.225490196	0.229188323
Kernia	6	158	0.735294118	0.197878443
Phaeosphaeria	4	149	0.490196078	0.186606886
Hypholoma	2	148	0.245098039	0.18535449
Rhizopogon	2	145	0.245098039	0.181597305
Pyrenochaetopsis	2	141	0.245098039	0.176587724
Occultifur	2	140	0.245098039	0.175335329
Acremonium	4	133	0.490196078	0.166568562
Hypocrea	4	125	0.490196078	0.156549401
Aureobasidium	2	124	0.245098039	0.155297006
Trichoderma	5	114	0.612745098	0.142773053
Myriangium	3	108	0.367647059	0.135258682
Ciboria	2	97	0.245098039	0.121482335
Talaromyces	7	96	0.857843137	0.12022994
Phaeosphaeriopsis	3	84	0.367647059	0.105201197
Fellomyces	4	83	0.490196078	0.103948802
Sporobolomyces	4	75	0.490196078	0.09392964
Mortierella	7	74	0.857843137	0.092677245
Capnodium	2	73	0.245098039	0.09142485
Myrothecium	2	71	0.245098039	0.08892006
Paraconiothyrium	4	69	0.490196078	0.086415269
Pseudocercospora	1	66	0.12254902	0.082658084
Eurotium	1	64	0.12254902	0.080153293
Beauveria	2	64	0.245098039	0.080153293
Purpureocillium	1	56	0.12254902	0.070134132
Didymella	3	54	0.367647059	0.067629341
Microascus	3	53	0.367647059	0.066376946
Cordyceps	1	52	0.12254902	0.065124551
Verticillium	1	49	0.12254902	0.061367365
Chaetomium	4	47	0.490196078	0.058862575
Trechispora	1	47	0.12254902	0.058862575
Lophiostoma	2	46	0.245098039	0.057610179
Pichia	2	45	0.245098039	0.056357784
Bionectria	1	44	0.12254902	0.055105389
Dinemasporium	1	43	0.12254902	0.053852994
Stropharia	3	41	0.367647059	0.051348203
Nectria	2	39	0.245098039	0.048843413
Psathyrella	1	39	0.12254902	0.048843413
Coniothyrium	1	37	0.12254902	0.046338623

Massarina	1	34	0.12254902	0.042581437
Colletotrichum	1	33	0.12254902	0.041329042
Wallemia	2	33	0.245098039	0.041329042
Candida	2	32	0.245098039	0.040076647
Coprinopsis	2	32	0.245098039	0.040076647
Peniophora	1	32	0.12254902	0.040076647
Oidiodendron	1	31	0.12254902	0.038824251
Passalora	2	30	0.245098039	0.037571856
Pestalotiopsis	1	30	0.12254902	0.037571856
Guehomyces	1	29	0.12254902	0.036319461
Hyalodendriella	1	26	0.12254902	0.032562275
Coprinellus	2	22	0.245098039	0.027552695
Pseudocercosporella	1	21	0.12254902	0.026300299
Plectosphaerella	1	21	0.12254902	0.026300299
Neosartorya	2	20	0.245098039	0.025047904
Xenomeris	1	19	0.12254902	0.023795509
Pseudeurotium	1	19	0.12254902	0.023795509
Villosiclava	1	19	0.12254902	0.023795509
Annulohypoxylon	1	19	0.12254902	0.023795509
Stachybotrys	1	18	0.12254902	0.022543114
Devriesia	2	17	0.245098039	0.021290718
Phoma	2	17	0.245098039	0.021290718
Chrysosporium	3	17	0.367647059	0.021290718
Cercospora	1	16	0.12254902	0.020038323
Phomopsis	1	16	0.12254902	0.020038323
Sebacina	2	16	0.245098039	0.020038323
Trimorphomyces	2	16	0.245098039	0.020038323
Preussia	2	15	0.245098039	0.018785928
Neurospora	1	15	0.12254902	0.018785928
Psilocybe	1	15	0.12254902	0.018785928
Ophiosphaerella	1	14	0.12254902	0.017533533
Mucor	2	14	0.245098039	0.017533533
Uwebraunia	1	13	0.12254902	0.016281138
Keissleriella	1	13	0.12254902	0.016281138
Sclerostagonospora	1	13	0.12254902	0.016281138
Cercophora	1	13	0.12254902	0.016281138
Stagonospora	1	12	0.12254902	0.015028742
Bipolaris	1	12	0.12254902	0.015028742
Myrmecridium	1	12	0.12254902	0.015028742

Eutypella	1	12	0.12254902	0.015028742
Cystodendron	1	11	0.12254902	0.013776347
Taphrina	1	11	0.12254902	0.013776347
Bensingtonia	1	11	0.12254902	0.013776347
Cylindrocarpon	1	10	0.12254902	0.012523952
Arthrographis	1	8	0.12254902	0.010019162
Metacordyceps	1	8	0.12254902	0.010019162
Phallus	1	7	0.12254902	0.008766766
Derxomyces	1	7	0.12254902	0.008766766
Septoria	1	5	0.12254902	0.006261976
Leptosphaerulina	1	5	0.12254902	0.006261976
Camptophora	1	5	0.12254902	0.006261976
Myrmaecium	1	5	0.12254902	0.006261976
Limonomyces	1	5	0.12254902	0.006261976
Mrakia	1	5	0.12254902	0.006261976

Supplementary Table S6. Data of geographical distance (km) between sampled sites.

site	1	2	3	4	5	6	7	8	9	10	11	12	13	14	15	16	17	18	19	20	21	22	23	24	25	26	27	28	29	30
**1**	0.00	206.36	308.62	159.46	192.58	234.60	311.69	325.49	433.16	423.01	433.16	542.31	534.33	646.37	647.43	762.72	699.67	781.72	732.98	787.65	859.62	888.25	919.62	997.47	890.38	947.99	903.95	839.04	818.58	778.06
**2**	206.36	0.00	102.26	241.72	182.38	120.43	254.19	205.85	398.39	345.78	312.38	487.82	446.41	560.84	539.02	659.66	610.52	700.04	647.40	731.63	840.58	855.02	873.27	941.89	826.34	921.69	881.20	818.27	795.98	751.46
**3**	308.62	102.26	0.00	325.48	250.07	160.24	281.79	204.77	418.23	347.59	289.45	490.35	432.43	541.78	507.03	626.48	587.28	678.14	624.91	723.01	848.42	855.34	866.40	928.87	811.02	924.24	886.32	825.76	803.08	757.68
**4**	159.46	241.72	325.48	0.00	89.22	182.04	185.15	237.64	282.37	290.74	326.29	393.48	399.46	505.95	518.09	627.25	559.35	636.76	590.43	634.72	700.59	730.45	763.64	842.86	738.50	789.30	745.07	680.17	659.90	619.93
**5**	192.58	182.38	250.07	89.22	0.00	95.95	119.11	151.03	248.23	230.43	248.23	353.85	341.75	454.07	455.54	570.19	507.29	590.22	540.93	601.19	687.39	710.11	736.89	812.28	702.49	773.09	730.33	665.86	644.41	601.79
**6**	234.60	120.43	160.24	182.04	95.95	0.00	134.10	96.85	278.10	227.67	208.17	368.33	332.37	447.51	433.02	552.69	498.63	586.80	534.81	613.60	720.16	735.10	754.50	824.50	709.96	801.51	760.86	697.87	675.59	631.12
**7**	311.69	254.19	281.79	185.15	119.11	134.10	0.00	92.29	144.25	111.32	144.25	240.58	222.64	335.29	337.27	451.13	388.40	472.22	422.39	488.24	587.20	604.35	626.82	699.72	587.79	669.76	628.48	565.04	542.92	498.82
**8**	325.49	205.85	204.77	237.64	151.03	96.85	92.29	0.00	214.60	144.25	111.32	287.78	240.58	355.03	336.78	456.84	405.06	494.29	441.76	527.44	645.13	654.85	669.91	736.89	620.84	722.88	683.93	622.53	599.93	554.71
**9**	433.16	398.39	418.23	282.37	248.23	278.10	144.25	214.60	0.00	91.19	182.37	111.32	143.54	230.67	261.35	355.55	282.94	355.47	311.22	354.52	443.30	462.60	488.71	564.86	457.33	526.86	485.02	421.25	399.27	355.59
**10**	423.01	345.78	347.59	290.74	230.43	227.67	111.32	144.25	91.19	0.00	91.19	143.54	111.32	224.61	227.51	339.91	277.47	362.59	311.99	386.04	501.08	510.83	527.50	596.83	482.52	578.68	539.69	478.45	455.82	410.54
**11**	433.16	312.38	289.45	326.29	248.23	208.17	144.25	111.32	182.37	91.19	0.00	212.70	143.54	252.81	226.80	347.35	300.07	390.57	337.45	433.99	567.09	568.99	577.45	639.48	521.80	638.69	602.61	544.39	521.66	476.13
**12**	542.31	487.82	490.35	393.48	353.85	368.33	240.58	287.78	111.32	143.54	212.70	0.00	90.06	126.44	175.76	251.14	175.87	244.31	201.43	247.80	358.11	367.52	386.60	459.24	348.66	435.15	396.23	335.43	312.74	267.33
**13**	534.33	446.41	432.43	399.46	341.75	332.37	222.64	240.58	143.54	111.32	143.54	90.06	0.00	115.17	120.65	228.76	167.06	254.43	202.61	290.60	426.75	425.94	434.03	497.56	380.70	495.90	460.76	404.14	381.51	336.20
**14**	646.37	560.84	541.78	505.95	454.07	447.51	335.29	355.03	230.67	224.61	252.81	126.44	115.17	0.00	75.57	125.26	53.46	139.34	87.52	188.20	344.45	331.45	330.00	387.40	269.28	402.00	371.99	322.85	301.36	258.89
**15**	647.43	539.02	507.03	518.09	455.54	433.02	337.27	336.78	261.35	227.51	226.80	175.76	120.65	75.57	0.00	120.68	90.92	177.53	126.38	250.26	414.37	395.68	386.41	434.18	316.06	465.64	438.85	393.37	372.51	331.38
**16**	762.72	659.66	626.48	627.25	570.19	552.69	451.13	456.84	355.55	339.91	347.35	251.14	228.76	125.26	120.68	0.00	76.72	88.95	69.71	187.59	359.46	324.90	299.99	332.12	218.92	390.48	373.01	341.79	324.68	292.56
**17**	699.67	610.52	587.28	559.35	507.29	498.63	388.40	405.06	282.94	277.47	300.07	175.87	167.06	53.46	90.92	76.72	0.00	90.87	37.66	160.26	327.81	305.69	295.56	345.66	227.14	375.33	349.89	307.57	287.65	249.06
**18**	781.72	700.04	678.14	636.76	590.22	586.80	472.22	494.29	355.47	362.59	390.57	244.31	254.43	139.34	177.53	88.95	90.87	0.00	53.23	100.43	271.18	236.10	214.39	256.67	138.85	302.38	284.07	254.14	237.98	209.14
**19**	732.98	647.40	624.91	590.43	540.93	534.81	422.39	441.76	311.22	311.99	337.45	201.43	202.61	87.52	126.38	69.71	37.66	53.23	0.00	130.51	301.57	274.73	260.73	308.47	190.05	343.50	320.47	282.34	263.61	228.14
**20**	787.65	731.63	723.01	634.72	601.19	613.60	488.24	527.44	354.52	386.04	433.99	247.80	290.60	188.20	250.26	187.59	160.26	100.43	130.51	0.00	172.18	145.47	143.46	211.48	104.70	215.40	190.08	154.20	137.56	110.76
**21**	859.62	840.58	848.42	700.59	687.39	720.16	587.20	645.13	443.30	501.08	567.09	358.11	426.75	344.45	414.37	359.46	327.81	271.18	301.57	172.18	0.00	63.31	125.60	202.51	189.76	90.91	45.55	22.71	45.45	91.00
**22**	888.25	855.02	855.34	730.45	710.11	735.10	604.35	654.85	462.60	510.83	568.99	367.52	425.94	331.45	395.68	324.90	305.69	236.10	274.73	145.47	63.31	0.00	62.29	141.59	133.11	70.57	49.76	67.39	78.10	110.93
**23**	919.62	873.27	866.40	763.64	736.89	754.50	626.82	669.91	488.71	527.50	577.45	386.60	434.03	330.00	386.41	299.99	295.56	214.39	260.73	143.46	125.60	62.29	0.00	85.23	86.70	97.99	103.49	127.74	133.69	155.09
**24**	997.47	941.89	928.87	842.86	812.28	824.50	699.72	736.89	564.86	596.83	639.48	459.24	497.56	387.40	434.18	332.12	345.66	256.67	308.47	211.48	202.51	141.59	85.23	0.00	118.56	141.73	169.05	208.97	217.57	240.24

**25**	890.38	826.34	811.02	738.50	702.49	709.96	587.79	620.84	457.33	482.52	521.80	348.66	380.70	269.28	316.06	218.92	227.14	138.85	190.05	104.70	189.76	133.11	86.70	118.56	0.00	184.20	181.87	182.66	178.11	177.41
**26**	947.99	921.69	924.24	789.30	773.09	801.51	669.76	722.88	526.86	578.68	638.69	435.15	495.90	402.00	465.64	390.48	375.33	302.38	343.50	215.40	90.91	70.57	97.99	141.73	184.20	0.00	45.36	109.51	129.42	171.36
**27**	903.95	881.20	886.32	745.07	730.33	760.86	628.48	683.93	485.02	539.69	602.61	396.23	460.76	371.99	438.85	373.01	349.89	284.07	320.47	190.08	45.55	49.76	103.49	169.05	181.87	45.36	0.00	64.91	85.93	129.74
**28**	839.04	818.27	825.76	680.17	665.86	697.87	565.04	622.53	421.25	478.45	544.39	335.43	404.14	322.85	393.37	341.79	307.57	254.14	282.34	154.20	22.71	67.39	127.74	208.97	182.66	109.51	64.91	0.00	22.74	68.29
**29**	818.58	795.98	803.08	659.90	644.41	675.59	542.92	599.93	399.27	455.82	521.66	312.74	381.51	301.36	372.51	324.68	287.65	237.98	263.61	137.56	45.45	78.10	133.69	217.57	178.11	129.42	85.93	22.74	0.00	45.55
**30**	778.06	751.46	757.68	619.93	601.79	631.12	498.82	554.71	355.59	410.54	476.13	267.33	336.20	258.89	331.38	292.56	249.06	209.14	228.14	110.76	91.00	110.93	155.09	240.24	177.41	171.36	129.74	68.29	45.55	0.00
